# Rapid Assembly of Functionalised Spirocyclic Indolines by Palladium-Catalysed Dearomatising Diallylation of Indoles with Allyl Acetate

**DOI:** 10.1002/chem.201403940

**Published:** 2014-08-29

**Authors:** Persis Dhankher, Laure Benhamou, Tom D Sheppard

**Affiliations:** *[a] Department of Chemistry, University College London, 20 Gordon St, London, WC1H 0AJ (UK)

**Keywords:** allylation, heterocycles, homogeneous catalysis, indoles, palladium

## Abstract

Herein, we report the application of allyl acetate to the palladium-catalysed dearomatising diallylation of indoles. The reaction can be carried out by using a readily available palladium catalyst at room temperature, and can be applied to a wide range of substituted indoles to provide access to the corresponding 3,3-diallylindolinines. These compounds are versatile synthetic intermediates that readily undergo Ugi reactions or proline-catalysed asymmetric Mannich reactions. Alternatively, acylation of the 3,3-diallylindolinines with an acid chloride or a chloroformate, followed by treatment with aluminium chloride, enables 2,3-diallylindoles to be prepared. By using ring-closing metathesis, functionalised spirocyclic indoline scaffolds can be accessed from the Ugi products, and a dihydrocarbazole can be prepared from the corresponding 2,3-diallylindole.

## Introduction

Medicinal chemistry has traditionally focused on the synthesis of aromatic heterocycles and related derivatives as lead compounds due to their drug-like physical properties and synthetic accessibility. However, it is widely considered that an increasing focus on three-dimensional structures, which incorporate a greater proportion of sp^3^ carbons, is probably desirable,^1^ in order for medicinal chemistry programmes to be successful against more complex drug targets. Whilst natural products can provide suitable three-dimensional functionalised architectures, such compounds are often highly complex, difficult to synthesise, and display undesirable physical properties. As a consequence, there is considerable interest in the development of synthetic routes to access small chiral saturated rings including cyclopropanes,^2^ oxetanes^3^–^4^ and azetidines,^5^ as well as benzofused heterocyclic systems, such as indolines^6^ and dihydrobenzofurans.^7^ In addition, the incorporation of these motifs into spirocyclic frameworks can lead to increased molecular complexity whilst maintaining a relatively low molecular weight.^4^, ^8^ Such systems can potentially provide a structurally rigid three-dimensional core, which can be functionalised at several sites to provide a library of drug-like compounds. Herein, we report a short synthetic route to convert readily available indoles into polyfunctionalised spirocyclic indolines,^9^ which enables the incorporation of functional groups at a variety of different positions in the scaffold.

**Scheme 1:**
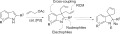
Proposed synthetic route to spirocyclic indolines (RCM=ring-closing metathesis).

We envisaged that introduction of two allyl groups at the 3 position of an indole core, with concomitant dearomatisation, would enable the formation of a diallylindolinine. Such a compound is potentially a very versatile synthetic intermediate that can undergo reactions with a variety of nucleophiles and electrophiles at the imine moiety, cross-coupling/C=H functionalisation reactions on the aromatic ring, and can be readily converted into a spirocyclic alkaloid-like structure by ring-closing metathesis (Scheme 1). Given the large number of indoles that are readily available commercially, a diverse range of functionalised spirocylic scaffolds could readily be constructed. The spirocyclic ring structure embedded in these scaffolds has not been widely explored in existing drugs,^10^ though it forms part of the polycyclic frameworks of the ajmaline alkaloid natural-product family,^11^ which contains compounds possessing antiarrhythmic^12^ and antiplasmodial activity,^13^ some of which have found application in medical treatment.

Our synthetic plan involved the development of a Pd-catalysed allylation procedure to achieve regioselective introduction of the allyl groups in a dearomatising reaction, to generate the desired isoindolinine. Whilst there is good precedent for the Pd-catalysed allylation of indoles with a range of allyl sources,^14^–^18^ and even for the dearomatising allylation of 3-substituted indoles with allyl carbonates, with allyl alcohols in combination with organoboranes^15^ or by rearrangement of *N*-alloc protected indoles,^14^, ^19^–^22^ the direct use of allyl acetate in such reactions has proved challenging to date.^23^ Although highly activated allylic esters containing two conjugated aromatic rings can be successfully used in Pd-catalysed allylation reactions,^24^ there is only a single report of the Pd-catalysed reaction of indole **1 a** with allyl acetate **2**, and the reaction was reported to produce a relatively complex mixture of *N* and *C* allylated products **3 a**–**6 a** from which 3-allylindole **4 a** was isolated in up to 54 % yield.^23^ Allyl acetate is a considerably cheaper starting material than the carbonates and carbamates typically employed, because it is a bulk chemical that can be prepared on an industrial scale by direct acetoxylation of propene.^25^ Therefore, we decided to explore whether it could be successfully employed in the direct Pd-catalysed diallylation of indole.

## Results and Discussion

**Scheme 2:**
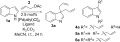
Screening of reaction conditions for the diallylindolinine formation.

An initial test reaction of indole with Pd catalyst, 1,1′-bis(diphenylphosphino)ferrocene (dppf) ligand, allyl acetate **2** and K_2_CO_3_ led to the formation of a mixture of the desired product **3 a**, 3-allylindole **4 a**, as well as traces of 1,3-diallylindole **6 a** (Scheme 2; Table 1, entry 1). By increasing the number of equivalents of allyl acetate, a higher conversion was observed (entry 2), though large quantities of allyl acetate appeared to significantly slow down the reaction (entry 3). In the absence of base, no conversion to either **3 a** or **4 a** was seen (entry 4). By increasing the quantity of base in the reaction to three equivalents, and by employing five equivalents of allyl acetate, almost complete conversion of indole into **3 a** and **4 a** was observed with **3 a** becoming the major product (entry 5). The choice of ligand proved to be critical to controlling both the reactivity and the product distribution. A range of bidentate phosphines were explored, with ethylenebis(diphenylphosphine) (dppe) proving to be very ineffective (entry 6) and both (2,2′-bis(diphenylphosphino)-1,1′-binaphthyl) (BINAP) and 4,5-bis(diphenylphosphino)-9,9-dimethylxanthene (Xantphos) leading to lower conversions and selectivity in comparison to dppf (entries 7 and 8). However, with bis-[2-(diphenylphosphino)phenyl]ether (DPEPhos), we were able to obtain an excellent conversion to the desired diallylindolinine **3 a** with very high selectivity (entry 9). Pleasingly, an 82 % isolated yield of **3 a** could be obtained with only 5 mol % palladium catalyst at room temperature.

**Table 1:** Screening of reaction conditions.

Entry	K_2_CO_3_ [equiv]	2[equiv]	Ligand (5 mol %)	Conv. [%] (yield [%]3 a)	Ratio3 a/4 a^[a]^
1^[b]^	2	1.5	dppf	53 (15)	1:2
2^[b]^	2	2.2	dppf	62	1:1
3^[b]^	2	7	dppf	29	1:5
4^[b]^	0	5	dppf	0	–
5^[c]^	3	5	dppf	>95	2:1^[d]^
6^[c]^	3	5	dppe	13	–
7^[c]^	3	5	*rac*-BINAP	63 (45)	1:1
8^[c]^	3	5	Xantphos	76 (47)	5:3
9^[c]^	3	5	DPEPhos	>95 (82)	>10:1^[d]^

[a] Determined by ^1^H NMR. [b] Reaction was performed at 40 °C. [c] Reaction was performed at RT. [d] Small quantities of **6 a** were also observed.

**Scheme 3:**
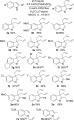
Scope of the Pd-catalysed diallylation reaction. [a]Reaction performed at 50 °C for 8–24 h.

With practical conditions in hand, we went on to explore the scope of this dearomatising double allylation reaction (Scheme 3). A wide range of substituted indoles **1 a–o** were converted to the corresponding diallylindolenines **3 a**–**o** in generally good to excellent yield. Notably, the reaction was tolerant of alkoxy or alkyl substituents at any position on the benzene ring (**3 b**–**g**). However, the allylation reactions of several haloindoles were somewhat sluggish at room temperature, requiring heating at 50 °C in order to obtain reasonable yields of the desired product (**3 h**-.**j**).^26^ Pleasingly, the presence of a substituent at C-2 did not impair the reaction, despite the potential steric crowding around the reaction site (**3 l**–**o**). The presence of a *tert*-butyldimethylsilyl(TBS)-protected alcohol at C-2 was compatible with the reaction, giving a good yield of the diallylindolinine **3 o** considering the presence of this relatively large substituent so close to the newly formed quaternary carbon centre. As was anticipated, electron-rich indoles were generally better substrates for the reaction (**3 b**, **d**, **f**, **m**). In contrast, highly electron-deficient systems did not form the diallylindolinine at all, with the *N*-allylindoles **7 a** and **7 b** being obtained as the major product from reactions of 5-nitroindole and 2-methyl-5-nitroindole, respectively. No allylation or diallylation of *N*-methylindole was observed under these conditions, demonstrating that a free NH is essential for the reaction to take place.

**Scheme 4:**
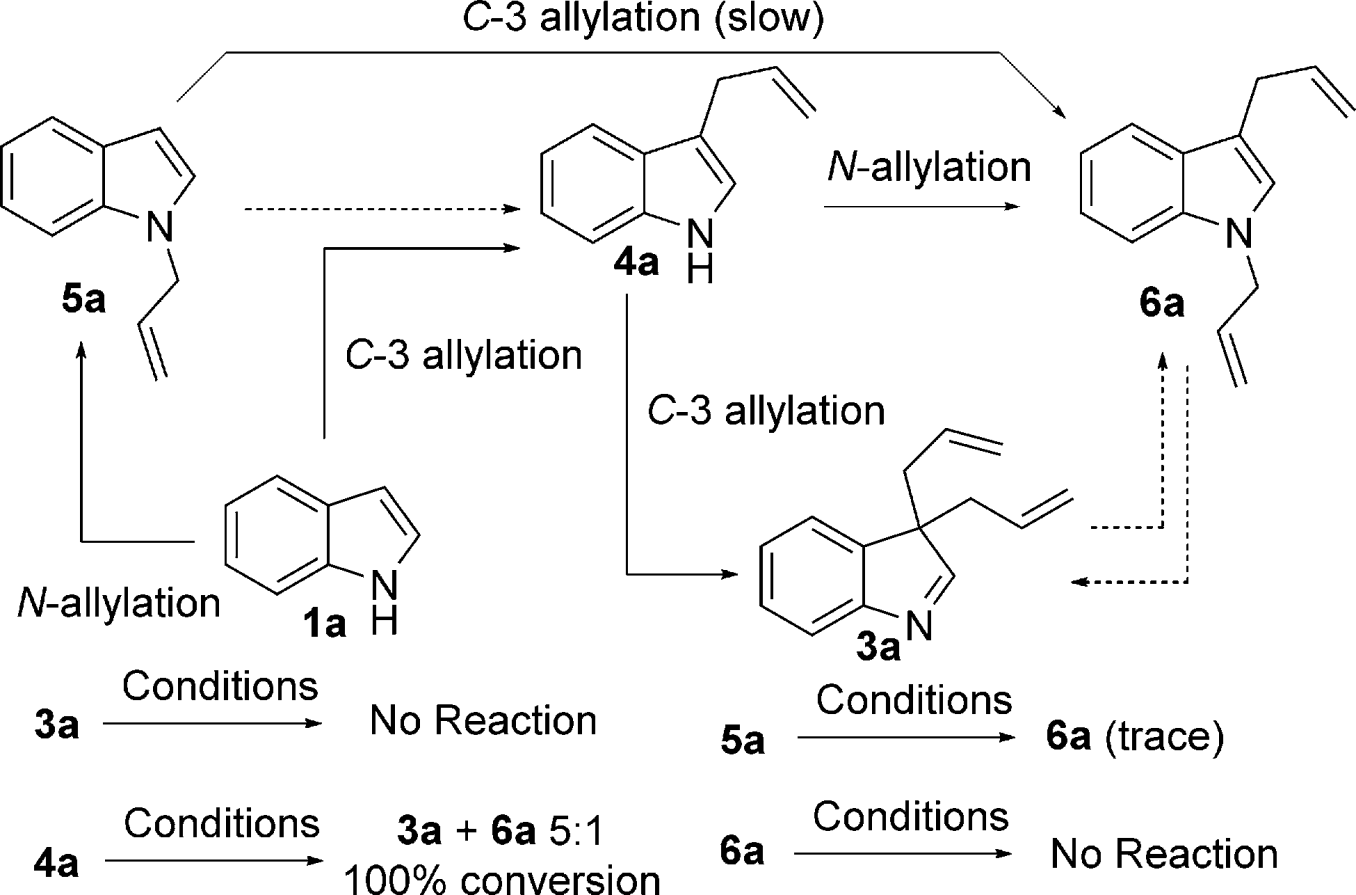
Identification of possible reaction pathways by resubmission of potential intermediates to the reaction conditions. Conditions: [Pd(allyl)Cl]_2_ (2.5 mol %), 2 (5 equiv), DPEPhos (5 mol %), K_2_CO_3_ (3 equiv), MeCN, 24 h, RT.

Given the difficulties initially encountered with achieving selectivity in the indole allylation reaction, we recognised that several different reaction pathways may be operative (Scheme 4). A likely possibility is that the reaction proceeds directly through allylation at C-3 to give **4 a**, followed by a second allylation at C-3 to give the diallylindolinine **3 a**. However, it was also possible that **4 a** might undergo *N*-allylation to give **6 a**, followed by a (potentially Pd-catalysed) rearrangement to generate diallylindolinine **3 a**. Conversely, the by-product **6 a** may be formed by rearrangement of **3 a**. In this latter case, the yield of **3 a** would be eroded during prolonged exposure to the reaction conditions as it was gradually converted into the undesired by-product **6 a**. Furthermore, initial *N*-allylation of **1 a** to give **5 a**, could be followed by (potentially, Pd-catalysed) rearrangement to generate **4 a**. Therefore, we synthesised pure samples of **4 a**,^27^ **5 a**^27^ and **6 a** and resubmitted them to the reaction conditions to determine, which of these compounds, if any, are plausible reaction intermediates. Neither **5 a** nor **6 a** apparently underwent rearrangement under the reaction conditions, but small quantities of **6 a** were produced from **5 a**. This indicates that **5 a** and **6 a** are not plausible intermediates in the formation of diallylindolinine **3 a**. However, 3-allylindole **4 a** was completely converted into a mixture of **3 a** and **6 a** upon resubmission to the reaction conditions. The diallylindolinine **3 a** did not undergo conversion into **6 a** upon resubmission to the reaction conditions, but some degradation of **3 a** did take place suggesting that prolonged exposure of the product to the reaction conditions will have a detrimental effect on the reaction yield. These observations suggest that only **4 a** is an intermediate in the reaction, and that the formation of by-products **5 a** and **6 a** is solely a result of competing *N*-allylation on reaction of **1 a** or **4 a** with the electrophilic π-allyl complex. The use of K_2_CO_3_ as base in related reactions has been reported to promote *N*-allylation over *C*-allylation due to the formation of a looser ion pair,^24^ but this is obviously not a significant factor in our reaction. It should be noted that the use of lithium or sodium carbonates in this diallylation reaction was ineffective.

**Scheme 5:**
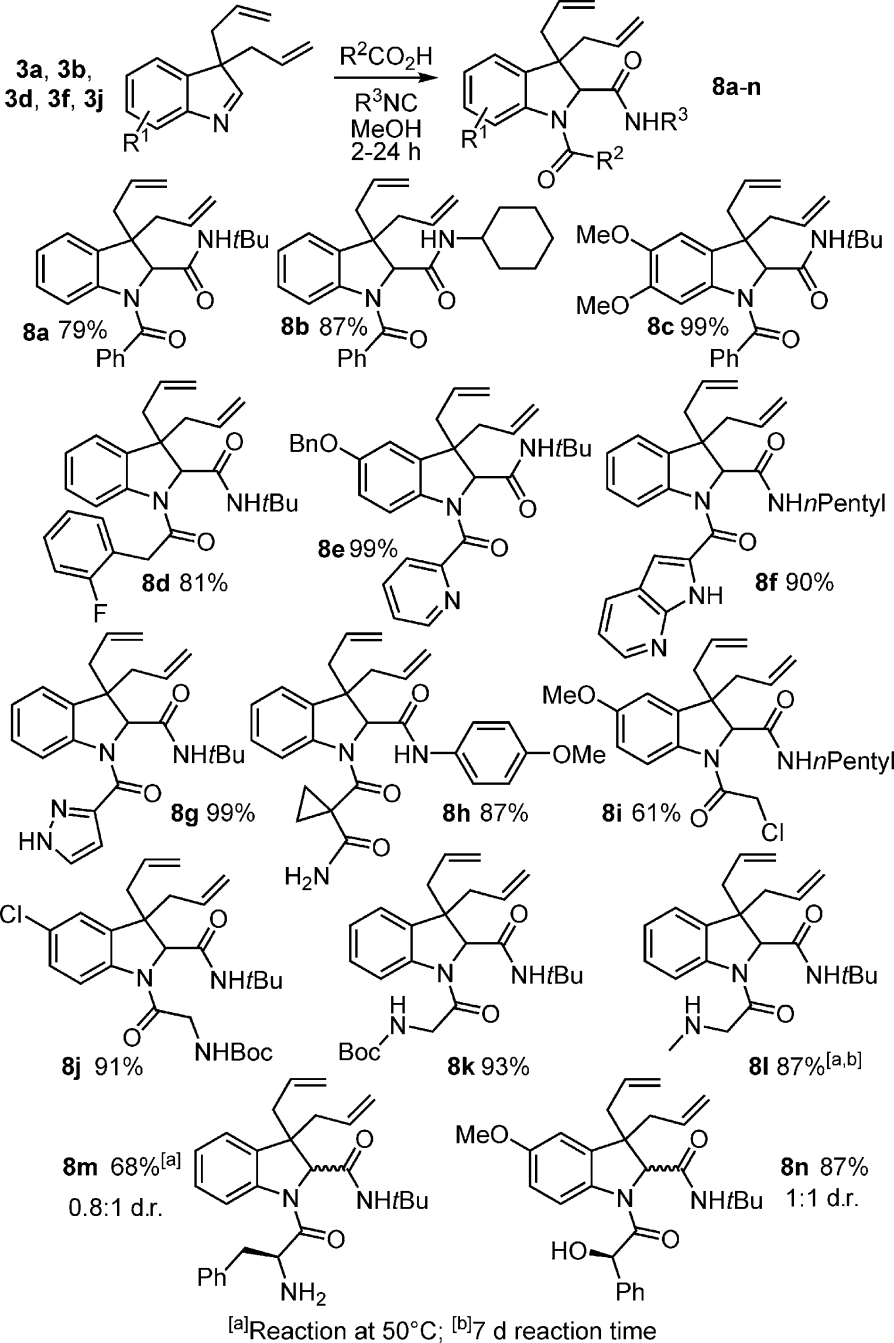
Ugi reactions of the diallylindolinines (d.r.=diastereomeric ratio). [a]Reaction at 50 °C; [b]7 d reaction time.

With these useful diallylindolinine building blocks in hand, we then began to investigate the reactivity of these compounds in further transformations (see Scheme 5). To rapidly introduce diverse functionality onto the indoline core, we explored the use of the diallylindolinines **3** in multicomponent reactions. Multicomponent reactions offer a highly efficient route to construct functionalised molecules based on a central core structure.^28^ The use of imines and their derivatives, in combination with isonitriles and a range of different nucleophiles, has often proved to be a highly effective strategy for constructing bioactive small molecules of potential interest. Indeed, the classical Ugi reaction and closely related processes have proved to be of great value in the synthesis of a vast array of medicinally relevant structures containing an α-aminoamide core.^29^ We envisaged that the highly stable imine unit present in the indolinine core should be an excellent building block for use in Ugi reactions, and surprisingly, the use of such compounds in these multicomponent reactions does not seem to have previously been explored. The diallyl indolinines **3** proved to be excellent substrates for Ugi reactions under standard conditions (Scheme 5). Reaction of selected indolinines with a diverse selection of carboxylic acids and isocyanides in MeOH gave access to a wide range of multicomponent products **8 a**–**n** (Scheme 5). As well as simple aliphatic and aromatic groups (**8 a**–**d**), products containing heterocyclic rings (**8 e**–**g**), primary amides (**8 h**), activated chlorides (**8 i**) and carbamate-protected amines (**8 j**–**k**) could be prepared. Remarkably, even unprotected α-aminoacids (**8 l**–**m**) and α-hydroxyacids (**8 n**) could be used directly in the multicomponent reactions, although, as with many other Ugi reactions using chiral components, only low levels of diastereoselectivity were observed.^30^

**Scheme 6:**
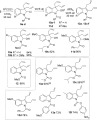
Acylation of diallylindolinines to give 2-hydroxyindolines and 2,3-diallylindoles by subsequent rearrangement. [a]From purified 10 a/10 c; yield for the rearrangement step.

An alternative strategy for functionalisation of the indolinine was devised by reaction of the imine unit with a chloroformate or acid chloride^31^ to give access to 2-chloroindolines, which after hydrolysis during work-up gave the corresponding 2-hydroxyindolines **10 a**–**c** in good yield (Scheme 6). Alternatively, acylation with an acid chloride, followed by quenching with methanol could be used to access a 2-methoxyindoline **11 d**. Acylation of 2-methylindolinine **3 l** with methyl chloroformate resulted in the formation of *N*-acylenamine **12** in good yield. The 2-hydroxyindolines **10 a** and **10 c** readily underwent rearrangement to the corresponding 2,3-diallylindoles **13 a** and **13 c** upon treatment with aluminium trichloride, providing a convenient route to an alternative structural motif. The acylation and rearrangement reactions could be conveniently combined into a single process to provide access to the 2,3-diallylindoles **13 d**–**f** without the need to isolate the intermediate 2-hydroxyindolines.

**Scheme 7:**
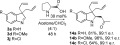
Asymmetric Mannich reactions of diallylindolinines (e.r.=enantiomeric ratio). For compound 14c, the reaction was carried out in Acetone/DMSO.

A strategy for desymmetrising the achiral diallylindolines **3** through asymmetric addition of a nucleophile to the imine group was also explored (Scheme 7). Gratifyingly, we found that L-proline-catalysed Mannich reaction of acetone with three diallylindolinines (**3 a**, **d**, **j**) gave the corresponding 3-aminoketones **14 a**–**c** in good yield and with very high enantioselectivity. During the preparation of this manuscript, similar conditions were reported for the asymmetric Mannich reaction of closely related 3,3-disubstituted indolinines,^32^ so this reaction was not explored in further detail.

**Scheme 8:**
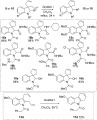
Synthesis of spirocyclic indolines and a carboazole by ring-closing metathesis.

With a range of different substituted indolines in hand, the synthesis of the corresponding spirocyclic indolines via ring closing metathesis was studied (Scheme 8).^33^ A selection of the Ugi products (**8 a**–**c**, **e**, **i**–**k**, **n**) underwent efficient ring-closing metathesis by using Grubbs’ first-generation catalyst in dichloromethane as solvent. The corresponding spirocyclic indolines **15** were isolated in good to excellent yield. The 2-hydroxyindolines **10 a** and **b** could also be smoothly converted into the corresponding spirocyclic indolines **16** in good yield. A dihydrocarbazole **17 d** could also be accessed by a ring-closing metathesis reaction of 2,3-diallylindole **13 d**.

## Conclusion

**Figure 1:**
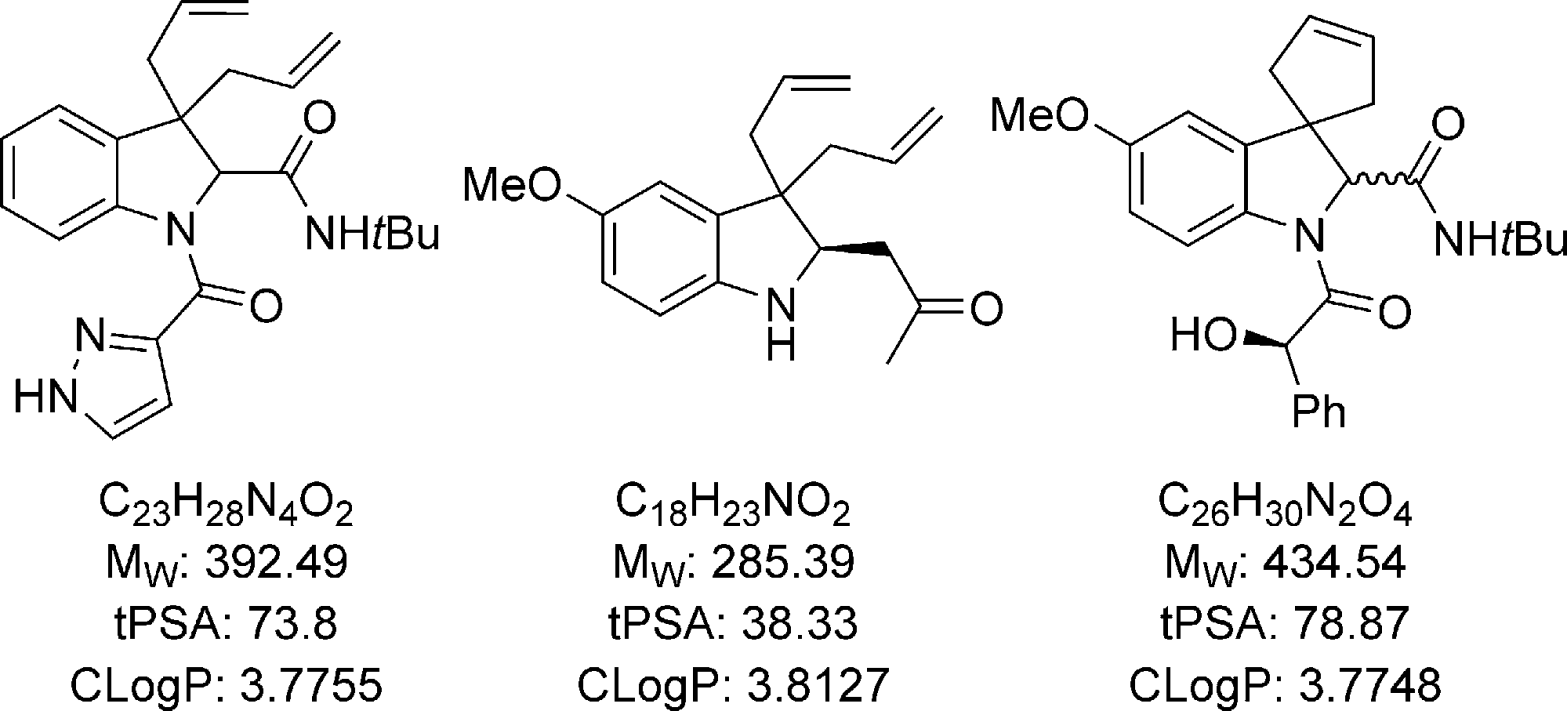
Calculated properties of selected indolines.

We have described the application of the bulk chemical allyl acetate to the palladium-catalysed dearomatising diallylation of indoles. The choice of ligand was found to be critical to controlling the product distribution, and under the optimised conditions, the reaction proceeds efficiently and selectively at room temperature by using 5 mol % of a readily available palladium catalyst. This procedure enables the rapid assembly of a range of substituted indolines from readily available indoles by short synthetic sequences, including a selection of compounds containing the spirocyclic indoline motif. A diverse array of functional groups can be introduced into the scaffold through Ugi multicomponent reactions, and the achiral scaffold can also be desymmetrised by an asymmetric Mannich reaction. Many of these molecules incorporate hydrogen-bond donor and acceptor groups and possess potentially useful drug-like properties (Figure 1). Furthermore, the indolinines generated from the diallylation reaction can also be used to access a variety of 2,3-diallylindoles via acylation and rearrangement.

## Experimental Section

### General methods

All chemicals were purchased from Sigma–Aldrich, Acros, Alfa Aesar or Santa Cruz Biotechnology and used without further purification. Samples of functionalised carboxylic acids for the Ugi reactions were provided by GlaxoSmithKline. 1-Allyl-1*H*-indole (**5 a**), 3-allyl-1*H*-indole (**4 a**), 1,3-diallyl-1*H*-indole (**6 a**) were synthesized according to literature procedures.^27^ Anhydrous THF, dichloromethane and acetonitrile were purchased from Fisher Scientific. All other solvents were used as received. PE refers to petroleum ether. Flash-column chromatography was carried out by using normal-phase silica gel (33–70 μm) supplied by VWR. Thin-layer chromatography was carried out by using Merck TLC Silica gel 60 F_254_ plates and products were visualized by using combinations of UV light (*λ*=254 nm) and potassium permanganate (KMnO_4_) when required.

#### General procedure A: Synthesis of 3,3-diallyl-3H-indolinines (3)

The indole (1 equiv), [Pd(allyl)Cl)]_2_ (2.5 mol %), DPEPhos (5 mol %) and K_2_CO_3_ (3 equiv) were placed in an oven-dried carousel tube. After three vacuum/Ar cycles, acetonitrile (*C*≈0.025 mol L^−1^) and allyl acetate (5 equiv) were successively added. The heterogeneous mixture was stirred at RT for 18–24 h before addition of water. The solution was extracted with Et_2_O and washed with water. The combined organic layers were dried with Na_2_SO_4_, filtered, and volatiles were removed under vacuum. Purification by flash chromatography on SiO_2_ gave the corresponding 3,3-diallyl-3*H*-indole compound **3**.

#### General procedure B: Synthesis of Ugi products (8)

The carboxylic acid (1 equiv) and the isocyanide (1 equiv) were added to a solution of 3,3-diallyl-3*H*-indole (1 equiv) in MeOH (ca. 0.25 mol L^−1^). The reaction mixture was stirred for 2–24 h at RT, before evaporation of the volatiles under vacuum. Pure compounds were obtained by washing the crude residue with PE, or by purification by column chromatography on SiO_2_.

#### General procedure C: Synthesis of 3,3-diallyl-2-hydroxyindolines (10)

The chloroformate or acid chloride (1 equiv) was added to a solution of the 3,3-diallyl-3*H*-indole in CH_2_Cl_2_ (ca. 0.07 mol L^−1^), and the reaction was left to stir for 30 min at RT, before addition of saturated NaHCO_3_. After extraction with CH_2_Cl_2_ (three times), the combined organic layers were washed with water, dried over Na_2_SO_4_ and filtered through cotton wool. Pure compounds were obtained by evaporation of the volatiles under reduced pressure or by purification by column chromatography on SiO_2_.

#### General procedure D: Preparation of 2,3-diallylindoles (13) from 2-hydroxy-3,3-diallylindolines (10)

Aluminium chloride (1.1 equiv) was added to a solution 3,3-diallyl-2-hydroxyindoline (1.0 equiv) in CH_2_Cl_2_ (ca. 1 mol L^−1^) at RT. The mixture was stirred for 30 min before addition of NEt_3_ (2 equiv). After 5 min at RT, water was added, and the product was extracted with CH_2_Cl_2_ (three times). The combined organic layers were dried over Na_2_SO_4_. After evaporation, the crude material was purified by filtration through a small pad of SiO_2_ to give the rearranged product.

### Preparation of 2,3-diallylindoles (13) from 3,3-diallylindolinines (3)

#### General procedure E: Using an acyl chloride

The acyl chloride (1 equiv) was added to a solution of 3,3-diallyl-*3H*-indole (1 equiv) in CH_2_Cl_2_ (ca. 0.3 mol L^−1^) at room temperature. After 30 min, the reaction was quenched by addition of water and the mixture was extracted with CH_2_Cl_2_ (three times). The combined organic layers were dried over Na_2_SO_4_ and filtered. Evaporation of the volatiles give the 3,3-diallyl-2-hydroxyindoline which was directly dissolved in CH_2_Cl_2_ (ca. 0.3 mol L^−1^) and AlCl_3_ (1.1 equiv) was added. After 30 min at room temperature, the reaction was quenched with saturated NaHCO_3_ before extraction with CH_2_Cl_2_ (three times). The combined organic phases were washed with water, dried over Na_2_SO_4_ and filtered. After evaporation of the volatiles under vacuum, the crude residue was purified by a filtration on a small pad of SiO_2_ using PE/Et_2_O (90:10) as eluent.

#### General procedure F: Using a chloroformate

The chloroformate (1 equiv) was added to a solution of 3,3-diallyl-*3H*-indole (1 equiv) in CH_2_Cl_2_ (ca. 0.2 mol L^−1^) at room temperature. After 30 min, the reaction was quenched by addition of saturated NaHCO_3_, and the mixture was extracted with CH_2_Cl_2_ (three times). The combined organic layers were washed with water and dried over Na_2_SO_4_ before filtration. Evaporation of the volatiles gave the 3,3-diallyl-2-hydroxy-indoline derivative which was directly dissolved in CH_2_Cl_2_ (ca. 0.2 mol L^−1^) and AlCl_3_ (1.1 equiv) was added. After 30 min at RT, NEt_3_ (2 equiv) was added. The solution was left to stir for 5 min before addition of a saturated solution of K_2_CO_3_ and extraction with CH_2_Cl_2_ (three times). The combined organic phases were washed with water, dried over Na_2_SO_4_ and filtered. After evaporation of the volatiles under vacuum, the crude residue was purified by filtration through a small pad of SiO_2_ using PE/Et_2_O (100:0 to 90:10) as eluent.

### Asymmetric synthesis of Mannich reaction products (15)

#### General procedure G

L-Proline (30 mol %) was added at 0 °C to a solution of 3,3-diallyl-3*H*-indole (1 equiv) in a mixture of acetone/CHCl_3_ (4.5:1, ca. 0.022 mol L^−1^). The reaction mixture was allowed to warm up slowly to RT and stirred for two days. Evaporation of the solvent followed by purification by column chromatography on SiO_2_ gave the Mannich product.

#### General procedure H

L-Proline (30 mol %) was added at 0 °C to a solution of 3,3-diallyl-3*H*-indole (1 equiv) in a mixture of acetone/DMSO (4:1, ca. 0.016 mol L^−1^). The solution was allowed to warm up slowly to RT and stirred for two days. The reaction mixture was diluted with diethyl ether and washed with saturated NaHCO_3_. The product was extracted with Et_2_O (three times), and the combined organic layers were washed with water, brine and dried with MgSO_4._ After filtration and removal of the solvents under reduce pressure, the crude product was purified by column chromatography on SiO_2_.

#### General procedure I: Synthesis of spirocyclic indolines (**16**)

First-generation Grubbs’ catalyst (15 mol %) was added to a degassed solution of the corresponding Ugi product (1 equiv) in CH_2_Cl_2_ (ca. 0.06 mol L^−1^) at 45 °C. The reaction mixture was heated at reflux for 24 h under argon before evaporation of the solvent under reduced pressure. The crude residue was purified by column chromatography on SiO_2_ to give the desired product.

#### General procedure J: Synthesis of spirocyclic indolines (**17**)

The corresponding substituted 3,3-diallyl-2-hydroxyindoline compound was added to a refluxing solution of first generation Grubbs’ catalyst (15 mol %) in CH_2_Cl_2_ (ca. 0.04 mol L^−1^) under argon. The reaction was heated at reflux for 24 h before evaporation of the volatiles under vacuum. The crude material was purified by column chromatography on SiO_2_ to yield the desired product.

#### General procedure K: Synthesis of dihydro-1H-carbazole (**18**)

Grubbs’ first-generation catalyst (5 mol %) was added to a dried and degassed solution of 2,3-diallyl-1*H*-indole in CH_2_Cl_2_. The mixture was heated overnight at 55 °C before evaporation of the volatiles under reduced pressure. The residue obtained was purified by column chromatography on SiO_2_.
