# Supplementary material for: Rapid Assembly of Functionalised Spirocyclic Indolines by Palladium-Catalysed Dearomatising Diallylation of Indoles with Allyl Acetate
Source: Chemistry. 2014 Aug 29;20(41):13375–81. doi: 10.1002/chem.201403940 (PMC4304291; doi:10.1002/chem.201403940)

CHEMISTRY
A European Journal

Supporting Information

© Copyright Wiley-VCH Verlag GmbH & Co. KGaA, 69451 Weinheim, 2014

Rapid Assembly of Functionalised Spirocyclic Indolines by Palladium-Catalysed Dearomatising Diallylation of Indoles with Allyl Acetate

Persis Dhankher, Laure Benhamou, and Tom D. Sheppard*[a]

chem_201403940_sm_miscellaneous_information.pdf

Table of contents

1. GENERAL METHODS 2

2. SYNTHESIS OF SUBSTITUTED 3,3-DIALLYL-3*H*-INDOLES 3

3. UGI REACTIONS 12

4. SYNTHESIS OF SUBSTITUTED 3,3-DIALLYL-2-HYDROXYINDOLINE 20

5. SYNTHESIS OF 2,3-DIALLYLINDOLES 23

6. (L)-PROLINE CATALYSED ASYMMETRIC MANNICH REACTION 26

7. RING CLOSING METATHESIS REACTIONS OF UGI COMPOUNDS 28

8. RING CLOSING METATHESIS REACTIONS OF SUBSTITUTED 3,3-DIALLYL-2-HYDROXYINDOLINES 32

9. PREPARATION OF DIHYDRO-1*H*-CARBAZOLE BY RING CLOSING METATHESIS 33

10. REFERENCES 34

11. SPECTRA 35

Synthesis of substituted 3,3-diallyl-3H-indole 35
UGI reactions 51
Synthesis of substituted 3,3-diallyl-2-hydroxyindoline 66
Synthesis of 2,3-diallylindoles 71
(L)-Proline catalysed asymmetric Mannich reaction 76
Ring closing metathesis reaction on UGI compounds 79
Ring closing metathesis reaction on substituted 3,3-diallyl-2-hydroxyindoline 85
(L)-Proline catalysed asymmetric Mannich reaction: HPLC data 87
Solvent screening 88
Substrate scope 88
Chiral HPLC chromatograms of Mannich products 89

1. General methods

All chemicals were purchased from Sigma-Aldrich, Acros, Alfa Aesar, Santa Cruz Biotechnology and used without further purification. 1-allyl-1*H*-indole **5a**, 3-allyl-1*H*-indole **4a**, 1,3-diallyl-1*H*-indole **6a** were synthesized according to literature procedures.[1] Anhydrous Tetrahydrofuran, Dichloromethane and Acetonitrile were purchased from Fisher Scientific. All other solvents used as received. PE refers to Petroleum Ether. Flash column chromatography was carried out using normal phase silica gel (33-70 μm) supplied by VWR. Thin layer chromatography was carried out using Merck TLC Silica gel 60 F_{254} plates and products were visualized using combinations of UV light (254 nm) and potassium permanganate ($KMnO_4$) when required. ^{1}H NMR spectra were recorded at 400 or 600 MHz on a Bruker AMX400 and AMX600 spectrometer using the residual protic solvent $CDCl_3$ (δ = 7.26 ppm, s) as the internal standard. Chemical shifts are quoted in ppm to the nearest 0.01 ppm using the following abbreviations: s (singlet), d, (doublet), t (triplet), q, (quartet), qn (quintet), sext (sextet), dd (doublet of doublets), dt (doublet of triplets), m (multiplet) defined as all multi-peak signals where overlap or complex coupling of signals makes definitive descriptions of peaks difficult. The coupling constants *J* are measured in Hz. ^{13}C{^{1}H} NMR spectra were recorded at 100 or 150 MHz on a Bruker AMX400 and AMX600 at 25°C in $CDCl_3$ as described below. All chemical shifts were referenced with $CDCl_3$ solvent (δ = 77.0 ppm, t) as the internal standard. Chemical shifts are reported to the nearest 0.1 ppm. Coupling constants are defined as *J* and quoted in Hz. Mass spectra were performed in the Department of Chemistry, University College London. Infrared spectra were obtained on a Perkin Elmer Spectrum 100 FT-IR Spectrometer operating in ATR mode. Melting points were measured with a Gallenkamp apparatus and are uncorrected. The enantiomeric excess were determined using a Varian ProStar and PrepStar HPLC, with a UV detector system at 254 nm with a CHIRALPAK-AD column (Daicel; Chemical Industries, LTD) 25 × 0.46 cm. Optical rotation $[\alpha]_D^{20}$ values are given in 10^{-1}deg cm^2 g^{-1}, concentration (c) in g/100 mL and were measured on a Perkin-Elmer 343 polarimeter.

2. Synthesis of substituted 3,3-diallyl-3*H*-indoles

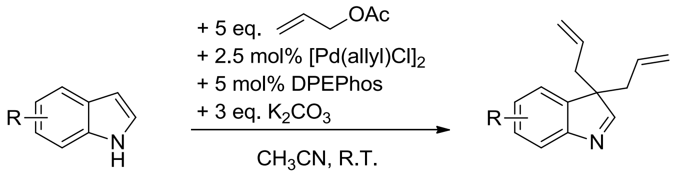

General procedure A: The indole (1 eq.), $[Pd(allyl)Cl)]_2$ (2.5 mol%), DPEPhos (5 mol%), K_2CO_3 (3 eq.) were placed in an oven dried carousel tube. After three vacuum/Ar cycles, acetonitrile (C ≈ 0.025 mol/L) and allyl acetate (5 eq.) were successively added. The heterogenous mixture was stirred at room temperature for 18-14 h before addition of water. The solution was extracted with Et_2O and washed with water. The combined organic layers were dried with Na_2SO_4, filtered and volatiles were removed under vacuum. Purification by flash chromatography on SiO_2 gave the corresponding 3,3-diallyl-3*H*-indole compound.

3,3-Diallyl-3*H*-indole (3a)

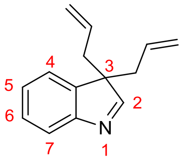

The product was obtained by following the **General procedure A**. The crude residue was purified by column chromatography on SiO_2 using a mixture of PE/EtOAc (100/0 to 90/10) as eluent. The product was obtained as pale orange oil (70 mg, 82%). R_f = 0.64 (PE/EtOAc 1/1); ^1H NMR (600 MHz, $CDCl_3$) δ 8.05 (br s, 1H, H_2), 7.62 (d, 1H, J = 7.1 Hz, H_7), 7.35 (td, 1H, J = 7.1, 1.1 Hz, H_6), 7.31 (brd, 1H, J = 7.1 Hz, H_4), 7.26 (br t, 1H, J = 7.1 Hz, H_5), 5.44 (ddt, 2H, J = 17.1, 10.1, 7.3 Hz, =CH), 5.01 (dd, 2H, J = 17.1, 1.1 Hz, =CH_2), 4.95 (dd, 2H, J = 10.1, 1.1 Hz, =CH_2), 2.57 (dd, 2H, J = 13.9, 7.3 Hz, C$H_{2\ allyl}$), 2.52 (dd, 2H, J = 13.9, 7.3 Hz, C$H_{2\ allyl}$); $^{13}C\{^1H\}$ NMR (150 MHz, $CDCl_3$) δ 177.9 (CH), 155.6 (C_q), 141.4 (C_q), 132.5 (CH), 128.0 (CH), 126.1 (CH), 122.4 (CH), 121.3 (CH), 118.8 (=CH$_2$), 60.9 (C_q), 38.6 (CH$_2$); HRMS (EI) calcd. for $C_{14}H_{15}N$ $[M]^+$ 197.1199, found 197.1194; FT-IR (ATR) υ = 3074 (CH), 2978 (CH), 1600 (N=CH), 1559, 1475 cm^{-1}. Data in accordance with the literature.[2]

3,3-Diallyl-5,6-dimethoxy-3*H*-indole (3b)

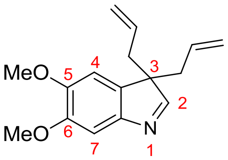

The product was obtained by following the **General procedure A**. The crude residue was purified by column chromatography on SiO_2 using a mixture of PE/EtOAc (100/0 to 80/20) as eluent. The product was obtained as a yellow oil (169 mg, 88%); R_f = 0.44 (PE/EtOAc 4/1); ^1H NMR (600 MHz, $CDCl_3$) δ 7.94 (s, 1H, H_2), 7.20 (s, 1H, H_7), 6.81 (s, 1H, H_4), 5.42 (ddt, 2H, J = 17.0, 10.2, 7.3 Hz, =CH), 5.00 (dq, 2H, J =

17.0, 1.1 Hz, =CH_2), 4.95 (dq, 2H, J = 10.2, 1.1 Hz, =CH_2), 3.92 (s, 3H, OCH_3), 3.90 (s, 3H, OCH_3), 2.53 (ddt, 2H, J = 13.9, 7.3, 1.1 Hz, C$H_{2\ allyl}$), 2.48 (dtd, 2H, J = 13.9, 7.3, 1.1 Hz, C$H_{2\ allyl}$); $^{13}C\{^1H\}$ NMR (150 MHz, $CDCl_3$) δ 176.8 (*C*H), 149. 1 (C_q), 148.8 (C_q), 148.0 (C_q), 133.3 (C_q), 132.6 (*C*H), 118.7 (=*C*H_2), 105.6 (*C*H), 104.9 (*C*H), 61.3 (C_q), 56.5 (*C*H_3), 56.2 (*C*H_3), 38.8 (*C*H_2); HRMS (CI) calcd. for $C_{16}H_{20}NO_2$ $[M+H]^+$ 258.1488, found 258.1484; FT-IR (ATR) υ = 3075 (CH), 2938 (CH), 1600 (N=C), 1465, 1442, 1305, 1214 cm^{-1}.

3, 3-Diallyl-5-methyl-3*H*-indole (3c)

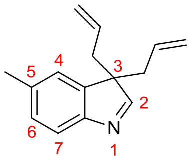

The product was obtained by following the **General procedure A**. The crude residue was purified by column chromatography on SiO_2 using a mixture of PE/EtOAc (100/0 to 90/10) as eluent. The product was obtained as a brown oil (212 mg, 66%); R_f = 0.49 (PE/EtOAc 4/1); ^1H NMR (600 MHz, $CDCl_3$) δ 7.97 (s, 1H, H_2), 7.49 (d, 1H, J = 7.9 Hz, H_7), 7.14 (dd, 1H, J = 7.9, 1.3 Hz, H_6), 7.11 (d, 1H, J = 1.3 Hz, H_4), 5.44 (ddt, 2H, J = 17.1, 10.2, 7.3 Hz, =C*H*), 5.01 (dq, 2H, J = 17.1, 1.4 Hz, =CH_2), 4.96 (dd, 2H, J = 10.2, 1.4 Hz, =CH_2), 2.54 (ddt, 2H, J = 14.0, 7.3, 1.4 Hz, C$H_{2\ allyl}$), 2.49 (ddt, 2H, J = 14.0, 7.3, 1.4 Hz, C$H_{2\ allyl}$), 2.41 (s, 3H, CH_3); $^{13}C\{^1H\}$ NMR (150 MHz, $CDCl_3$) δ 176.9 (*C*H), 153.5 (C_q), 141.7 (C_q), 136.0 (C_q), 132.6 (*C*H), 128.7 (*C*H), 123.1 (*C*H), 120.8 (*C*H), 118.7 (=*C*H_2), 60.9 (C_q), 38.8 (*C*H_2), 21.2 (*C*H_3); HRMS (EI) calcd for $C_{15}H_{17}N$ $[M]^+$ 211.1355, found 211.1349; FT-IR (ATR) υ = 3077 (CH), 2921 (CH), 1640 (N=C), 1556, 1465 cm^{-1}.

3,3-Diallyl-5-methoxy-3*H*-indole (3d)

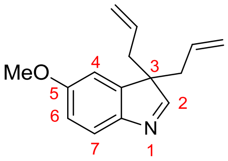

The product was obtained by following the **General procedure A**. The crude residue was purified by column chromatography on SiO_2 using a mixture of PE/EtOAc (100/0 to 80/20) as eluent. The product was obtained as orange oil (233 mg, 76%). R_f = 0.22 (PE/EtOAc 4/1); ^1H NMR (600 MHz, $CDCl_3$) δ 7.91 (s, 1H, H_2), 7.51 (d, 1H, J = 8.3 Hz, H_7), 6.86 (dd, 1H, J = 8.3, 2.2 Hz, H_6), 6.84 (d, 1H, J = 2.2 Hz, H_4), 5.45 (ddt, 2H, J = 17.0, 10.1, 7.2 Hz, =C*H*), 5.02 (dq, 2H, J = 17.0, 1.1 Hz, =CH_2), 4.96 (dd, 2H, J = 10.1, 1.1 Hz, =CH_2), 3.84 (s, 3H, OCH_3), 2.54 (ddt, 2H, J = 13.9, 7.2, 1.1 Hz, C$H_{2\ allyl}$), 2.49 (ddt, 2H, J = 13.9, 7.2, 1.1 Hz, C$H_{2\ allyl}$); $^{13}C\{^1H\}$ NMR (150 MHz, $CDCl_3$) δ 175.8 (*C*H), 158.6 (C_q), 149.4 (C_q), 143.2 (C_q), 132.5 (*C*H), 121.6 (*C*H), 118.8 (=*C*H_2), 112.4 (*C*H), 109.2 (*C*H), 61.0 (C_q), 55.9 (*C*H_3), 38.8 (*C*H_2); HRMS (EI) calcd. for $C_{15}H_{17}NO$ $[M]^+$ 227.1310, found 227.1303; FT-IR (ATR) υ = 3074 (CH), 2921 (CH), 1591 (N=C), 1500, 1437, 1264 cm^{-1}.

3,3-Diallyl-4-methyl-3*H*-indole (3e)

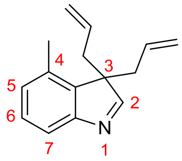

The product was obtained by following the **General procedure A**. The crude residue was purified by column chromatography on SiO_2 using a mixture of PE/EtOAc (100/0 to 70/30) as eluent. The product was obtained as a brown oil (185 mg, 57%); R_f = 0.47 (PE/EtOAc 4/1); ^{1}H NMR (600 MHz, $CDCl_3$) δ 8.04 (s, 1H, H_2), 7.16 (m, 2H, $H_{5,7}$), 7.12 (m, 1H, H_6), 5.45 (ddt, 2H, *J* = 17.2, 10.0, 7.3 Hz, =C*H*), 5.01 (dq, 2H, *J* = 17.2, 1.4 Hz, =CH_2), 4.96 (dq, 2H, *J* = 10.0, 1.4 Hz, =CH_2), 2.59 (s, 3H, CH_3), 2.55 (ddt, 2H, *J* = 14.0, 7.3, 1.4 Hz, C$H_{2\ allyl}$), 2.49 (ddt, 2H, *J* = 14.0, 7.3, 1.4 Hz, C$H_{2\ allyl}$); ^{13}C{^{1}H} NMR (150 MHz, $CDCl_3$) δ 176.7 (*C*H), 154.1 (C_q), 141.4 (C_q), 132.7 (*C*H), 130.9 (C_q), 129.4 (*C*H), 126.1 (*C*H), 119.8 (*C*H), 118.7 (=*C*H_2), 61.1 (C_q), 38.7 (*C*H_2), 17.0 (*C*H_3); HRMS (EI) calcd. for $C_{15}H_{18}N$ $[M]^+$ 211.1355, found 211.1359; FT-IR (ATR) υ = 3076 (CH), 2921 (CH), 1640 (N=C), 1558, 1439 cm $^{-1}$.

3,3-Diallyl-5-benzyloxy-3*H*-indole (3f)

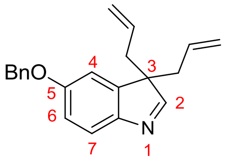

The product was obtained by following the **General procedure A**. The crude residue was purified by column chromatography on SiO_2 using a mixture of PE/EtOAc (100/0 to 70/30) as eluent. The product was obtained as orange oil (196 mg, 76%). R_f = 0.29 (PE/EtOAc 4/1); ^{1}H NMR (600 MHz, $CDCl_3$) δ = 7.92 (s, 1H, H_2), 7.52 (d, 1H, *J* = 8.4 Hz, H_7), 7.45 (br d, 2H, *J* = 7.3 Hz, C$H_{o\text{-Bn}}$), 7.40 (t, 2H, *J* = 7.3 Hz, C$H_{m\text{-Bn}}$), 7.34 (td, 1H, *J* = 7.3, 1.4 Hz, C$H_{p\text{-}Bn}$), 6.95 (dd, 1H, *J* = 8.4, 2.5 Hz, H_6), 6.92 (d, 1H, *J* = 2.5 Hz, H_4), 5.43 (ddt, 2H, *J* = 17.1, 10.5, 7.4 Hz, =C*H*), 5.08 (s, 2H, OCH_2), 5.00 (dq, 2H, *J* = 17.1, 1.4 Hz, =CH_2), 4.96 (br d, 2H, *J* = 10.5 Hz, =CH_2), 2.53 (dd, 2H, *J* = 14.1, 7.4 Hz, C$H_{2\ allyl}$), 2.48 (dd, 2H, 14.1, 7.4 Hz, C$H_{2\ allyl}$); ^{13}C{^{1}H} NMR (150 MHz, $CDCl_3$) δ 175.9 (*C*H), 157.7 (C_q), 149.5 (C_q), 143.2 (C_q), 137.0 (C_q), 132.4 (*C*H), 128.7 (*C*H), 128.2 (*C*H), 127.7 (*C*H), 121.6 (*C*H), 118.9 (=*C*H_2), 113.6 (*C*H), 110.2 (*C*H), 70.7 (*C*H_2), 61.1 (C_q), 38.8 (*C*H_2); HRMS (EI) calcd. for $C_{21}H_{21}NO$ $[M]^+$ 303.1617 found, 303.1612; FT-IR (ATR) υ = 3076 (CH), 2910 (CH), 1590 (N=C), 1580, 1575, 1181, 1022 cm^{-1}.

3,3-Diallyl-3,6,7,8-tetrahydrocyclopenta-3*H*-indole (3g)

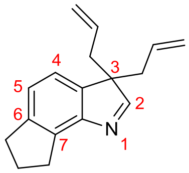

The product was obtained by following the **General procedure A**. The crude residue was purified by column chromatography on SiO_2 using a mixture of PE/EtOAc (100/0 to 80/20) as eluent. The title compound was obtained as a yellow oil (152 mg, 50%); R_f = 0.31 (PE/EtOAc 4/1); ^{1}H NMR (600 MHz, $CDCl_3$) δ 8.03 (br s, 1H, H_2), 7.12 (d, 1H, *J* = 7.5 Hz, H_4), 7.08 (d, 1H, *J* = 7.5 Hz, H_5), 5.47 (ddt, 2H, *J* = 17.1, 10.0, 7.2 Hz, =C*H*), 5.02 (dd, 2H, *J*

= 17.1, 1.2 Hz, H=CH_2), 4.96 (d, 2H, J = 10.0 Hz, =CH_2), 3.19 (t, 2H, J = 7.5 Hz, C_7CH_2), 2.96 (t, 2H, J = 7.5 Hz, C_6CH_2), 2.70 (dd, 2H, J = 13.4, 7.2 Hz, C$H_{2\ allyl}$), 2.49 (dd, 2H, J = 13.8, 7.2 Hz, C$H_{2\ allyl}$), 2.16 (quintet, 2H, J = 7.2 Hz, CH_2); $^{13}C\{^1H\}$ NMR (150 MHz, $CDCl_3$) δ 177.8 (*C*H), 151.4 (C_q), 145.4 (C_q), 139.4 (C_q), 136.6 (C_q), 132.8 (*C*H), 122.7 (*C*H), 120.1 (*C*H), 118.6 (=CH_2), 60.6 (C_q), 38.9 (CH_2), 33.0 (CH_2), 30.0 (CH_2), 25.7 (CH_2); HRMS (CI) $C_{17}H_{20}N$ $[M+H]^+$ 238.1590, found 238.1590; FT-IR (ATR) υ = 3078 (CH), 2951 (CH), 1639 (N=C), 1553, 1438 cm^{-1}.

3,3-Diallyl-4-bromo-3*H*-indole (3h)

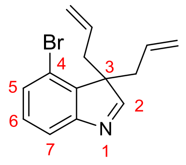

The product was obtained by following the **General procedure A** with slight modifications. The reaction was conducted at 50 °C for 24 hours. The crude residue was purified by column chromatography on SiO_2 using a mixture of PE/EtOAc (100/0 to 90/10) as eluent. The product was obtained as a purple oil (159 mg, 56%) ; R_f = 0.41 (PE/EtOAc 4/1); ^1H NMR (600 MHz, $CDCl_3$) δ 8.02 (s, 1H, H_2), 7.53 (d, 1H, J =7.7 Hz, $H_{5/7}$), 7.36 (d, 1H, J = 7.7 Hz, $H_{5/7}$), 7.21 (t, 1H, J = 7.7 Hz, H_6), 5.24 (ddt, 2H, J = 16.9, 10.2, 7.7 Hz, =CH), 5.02 (d, 2H, J = 16.9 Hz, =CH_2), 4.85 (d, 2H, J = 10.2 Hz, =CH_2), 2.99 (dd, 2H, J = 13.9, 7.7 Hz, C$H_{2\ allyl}$), 2.78 (dd, 2H, J = 13.9, 7.7 Hz, C$H_{2\ allyl}$); $^{13}C\{^1H\}$ NMR (150 MHz, $CDCl_3$) δ 178.7 (*C*H), 157.8 (C_q), 138.9 (C_q), 131.8 (*C*H), 130.2 (*C*H), 129.8 (*C*H), 120.4 (*C*H), 118.6 (=CH_2), 118.0 (C_q), 64.7 (C_q), 36.2 (CH_2); HRMS (EI) calcd. for $C_{14}H_{14}NBr$ $[M]^+$ 275.0304, found 275.0302; FT-IR (ATR) υ = 3077 (CH), 2979 (CH), 1641 (N=C), 1574, 719 cm^{-1}.

3,3-Diallyl-5-bromo-3*H*-indole (3i)

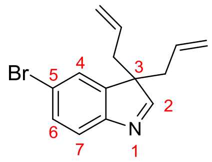

The product was obtained by following the **General procedure A** with slight modifications. The reaction was conducted at 50 °C for 8 hours. The crude residue was purified by column chromatography on SiO_2 using a mixture of PE/EtOAc (100/0 to 80/20) as eluent. The product was obtained as a brown oil (123 mg, 58%); R_f = 0.36 (PE/EtOAc 7/3); ^1H NMR (600 MHz, $CDCl_3$) δ = 8.02 (s, 1H, H_2), 7.48 (br s, 2H, H_6, H_7), 7.44 (s, 1H, H_4), 5.43 (ddt, 2H, J = 17.2, 10.1, 7.3 Hz, =CH), 5.03 (dq, 2H, J = 17.2, 1.5 Hz, =CH_2), 4.99 (dq, 2H, J = 10.1, 1.5 Hz, =CH_2), 2.55 (dd, 2H, J = 14.1, 7.3 Hz, C$H_{2\ allyl}$), 2.50 (dd, 2H, J = 14.1, 7.3 Hz, C$H_{2\ allyl}$); $^{13}C\{^1H\}$ NMR (150 MHz, $CDCl_3$) δ 178.4 (*C*H), 154.7 (C_q), 143.7 (C_q), 131.9 (*C*H), 131.2 (*C*H), 125.8 (*C*H), 122.7 (*C*H), 120.2 (C_q), 119.4 (=CH_2), 61.5 (C_q), 38.4 (CH_2); HRMS (ES) calcd. for $C_{14}H_{13}NBr$ $[M-H]^-$ 274.0231, found 274.0232; FT-IR (ATR) υ = 3076 (CH), 2911 (CH), 1719 (N=C), 1596, 1435 cm^{-1}.

3,3-Diallyl-5-chloro-3*H*-indole (3j)

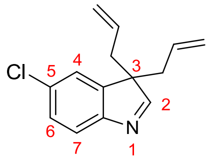

The product was obtained by following the **General procedure A** with slight modifications. The reaction was conducted at 50 °C for 3 hours, then stirred at room temperature for a further 18 hours. The crude residue was purified by column chromatography on SiO_2 using a mixture of PE/EtOAc (100/0 to 90/10) as eluent. The product was obtained as pale yellow oil (126 mg, 41%). R_f = 0.21 (PE/EtOAc 4/1); ^{1}H NMR (600 MHz, $CDCl_3$) δ 8.03 (s, 1H, H_2), 7.53 (d, 1H, J = 8.3 Hz, H_7), 7.32 (dd, 1H, J = 8.3, 2.1 Hz, H_6), 7.28 (d, 1H, J = 2.1 Hz, H_4), 5.43 (ddt, 2H, J = 17.0, 10.1, 7.2 Hz, =C*H*), 5.03 (dq, 2H, J = 17.0, 1.0 Hz, =CH_2), 5.00 (br dd, 2H, J = 10.1, 1.0 Hz, =CH_2), 2.56 (dd, 2H, J = 13.7, 7.2 Hz, C$H_{2\ allyl}$), 2.51 (dd, 2H, J = 13.7, 7.2 Hz, C$H_{2\ allyl}$); ^{13}C{^{1}H} NMR(150 MHz, $CDCl_3$) δ 178.2 (*C*H), 154.2 (C_q), 143.3 (C_q), 132.1 (C_q), 131.9 (*C*H), 128.3 (*C*H), 122.9 (*C*H), 122.2 (*C*H), 119.3 (=*C*H_2), 61.5 (C_q), 38.2 (*C*H_2); HRMS (CI) calcd. for $C_{14}H_{15}ClN$ $[M+H]^+$ 232.0887, found 232.0886; FT-IR (ATR) υ = 3055 (CH), 2954 (CH), 1640 (N=C), 1555, 1447 cm^{-1}.

3,3-Diallyl-5-flouro-3*H*-indole (3k)

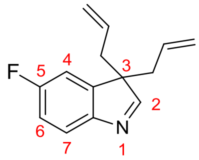

The product was obtained by following the **General procedure A**. The crude residue was purified by column chromatography on SiO_2 using a mixture of PE/EtOAc (100/0 to 80/20) as eluent. The product was obtained as red-brown oil (101 mg, 32%). R_f = 0.4 (PE/EtOAc 7/3); ^{1}H NMR (600 MHz, $CDCl_3$) δ 7.99 (s, 1H, H_2), 7.53 (dd, 1H, J = 8.4, 4.6 Hz, H_7), 7.02 (dd, 1H, J = 8.4, 2.8 Hz, H_6), 6.99 (dd, 1H, J = 8.0, 2.8 Hz, H_4), 5.41 (dtd, 2H, J = 17.1, 10.0, 7.5 Hz, =C*H*), 5.00 (dd, 2H, J = 17.1, 1.3 Hz, =CH_2), 4.96 (br d, 2H, J = 10.0 Hz, =CH_2), 2.53 (dd, 2H, J = 14.0, 7.5 Hz, C$H_{2\ allyl}$), 2.48 (dd, 2H, J = 14.0, 7.5 Hz, C$H_{2\ allyl}$); ^{13}C{^{1}H} NMR (150 MHz, $CDCl_3$) δ 177.7 (*C*H), 161.7 (d, $J_{C\text{-}F}$ = 245.8 Hz, C_q), 151.5 (d, $J_{C\text{-}F}$ = 2.1 Hz, C_q), 143.6 (d, $J_{C\text{-}F}$ = 8.7 Hz, C_q), 132.0 (*C*H), 122.0 (d, $J_{C\text{-}F}$ = 9.1 Hz, *C*H), 119.2 (=*C*H_2), 114.7 (d, $J_{C\text{-}F}$ = 23.7 Hz, *C*H), 110.2 (d, $J_{C\text{-}F}$ = 24.6 Hz, *C*H), 61.6 (C_q), 38.5 (*C*H_2); HRMS (CI) calcd. for ($C_{14}H_{15}NF$) $[M+H]^+$ 216.1183, found 216.1187.; FT-IR (ATR) υ = 3010 (CH), 2928 (CH), 1597 (N=C), 1460, 1165 cm^{-1}.

3,3-Diallyl-2-methyl-3*H*-indole (3l)

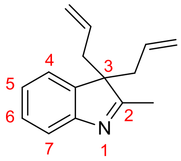

The product was obtained by following the **General procedure A**. The crude residue was purified by column chromatography on SiO_2 using a mixture of PE/EtOAc (100/0 to 80/20) as eluent. The product was obtained as a yellow oil (290 mg, 98%); R_f = 0.60 (PE/EtOAc 7/3) ; ^{1}H NMR (600 MHz, $CDCl_3$) δ 7.51 (d, 1H, J = 7.4 Hz, H_7), 7.31 (t,

1H, J = 7.4 Hz, H_2), 7.26 (d, 1H, J = 7.4 Hz, H_4), 7.19 (t, 1H, J = 7.4 Hz, H_5), 5.10 (ddt, 2H, J = 16.7, 9.9, 7.1 Hz, =CH), 4.95 (br d, 2H, J = 16.7 Hz, =CH_2), 4.84 (br d, 2H, J = 9.9 Hz, =CH_2), 2.68 (dd, 2H, J = 14.0, 7.1 Hz, C$H_{2\ \text{allyl}}$), 2.45 (dd, 2H, J = 14.0, 7.1 Hz, C$H_{2\ \text{allyl}}$), 2.25 (s, 3H, CH_3); ^{13}C{^{1}H} NMR (150 MHz, $CDCl_3$) δ 185.2 (C_q), 155.2 (C_q), 141.2 (C_q), 132.2 (CH), 128.0 (CH), 125.1 (CH), 122.3 (CH), 120.0 (CH), 118.2 (=CH$_2$), 61.9 (C_q), 40.5 (CH$_2$), 16.7 (CH$_3$); HRMS (ES) calcd. for $C_{15}H_{16}N$ [M-H]$^-$ 210.1282, found 210.1273; FT-IR (ATR) υ = 3077 (CH), 2916 (CH), 1640 (N=C), 1457 cm^{-1}. Characterizations were in accordance with literature reports.[2]

3,3-Diallyl-5-methoxy-2-methyl-3*H*-indole (3m)

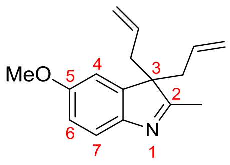

The product was obtained by following the **General procedure A**. The crude residue was purified by column chromatography on SiO_2 using a mixture of PE/EtOAc (100/0 to 90/10) as eluent. The product was obtained as a yellow oil (173 mg, 80%); R_f = 0.33 (PE/EtOAc 7/3); ^{1}H NMR (600 MHz, $CDCl_3$) δ 7.40 (dd, 1H, J = 7.2, 1.8 Hz, H_7), 6.83 (dd, 1H, J = 7.2, 2.5 Hz, H_6), 6.82 (s, 1H, H_4), 5.12 (ddt, 2H, J = 16.9, 10.1, 7.0 Hz, =CH), 4.96 (dq, 2H, J = 16.9, 1.4 Hz, =CH_2), 4.85 (dq, 2H, J = 10.1, 1.2 Hz, =CH_2), 3.83 (s, 3H, OCH_3), 2.64 (ddt, 2H, J = 13.9, 7.0 Hz, C$H_{2\ \text{allyl}}$), 2.44 (ddt, 2H, J = 13.9, 7.0 Hz, C$H_{2\ \text{allyl}}$), 2.21 (s, 3H, CH_3); ^{13}C{^{1}H} NMR (150 MHz, $CDCl_3$) δ 183.0 (C_q), 157.9 (C_q), 148.8 (C_q), 143.0 (C_q), 132.3 (CH), 120.1 (CH), 118.2 (=CH$_2$), 112.2 (CH), 109.2 (CH), 61.9 (C_q), 55.8 (CH$_3$), 40.5 (CH$_2$), 16.6 (CH$_3$); HRMS (ES) calcd. for $C_{16}H_{18}NO$ [M-H]$^-$ 240.1388, found 240.1376; FT-IR (ATR) υ = 2919 (CH), 1581 (N=C), 1471, 1266 cm^{-1}.

3,3-Diallyl-2-phenyl-3*H*-indole (3n)

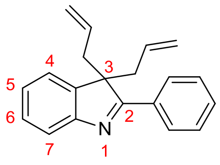

The product was obtained by following the **General procedure A**. The crude residue was purified by column chromatography on SiO_2 using a mixture of PE/EtOAc (100/0 to 80/20) as eluent. The product was obtained as a colourless oil (232 mg, 74%) ; R_f = 0.63 (PE/EtOAc 4/1); ^{1}H NMR (600 MHz, $CDCl_3$) δ 8.11 (m, 2H, C$H_{o\text{-Ph}}$), 7.66 (d, 1H, J = 7.5 Hz, H_7), 7.48 (m, 3H, C$H_{m,p\text{-Ph}}$), 7.37 (td, 1H, J = 7.5, 1.0 Hz, H_6), 7.34 (d, 1H, J = 7.5 Hz, H_4), 7.28 (d, 1H, J = 7.5 Hz, H_5), 5.11 (ddt, 2H, 17.2, 9.8, 7.2 Hz, =CH), 4.76 (br d, 2H, J = 17.2 Hz, =CH_2), 4.72 (br d, 2H, J = 9.8 Hz, =CH_2), 2.92 (dd, 2H, J = 14.1, 7.2 Hz, C$H_{2\ \text{allyl}}$), 2.88 (dd, 2H, J = 14.1, 7.2 Hz, C$H_{2\ \text{allyl}}$); ^{13}C{^{1}H} NMR (150 MHz, $CDCl_3$) δ 180.4 (C_q), 154.6 (C_q), 143.1 (C_q), 134.1 (C_q), 132.0 (=CH$_2$), 130.7 (CH), 128.7 (CH), 128.2 (CH), 128.1 (CH), 125.8 (CH), 121.8 (CH), 120.9 (CH), 118.3 (CH), 62.5 (C_q), 42.0 (CH$_2$); HRMS (CI) calcd. for $C_{20}H_{20}N$ [M+H]$^+$ 274.1590, found 274.1571; FT-IR (ATR) υ = 3075 (CH), 2924 (CH), 1640 (N=C), 1522, 1443 cm^{-1}.

2-(((*tert*-Butyldimethylsilyl)oxy)methyl)-1*H*-indole [3]

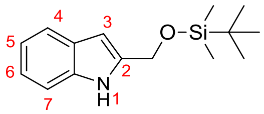

To an oven dry flask purged with vacuum/Argon cycles, were added indole-2-methanol (250 mg, 1.7 mmol), NEt_3 (0.71 ml, 5.1 mmol, 3 eq) and TBDMSCl (384 mg, 2.5 mmol, 1.5 eq) in CH_2Cl_2 (2 ml). The reaction mixture was stirred for 4 hrs at R.T. before addition of a $NaHCO_{3sat}$ (2 × 20 ml). The product was extracted with CH_2Cl_2 and the combined organic layers were washed with water (15 ml), brine (15 ml) and concentrated under vacuum. The residue was purified by flash chromatography on SiO_2 using (PE/EtOAc 100/0 to 90/10) as eluent to yield the title compound as an orange brown oil (254 mg, 57%). R_f = 0.63 (PE/EtOAc 4/1); ^{1}H NMR (600 MHz, $CDCl_3$) δ 8.29 (s, 1H, N*H*), 7.56 (d, 1H, *J* = 7.8 Hz, H_7), 7.37 (d, 1H, *J* = 7.8 Hz, H_4), 7.16 (td, 1H, *J* = 7.8, 1.1 Hz, H_5), 7.08 (td, 1H, *J* = 7.8, 1.1 Hz, H_6), 6.31 (s, 1H, H_3), 4.87 (s, 2H, CH_1), 0.93 (s, 9H, C$H_{3\ tBu}$), 0.12 (s, 6H, CH_3); ^{13}C{^{1}H} NMR (150 MHz, $CDCl_3$) δ 138.3 (C_q), 136.0 (C_q), 128.5 (C_q), 121.7 (*C*H), 120.5 (*C*H), 119.8 (*C*H), 110.9 (*C*H), 98.9 (*C*H), 59.4 (CH_2), 26.0 ($CH_{3\ tBu}$), 18.5 (C_q), 5.17 (CH_3); FT-IR (ATR) υ = 3015 (CH), 2928 (CH), 2857 (CH), 1457, 1255, 1074 cm^{-1}.

3,3-Diallyl-2-(((*tert*-butyldimethylsilyl)oxy)methyl)-3*H*-indole (3o)

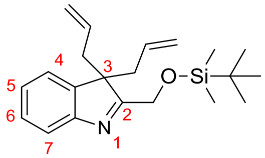

The product was obtained by following the **General procedure A**. The crude residue was purified by column chromatography on SiO_2 using a mixture of PE/EtOAc (100/0 to 90/10) as eluent. The title compound was obtained as orange oil (172 mg, 66%). R_f = 0.80 (PE/EtOAc 4/1); ^{1}H (600 MHz, $CDCl_3$) δ 7.52 (d, 1H, *J* = 7.5 Hz, H_7), 7.25 (td, 1H, *J* = 7.5, 1.3 Hz, H_6), 7.25 (d, 1H, *J* = 7.5 Hz, H_4), 7.21 (td, 1H, *J* = 7.5, 1.3 Hz, H_5), 5.18 (ddt, 2H, *J* = 17.0, 9.9, 7.2 Hz, =C*H*), 4.91 (dq, 2H, *J* = 17.0, 1.9 Hz, =CH_2), 4.80 (dq, 2H, *J* = 9.9, 1.9 Hz, =CH_2), 4.77 (s, 2H, OCH_2), 2.84 (ddt, 2H, *J* = 13.5, 7.2, 1.1 Hz, C$H_{2\ allyl}$), 2.71 (ddt, 2H, *J* = 13.6, 7.2, 1.1 Hz, C$H_{2\ allyl}$), 0.97 (s, 9H, C$H_{3\ tBu}$), 0.17 (s, 6H, CH_3); ^{13}C{^{1}H} (150 MHz, $CDCl_3$) δ 185.5 (C_q), 154.5 (C_q), 142.3 (C_q), 132.9 (*C*H), 127.9 (*C*H), 125.6 (*C*H), 122.1 (*C*H), 120.5 (*C*H), 117.9 (=CH_2), 64.3 (CH_2), 62.7 (C_q), 40.4 (CH_2), 26.4 ($CH_{3\ tBu}$), 18.6 (C_q), 5.6 (SiCH_3); HRMS (CI) calcd. for $C_{15}H_{16}NO$ $[M-SiC_7H_{16}]^-$ 226.1237, found 226.1226; FT-IR (ATR) υ = 3015 (CH), 2929 (CH), 1693 (N=C), 1501, 1490, 1254, 1090 cm^{-1}.

1-Allyl-5-nitro-1*H*-indole (7a)

The product was obtained by following the **General procedure A** with slight modifications. The reaction mixture was heated at 50 °C overnight. The crude residue was purified by column chromatography on SiO_2 using a mixture of PE/EtOAc (100/0 to 80/20) as eluent. The title compound was obtained as a

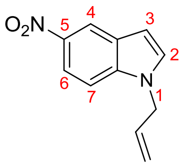

yellow oil (177 mg, 79%); R_f = 0.88 (PE/EtOAc 7/3); ^{1}H NMR (600 MHz, $CDCl_3$) δ 8.60 (d, 1H, *J* = 2.4 Hz, H_4), 8.11 (dd, 1H, *J* = 9.2, 2.4 Hz, H_6), 7.34 (d, 1H, *J* = 9.2 Hz, H_7), 7.25 (d, 1H, *J* = 3.3 Hz, H_2), 6.70 (dd, 1H, *J* = 3.3, 0.7 Hz, H_3), 6.00 (ddt, 1H, *J* = 17.1, 10.1, 7.8 Hz, =C*H*), 5.26 (dq, 1H, *J* = 10.1, 1.3 Hz, =CH*H*), 5.08 (dq, 1H, *J* = 17.1, 1.3 Hz, =CH*H*), 4.79 (dt, 2H, *J* = 5.3, 1.3 Hz, CH_2); ^{13}C{^{1}H} NMR (150 MHz, $CDCl_3$) δ 141.8(C_q), 139.0 (C_q), 132.4 (*C*H), 131.2 (*C*H), 127.9 (C_q), 118.4 (*C*H), 118.2 (=CH_2), 117.4 (*C*H), 109.6 (*C*H), 104.3 (*C*H), 49.4 (CH_2); HRMS (ES^-) calcd. for $C_{11}H_{11}N_2O_2$ $[M\text{-}H]^-$ 201.0664, found 201.0666; FT-IR (ATR) υ = 2923 (CH), 2924 (CH), 1509, 1478, 1340 (O=N=O), 1069 cm^{-1}. Data were in accordance with the literature.[4]

1-Allyl-2-methyl-5-nitro-1*H*-indole (7b)

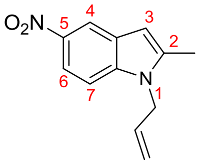

The product was obtained by following the **General procedure A** with slight modifications. The reaction mixture was heated at 50 °C overnight. The crude residue was purified by column chromatography on SiO_2 using a mixture of PE/EtOAc (100/0 to 80/20) as eluent. The title compound was obtained as a orange oil (234 mg, 86%); R_f = 0.56 (PE/EtOAc 4/1); ^{1}H NMR (600 MHz, $CDCl_3$) δ 8.48 (d, 1H, *J* = 1.9 Hz, H_4), 8.04 (dd, 1H, *J* = 8.9, 1.9 Hz, H_6), 7.22 (d, 1H, *J* = 8.9 Hz, H_7), 6.45 (s, 1H, H_3), 5.94 (dtd, 1H, *J* = 17.2, 9.7, 5.5 Hz, =C*H*), 5.16 (d, 1H, *J* = 9.7 Hz, =CH*H*), 4.77 (d, 2H, *J* = 17.2 Hz, =CH_2), 4.23 (dd, 2H, *J* = 5.5, 2.8 Hz, NCH_2), 2.42 (s, 3H, CH_3); ^{13}C{^{1}H} NMR (150 MHz, $CDCl_3$) δ 141.7 (C_q), 140.4 (C_q), 139.9 (C_q), 132.4 (*C*H), 127.4 (C_q), 116.9 (=CH_2), 108.9 (*C*H), 102.7 (*C*H), 45.7 (CH_2), 12.8 (CH_3); HRMS (ES) calcd. for $C_{12}H_{12}N_2O_2$ $[M]^+$ 216.2400, found 216.2430; FT-IR (ATR) υ = 2923 (CH), 1513 (N=C), 1476, 1350 (O=N=O), 1071 cm^{-1}.

3. UGI reactions

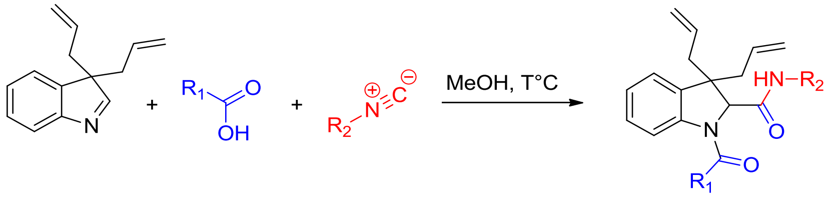

General procedure B: The carboxylic acid (1eq.) and the isocyanide (1eq.) were added to a solution of 3,3-diallyl-3*H*-indole (1eq.) in MeOH (C ≈ 0.25 mol/L). The reaction mixture was left to stir between 2 - 24 hours at room temperature before evaporation of the volatiles under vacuum. Pure compounds were obtained by washing the crude residue with PE, or by purification by column chromatography on SiO_2.

3,3-Diallyl-1-benzoyl-*N*-(*tert*-butyl)indoline-2-carboxamide (8a)

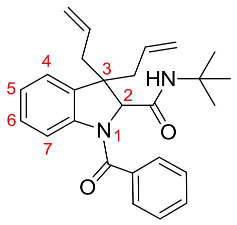

The product was obtained by following the **General procedure B**. After evaporation of the volatiles The desired product was obtained as a pale yellow oil (81 mg, 79%); m.p. 137-138 °C; R_f = 0.37 (PE/EtOAc 4/1); ^{1}H NMR (600 MHz, $CDCl_3$, R.T.) δ 7.53 (d, 2H, *J* = 7.4 Hz, C$H_{o\text{-Bz}}$), 7.50 (t, 1H, *J* = 7.4 Hz, C$H_{p\text{-Bz}}$), 7.43 (t, 2H, *J* = 7.4 Hz, C$H_{m\text{-Bz}}$), 7.17 (d, 1H, *J* = 7.5 Hz, H_4), 7.06 (br s, 1H, H_6), 7.02 (t, 1H, *J* = 7.5 Hz, H_5), 6.00 (ddt, 1H, *J* = 16.5, 9.2, 7.2 Hz, =C*H*), 5.58 (ddt, 1H, *J* = 16.5, 9.2, 7.2 Hz, =C*H*), 5.49 (br s, 1H, N*H*), 5.15-5.05 (m, 4H, =CH_2), 4.48 (s, 1H, H_2), 2.62 (dd, 1H, *J* = 14.7, 7.2 Hz, CH*H* allyl), 2.55 (dd, 1H, *J* = 14.7, 7.2 Hz, CH*H* allyl), 2.42 (m, 2H, C$H_{2\ \text{allyl}}$), 1.30 (s, 9H, C$H_{3\ t\text{Bu}}$); H_7 peak present at baseline 7.06 ppm; ^{13}C{^{1}H} NMR (150 MHz, $CDCl_3$, R.T.) δ 169.5 (C_q), 167.6 (C_q), 142.0 (C_q), 137.1 (C_q), 136.1 (C_q), 134.0 (*C*H), 133.6 (*C*H), 131.1 (*C*H), 128.9 (*C*H), 128.1 (*C*H), 127.6 (*C*H), 124.2 (*C*H), 124.0 (*C*H), 119.4 (=CH_2), 119.2 (=CH_2), 73.1 (*C*H), 51.9 (C_q), 49.8 (C_q), 44.7 (*C*H), 38.9 (CH_2), 28.6 ($CH_{3\ t\text{Bu}}$); C_7H not observed by ^{13}C{^{1}H} NMR; HRMS (CI) calcd. for $C_{26}H_{31}N_2O_2$ $[M+H]^+$ 403.2380, found 403.2353; FT-IR υ = 3347 (N-H), 2970 (CH), 2925 (CH), 1685 (C=O), 1624 (C=O), 1501, 1393 cm^{-1}.

3,3-Diallyl-1-benzoyl-*N*-cyclohexylindoline-2-carboxamide (8b)

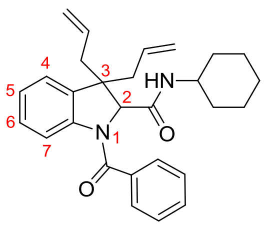

The product was obtained by following the **General procedure B**. After evaporation of the volatiles under vacuum, the residue was purified by column chromatography on SiO_2 using (PE/EtOAc 100/0 to 70/30) as eluent. The desired product was obtained as a pale yellow oil (174 mg, 87%); R_f = 0.30 (PE/EtOAc 4/1); ^{1}H NMR (400 MHz, $CDCl_3$, 55°C) δ 7.54-7.47 (m, 3H, C$H_{o\text{-Bz}}$, C$H_{p\text{-Bz}}$),

7.42 (t, 2H, J = 7.9 Hz, C$H_{m\text{-Bz}}$), 7.20 (d, 1H, J = 7.5 Hz, H_4), 7.15 (br s, 1H, H_7), 7.11 (t, 1H, J = 7.8 Hz, H_6), 7.05 (td, 1H, J = 7.1, 1.4 Hz, H_5), 6.00 (ddt, 1H, J = 17.0, 9.9, 7.5 Hz, =C*H*), 5.62 (ddt, 2H, J = 17.0, 9.9, 7.5 Hz, =C*H*, N*H*), 5.14-5.03 (m, 4H, =CH_2), 4.60 (s, 1H, H_2), 3.75 (m, 1H, (NH)CH_{Cy}), 2.63 (dd, 2H, J = 14.3, 7.5 Hz, C$H_{2\text{ allyl}}$), 2.47 (dt, 2H, J = 14.3, 7.5 Hz, C$H_{2\text{ allyl}}$), 1.95 (dd, 1H, J = 11.7, 2.8 Hz, CHH_{Cy}), 1.81-1.73 (m, 1H, CHH_{Cy}), 1.69-1.64 (m, 1H, CHH_{Cy}), 1.62-1.54 (m, 2H, C$H_{2\text{ Cy}}$), 1.44-1.28 (m, 2H, C$H_{2\text{ Cy}}$), 1.23-1.12 (qd, 2H, J = 12.0, 4.0 Hz, C$H_{2\text{ Cy}}$), 1.07 (qd, 1H, J = 11.3, 3.4 Hz, CHH_{Cy}); ^{13}C{^{1}H} NMR (150 MHz, $CDCl_3$, R.T.) δ 169.5 (C_q), 167.6 (C_q), 141.8 (C_q), 137.2 (C_q), 136.0 (C_q), 133.9 (*C*H), 133.5 (*C*H), 131.0 (*C*H), 128.8 (*C*H), 128.0 (*C*H), 127.4 (*C*H), 124.3 (*C*H), 119.5 (=CH_2), 119.0 (=CH_2), 73.0 (*C*H), 50.0 (C_q), 48.2 (*C*H), 45.1 (CH_2), 38.8 (CH_2), 32.9 (CH_2), 32.8 (CH_2), 25.5 (CH_2), 24.8 (CH_2), 24.7 (CH_2); C_7H was not observed by ^{13}C{^{1}H} NMR; HRMS (CI) calcd. for $C_{28}H_{32}N_2O_2$ $[M]^+$ 428.2463, found 428.2460; FT-IR (ATR) υ = 3314 (N-H), 2930 (CH), 2855 (CH), 1638 (C=O), 1594, 1559, 1470 cm^{-1}.

3,3-Diallyl-1-benzoyl-*N*-(*tert*-butyl)-5,6-dimethoxyindoline-2-carboxamide (8c)

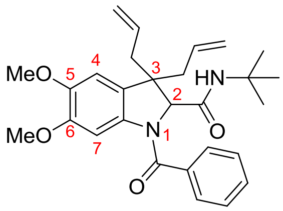

The product was obtained by following the **General procedure B**. Evaporation of the volatiles gave the title compound as yellow brown oil (249 mg, 99%). R_f = 0.15 (PE/EtOAC 4/1); ^{1}H NMR (400 MHz, $CDCl_3$, 55°C) δ 7.53 (d, 2H, J = 8.2 Hz, C$H_{o\text{-Bz}}$), 7.46 (t, 3H, J = 8.2 Hz, C$H_{m,p\text{-Bz}}$), 6.88 (br s, 1H, H_7), 6.76 (s, 1H, H_4), 6.04 (ddt, 1H, J = 17.4, 10.6, 7.1 Hz, =C*H*), 5.61 (ddt, 1H, J = 17.4, 10.6, 7.1 Hz, =C*H*), 5.18-5.05 (m, 4H, =CH_2), 5.52 (br s, 1H, N*H*), 4.52 (s, 1H, H_2), 3.84 (s, 3H, OCH_3), 3.67 (s, 3H, OCH_3), 2.64 (dd, 1H, J = 14.6, 7.1 Hz, CHH_{allyl}), 2.59 (dd, 1H, J = 14.6, 7.1 Hz, CHH_{allyl}), 2.46 (dd, 1H, J = 13.9, 7.1 Hz, CHH_{allyl}), 2.40 (dd, 1H, J = 13.7, 7.1 Hz, CHH_{allyl}), 1.30 (s, 9H, C$H_{3\text{ }tBu}$); ^{13}C{^{1}H} NMR (150 MHz, $CDCl_3$, R.T.) δ 168.9 (C_q), 167.8 (C_q), 148.4 (C_q), 145.9 (C_q), 136.2 (C_q), 135.3 (C_q), 134.3 (*C*H), 133.6 (*C*H), 130.8 (*C*H), 128.8 (*C*H), 128.4 (C_q), 127.4 (*C*H), 119.4 (=CH_2), 119.1 (=CH_2), 107.4 (*C*H), 101.0 (C_q), 73.9 (*C*H), 56.6 (CH_3), 55.8 (CH_3), 51.8 (C_q), 45.2 (CH_2), 39.1 (CH_2), 28.7 ($CH_{3\text{ }tBu}$), C_7H and one signal of OCH_3 were not observed by ^{13}C{^{1}H} NMR; HRMS (EI) calcd. for $C_{28}H_{34}N_2O_4$ $[M]^+$ 462.2519, found 462.2524; FT-IR (ATR) υ = 3342 (N-H), 2966 (CH), 2936 (CH), 1682 (C=O), 1623 (C=O), 1501, 1448, 1214 cm^{-1}.

3,3-Diallyl-*N*-(*tert*-butyl)-1-(2-(2-fluoro)phenyl)acetyl)indoline-2-carboxamide (8d)

The product was obtained according to the **General procedure B**. After 4 hours at room temperature the crude residue was purified by washing with cold Petroleum ether and dried under vacuum to yield the product as a yellow solid (89 mg, 81%); mp = 128 - 129 °C; R_f = 0.59 (PE/EtOAc 70/30); ^{1}H NMR (400 MHz, $CDCl_3$, R.T.) δ 8.18 (br s, 1H, H_7), 7.40 – 7.19 (m, 3H, CH_{Ph}, H_4, H_6), 7.19 – 6.92 (m, 4H, CH_{Ph},

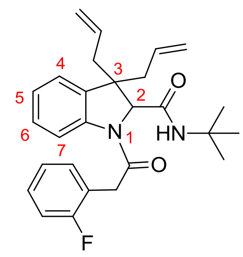

H_5), 6.08 (dtd, J = 14.9, 8.6, 6.0 Hz, 1H, =CH), 5.51 – 5.30 (m, 2H, NH, =CH), 5.25 – 5.17 (m, 2H, =CH_2), 4.91 (m, 2H, =CH_2), 4.51 (s, 1H, H_2), 3.76 (s, 2H, COCH_2), 2.65 (qd, J = 14.6, 7.0 Hz, 2H, C$H_{2\ allyl}$), 2.31 (qd, J = 13.7, 7.4 Hz, 2H, C$H_{2\ allyl}$), 1.29 (s, 9H, C$H_{3\ tBu}$); ^{13}C{^{1}H} NMR (151 MHz, CDCl$_3$, R.T.) δ 169.1 (NHC=O), 168.4 (NC=O), 163.0 (d, J_{CF} = 246.3 Hz, C-F), 141.5 (C_q), 136.0 (C_q), 133.8 (=CH), 132.8 (=CH), 130.3 (d, J_{CF} = 8.4 Hz, CH$_{Ph}$), 128.7 (C_5H), 125.2 (d, J_{CF} = 2.3 Hz, CH$_{Ph}$), 124.9 (C_6H), 124.2 (C_4H), 119.4 (=CH$_2$), 119.3 (=CH$_2$), 116.5 (d, J_{CF} = 21 Hz, CH$_{Ph}$), 114.4 (d, J_{CF} = 21 Hz, CH$_{Ph}$), 72.4 (C_2H), 52.0 (C_3), 46.0 (CH$_{2\ allyl}$), 42.4 (COCH$_2$), 38.6 (CH$_{2\ allyl}$), 28.6 (CH$_{3\ tBu}$), C_7H was not observed by ^{13}C{^{1}H} NMR; HRMS (EI) calcd for $C_{27}H_{31}FN_2O_2$ [M]$^+$ 434.2369 found 434.2358; FT-IR (ATR) υ = 3344 (NH), 3071 (CH), 2967 (CH), 2924 (CH), 1672 (C=O), 1639 (C=O), 1626 (C=O), 1591, 1543, 1482, 1453, 1425, 1392, 1302, 1243, 1032, 995, 794, 770 cm^{-1}.

3,3-Diallyl-5-(benzyloxy)-*N*-(*tert*-butyl)-1-picolinoylindoline-2-carboxamide (8e)

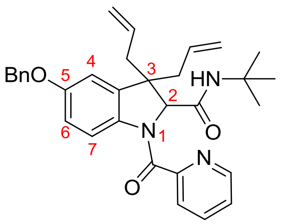

The product was obtained by following the **General procedure B**. Evaporation of the volatiles gave the title compound as a yellow brown oil (286 mg, 99%); R$_f$ = 0.15 (PE/EtOAc 4/1); ^{1}H NMR (400 MHz, CDCl$_{3,}$ 55°C) δ 8.56 (d, 1H, J = 4.7 Hz, CH $_{o\text{-Py}}$), 8.12-8.00 (br s, 1H, H_7), 7.88 (d, 1H, J = 7.6 Hz, CC$H_{m\text{-Py}}$), 7.81 (td, 1H, J = 7.6, 1.4 Hz, C$H_{p\text{-Py}}$), 7.44 (d, 2H, J = 7.4 Hz, C$H_{o\text{-Bn}}$), 7.40 (t, 2H, J = 7.4 Hz, C$H_{m\text{-Bn}}$), 7.36 (ddd, 1H, J = 7.6, 4.7, 1.4 Hz, (CH)C$H_{m\text{-Py}}$), 7.33 (tt, 1H, J = 7.4, 1.3 Hz, C$H_{p\text{-Bn}}$), 6.89 (br s, 1H, H_6), 6.86 (br s, 1H, H_4), 6.03 (ddt, 1H, J = 16.8, 10.7, 7.4 Hz, =CH), 5.51 (ddt, 2H, J = 16.8, 10.7, 7.4 Hz, =CH, NH), 5.10 (dd, 2H, J = 16.8, 10.7 Hz, =CH_2), 5.06 (s, 2H, OCH_2), 5.00 (d, 2H, J = 10.7 Hz, =CH_2), 4.92 (s, 1H, H_2), 2.58 (m, 2H, C$H_{2\ allyl}$), 2.34 (br d, 2H, ,J = 7.4 Hz, C$H_{2\ allyl}$), 1.21 (s, 9H, C$H_{3\ tBu}$); ^{13}C{^{1}H} NMR (150 MHz, CDCl$_3$, R.T.) δ 168.6 (C_q), 166.3 (C_q), 156.3 (C_q), 153.5 (C_q), 147.9 (CH), 138.9 (C_q), 137.4 (CH), 137.0 (C_q), 136.1 (C_q), 134.4 (CH), 133.5 (CH), 128.7 (CH), 128.2 (CH), 127. 2 (CH), 125.2 (CH), 124.4 (CH), 119.1 (=CH$_2$), 118.8 (=CH$_2$), 118.5 (C_q), 113.7 (CH), 111.6 (CH), 74.1 (CH), 70.7 (CH$_2$), 51.4 (C_q), 50.9 (C_q), 45.5 (CH$_2$), 39.0 (CH$_2$), 28.6 (CH$_{3\ tBu}$); C_7H was not observed by ^{13}C{^{1}H} NMR; HRMS (CI) calcd. for $C_{32}H_{36}N_3O_3$ [M+H]$^+$ 510.2757, found 510.2729; FT-IR (ATR) υ = 3342 (N-H), 2965 (CH), 2933 (CH), 1683 (C=O), 1630 (C=O), 1501, 1446, 1214 cm^{-1}.

3,3-Diallyl-*n*pentyl-1-(1*H*-indole-3-carbonyl)indoline-2-carboxamide (8f)

The product was obtained according to the **General procedure B**. After 24 hours at room temperature, purification of the crude residue by column chromatography on SiO$_2$ (PE/EtOAc 90/10 to 0/100) afford the product as a pale yellow solid (103 mg, 90%), mp = 138 - 139°C; R$_f$ = 0.47 (EtOAc); ^{1}H NMR (400

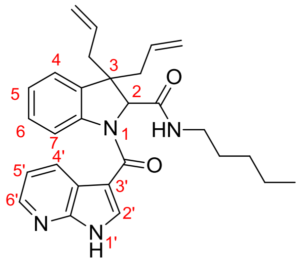

MHz, $CDCl_3$, 55°C) δ 8.54 (dd, *J* = 7.9, 1.1 Hz, 1H, $H_{4'}$), 8.44 (br s, 1H, $H_{6'}$), 7.89 (m, 2H, $H_{2'}$, H_7), 7.31 – 7.21 (m, 3H, $H_{5'}$, H_4, H_6), 7.12 (dd, *J* = 11.2, 3.7 Hz, 1H, H_5), 6.01 (m, 2H, =C*H*, N*H*), 5.58 (ddt, *J* = 17.4, 10.1, 7.3 Hz, 1H, =C*H*), 5.17 (t, *J* = 13.0 Hz, 2H, =CH_2), 5.03 – 4.89 (m, 2H, =CH_2, H_2), 3.25 (td, *J* = 13.3, 7.0 Hz, 1H, NC*H*H), 3.14 (td, *J* = 12.9, 7.0 Hz, 1H, NCH*H*), 2.69 (qd, *J* = 14.8, 7.1 Hz, 2H, C$H_{2\ allyl}$), 2.46 (d, *J* = 7.2 Hz, 2H, C$H_{2\ allyl}$), 1.43 – 1.33 (m, 2H, CH_2), 1.27 – 1.06 (m, 4H, CH_2), 0.79 (t, *J* = 7.1 Hz, 3H, CH_3); $^{13}C\{^1H\}$ NMR (101 MHz, $CDCl_3$, R.T.)169.9 (NH*C*=O), 164.5 (N*C*=O), 148.0 (N*C*N), 144.0 ($C_{6'}$H), 142.8 (C_q), 136.9 (C_q), 134.0 (=*C*H), 133.2 (=*C*H), 131.6 ($C_{4'}$H), 128.4 (C_6H), 128.1 ($C_{2'}$H), 124.5 (C_5H), 124.4 (C_4H), 119.3 (=*C*H_2), 118.7 (=*C*H_2), 118.0 ($C_{5'}$H), 117.6 (C_7H), 110.6 ($C_{3'}$H), 73.5 (C_2H), 51.2 (C_3), 45.9 (*C*$H_{2\ allyl}$), 39.6 (N*C*H_2), 38.8 (*C*$H_{2\ allyl}$), 29.02 (*C*H_2), 29.0 (*C*H_2), 22.3 (*C*H_2), 13.8 (*C*H_3); HRMS (CI) calcd for $C_{28}H_{33}N_4O_2$ $[M+H]^+$ 457.2603 found 457.2597; FT-IR (ATR) υ = 3197 (NH), 3076 (CH), 2955 (CH), 2927 (CH), 2859 (CH), 1653 (C=O), 1616 (C=O), 1586, 1520, 1477, 1413, 1377, 1290, 1202, 1129, 996, 915, 831, 775, 736, 665 cm^{-1}.

3,3-Diallyl-*N*-(*tert*-butyl)-1-(1*H*-pyrazole-3-carbonyl)indoline-2-carboxamide (8g)

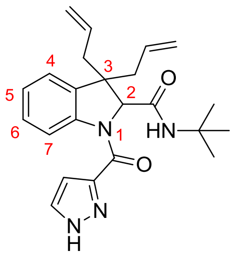

The product was obtained according to the **General procedure B**. After 4 hours at room temperature, volatiles were removed under vacuum and the crude residue was purified by several washing with PE and dried. The desired product was obtained as a pale yellow solid (145 mg, 97%); mp = 123 - 124 °C; R_f = 0.17 (PE/EtOAc 1/1); ^1H NMR (600 MHz, $CDCl_3$, R.T.) δ 8.24 (s, 1H, H_7), 7.72 (d, *J* = 2.3 Hz, 1H, CH_{Py}), 7.30 (t, *J* = 7.7 Hz, 1H, H_6), 7.25 (d, *J* = 6 Hz, 1H, H_4), 7.13 (t, *J* = 7.4 Hz, 1H, H_5), 6.97 (d, *J* = 2.1 Hz, 1H, CH_{Py}), 6.08 (ddt, *J* = 17.0, 10.1, 7.1 Hz, 1H, =C*H*), 5.63 (s, 1H, NH_{tBu}), 5.56 – 5.40 (m, 2H, =C*H*, H_2), 5.16 (m, 2H, =CH_2), 4.88 (dd, *J* = 29.4, 13.5 Hz, 2H, =CH_2), 2.68 (d, *J* = 7.1 Hz, 2H, C$H_{2\ allyl}$), 2.37 (d, *J* = 7.3 Hz, 2H, C$H_{2\ allyl}$), 1.12 (s, 9H, C$H_{3\ tBu}$); $^{13}C\{^1H\}$ NMR (151 MHz, $CDCl_3$, R.T.) δ 170.0 (NH*C*=O), 162.1 (N*C*=O), 146.1 (C_{qPy}), 142.3 (C_q), 136.7 (C_q), 134.4 (=*C*H), 132.8 (=*C*H), 130.6 (*C*H_{Py}), 128.4 (C_6H), 124.8 (C_5H), 124.3 (C_4H), 119.5 (=*C*H_2), 118.9 (=*C*H_2), 117.9 (C_7H), 108.6 (*C*H_{Py}), 73.0 (C_2H), 51.7 ($C_{q\ tBu}$), 51.0 (C_3), 46.1 (*C*$H_{2\ allyl}$), 38.7 (*C*$H_{2\ allyl}$), 28.4 (*C*$H_{3\ tBu}$); HRMS (EI) calcd for $C_{23}H_{28}N_4O_2$ $[M]^+$ 392.2212 found 392.2213; FT-IR (ATR) υ = 3215 (NH), 3075 (CH), 3043 (CH), 2968 (CH), 2928 (CH), 1662 (C=O), 1636 (C=O), 1592, 1523, 1479, 1456, 1364, 1318, 1224, 1116.8, 917, 755 cm^{-1}.

3,3-Diallyl-1-(1-carbamoylcyclopropanecarbonyl)-*N*-(4-methoxyphenyl)indoline-2-carboxamide (8h)

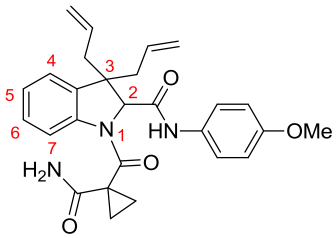

The product was obtained according to the **General procedure B**. After 4 hours at room temperature, purification of the crude residue by column chromatography on SiO_2 (PE/EtOAc 90/10 to 50/50) afford the product as an off-white solid (101 mg, 87%); mp = 121°C; R_f = 0.71 (EtOAc); ^{1}H NMR (400 MHz, $CDCl_3$, R.T.) δ 8.80 (s, 1H, N*H*H), 8.28 (s, 1H, NH*H*), 7.46 (d, *J* = 8.1 Hz, 1H, H_7), 7.35 – 7.24 (m, 3H, CH_3OCC*H*, H_6), 7.19 (d, *J* = 7.6 Hz, 1H, H_4), 7.08 (t, *J* = 7.5 Hz, 1H, H_5), 6.69 (d, *J* = 9.0 Hz, 2H, NHCC*H*), 5.85 (dq, *J* = 10.0, 7.1 Hz, 1H, =C*H*), 5.67 – 5.49 (m, 2H, =CH, N*H*), 5.16 – 4.95 (m, 5H, =CH_2, H_2), 3.73 (s, 3H, OCH_3), 2.61 (d, *J* = 7.1 Hz, 2H, C$H_{2\ allyl}$), 2.48 (dd, *J* = 13.9, 7.4 Hz, 2H, C$H_{2\ allyl}$), 2.04 (br, 1H, C*H*H), 1.79 (br, 1H, CH*H*), 1.59 (br, 1H, C*H*H), 1.25 (br, 1H, CH*H*); ^{13}C{^{1}H} NMR (101 MHz, $CDCl_3$, R.T.) δ = 171.4 (NH_2*C*=O), 168.1 (NH*C*=O), 167.8 (N*C*=O), 156.6 (C_q), 140.3 (C_q), 136.6 (C_q), 133.5 (=*C*H), 132.7 (=*C*H), 130.4 (C_q), 128.8 (C_6H), 125.0 (C_4H), 124.0 (C_5H), 121.7 (NHC*C*H), 119.9 (=*C*H_2), 119.6 (=*C*H_2), 114.0 (CH_3OC*C*H), 71.7 (C_2H), 55.5 (O*C*H_3), 48.7 (C_3), 45.6 (*C*$H_{2\ allyl}$), 40.2 (*C*$H_{2\ allyl}$), 31.6 (C_q), 17.5 (*C*H_2), 16.8 (*C*H_2); HRMS (CI) calcd for $C_{27}H_{30}N_3O_4$ $[M+H]^+$ 460.2236 found 460.2229; FT-IR (ATR) υ = 3310 (NH), 3197 (NH), 3076 (CH), 2930 (CH), 1666 (C=O), 1613 (C=O), 1548, 1510, 1482, 1394, 1298, 1237, 1174, 1032, 919, 829, 750, 472 cm^{-1}.

3,3-Diallyl-1-(2-chloroacetyl)-5-methoxy-*N*-*n*pentylindoline-2-carboxamide (8i)

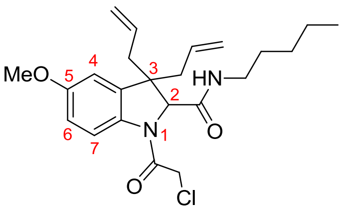

The product was obtained by following the **General procedure B**. After evaporation of the volatiles the crude mixture was purified by column chromatography on SiO_2 using (PE/EtOAc 100/0 to 80/20) as eluent to obtained the desired product as a yellow oil (67 mg, 61%); R_f = 0.27 (PE/EtOAc 4/1); ^{1}H NMR (400 MHz, $CDCl_3$, 55°C) δ 8.00 (br s, 1H, H_7), 6.83 (dd, 1H, *J* = 8.0, 2.5 Hz, H_6), 6.80 (d, 1H, *J* = 2.5 Hz, H_4), 5.94 (ddt, 1H, *J* = 16.4, 9.5, 7.4 Hz, =C*H*), 5.62 (br s, 1H, N*H*), 5.53 (ddt, 1H, *J* = 16.4, 9.5, 7.4 Hz, =C*H*), 5.20-5.05 (m, 4H, =CH_2), 4.66 (s, 1H, H_2), 4.15 (d, 1H, *J* = 13.0 Hz, ClCH*H*), 4.08 (d, 1H, *J* = 13.0 Hz, ClCH*H*), 3.81 (s, 3H, OCH_3), 3.21 (sext., 1H, *J* = 13.3, 7.1, 6.1 Hz, NCH*H*), 3.10 (sext, 1H, *J* = 13.3, 7.1, 6.1 Hz, NCH*H*), 2.64 (d, 2H, *J* = 7.4 Hz, C$H_{2\ allyl}$), 2.44 (ddt, 1H, *J* = 13.9, 7.4, 1.0 Hz, CHH_{allyl}), 2.39 (ddt, 1H, *J* = 13.9, 7.4, 1.0 Hz, CHH_{allyl}), 1.42 (qn, 2H, *J* = 14.7, 7.1 Hz, NCH_2CH_2), 1.27 (m, 2H, CH_3CH_2), 1.18 (m, 2H, CH_3CH_2CH_2), 0.86 (t, 3H, *J* = 6.7 Hz, CH_3); ^{13}C{^{1}H} NMR (150 MHz, $CDCl_3$, R.T.) δ 168.5 (C_q), 164.1 (C_q), 157.5

(C_q), 137.9 (C_q), 134.8 (C_q), 133.3 (*C*H), 132.5 (*C*H), 120.1 (=CH_2), 119.1 (=CH_2), 118.2 (*C*H), 112.9 (*C*H), 110.7 (*C*H), 71.7 (*C*H), 55.8 (CH_3), 51.2 (C_q), 46.6 (CH_2), 42.7 (CH_2), 39.6 (CH_2), 38.3 (CH_2), 29.0 (CH_2), 28.9 (CH_2), 22.3 (CH_2), 14.0 (CH_3); HRMS (CI) calcd. for $C_{23}H_{32}ClN_2O_3$ $[M+H]^+$ 419.2096, found 419.2075; FT-IR (ATR) υ = 3010 (CH), 2930 (N-H), 1648 (C=O), 1488, 1398, 1246, 1204, 1156, 1033, 811 cm^{-1}.

tert-Butyl(2-(3,3-diallyl-2-(tert-butylcarbamoyl)-5-chloroindolin-1-yl)-2-(oxoethyl)carbamate (8j)

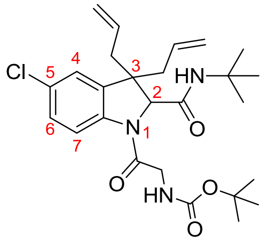

The product was obtained by following the **General procedure B**. After evaporation of the volatiles under vacuum, the residue was purified by column chromatography on SiO_2 using (PE/EtOAc 100/0 to 80/20) as eluent. The desired product was obtained as colourless oil (106 mg, 91%). R_f = 0.64 (PE/EtOAc 4/1); ^{1}H NMR (400 MHz, $CDCl_3$, 55°C) δ 7.89 (br s, 1H, H_7), 7.24 (dd, 1H, J = 8.5, 1.8 Hz, H_6), 7.19 (d, 1H, J = 1.8 Hz, H_4), 6.00 (ddt, 1H, J = 16.8, 8.3, 7.3 Hz, =C*H*), 5.51 (br s, 1H, N*H*), 5.50 (ddt, 1H, J = 16.8, 8.3, 7.3 Hz, =C*H*), 5.31 (br s, 1H, N*H*), 5.23-5.07 (m, 4H, =CH_2), 4.45 (br s, 1H, H_2), 4.09 (dd, 1H, J = 16.8, 15.4 Hz, NCH*H*), 3.93 (br d, 1H, J = 15.4 Hz, NCH*H*), 2.67 (dd, 1H, J = 15.4, 7.3 Hz, CH*H* $_{allyl}$), 4.49 (dd, 1H, J = 15.4, 7.3 Hz, CH*H* $_{allyl}$), 2.41 (dd, 1H, J = 15.4, 7.3 Hz, CH*H* $_{allyl}$), 2.34 (dd, 1H, J = 15.4, 7.3 Hz, CH*H* $_{allyl}$), 1.48 (s, 9H, $CH_{3\ tBu}$), 1.33 (s, 9H, $CH_{3\ tBu}$); $^{13}C\{^1H\}$ NMR (150 MHz, $CDCl_3$, R.T.) δ 167.5 (C_q), 167.1 (C_q), 155.8 (C_q), 140.3 (C_q), 138.2 (C_q), 133.3 (*C*H), 132.5 (*C*H), 129.7 (C_q), 128.5 (*C*H), 124.4 (*C*H), 120.1 (=CH_2), 119.9 (=CH_2), 80.2 (C_q), 71.1 (*C*H), 52.3 (C_q), 50.8 (C_q), 45.8 (CH_2), 44.1 (CH_2), 38.2 (CH_2), 28.6 ($CH_{3\ tBu}$), 28.4 ($CH_{3\ tBu}$); C_7H was not observed by $^{13}C\{^1H\}$ NMR; HRMS (CI) calcd. for $C_{26}H_{37}N_3O_4Cl$ $[M+H]^+$ 490.2467, found 490.2458; FT-IR (ATR) υ = 3455 (N-H), 3348 (N-H), 2977 (CH), 2940 (CH), 1667 (C=O), 1476, 1394, 1366, 1251 cm^{-1}.

tert-Butyl (2-(3,3-diallyl-2-(tert-butylcarbamoyl)indolin-1-yl)-2-oxoethyl)carbamate (8k)

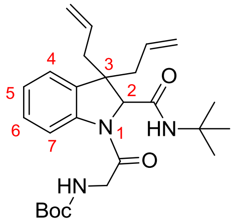

The product was obtained according to the **General procedure B**. After 4 hours at room temperature, purification of the crude residue was purified by washing with cold Petroleum ether and dried under vacuum to yield the product as a white solid (107 mg, 93%); mp = 155 - 156 °C; R_f = 0.43 (PE/EtOAc 70/30); ^{1}H NMR (400 MHz, $CDCl_3$, 55°C) δ 7.89 (br s, 1H, H_7), 7.34 – 7.15 (m, 3H, H_4, H_6, H_7), 7.09 (t, J = 7.5 Hz, 1H, H_5), 6.02 (td, J = 16.0, 7.9 Hz, 1H, =C*H*), 5.55 – 5.38 (m, 2H, =C*H*, N*H*), 5.33 (br s, 1H, N*H*), 5.17 (d, J = 12.3 Hz, 2H, =CH_2), 5.11 – 4.98 (m, 2H, =CH_2), 4.45 (s, 1H, H_2), 4.11 (d, J = 16.5 Hz, 1H, C*H*HNH), 4.03 – 3.88 (m, 1H, CH*H*NH), 2.64 (m, 2H, $CH_{2\ allyl}$), 2.39 (m, 2H, $CH_{2\ allyl}$), 1.47 (s, 9H, $CH_{3\ tBu}$), 1.29 (s, 9H, $CH_{3\ tBu}$); $^{13}C\{^1H\}$ NMR (150 MHz, $CDCl_3$, R.T.) δ 167.5 (*C*=O), 167.4 (*C*=O), 155.8 (N(*C*=O)O), 133.7 (=*C*H), 133.0 (=*C*H), 128.6 (C_6H), 124.7 (C_5H), 124.2

(C_4H), 119.7 (=CH_2), 119.4 (=CH_2), 80.0 (C_q), 71.5 (C2H), 52.1 (C_q), 50.9 (C_q), 45.9 ($CH_{2\ allyl}$), 44.3 (C_q), 42.4 ($CH_{2\ allyl}$), 38.5 (COCH_2), 28.5 ($CH_{3\ tBu}$), 28.4 ($CH_{3\ tBu}$), C_7H was not observed in $^{13}C\{^1H\}$ NMR; HRMS (EI) calcd for $C_{26}H_{37}N_3O_4$ $[M]^+$ 455.2784 found 455.2781; FT-IR (ATR) υ = 3330 (NH), 3300 (NH), 3074 (CH), 2975 (CH), 2930 (CH), 1669 (C=O), 1647 (C=O), 1527, 1481, 1454, 1406, 1364, 1238, 1163, 916, 752 $cm^{-1.}$

3,3-Diallyl-*N*-(*tert*-butyl)-1-(2-(methylamino)acetyl)indoline-2-carboxamide (8l)

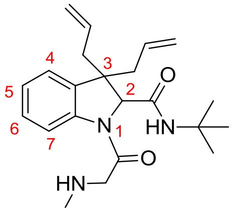

The product was obtained according to the **General procedure for B** with slight modifications. After a week at 50°C, purification of the crude residue was purified by column chromatography on silica (PE/EtOAc/NEt_3 90/10/1 to PE/EtOAc/NEt_3 10/90/1) to give the desired compound as a white solid (78 mg, 87%); mp = 127 - 128 °C; ^{1}H NMR (400 MHz, $CDCl_3$, R.T.) δ 7.98 (s, 1H, H_7), 7.34 – 7.15 (m, 2H, H_4, H_6), 7.08 (t, J = 7.5 Hz, 1H, H_5), 6.04 (dd, J = 9.5, 6.4 Hz, 1H, =CH), 5.46 (m, 2H, =CH, NH), 5.16 (br d, J = 12.7 Hz, 2H, =CH_2), 5.03 (br d, J = 12.6 Hz, 2H, =CH_2), 4.48 (s, 1H, H_2), 3.59 (d, J = 16.3 Hz, 1H, NHCHH), 3.40 (d, J = 16.3 Hz, 1H, NHCHH), 2.75 – 2.46 (m, 6H, NHCH_3, NHCH$_3$, C$H_{2\ allyl}$), 2.38 (m, 2H, C$H_{2\ allyl}$), 1.27 (s, 9H, C$H_{3\ tBu}$); $^{13}C\{^1H\}$ NMR (101 MHz, $CDCl_3$, R.T.) δ 169.8 (NHC=O), 168.1 (NC=O), 141.7 (C_q), 136.5 (C_q), 134.1 (=CH), 133.2 (=CH), 128.6 (C_6H), 124.6 (C_5H), 124.3 (C_4H), 119.3 (=CH_2), 119.0 (=CH_2), 116.6 (C_7H), 71.7 (C_2H), 54.2 (NHCH_2), 52.0 (C_3), 50.7 (C_q), 46.2 ($CH_{2\ allyl}$), 38.7 ($CH_{2\ allyl}$), 36.4 (NCH_3), 28.7 ($CH_{3\ tBu}$); HRMS (CI) calcd for $C_{22}H_{32}N_3O_2$ $[M+H]^+$ 370.2495 found 370.2490; FT-IR (ATR) υ = 3310 (NH), 3072 (CH), 2970 (CH), 1654 (C=O), 1595, 1548, 1480, 1395, 1220, 996, 914, 750 cm^{-1}.

3,3-Diallyl-1-((S)-2-amino-3-phenylpropanoyl)-*N*-(*tert*-butyl)indoline-2-carboxamide (8m)

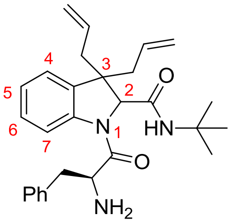

The product was obtained according to the **General procedure B** with slight modifications. After one week at 50°C volatiles were removed under vacuum and the crude residue was purified by column chromatography on SiO_2 (PE/EtOAc/NEt_3: 90/10/1 to 30/70/1) to afford the UGI product as an pale yellow oil (80 mg, 68%) and a mixture of two inseparable diastereoisomers (A/B : ratio 1/0.8); ^{1}H NMR ($CDCl_3$,400 MHz, 55°C) δ 8.20 (br s, 1H, H_{7A}), 8.02 (br s, 0.7H, H_{7B}), 7.41 – 6.96 (m, 14.4H, CH_{PhA}, H_{4A}, H_{5A}, H_{6A}, CH_{PhB}, H_{4B}, H_{5B}, H_{6B}), 6.11 (dt, J = 17.5, 7.5 Hz, 0.8H, =CH_B), 6.05 – 5.89 (m, 1H, =CH_A), 5.67 – 5.33 (m, 3.5H, =CH_A, NH_A, =CH_B, NH_B), 5.28 – 5.08 (m, 3.6H, =CH_{2A}, =CH_{2B}), 5.08 – 4.89 (m, 3.6H, =CH_{2A}, =CH_{2B}), 4.81 (br s, 0.8H, H_{2B}), 4.29 (br s, 1H, H_{2A}), 3.94 (br s, 0.7H, NH_2CH_B), 3.81 (br s, 1H, NH_2CH_A), 3.16 (dd, J = 12, 4 Hz, 0.8H, PhCHH_B), 3.13 (dd, J = 18.1, 4.5 Hz,

1H, PhCHH_A), 2.89 – 2.75 (m, 2.8H, PhCHH_A, PhCHH_B), 2.70 – 2.63 (m, 1.6H, C$H_{2\ allylB}$), 2.59 (d, J = 7.1 Hz, 2H, C$H_{2\ allylA}$), 2.35 (d, J = 6.7 Hz, 1.6H, C$H_{2\ allylB}$), 2.23 – 2.09 (m, 2H, C$H_{2\ allylA}$), 1.85 (br s, 4H, NH_{2A}, NH_{2B}), 1.29 (s, 9H, C$H_{3\ tBuA}$), 1.23 (s, 5.4H, C$H_{3\ tBuB}$); ^{13}C{1H} NMR (101 MHz, $CDCl_3$, 55°C) δ 174.6 (*C*=O), 172.8 (*C*=O), 168.1 (*C*=O), 141.4 (C_q), 137.6 (=*C*H), 137.5 (=*C*H), 134.0, 133.7, 133.1, 132.9, 129.6, 129.3, 128.6, 128.5, 128.4, 128.3, 126.8, 126.4, 124.4, 124.2, 124.1, 119.2 (=CH_2), 119.1 (=CH_2), 118.8 (=CH_2), 118.7 (=CH_2), 72.4 (C_2H), 72.3 (C_2H), 55.7 (NH_2*C*H), 55.3 (NH_2*C*H), 51.8 (C_q), 51.6 (C_q), 45.2 ($CH_{2\ allyl}$), 45.1 ($CH_{2\ allyl}$), 42.2 (PhCH_2), 41.4 (PhCH_2), 38.2 ($CH_{2\ allyl}$), 37.7 ($CH_{2\ allyl}$), 28.5 ($CH_{3\ tBu}$); HRMS (CI) calcd for $C_{28}H_{35}N_3O_2$ $[M+H]^+$ 446.2806 found 446.2802; FT-IR (ATR) υ = 3310 (NH), 3068 (CH), 2970 (CH), 2924 (CH), 2874 (CH), 1657 (C=O), 1634 (C=O), 1479, 1455, 1362, 1219, 915, 749, 698, 503 cm^{-1}.

3,3-Diallyl-*N*-(*tert*-butyl)-1-((R)-2-hydroxy-2-phenylacetyl)-5-methoxyindoline-2-carboxamide (8n)

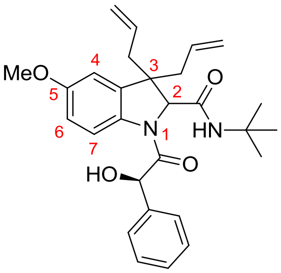

The product was obtained according to the **General procedure B**. NMR analysis revealed a mixture of two diastereoisomers (1/1). Purification of the crude residue by column chromatography on SiO_2 (PE/Et_2O: 100/0 to 30/70) allowed separation of the two diastereoisomers (diast 1 = 48 mg, diast 2 = 41 mg, 87%).

Diastereoisomer 1: Pale yellow oil; R_f = 0.35 (PE/EtOAc 70/30); ^{1}H NMR ($CDCl_3$,600 MHz, R.T.) δ 8.26 (d, J = 8.8 Hz, 1H, H_7), 7.31 - 7.42 (m, 5H, CH_{Ph}), 6.82 (dt, J = 8.8; 2.4 Hz, 1H, H_6), 6.75 (d, J = 2.4 Hz, 1H, H_4), 5.95 (ddd, J = 16.3; 9.3; 5.7 Hz, 1H, =C*H*), 5.30 (s, 1H, N*H*), 5.12 (m, 2H, =CH_2), 5.00 (dt, J = 20.9; 7.0 Hz, 1H, =C*H*), 4.96 (d, J = 7.0 Hz, 1H, C*H*OH), 4.59 (d, J = 10.2 Hz, 1H, =C*H*H), 4.49 (d, J = 7.0 Hz, 1H, O*H*), 4.25 (d, J = 17.0 Hz, 1H, =CH*H*), 4.19 (s, 1H, NC*H*), 3.78 (s, 3H, OCH_3), 2.53 (dd, J = 14.7; 5.4 Hz, 1H, C*H*H_{allyl}), 2.44 (dd, J = 14.7; 8.6 Hz, 1H, CHH_{allyl}), 1.96 (dd, J = 13.8; 7.4 Hz, 1H, C*H*H_{allyl}), 1.81 (dd, J = 13.8, 7.4 Hz, 1H, CHH_{allyl}), 1.29 (s, 9H, C$H_{3\ tBu}$); ^{13}C{^{1}H} NMR ($CDCl_3$,150 MHz, R.T.) δ 171.0 (N*C*=O), 167.8 (NH*C*=O), 157.4 (C_q), 138.1 (C_q), 138.0 (C_q), 134.0 (C_q), 133.5 (=*C*H), 131.4 (=*C*H), 129.4 (CH_{Ph}), 129.3 (CH_{Ph}), 128.0 (CH_{Ph}), 119.8 (=CH_2), 119.4 (=CH_2), 117.8 (C_7H), 112.8 (C_6H), 110.9 (C_4H), 73.1 (*C*HOH), 70.6 (N*C*H), 55.7 (OCH_3), 52.1 (C_q), 50.8 (C_q), 45.2 (CH_2), 38.0 (CH_2), 28.5 ($CH_{3\ tBu}$); HRMS (CI) calcd for $C_{28}H_{34}N_2O_4$ $[M+H]^+$ 463.2597, found 463.2579; FT-IR (ATR) υ = 3419 (OH), 2966 (CH), 2927 (CH), 1679 (C=O), 1650 (C=O), 1486, 1454, 1365, 1270, 1198, 1182, 1062, 1030, 918, 699, 513, 490, 451 cm^{-1}.

Diastereoisomer 2: White solid; mp = 113 - 114°C; R_f = 0.20 (PE/EtOAc 70/30); ^{1}H NMR ($CDCl_3$, 400 MHz, 55°C) δ 7.80 (br s, 1H, H_7), 7.23 - 7.46 (m, 5H, CH_{Ph}), 6.79 - 6.81 (m, 2H, H_4, H_6), 5.97 - 6.21 (m, 1H, =C*H*), 5.32 - 5.54 (m, 2H, =C*H*, C*H*OH), 4.88 - 5.27 (m, 5H, =CH_2, N*H*), 4.55 (s, 1H, NC*H*), 4.20 (d, J = 6.2 Hz, 1H, O*H*), 3.81 (s, 3H, OCH_3), 2.58 (ddd, J = 23.0, 14.0, 7.0 Hz, 2H, C$H_{2\ allyl}$), 2.26 (d, J = 5.2

Hz, 2H, CH_2 $_{allyl}$), 0.96 (br s, 9H, CH_3 $_{tBu}$); $^{13}C\{^1H\}$ NMR (CDCl$_3$,100 MHz, 55°C) δ 171.0 (N*C*=O), 166.7 (NH*C*=O), 138.3 (C_q), 133.9 (=*C*H), 132.7 (=*C*H), 128.9 (*C*H_{Ph}), 128.7 (*C*H_{Ph}), 127.6 (*C*H_{Ph}), 119.9 (=*C*H_2), 118.8 (=*C*H_2), 112.5 (C_6H), 110.8 (C_4H), 72.9 (N*C*H), 55.6 (O*C*H_3), 51.3 (C_q), 44.6 (*C*H_2), 37.6 (*C*H_2), 28.3 (*C*H_3 $_{tBu}$), C_7H not observed by $^{13}C\{^1H\}$ NMR; HRMS (CI) calcd for $C_{28}H_{34}N_2O_4$ $[M+H]^+$ 463.2597, found 463.2543; FT-IR (ATR) υ = 3328 (OH), 3072 (CH), 2963 (CH), 1686 (C=O), 1630 (C=O), 1597, 1453, 1270, 1252, 1198, 1030, 804, 624, 568 cm^{-1}.

4. Synthesis of substituted 3,3-diallyl-2-hydroxyindoline

General procedure C: The chloroformate or acid chloride (1 eq.) was added to a solution of the 3,3-diallyl-*3H*-indole in CH_2Cl_2 (C ≈ 0.07 mol/L) and left to stir for 30 minutes at room temperature before addition of $NaHCO_{3sat}$. After extraction of the reaction mixture with CH_2Cl_2, the combined organic layers were washed with water, dried over Na_2SO_4 and filtered through cotton wool. Pure compounds were obtained by evaporation of the volatiles under reduced pressure or by purification by column chromatography on silica.

Methyl 3,3-diallyl-2-hydroxyindoline-1-carboxylate (10a)

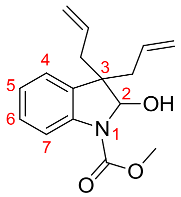

The product was obtained by following the **General procedure C**. After evaporation of the volatiles the crude mixture was purified by column chromatography on SiO_2 using (PE/EtOAc 100/0 to 70/30) as eluent to obtained the desired product as a yellow oil (78 mg, 94%); R_f = 0.47 (PE/EtOAc 4/1); ^1H NMR (400 MHz, CDCl$_3$, 60°C) δ 7.65 (br s, 1H, H_7), 7.23 (td, 1H, J = 7.4, 1.2 Hz, H_6), 7.12 (d, 1H, J = 7.4 Hz, H_4), 7.03 (td, 1H J = 7.4, 1.2 Hz, H_5), 5.99 (dtd, 1H, J = 17.8, 10.7, 7.3 Hz, =C*H*), 5.61 (s, 1H, H_2), 5.56 (dtd, 1H, J = 17.8, 10.7, 7.3 Hz, =C*H*), 5.14 (tdd, 2H, J = 17.8, 10.7, 1.6 Hz, =CH_2), 5.03 (tdd, 2H, J = 17.8, 10.7, 1.6 Hz, =CH_2), 3.93 (s, 3H, OCH_3), 2.65 (d, 2H, J = 7.2 Hz, CH_2 $_{allyl}$), 2.39 (dd, 1H, J = 13.9, 7.2 Hz, C*H*H_{allyl}), 2.32 (dd, 1H, J = 14.1, 7.7 Hz, CHH_{allyl}); $^{13}C\{^1H\}$ NMR (100 MHz, CDCl$_3$, 60°C) δ 139.8 (C_q), 135.1 (=*C*H), 132.9 (=*C*H), 129.6 (C_q), 128.0 (*C*H), 123.8 (*C*H), 122.8 (*C*H), 118.5 (=*C*H_2), 117.9 (=*C*H_2), 114.5 (*C*H), 89.8 (C_2H), 52.7 (*C*H_3), 50.0 (C_3), 42.8 (*C*H_2 $_{allyl}$), 36.9 (*C*H_2 $_{allyl}$); *C*=O was not observed by $^{13}C\{^1H\}$ NMR; HRMS (CI) calcd for $C_{16}H_{18}NO_2$ $[M-OH]^+$ 256.1332, found 256.1333; FT-IR (ATR) υ = 3437 (OH), 3074 (CH), 2977 (CH), 2955 CH), 2916 (CH), 1686 (C=O), 1482, 1443, 1382, 1059, 952 cm^{-1}.

Methyl 3,3-diallyl-2-hydroxy-5-methoxyindoline-1-carboxylate (10b)

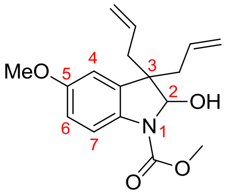

The product was obtained by following the **General procedure C**. After evaporation of the volatiles the crude mixture was purified by column chromatography on SiO_2 using (PE/EtOAc 100/0 to 70/30) as eluent to obtained the desired product as an orange oil (91 mg, 96%); R_f = 0.44 (PE/EtOAc 4/1); ^{1}H NMR (600 MHz, $CDCl_3$, 55°C) δ 7.54 (br s, 1H, H_7), 6.78 (dd, 1H, J = 9.0, 2.7 Hz, H_6), 6.71 (d, 1H, J = 2.7 Hz, H_4), 6.00 (ddt, 1H, J = 17.0, 10.2, 7.5 Hz, =CH_2), 5.57 (ddt, 2H, J = 17.0, 10.2, 7.5 Hz, =CH_2, H_2), 5.17-5.02 (m, 4H, =CH_2), 3.91 (s, 3H, C(O)OCH_3), 3.79 (s, 3H, OCH_3), 2.65 (dd, 1H, J = 14.3, 7.5 Hz, CHH_{allyl}), 2.60 (dd, 1H, J = 14.3, 7.5 Hz, CHH$_{allyl}$), 2.38 (dd, 1H, J = 14.0, 7.5 Hz, CHH_{allyl}), 2.31 (dd, 1H, J = 13.7, 7.5 Hz, CHH$_{allyl}$); ^{13}C{^{1}H} NMR (150 MHz, $CDCl_3$, R.T.) δ 156.0 (C=O), 137.1 (C_q), 136.5 (C_q), 135.5 (=CH), 133.9 (C_q), 132.9 (=CH), 119.0 (=CH$_2$), 118.5 (=CH$_2$), 115.8 (CH), 112.5 (CH), 110.8 (CH), 90.4 (C_2H), 55.7 (CH$_3$), 54.6 (C_3), 52.5 (CH$_3$), 47.6 (CH$_2$), 37.3 (CH$_2$); HRMS (CI) calcd $C_{17}H_{20}NO_3$ $[M\text{-}OH]^+$ 286.1438 , found 286.1439; FT-IR (ATR) υ = 3435 (OH), 3074 (CH), 2954 (CH), 2917 (CH), 2849 (CH), 1689 (C=O), 1489, 1459, 1434, 1269, 1031, 998 cm^{-1}.

Isobutyl 3,3-diallyl-2-hydroxy-5-methoxyindoline-1-carboxylate (10c)

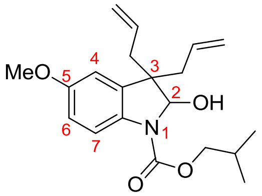

The product was obtained by following the **General procedure C**. After work up and evaporation of the volatiles the crude mixture was purified by column chromatography on SiO_2 using (PE/EtOAc 100/0 to 70/30) as eluent to obtained the desired product as a yellow oil (67 mg, 82%); R_f = 0.45 (PETh/EtOAc 4/1); NMR at R.T. showed a mixture of two rotamers (1/1); ^{1}H NMR (400 MHz, $CDCl_3$, 55°C) δ 7.53 (br s, 1H, H_7), 6.76 (d, J = 8.8 Hz, 1H, H_6), 6.70 (s, 1H, H_4), 6.09 – 5.89 (m, 1H, =CH), 5.68 – 5.44 (m, 2H, =CH, H_2), 5.13 (t, J = 14.2 Hz, 2H, =CH_2), 5.01 (t, J = 12.1 Hz, 2H, =CH_2), 4.20 – 3.97 (m, 2H, OCH_2), 3.79 (s, 3H, OCH_3), 2.71 – 2.51 (m, 2H, C$H_{2\ allyl}$), 2.42 – 2.24 (m, 2H, C$H_{2\ allyl}$), 2.15 – 2.02 (m, 1H, CH_{iPr}), 1.03 (d, J = 6.5 Hz, 6H, C$H_{3\ iPr}$); ^{13}C{^{1}H} NMR (151 MHz, $CDCl_3$, R.T.) δ 155.9 (br, C=O), 154.8 (br, C=O), 137.1 (br, C_q), 136.6 (br, C_q), 135.2 (=CH), 133.1 (=CH), 118.9 (=CH$_2$), 118.5 (br, =CH$_2$), 115.1 (br, C_7H), 112.5 (C_6H), 110.8 (br, C_4H), 90.2 (C_2H), 89.8 (C_2H), 72.6 (OCH$_2$), 71.8 (OCH$_2$), 55.9 (OCH$_3$), 50.5 (C_3), 49.8 ($C_{3)}$, 42.9 (br, CH$_{2\ allyl}$), 37.0 (br, CH$_{2\ allyl}$), 28.1 (CH$_{3\ iPr}$), 19.4 (CH$_{iPr}$); HRMS (CI) calcd for $C_{20}H_{28}NO_4$ $[M+H]^+$ 345.1940 found 345.19384; FT-IR (ATR) υ = 3425 (OH), 2960 (CH), 2928 (CH), 2874 (CH), 2834 (CH), 1677 (C=O), 1638 (C=O), 1490, 1466, 1435, 1406, 1384, 1321, 1263, 1206, 1183, 1126, 1053, 1033, 999, 911, 864, 807, 761, 731, 687 cm^{-1}.

1-(3,3-Diallyl-2-methoxyindolin-1-yl)-2-phenylethanone (11d)

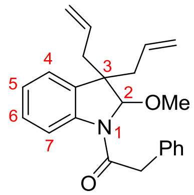

Phenyl acetyl chloride (21 µL, 0.16 mmol, 1 eq.) was added to a solution of 3,3-diallyl-5-methoxy-*3H*-indole (31 mg, 0.16 mmol) in dichloromethane. After 30 minutes at room temperature methanol (0.1 mL) was added and the mixture was stirred for 30 minutes before evaporation of the volatiles under reduced pressure. The crude residue obtained was purified by column chromatography on silica (PE/EtOAc 100/0 to 95/5) to afford the pure compound as a colourless oil (28.5 mg, 51%); NMR at R.T. showed a mixture of two rotamers (1/1); R_f = 0.53 (PE/EtOAc 9/1); ^1H NMR (400 MHz, $CDCl_3$, 55°C) δ 7.72 (br s, 1H, H_7), 7.35 - 6.99 (m, 8H, CH_{Ph}, H_4, H_5, H_6), 5.91 (td, *J* = 16.7, 8.3 Hz, 1H, =C*H*), 5.37 (m, 2H, =C*H*, H_2), 5.14 (m, 2H, =CH_2), 4.83 (m, 2H, =CH_2), 3.93 (s, 2H, COCH_2), 3.38 (s, 3H, OCH_3), 2.76 – 2.42 (m, 2H, C$H_{2\ allyl}$), 2.25 – 1.94 (m, 2H, C$H_{2\ allyl}$); $^{13}C\{^1H\}$ NMR (151 MHz, $CDCl_3$, R.T.) δ 171.0 (*C*=O), 170.2 (*C*=O), 141.5 (C_q), 140.1 (C_q), 139.2 (C_q), 136.4 (C_q), 134.7 (C_q), 134.6 (br, =CH), 134.5 (C_q), 132.7 (=*C*H), 129.3 (*C*H), 128.8 (*C*H), 128.2 (*C*H), 127.7 (*C*H), 127.2 (*C*H), 127.1 (*C*H), 124.6 (*C*H), 124.3 (*C*H), 123.0 (*C*H), 119.0 (=*C*H_2), 118.8 (*C*H_2), 117.6 (C_7H), 116.7 (*C*H), 97.3 (C_2H), 95.9 (C_2H), 57.6 (O*C*H_3), 54.5 (O*C*H_3), 51.0 (C_3), 50.4 (C_3), 42.4 (CO*C*H_2), 42.1 (CO*C*H_2), 41.5 (*C*$H_{2\ allyl}$), 41.0 (*C*$H_{2\ allyl}$), 35.5 (*C*$H_{2\ allyl}$); HRMS (CI) calcd for $C_{23}H_{26}NO_2$ $[M+H]^+$ 348.1963 found 438.1961; FT-IR (ATR) υ = 3072 (CH), 3030 (CH), 2978 (CH), 2936 (CH), 2834 (CH), 1662 (C=O), 1599, 1495, 1476, 1379, 1100, 1075, 996, 914, 751, 718, 694 cm^{-1}.

Methyl 3,3-diallyl-2-methyleneindoline-1-carboxylate (12)

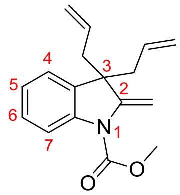

The product was obtained by following the **General procedure C**. After evaporation of the volatiles the product was obtained as a dark purple oil (280 mg, 86%); R_f = 0.58 (PE/EtOAc 4/1); ^1H NMR (600 MHz, $CDCl_3$, R.T.) δ 7.76 (d, 1H, *J* = 7.6 Hz, H_7), 7.20 (td, 1H, *J* = 7.6, 1.5 Hz, H_6), 7.13 (dd, 1H, *J* = 7.6, 0.9 Hz, H_4), 7.07 (td, 1H, *J* = 7.6, 0.9 Hz, H_5), 5.82 (s, 1H, C_2CH*H*), 5.43 (ddt, 2H, *J* = 17.2, 10.2, 7.2 Hz, =C*H*), 4.93-4.87 (m, 4H, =CH_2), 4.62 (d, 1H, *J* = 1.5 Hz, C_2CH*H*), 3.95 (s, 3H, C(O)OCH_3), 2.56 (dd, 2H, *J* = 13.8, 7.2 Hz, C$H_{2\ allyl}$), 2.43 (dd, 2H, *J* = 13.8, 7.2 Hz, C$H_{2\ allyl}$); $^{13}C\{^1H\}$ NMR (100 MHz, $CDCl_3$, 55°C) δ 153.5 (C=O), 150.2 (C_q), 141.7 (C_q), 133.8 (C_q), 133.2 (*C*H), 127.9 (*C*H), 123.5 (*C*H), 123.2 (*C*H), 118.4 (=*C*H_2), 94.3 (=*C*H_2), 53.0 (*C*H_3), 52.2 (C_3), 46.6 (*C*H_2); HRMS (CI) calcd for $C_{17}H_{20}NO_2$ $[M+H]^+$ 270.1494 found, 270.1481; FT-IR (ATR) υ = 3020 (CH), 2938 (CH), 1714 (C=O), 1481, 1354, 1237, 1094 cm^{-1}.

5. Synthesis of 2,3-diallylindoles

General procedure D: Preparation of 2,3-diallylindole from 2-hydroxy-3,3-diallylindoline (13)

Aluminium chloride (1.1 eq.) was added to a solution 3,3-diallyl-2-hydroxyindoline (1.0 eq.) in CH_2Cl_2 (C ≈ 1 mol/L) at room temperature. The mixture was stirred for 30 minutes before addition of NEt_3 (≈ 2eq.). After 5 minutes at room temperature water was added and the product was extracted with CH_2Cl_2 (3 times). The combined organic layers were dried over Na_2SO_4. After evaporation, the crude material was purified by filtration through a small pad of SiO_2 to yield the rearranged product.

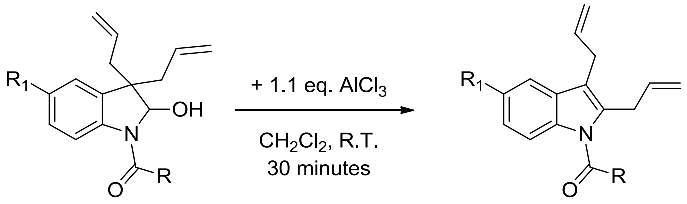

Methyl 2,3-diallyl-1*H*-indole-1-carboxylate (13a)

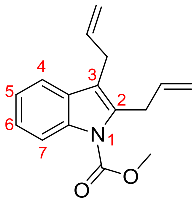

The product was obtained by following the **General procedure D**. After evaporation, the crude material was purified by filtration through a small pad of silica to yield the rearranged product as a colourless oil (120 mg, 97%); Rf = 0.3 (PE/Et2O 90/10); ^{1}H NMR (600 MHz, $CDCl_3$, R.T.) δ 8.09 (d, J = 8.2 Hz, 1H, H_7), 7.48 (d, J = 7.3 Hz, 1H, H_4), 7.37 – 7.16 (m, 2H, H_5, H_6), 6.08 – 5.83 (m, 2H, =C*H*), 5.19 – 4.84 (m, 4H, =CH_2), 4.02 (s, 3H, OCH_3), 3.79 (dt, J = 5.6, 1.4 Hz, 2H, C$H_{2\ allyl}$), 3.44 (dt, J = 5.8, 1.4 Hz, 2H, C$H_{2\ allyl}$); ^{13}C{^{1}H} NMR (151 MHz, $CDCl_3$) δ 152.5 (*C*=O), 136.0 (=*C*H), 135.8 (=*C*H), 134.8 (C_q), 130.1 (C_q), 124.1 ($C_{5/6}$H), 122.9 ($C_{5/6}$H), 118.7 (C_4H), 117.8 (C_q), 115.7 (=*C*H_2), 115.6 (=*C*H_2), 115.5 (C_7H), 53.5 (O*C*H_3), 30.7 (*C*$H_{2\ allyl}$), 28.4 (*C*$H_{2\ allyl}$); HRMS (EI) calcd for $C_{16}H_{17}NO_2$ $[M]^+$ 255.12593 found 255.12581; FT-IR (ATR) υ = 3076 (CH), 3005 (CH), 2954 (CH), 2925 (CH), 2852 (CH), 1732 (C=O), 1638 (C=C), 1475, 1457, 1385, 1355, 1284, 1264, 1227, 1187, 1187, 1134, 1066, 1024, 909, 817, 412 cm^{-1}.

Isobutyl 2,3-diallyl-1*H*-indole-1-carboxylate (13c)

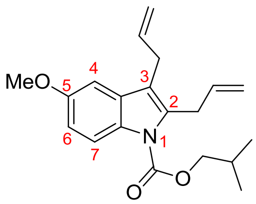

The product was obtained by following the **General procedure D**. After evaporation, the crude material was purified by filtration through a small pad of silica to yield the rearranged product as a colourless oil (20 mg, 97%); Rf = 0.35 (PE/Et_2O 90/10); ^{1}H NMR (600 MHz, $CDCl_3$) δ 8.00 (d, J = 9.0 Hz, 1H, H_7), 6.93 (d, J = 2.5 Hz, 1H, H_4), 6.87 (dd, J = 9.0, 2.6 Hz, 1H, H_6), 6.00 (ddt, J = 16.9, 10.3, 5.6 Hz, 1H, =C*H*), 5.96 – 5.09 (m, 1H, =C*H*), 5.11 – 4.91 (m, 4H, =CH_2), 4.20 (d, J = 6.6 Hz, 2H,

OCH_2), 3.85 (s, 3H, OCH_3), 3.79 (dt, J = 5.6, 1.4 Hz, 2H, C$H_{2\ allyl}$), 3.40 (dt, J = 6.0, 1.5 Hz, 2H, C$H_{2\ allyl}$), 2.16 (m, 1H, CH_{iPr}), 1.05 (d, J = 6.7 Hz, 6H, C$H_{3\ iPr}$); ^{13}C{^{1}H} NMR (151 MHz, $CDCl_3$) δ 156.0 (C_q), 152.0 (C_q), 135.9 (=CH), 135.8 (=CH), 135.7 (C_q), 131.0 (C_q), 130.6 (C_q), 117.5 (C_q), 116.9 (CH), 115.7 (=CH$_2$), 115.5 (=CH$_2$), 112.0 (CH), 101.8 (CH), 73.3 (OCH$_2$), 55.8 (OCH$_3$), 30.7 (CH$_{2\ allyl}$), 28.4 (CH$_{2\ allyl}$), 28.0 (CH $_{iPr}$), 19.5 (CH$_{3\ iPr}$); HRMS (CI) calcd for $C_{20}H_{26}NO_3$ $[M+H]^+$ 328.19127 found 328.19681; FT-IR (ATR) υ = 3079 (CH), 2959 (CH), 2928 (CH), 2874 (CH), 1726 (C=O), 1637 (C=C), 1607, 1476, 1454, 1437, 1400, 1382, 1355, 1323, 1283, 1264, 1237, 1210, 1176, 1118, 1103, 1041, 1017 cm^{-1}.

Preparation of 2,3-diallylindole starting from 3,3-diallylindolinine

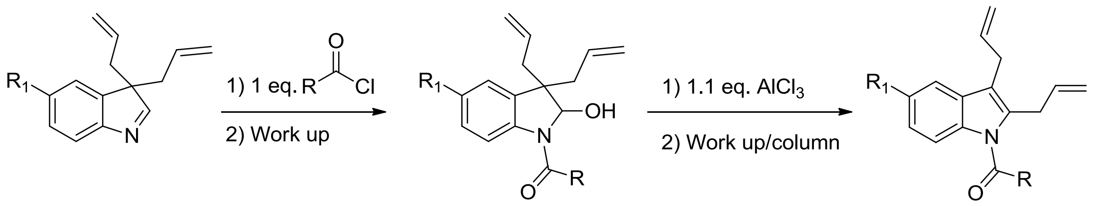

General procedure E: Using an acyl chloride

The acyl chloride (1 eq.) was added to a solution of 3,3-diallyl-*3H*-indole (1 eq.) in CH_2Cl_2 (C ≈ 0.3 mol/L) at room temperature. After 30 minutes, the reaction was quenched by addition of water and the mixture was extracted with CH_2Cl_2 (3 times). The combined organic layers were dried over Na_2SO_4 and filtered. Evaporation of the volatiles give the 3,3-diallyl-2-hydroxyindoline which was directly dissolved in CH_2Cl_2 (C ≈ 0.3 mol/L) and $AlCl_3$ (1.1 eq.) was added. After 30 minutes at room temperature the reaction was quenched with $NaHCO_{3sat}$ before extraction with CH_2Cl_2 (3 times). The combined organic phases were washed with water, dried over Na_2SO_4 and filtered. After evaporation of the volatiles under vacuum, the crude residue was purified by a filtration on a small pad of SiO_2 using PE/Et_2O (90/10) as eluent.

General procedure F: Using a chloroformate

The chloroformate (1 eq.) was added to a solution of 3,3-diallyl-*3H*-indole (1 eq.) in CH_2Cl_2 (C ≈ 0.2 mol/L) at room temperature. After 30 minutes, the reaction was quenched by addition of $NaHCO_{3\ sat}$ and the mixture was extracted with CH_2Cl_2 (3 times). The combined organic layers were washed with water and dried over Na_2SO_4 before filtration. Evaporation of the volatiles gave 3,3-diallyl-2-hydroxy-indoline derivative which was directly dissolved in CH_2Cl_2 (C ≈ 0.2 mol/L) and $AlCl_3$ (1.1 eq.) was added. After 30 minutes at room temperature NEt_3 (≈ 2 eq.) was added. The solution was left to stir for 5 minutes before addition of a saturated solution of K_2CO_3 and extraction with CH_2Cl_2 (3 times). The combined

organic phases were washed with water, dried over Na_2SO_4 and filtered. After evaporation of the volatiles under vacuum, the crude residue was purified by filtration through a small pad of SiO_2 using PE/Et_2O (100/0 to 90/10) as eluent.

1-(2,3-Diallyl-5-methoxy-1*H*-indol-1-yl)-2-phenylethanone (13d)

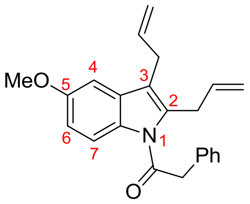

The product was obtained by following the **General procedure E**. After evaporation of the volatiles under vacuum, the crude residue was purified by a filtration on a small pad of SiO_2 using Et_2O as eluent. The pure product was obtained as pale yellow oil (82 mg, 78%); Rf = 0.16 (PE/Et_2O 90/10); ^{1}H NMR (600 MHz, $CDCl_3$) δ 7.80 (d, *J* = 9.1 Hz, 1H, H_7), 7.35 (m, 2H, CH_{Ph}), 7.31 – 6.96 (m, 3H, CH_{Ph}), 6.95 (d, *J* = 2.6 Hz, 1H, H_4), 6.85 (dd, *J* = 9.0, 2.6 Hz, 1H, H_6), 5.99 (ddt, *J* = 17.1, 10.5, 5.4 Hz, 1H, =C*H*), 5.91 (ddt, *J* = 16.1, 10.1, 6.0 Hz, 1H, =C*H*), 5.11 – 5.01 (m, 3H, =CH_2, =C*H*H), 4.95 (dd, *J* = 17.2, 1.6 Hz, 1H, =CH*H*), 4.32 (s, 2H, COCH_2), 3.84 (s, 3H, OCH_3), 3.78 – 3.72 (m, 2H, $CH_{2\ allyl}$), 3.39 (m, 2H, $CH_{2\ allyl}$); ^{13}C{^{1}H} NMR (151 MHz, $CDCl_3$) δ 171.0 (*C*=O), 156.1 (C_{OMe}), 135.9 (C_q), 135.60 (=*C*H), 135.59 (=*C*H), 134.1 (C_q), 131.6 (C_q), 130.4 (C_q), 129.5 (*C*H), 128.8 (*C*H), 127.3 (*C*H), 118.6 (C_q), 116.2 (=CH_2), 116.0 (*C*H), 115.9 (=CH_2), 112.0 (*C*H), 102.2 (*C*H), 55.8 (OCH_3), 45.0 (CH_2), 31.0 (CH_2), 28.4 (CH_2); HRMS (CI) calcd for $C_{23}H_{24}NO_2$ $[M+H]^+$ 346.1807 found 346.1799; FT-IR (ATR) υ = 3077 (CH), 3030 (CH), 3003 (CH), 2926 (CH), 2833 (CH), 1697 (C=O), 1637 (C=C), 1606, 1496, 1476, 1454, 1434, 1360, 1319, 1281, 1233, 1169, 1110, 1076, 1052, 1033 cm^{-1}.

(2,3-Diallyl-1*H*-indol-1-yl)(4-methoxyphenyl)methanone (13e)

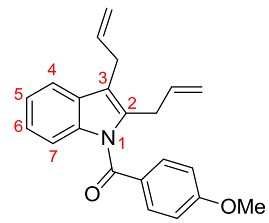

The product was obtained by following the **General procedure E**. After evaporation of the volatiles under vacuum, the crude residue was purified by a filtration through a small pad of SiO_2 using PE/Et_2O (95/5) as eluent. The pure product was obtained as colourless oil (56 mg, 73%); Rf = 0.16 (PE/Et_2O 90/10); ^{1}H NMR (600 MHz, $CDCl_3$) δ 7.72 (d, *J* = 8.8 Hz, 2H, CH_{Ph}), 7.51 (d, *J* = 7.8 Hz, 1H, H_7), 7.14 (t, *J* = 7.5 Hz, 1H, H_6), 7.00 (t, *J* = 8.2 Hz, 1H, H_5), 6.96 (d, *J* = 8.8 Hz, 2H, CH_{Ph}), 6.81 (d, *J* = 8.4 Hz, 1H, H_4), 5.99 (ddt, *J* = 16.2, 10.0, 6.1 Hz, 1H, =C*H*), 5.88 (ddt, *J* = 16.2, 10.3, 5.9 Hz, 1H, =C*H*), 5.18 – 5.04 (m, 1H, =CH_2), 5.00 – 4.89 (m, 1H, =CH_2), 3.90 (s, 3H, OCH_3), 3.72 (d, *J* = 5.9 Hz, 2H, $CH_{2\ allyl}$), 3.51 (d, *J* = 6.1 Hz, 2H, $CH_{2\ allyl}$); ^{13}C{^{1}H} NMR (151 MHz, $CDCl_3$) δ 169.2 (C_q), 163.8 (C_q), 137.0 (C_q), 136.2 (=*C*H), 135.8 (C_q), 135.4 (=*C*H), 132.7 (*C*H), 129.7 (C_q), 127.5 (C_q), 122.8 (*C*H), 121.9 (*C*H), 118.9 (*C*H), 117.1 (C_q), 116.1 (=CH_2), 115.6 (=CH_2), 114.1 (*C*H), 113.9 (*C*H), 55.7 (OCH_3), 29.6 ($CH_{2\ allyl}$), 28.5 ($CH_{2\ allyl}$); HRMS (CI) calcd for $C_{22}H_{21}NO_2$ $[M+H]^+$ 332.16505 found

332.16502; FT-IR (ATR) υ = 3077 (CH), 3005 (CH), 2975 (CH), 2928 (CH), 2839 (CH), 1677 (C=O), 1637 (C=C), 1601, 1575, 1509, 1454, 1419, 1383, 1350, 1313, 1253, 1239, 1171, 1111, 1023 cm^{-1}.

4-Nitrophenyl 2,3-diallyl-5-methyl-1*H*-indole-1-carboxylate (13f)

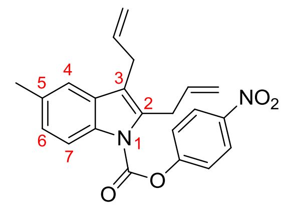

The product was obtained by following the **General procedure E**. After evaporation of the volatiles under vacuum, the crude residue was purified by filtration through a small pad of SiO_2 using PE/Et_2O (100/0 to 90/10) as eluent. The pure product was obtained as a white solid (56 mg, 74%); mp = 62 - 63°C; Rf = 0.21 (PE/Et_2O 90/10); ^{1}H NMR (600 MHz, $CDCl_3$) δ 8.43 – 8.28 (m, 2H, CH_{Ph}), 7.99 (d, *J* = 8.5 Hz, 1H, H_7), 7.59 – 7.39 (m, 2H, CH_{Ph}), 7.31 (s, 1H, H_4), 7.14 (dd, *J* = 8.4, 1.1 Hz, 1H, H_6), 6.12 – 5.83 (m, 2H, =C*H*), 5.21 – 4.96 (m, 4H, =CH_2), 3.81 (d, *J* = 5.5 Hz, 2H, C$H_{2\ allyl}$), 3.45 (dd, *J* = 4.5, 1.5 Hz, 2H, C$H_{2\ allyl}$), 2.46 (s, 3H, CH_3); ^{13}C{^{1}H} NMR (151 MHz, $CDCl_3$) δ 154.9 (C_q), 149.0 (C_q), 145.8 (C_q), 135.6 (=*C*H), 135.5 (=*C*H), 134.6 (C_q), 134.1 (C_q), 133.5 (C_q), 130.6 (C_q), 126.0 (*C*H), 125.6 (*C*H), 122. (*C*H), 119.5 (C_q), 119.1 (*C*H), 116.01 (=*C*H_2), 116.59 (=*C*H_2), 115.6 (*C*H), 30.6 (*C*$H_{2\ allyl}$), 28.4 (*C*$H_{2\ allyl}$), 21.5 (*C*H_3); HRMS (ESI) calcd for $C_{22}H_{21}N_2O_4$ $[M+H]^+$ 377,1501 found 337.1498 ;FT-IR (ATR) υ = 3080 (CH), 2919 (CH), 2858 (CH), 1741 (C=O), 1637 (C=C), 1615, 1592, 1522, 1490, 1473, 1375, 1358, 1342, 1320, 1257, 1215, 1194, 1159, 1110, 1091, 1011 cm^{-1}.

6. (L)-Proline catalysed asymmetric Mannich reaction

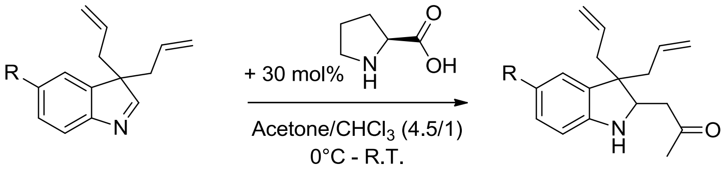

General procedure G: L-proline (30 mol%) was added at 0 °C to a solution of 3,3-diallyl-3*H*-indole (1 eq.) in a mixture of acetone:$CHCl_3$ (4.5:1, C ≈ 0.022 mol/L). The reaction mixture was allowed to warm up slowly to room temperature and stirred for 2 days. Evaporation of the solvent followed by purification by column chromatography on SiO_2 afforded the Mannich product.

General proce.ure H: L-proline (30 mol%) was added at 0 °C to a solution of 3,3-diallyl-3*H*-indole (1 eq.) in a mixture of acetone/DMSO (4:1, C ≈ 0.016 mol/L). The solution was allowed to warm up slowly to room temperature and stirred for 2 days. The reaction mixture was diluted with diethyl ether and washed with $NaHCO_{3sat}$. The product was extracted with Et_2O (3 times) and combined organic layers

were washed with water, brine and dried with $MgSO_4$. After filtration and removal of the solvents under reduce pressure the crude product was purified by column chromatography on SiO_2.

1-(3,3-Diallylindolin-2-yl)propan-2-one (14a)

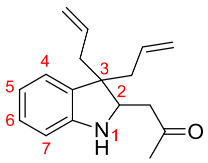

The product was obtained by following the **General procedure G**. After evaporation of the volatiles, the crude mixture was purified by column chromatography on SiO_2 using (CH_2Cl_2/Et_2O 100/0 to 95/5) as eluent. The title compound was obtained as a yellow oil (61 mg, 81%) with 99.3/0.7 er determined by chiral HPLC (Chiralpak Daicel AD // hexane/ *i*PrOH (95/5) // 0.5 mL/min), tr_{e1} = 16.71 min (minor), tr_{e2} = 21.45 min (Major); $[\alpha]_D^{20}$ = + 0.111 (c = 1.01 mg/mL, $CHCl_3$); R_f = 0.50 (CH_2Cl_2/Et2O 9.5/0.5); ^{1}H (600 MHz, $CDCl_3$) δ 7.04 (td, 1H, *J* = 7.4, 1.0 Hz, H_6), 6.97 (d, 1H, *J* = 7.4 Hz, H_4), 6.73 (td, 1H, *J* = 7.4, 1.0 Hz, H_5), 6.63 (d, 1H, *J* = 7.4 Hz, H_7), 5.75 (dtd, 1H, *J* = 17.1, 9.7, 7.3 Hz, =C*H*), 5.68 (dtd, 1H, *J* = 17.1, 9.7, 7.3 Hz, =C*H*), 5.07-5.00 (m, 4H, =CH_2), 4.53 (br s, 1H, N*H*), 4.00 (dd, 1H, *J* = 8.5, 4.6 Hz, H_2), 2.80 (m, 2H, C(O)CH_2), 2.52 (dd, 1H, *J* = 14.1, 7.3 Hz, CHH_{allyl}); 2.38 (dd, 1H, *J* = 14.1, 7.3 Hz, C*H*H_{allyl}), 2.43 (dd, 1H, *J* = 14.1, 7.3 Hz, CHH_{allyl}), 2.10 (dd, 1H, *J* = 14.1, 7.3 Hz, C*H*H_{allyl}), 2.20 (s, 3H, CH_3); $^{13}C\{^1H\}$ (150 MHz, $CDCl_3$) δ 208.6 (*C*=O), 150.0 (C_q), 143.8 (*C*H), 134.4 (*C*H), 133.8 (C_q), 127.8 (*C*H), 124.1 (*C*H), 118.6 (*C*H), 118.2 (=CH_2), 118.0 (=CH_2), 109.9 (*C*H), 62.1 (*C*H), 49.0 (C_q), 44.0 (CH_2), 41.0 (CH_2), 38.5 (CH_2), 30.7 (CH_3); HRMS (CI) calcd. for $C_{17}H_{22}NO$ $[M+H]^+$ 256.1696, found 256.1698; FT-IR (ATR) υ = 3010 (CH), 2929 (CH), 1693 (C=O), 1505, 1253 cm^{-1}.

1-(3,3-Diallyl-5-methoxyindolin-2-yl)propan-2-one (14b)

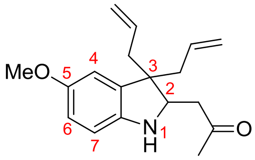

The product was obtained by following the **General procedure G**. After evaporation of the volatiles, the crude mixture was purified by column chromatography on SiO_2 using CH_2Cl_2/Et_2O (100/0 to 95/5) as eluent. The title compound was obtained as a yellow oil (60 mg, 96%) with 98.8/1.2 er determined by chiral HPLC (Chiralpak Daicel AD // hexane/ *i*PrOH (75/25) // 0.5 mL/min), tr_{e1} = 15.12 min (Major), tr_{e2} = 16.70 min (minor); $[\alpha]_D^{20}$ = + 0.100 (c = 1.01 mg/mL, $CHCl_3$); R_f = 0.50 (CH_2Cl_2/Et_2O 9.5/0.5); ^{1}H NMR (600 MHz, $CDCl_3$) δ 6.61 (dd, 1H, *J* = 8.3, 2.4 Hz, H_6), 6.59 (d, 1H, *J* = 2.4 Hz, H_4), 6.56 (d, 1H, *J* = 8.3 Hz, H_7), 5.74 (dtd, 1H, *J* = 16.3, 9.2, 7.3 Hz, =C*H*), 5.67(dtd, 1H, *J* = 16.3, 9.2, 7.3 Hz, =C*H*), 5.06-5.00 (m, 4H, =CH_2), 3.99 (dd, 1H, *J* = 10.5, 2.4 Hz, H_2), 3.74 (s, 3H, OCH_3), 2.81 (m, 2H, C(O)CH_2), 2.51 (dd, 1H, *J* = 14.3, 7.3 Hz, CHH_{allyl}), 2.39 (dd, 1H, *J* = 14.3, 7.3 Hz, CHH_{allyl}), 2.36 (dd, 1H, *J* = 14.3, 7.3 Hz, CHH_{allyl}), 2.12 (dd, 1H, *J* = 14.3, 7.3 Hz, CHH_{allyl}), 2.20 (s, 3H, CH_3); $^{13}C\{^1H\}$

NMR (150 MHz, $CDCl_3$) δ 208.9 (*C*=O), 154.3 (C_q), 143.8 (C_q), 135.5 (C_q), 134.7 (*C*H), 134.3 (*C*H), 118.3 (=CH_2), 118.1 (=CH_2), 112.4 (*C*H), 111.4 (*C*H), 110.1 (*C*H), 62.6 (*C*H), 56.1 (CH_3), 49.3 (C_q), 44.3 (CH_2), 40.9 (CH_2), 38.4 (CH_2), 30.8 (CH_3); HRMS (CI) calcd. for $C_{18}H_{24}NO_2$ $[M+H]^+$ 286.1801, found 286.1795; FT-IR (ATR) υ = 3367 (N-H), 2977 (CH), 1713 (C=O), 1500, 1434, 1220, 1168 cm^{-1}.

1-(3,3-Diallyl-5-chloroindolin-2-yl)propan-2-one (14c)

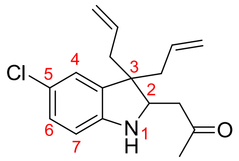

The product was obtained by following the **General procedure H**. The crude mixture was purified by column chromatography on SiO_2 using PE/EtOAc (100/0 to 80/20) as eluent. The title compound was obtained as a yellow oil (28 mg, 64%) with 99.0/1.0 er determined by chiral HPLC (Chiralpak Daicel AD // hexane/ *i*PrOH (75/25) // 0.5 mL/min), tr_{e1} = 11.56 min (minor), tr_{e2} = 13.14 min (Major); $[\alpha]_D^{20}$ = + 0.360 (c = 1.36 mg/mL, $CHCl_3$); R_f = 0.50 (CH_2Cl_2/Et_2O 9.5/0.5); ^1H (600 MHz, $CDCl_3$) δ 6.98 (dd, 1H, *J* = 8.3, 2.3 Hz, H_6), 6.90 (d, 1H, *J* = 2.3 Hz, H_4), 6.53 (d, 1H, *J* = 8.3 Hz, H_7), 5.71 (dtd, 1H, *J* = 17.0, 9.8, 7.0 Hz, =C*H*), 5.65 (dtd, 1H, *J* = 17.0, 9.8, 7.0 Hz, =C*H*), 5.09-5.00 (m, 4H, =CH_2), 4.55 (br s, 1H, N*H*), 4.01 (dd, 1H, *J* = 8.4, 4.8 Hz, H_2), 2.79 (m, 2H, C(O)CH_2), 2.48 (dd, 1H, *J* = 13.8, 7.0 Hz, CHH_{allyl}), 2.39 (dd, 1H, *J* = 13.8, 7.0 Hz, CHH_{allyl}), 2.35 (dd, 1H, *J* = 13.8, 7.0 Hz, CHH_{allyl}); 2.12 (dd, 1H, *J* = 13.8, 7.0 Hz, CHH_{allyl}), 2.20 (s, 3H, CH_3); $^{13}C\{^1H\}$ (150 MHz, $CDCl_3$) δ 208.3 (*C*=O), 148.4 (C_q), 135.9 (C_q), 134.1 (*C*H), 134.0 (*C*H), 127.7 (*C*H), 124.3 (*C*H), 123.2 (C_q), 118.6 (=CH_2), 118.5 (=CH_2), 110.8 (*C*H), 63.1 (*C*H), 49.2 (C_q), 43.8 (CH_2), 40.7 (CH_2), 38.2 (CH_2), 30.7 (CH_3); HRMS (CI) calcd. for $C_{17}H_{21}NO$ $[M+H]^+$ 290.1306, found 290.1304; FT-IR (ATR) υ = 3010 (CH), 2918 (CH), 1713 (C=O), 1479, 1427, 1169 cm^{-1}.

7. Ring closing metathesis reactions of UGI compounds

General procedure I: First generation Grubbs catalyst (15 mol %) to a degassed solution of the corresponding Ugi product (1eq.) in CH_2Cl_2 (C ≈ 0.06 mol/L) at 45°C. The reaction mixture was heated at reflux overnight under argon before evaporation of the solvent under reduced pressure. The crude residue was purified by column chromatography on SiO_2 to yield the desired product.

1'-Benzoyl-*N*-(*tert*-butyl)spiro[cyclopentane-1,3'-indolin]-3-ene-2'-carboxamide (15a)

The product was obtained by following the **General procedure I**. The crude mixture was purified by column chromatography on SiO_2 using (PE/EtOAc 60/40) as eluent. The title compound was obtained as

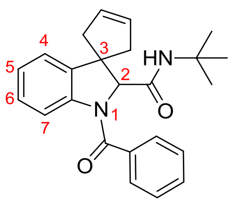

a grey oil (56 mg, 96%); R_f = 0.44 (PE/EtOAc 4/1); ^{1}H NMR (400 MHz, $CDCl_3$, 55°C) δ 7.54 (d, 2H, *J* = 7.4 Hz, C$H_{o\text{-Bz}}$), 7.49 (t, 1H, *J* = 7.4 Hz, *C*$H_{p\text{-Bz}}$), 7.44 (t, 2H, *J* = 7.4 Hz, C$H_{m\text{-Bz}}$), 7.22 (d, 1H, *J* = 7.6 Hz, H_4), 7.13 (br s, 1H, H_7), 7.08 (t, 1H, *J* = 7.6 Hz, H_6), 7.01 (t, 1H, *J* = 7.6 Hz, H_5), 5.91 (dt, 1H, *J* = 5.4, 2.1 Hz, =C*H*CH_2), 5.71 (dt, 1H, *J* = 5.4, 2.1 Hz, =C*H*CH_2), 5.39 (br s, 1H, N*H*), 4.50 (s, 1H, H_2), 2.90 (ddt, 1H, *J* = 17.0, 5.4, 2.1 Hz, C_3C*H*H), 2.79 (ddt, 1H, *J* = 16.2, 5.4, 2.1 Hz, C_3CH*H*), 2.72 (ddt, 1H, *J* = 17.0, 5.4, 2.1 Hz, C_3C*H*H), 2.62 (ddt, 1H, *J* = 16.2, 5.4, 2.1 Hz, C_3CH*H*), 1.27 (s, 9H, C$H_{3\ tBu}$); ^{13}C{^{1}H} NMR (150 MHz, $CDCl_3$, R.T.) δ 169.3 (C_q), 168.3 (C_q), 141.4 (C_q), 140.1 (C_q), 136.2 (C_q), 130.81 (*C*H), 130.76 (*C*H), 128.9 (*C*H), 128.1 (*C*H), 127.8 (*C*H), 127.5 (*C*H), 124.5 (*C*H), 122.3 (*C*H), 116.1 (*C*H), 76.0 (*C*H), 53.8 (C_q), 51.7 (C_q), 50.4 (CH_2), 39.9 (CH_2), 28.6 ($CH_{3\ tBu}$); HRMS (CI) calcd. for $C_{24}H_{27}N_2O_2$ $[M+H]^+$ 375.2067, found 375.2057; FT-IR (ATR) υ = 3339 (N-H), 2967 (CH), 2925 (CH), 1679 (C=O), 1630 (C=O), 1479, 1390 cm^{-1}.

1'-Benzoyl-*N*-cyclohexylspiro[cyclopentane-1,3'-indolin]-3-ene-2'-carboxamide (15b)

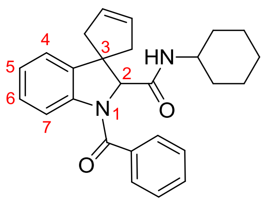

The product was obtained by following the **General procedure I**. The crude mixture was purified by column chromatography on SiO_2 using (PE/EtOAc 60/40) as eluent. The title compound was obtained as a grey oil (60 mg, 98%); R_f = 0.25 (PE/EtOAC 4/1); ^{1}H NMR (400 MHz, $CDCl_3$, 55°C) δ 7.53 (d, 2H, *J* = 7.6 Hz, C$H_{o\text{-Bz}}$), 7.48 (t, 1H, *J* = 6.9 Hz, C$H_{p\text{-Bz}}$), 7.44 (t, 2H, *J* = 7.2 Hz, C$H_{m\text{-Bz}}$), 7.21 (d, 1H, *J* = 7.3 Hz, H_4), 7.15 (br s, 1H, H_7), 7.08 (t, 1H, *J* = 7.1 Hz, H_6), 7.01 (t, 1H, *J* = 7.4 Hz, H_5), 5.89 (m, 1H, =C*H*CH_2), 5.69 (m, 1H, =C*H*CH_2), 5.52 (d, 1H, *J* = 6.5 Hz, N*H*), 4.64 (s, 1H, H_2), 3.76 (m, 1H, NCH_{Cy}), 2.87 (dd, 1H, *J* = 17.1, 1.5 Hz, C_3C*H*), 2.81 (d, 1H, *J* = 16.4 Hz, C_3C*H*), 2.68 (d, 1H, *J* = 16.9 Hz, C_3C*H*), 2.62 (d, 1H, *J* = 15.9 Hz, C_3C*H*), 1.88 (d, 1H, *J* = 10.4 Hz, C*H*H_{Cy}), 1.77 (m, 1H, CHH_{Cy}), 1.64 (m, 1H, C*H*H_{Cy}), 1.56 (br d, 1H, *J*= 12.6 Hz, CHH_{Cy}), 1.39 (m, 3H, C*H*H_{Cy}) 1.16 (q, 2H, CHH_{Cy}), 1.04 (q, 1H, *J* = 10.9 Hz, C*H*H_{Cy}); ^{13}C{^{1}H} NMR (150 MHz, $CDCl_3$, 55°C) δ 169.5 (C_q), 168.1 (C_q), 141.2 (C_q), 140.3 (C_q), 136.0 (C_q), 130.7 (*C*H), 130.7 (*C*H), 128.8 (*C*H), 127.9 (*C*H), 127.4 (*C*H), 124.7 (*C*H), 122.3 (*C*H), 75.7 (*C*H), 53.8 (C_q), 50.2 (CH_2), 48.2 (*C*H), 40.2 (CH_2), 32.9 (CH_2), 32.8 (CH_2), 25.5 (CH_2), 24.7 (CH_2), 24.6 (CH_2); C_7H was not observed by ^{13}C{^{1}H} NMR; HRMS (CI) calcd. for $C_{26}H_{28}N_2O_2$ $[M]^+$ 400.2145, found 400.2146; FT-IR (ATR) υ = 3314 (N-H), 2930 (CH), 2854 (CH), 1634 (C=O), 1479, 1374 cm^{-1}.

1'-Benzoyl-*N*-(*tert*-butyl)-5',6'-dimethoxyspiro[cyclopentane-1,3'-indolin]-3-ene-2'-carboxamide (15c)

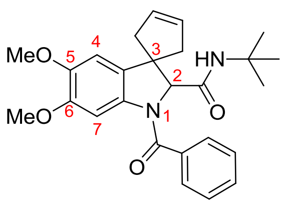

The product was obtained by following the **General procedure I**. The crude mixture was purified by column chromatography on SiO_2 using (PE/EtOAc 70/30 to 60/40) as eluent. The title compound was obtained as a grey oil (50 mg, 89%); R_f = 0.15 (PE/EtOAc 4/1); ^{1}H NMR (400 MHz, $CDCl_3$, 55°C) δ 7.77-7.45 (m, 5H, CH_{Bz}), 6.76 (s, 1H, H_4), 5.91 (td, 1H, J = 5.7, 2.1 Hz, =C$H$$CH_2$), 5.72 (td, 1H, J = 5.7, 2.1 Hz, =C$H$$CH_2$), 5.36 (br s, 1H, N$H$), 4.51 (s, 1H, H_2), 3.84 (s, 3H, OCH_3), 3.69 (s, 3H, OCH_3), 2.87 (ddt, 1H, J = 17.1, 5.7, 2.1 Hz, C_3CHH), 2.76 (ddt, 1H, J = 16.3, 5.7, 2.1 Hz, C_3CHH), 2.67 (ddt, 1H, J = 17.1, 5.7, 2.1 Hz, C_3CHH), 2.60 (ddt, 1H, J = 16.3, 5.7, 2.1 Hz, C_3CHH), 1.31 (s, 9H, C$H_{3\ tBu}$), H_7 was not observed in ^{1}H NMR; $^{13}C\{^1H\}$ NMR (150 MHz, $CDCl_3$, R.T.) δ 169.3 (C_q), 168.3 (C_q), 148.8 (C_q), 146.3 (C_q), 136.4 (C_q), 134.6 (C_q), 130.7 (CH), 131.1 (C_q), 130.5 (CH), 128.5 (2 × CH), 128.4 (CH), 127.4 (2 × CH), 105.6 (CH), 66.0 (CH), 56.4 ($C$$H_3$), 55.9 ($C$$H_3$), 54.1 ($C_q$), 51.8 ($C_q$), 50.1 ($C$$H_2$), 39.8 ($C$$H_2$), 28.7 ($C$H3 $_{tBu}$); C_7H was not observed in $^{13}C\{^1H\}$ NMR; HRMS (CI) calcd. for $C_{26}H_{30}N_2O_4$ $[M]^+$ 434.2205, found 434.2202; FT-IR (ATR) υ = 3342 (N-H), 3061 (CH), 2965 (CH), 1686 (C=O), 1634 (C=O), 1500, 1447, 1398, 1215 cm^{-1}.

***tert*-Butyl (2-(2'-(*tert*-butylcarbamoyl)-5'-chlorospiro[cyclopentane-1,3'-indolin]-3-en-1'-yl)-2-oxoethyl)carbamate (15j)**

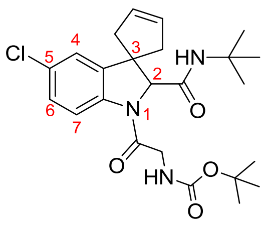

The product was obtained by following the **General procedure I**. The crude mixture was purified by column chromatography on SiO_2 using (PE/EtOAc 100/0 to 60/40) as eluent. The title compound was obtained as a pale yellow oil (78 mg, 77%); R_f = 0.35 (PE/EtOAc 3/2); ^{1}H NMR (400 MHz, $CDCl_3$, 55°C) δ 7.94 (brs, 1H, H_7), 7.21 (dd, 1H, J = 8.8, 2.0 Hz, H_6), 7.19 (s, 1H, H_4), 5.93 (m, 1H, =C$H$$CH_2$), 5.73 (m, 1H,=C$H$$CH_2$), 5.54 (br s, 1H, N$H$), 5.41 (br s, 1H, N$H$), 4.53 (s, 1H, H_2), 4.20 (dd, 1H, J = 16.7, 4.9 Hz, C(O)CHH), 3.91 (br s, 1H, C(O)CHH), 2.89 (d, 1H, J = 16.6 Hz, C_3CHH), 2.74 (d, 1H, J = 17.6 Hz, C_3CHH), 2.69 (d, 1H, J = 17.6 Hz, C_3CHH), 2.56 (d, 1H, J = 16.6 Hz, C_3CHH), 1.48 (s, 9H, C$H_{3\ tBu}$), 1.33 (s, 9H, C$H_{3\ tBu}$); $^{13}C\{^1H\ \}$ NMR (150 MHz, $CDCl_3$, R.T.) δ 167.4 (C_q), 155.9 ($C_{q),}$ 140.5 (C_q), 140.0 (C_q), 138.6 (C_q), 130.7 (CH), 129.9 (C_q), 128.4 (CH), 128.2 (CH), 122.5 (CH), 117.6 (CH), 80.2 (C_q), 73.8 (CH), 55.0 (C_q), 52.2 (C_q), 51.0 ($C$$H_2$), 43.8 ($C$$H_2$), 40.1 ($C$$H_2$), 28.6 ($C$$H_{3\ tBu}$), 28.4 ($C$$H_{3\ tBu}$); HRMS (CI) $C_{24}H_{33}ClN_3O_4$ $[M+H]^+$ 462.2154, found 462.2150; FT-IR (ATR) υ = 3450 (N-H), 3423 (N-H), 2957, 1662 (C=O), 1476, 1366, 1253, 821 cm^{-1}.

***tert*-Butyl(2-(2'-(*tert*-butylcarbamoyl)spiro[cyclopent[3]ene-1,3'-indolin]-1'-yl)-2-oxoethyl)carbamate (15k)**

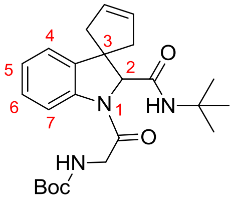

The product was obtained according to the **General procedure I**. After 24 hours at 50°C, purification of the crude residue by column chromatography on silica using PE/EtOAc (100/0 to 70/30) as eluent give cyclised compound as a white solid (50 mg, 88%), NMR at R.T. showed a mixture of rotamers; Rf = 0.27 (PE/EtOAc 90/10); ^{1}H NMR (400 MHz, $CDCl_3$, 55°C) δ 7.91 (s, H_7), 7.26 (dd, J = 13.2, 5.3 Hz, 2H, H_6, H_7), 7.07 (m, 1H, H_4), 5.93 (dt, J = 5.9, 2.1 Hz, 1H, =C*H*CH_2), 5.72 (dt, J = 6.0, 2.1 Hz, 1H, =C*H*CH_2), 5.43 (s, 2H, NH_{Boc}, NH_{tBu}), 4.53 (s, 1H, H_2), 4.23 (dd, J = 17.1, 4.9 Hz, 1H, NHC*H*H), 3.99 (br s, 1H, NHCH*H*), 2.99 – 2.80 (m, 1H, C_3CH*H*), 2.81 – 2.61 (m, 2H, C_3CH*H*), 2.67 – 2.30 (m, 1H, C_3C*H*H), 1.49 (s, 9H, C$H_{3\ tBu}$), 1.31 (s, 9H, C$H_{3\ tBu}$); ^{13}C{1H} NMR (151 MHz, $CDCl_3$, R.T.) δ 167.8 (br, *C*=O), 167.4 (*C*=O), 155.9 (*C*=O), 141.2 (C_q), 138.4 (C_q), 130.7 (*C*H), 128.5 (*C*H), 128.2 (*C*H), 125.1 (*C*H), 122.1 (*C*H), 116.9 (*C*H), 113.9 (C_q), 80.0 (C_q), 74.4 (*C*H), 73.9 (*C*H), 60.5 (CH_2), 55.1 (C_q), 52.9 (C_q), 52.0 (C_q), 51.1 (CH_2), 46.5 (CH_2), 44.8 (CH_2), 40.5 (CH_2), 40.1 (CH_2), 28.6 ($CH_{3\ tBu}$), 28.5 ($CH_{3\ tBu}$); HRMS (CI) calcd for $C_{24}H_{33}N_3O_4$ $[M+H]^+$ 428.2549 found 428.2551; FT-IR (ATR) υ = 3326 (NH), 2965 (CH), 2927 (CH), 2870 (CH), 1665 (C=O), 1596, 1529, 1479, 1454, 1413, 1392, 1365, 1340, 1250, 1227, 1163, 1110, 1083, 1051;1027, 973; 948, 931, 864, 750, 673 cm^{-1}.

***N*-(*tert*-butyl)-1'-((R)-2-hydroxy-2-phenylacetyl)spiro[cyclopent[3]ene-1,3'-indoline]-2'-carboxamide (15n)**

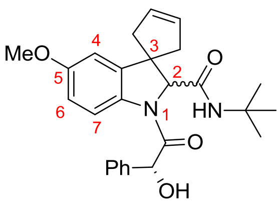

The product was obtained according to the **General procedure I**. Diastereoisomer 1 of compound **8m** was used as the starting material. After 24 hours at 50 °C, the crude residue was purified by column chromatography on silica (PE/Et_2O 100/0 to 50/50) give cyclised compound as a pale yellow solid (18 mg, 38%); mp = 63°C; Rf = 0.25 (PE/EtOAc 9/1); ^{1}H NMR (400 MHz, $CDCl_3$, R.T.) δ 8.21 (d, J = 8.8 Hz, 1H, H_7), 7.44 – 7.30 (m, 5H, CH_{Ph}), 6.81 (dd, J = 8.8, 2.6 Hz, 1H, H_6), 6.71 (d, J = 2.6 Hz, 1H, H_4), 5.77 (dt, J = 5.9, 2.1 Hz, 1H,=C*H*CH_2), 5.39 (dt, J = 5.9, 2.2 Hz, 1H, =C*H*CH_2), 5.18 (s, 1H, N*H*), 5.00 (d, J = 6.1 Hz, 1H, C*H*OH), 4.52 (d, J = 6.5 Hz, 1H, O*H*), 4.17 (s, 1H, H_2), 3.78 (s, 3H, OCH_3), 2.72 (dd, J = 10.6, 8.5 Hz, 1H, C_3C*H*H), 2.53 (d, J = 16.9 Hz, 1H, C_3CH*H*), 2.08 (dd, J = 10.1, 8.2 Hz, 1H, C_3C*H*H), 1.79 – 1.63 (m, 1H, C_3CH*H*), 1.25 (m, 9H, C$H_{3\ tBu}$); ^{13}C{^{1}H} NMR (151 MHz, $CDCl_3$, R.T.) δ 170.6 (C_q), 167.9 (C_q), 157.9 (C_q), 140.7 (C_q), 138.5 (C_q), 134.0 (C_q), 130.0 (*C*H), 129.4 (*C*H), 129.2 (*C*H), 127.9 (*C*H), 127.8 (*C*H), 118.0 (*C*H), 112.8 (*C*H), 108.8 (*C*H), 73.3 (*C*H), 73.0 (*C*H), 55.8 (*C*H), 54.5 (C_q), 51.9 (C_q), 50.2 (CH_2), 39.1 (CH_2), 28.6 ($CH_{3\ tBu}$); HRMS (EI) calcd for $C_{26}H_{30}N_2O_4$ $[M]^+$ 434.2199 found 434.2194; FT-IR (ATR) υ = 3412 (OH), 3335 (NH), 3059 (CH), 2958 (CH), 2919 (CH), 285 (CH), 1650 (C=O), 1485, 1454, 1365, 1330, 1267, 1228, 1181, 1080, 1063, 976, 866, 811, 761, 726, 670, 666, 469, 448 cm^{-1}.

8. Ring closing metathesis reactions on substituted 3,3-diallyl-2-hydroxyindolines

General procedure J: The corresponding 3,3-diallyl-2-hydroxyindoline compound was added to a refluxing solution of first generation Grubbs catalyst (15 mol %) in CH_2Cl_2 (C ≈ 0.04 mol/L) under argon. The reaction was heated at reflux for 24 hrs under argon before evaporation of the volatiles under vacuum. The crude material was purified by column chromatography on SiO_2 to yield the desired product.

Methyl 2'-hydroxyspiro[cyclopentane-1,3'-indolin]-3-ene-1'-carboxylate (16a)

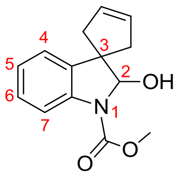

The product was obtained by following the **General procedure J**. The crude mixture was purified by column chromatography on SiO_2 using (PE/EtOAc 100/0 to 80/20) as eluent. The title compound was obtained as a dark green oil (59 mg, 83%); R_f = 0.20 (PE/EtOAc 4/1); ^{1}H NMR (400 MHz, $CDCl_3$, 55°C) δ 7.64 (br s, 1H, H_7), 7.28-7.20 (m, 2H, H_4, H_5), 7.02 (t, 1H, J = 7.1 Hz, H_6), 5.90 (s, 1H, =C*H*CH$_2$), 5.71 (s, 1H, =C*H*CH$_2$), 5.59 (s, 1H, H_6), 3.94 (s, 3H, OCH_3), 3.26 (dd, 1H, J = 16.9 Hz, C$_3$C*H*H), 2.66 (t, 2H, J = 16.9 Hz, C$_3$CH_2), 2.41 (d, 1H, J = 16.9 Hz, C$_3$C*H*H); ^{13}C{^{1}H} NMR (100 MHz, $CDCl_3$, 60°C) δ 139.9 (C_q), 138.1 (C_q), 130.5 (*C*H), 127.8 (*C*H), 127.5 (*C*H), 123.4 (*C*H), 122.2 (*C*H), 114.5 (*C*H), 91.3 (*C*H), 55.0 (C_q), 52.7 (CH_3), 47.7 (CH_2), 37.3 (CH_2); *C*=O was not observed by ^{13}C{^{1}H} NMR at 60°C; HRMS (CI) calcd. for $C_{14}H_{14}NO_2$ [M-OH]$^+$ 228.1119, found 228.1118; FT-IR (ATR) υ = 3020 (CH), 2938 (CH), 1687 (C=O), 1481, 1381, 1135, 690 cm^{-1}.

Methyl 2'-hydroxy-5'-methoxyspiro[cyclopentane-1,3'-indolin]-3-ene-1'-carboxylate (16b)

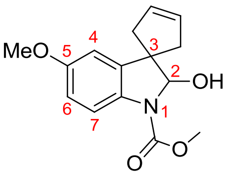

The product was obtained by following the **General procedure J**. The crude mixture was purified by column chromatography on SiO_2 using (PE/EtOAc 100/0 to 70/30) as eluent. The title compound was obtained as a dark green oil (59 mg, 74%); R_f = 0.29 (PE/EtOAc 7/3); ^{1}H NMR (400 MHz, $CDCl_3$, 60°C) δ 7.53 (br s, 1H, H_7), 6.81 (d, 1H, J = 2.6 Hz, H_4), 6.75 (dd, 1H, J = 8.5, 2.6 Hz, H_6), 5.90 (dtd, 1H, J = 6.4, 4.5, 2.3 Hz, =C*H*CH$_2$), 5.70 (dtd, 1H, J = 6.4, 4.5, 2.3 Hz, =C*H*CH$_2$), 5.57 (br s, 1H, H_2), 3.91 (s, 3H, C(O)OCH_3), 3.79 (s, 3H, OCH_3), 3.24 (d, 1H, J = 17.3 Hz, C$_3$C*H*H), 2.67 (d, 1H, J = 16.5 Hz, C$_3$CH*H*), 2.61 (d, 1H, J = 17.3 Hz, C$_3$C*H*H), 2.40 (d, 1H, J = 16.5 Hz, C$_3$C*H*H); ^{13}C{^{1}H} NMR (150 MHz, $CDCl_3$, 60°C) δ 156.6 (C_q), 140.0 (C_q), 132.7 (C_q), 130.4 (*C*H), 127.5 (*C*H), 125.8 (C_q), 115.1 (*C*H), 112.7 (*C*H), 108.9 (*C*H), 91.5 (*C*H), 55.7 (CH_3), 54.6 (C_q), 52.6 (CH_3), 47.5 (CH_2), 37.3 (CH_2); HRMS (CI) calcd. for

$C_{15}H_{16}NO_3$ [M-OH]$^+$ 258.1125, found 258.1127; FT - IR (ATR) υ = 3075 (CH), 2890 (CH), 1692 (C=O), 1490, 1272, 1135, 1032, 694 cm^{-1}.

9. Preparation of dihydro-1*H*-carbazole by ring closing metathesis

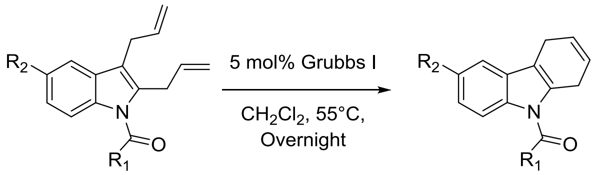

General procedure H: To a dried and degassed solution of 2,3-diallyl-1*H*-indole in CH_2Cl_2 (C ≈ 0.015 mol/L) was added the Grubbs catalyst (5 mol%). The mixture was heated overnight at 55°C before evaporation of the volatiles under reduced pressure. The residue obtained was purified by column chromatography on SiO_2.

1-(6-Methoxy-1*H*-carbazol-9(4*H*)-yl)-2-phenylethanone (18d)

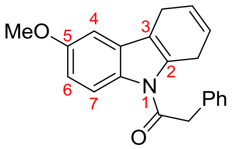

The product was obtained following the **General procedure H**. The crude residue was purified by column chromatography on SiO_2 using PEth/Et_2O (100/0 to 90/10) as eluent to afford the dihydrocarbazole compound as a colourless sticky foam (13 mg, 72%). Rf = 0.29 (PEth/Et_2O 2/1); ^{1}H NMR (600 MHz, $CDCl_3$) δ 7.96 (t, *J* = 8.7 Hz, 1H, H_7), 7.44 – 7.34 (m, 2H, CH_{Ph}), 7.35 – 7.22 (m, 3H, CH_{Ph}), 6.95 – 6.80 (m, 2H, H_4, H_6), 5.90 (dt, *J* = 47.5, 23.8 Hz, 2H, =C*H*), 4.32 (s, 2H, COCH_2), 3.88 (s, 3H; OCH_3), 3.76 (dd, *J* = 18.6, 11.0 Hz, 2H, =CHCH_2), 3.45 – 3.23 (m, 2H, =CHCH_2); ^{13}C{^{1}H} NMR (151 MHz, $CDCl_3$) δ = 170.6 (*C*=O), 156.3 (C_{OMe}), 133.9 (C_q), 133.6 (C_q), 131.2 (C_q), 130.4 (C_q), 129.7 (*C*H), 128.9 (*C*H), 127.4 (*C*H), 124.0 (*C*H), 123.3 (*C*H), 116.5 (*C*H), 115.4 (C_q), 111.9 (*C*H), 101.5 (*C*H), 55.8 (CH_3), 44.9 (CH_2), 28.5 (CH_2), 23.3 (CH_2); HRMS (CI) calcd for $C_{21}H_{20}NO_2$ [M+H]$^+$ 318.1494 found 318.1490; FT-IR (ATR) υ = 3030 (CH), 2928 (CH), 2829 (CH), 1690 (C=O), 1667 (C=O), 1609, 1464, 1433, 1400, 1362, 1316, 1283, 1271, 1206, 1167, 1126, 1091, 1031 cm^{-1}.

10. References

1. Y. Zhang, D. Stephens, G. Hernandez, R. Mendoza, and O. V Larionov, *Chem. Eur. J.*, 2012, **18**, 16612.

2. T. D. Montgomery, Y. Zhu, N. Kagawa, and V. H. Rawal, *Org. Lett.*, 2013, **15**, 1140.

3. P. Agarwal, J. van der Weijden, E. M. Sletten, D. Rabuka, and C. R. Bertozzi, *Proc. Natl. Acad. Sci. USA*, 2013, **110**, 46.

4. S. L. Bender, D. Bhumralkar, M. R. Collins, S. J. Cripps, J. G. Deal, L. Jia, M. D. Nambu, C. L. Palmer, Z. Peng, M. D. Varney, US2002/103203 A1, 2002.

11. Spectra

Synthesis of substituted 3,3-diallyl-3H-indole

3,3-Diallyl-3*H*-indole (3a)

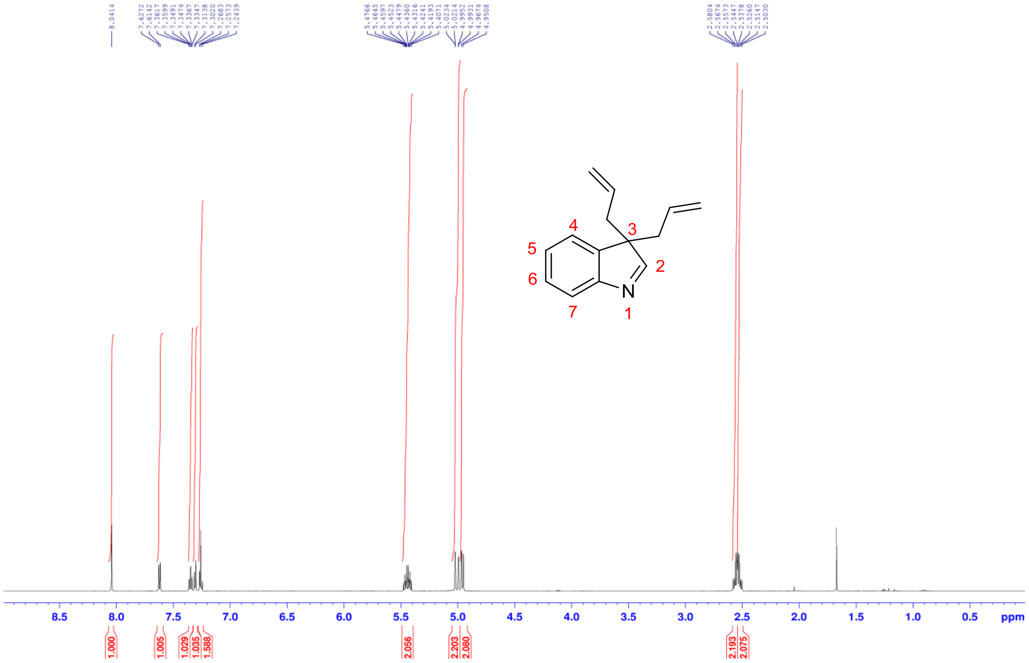

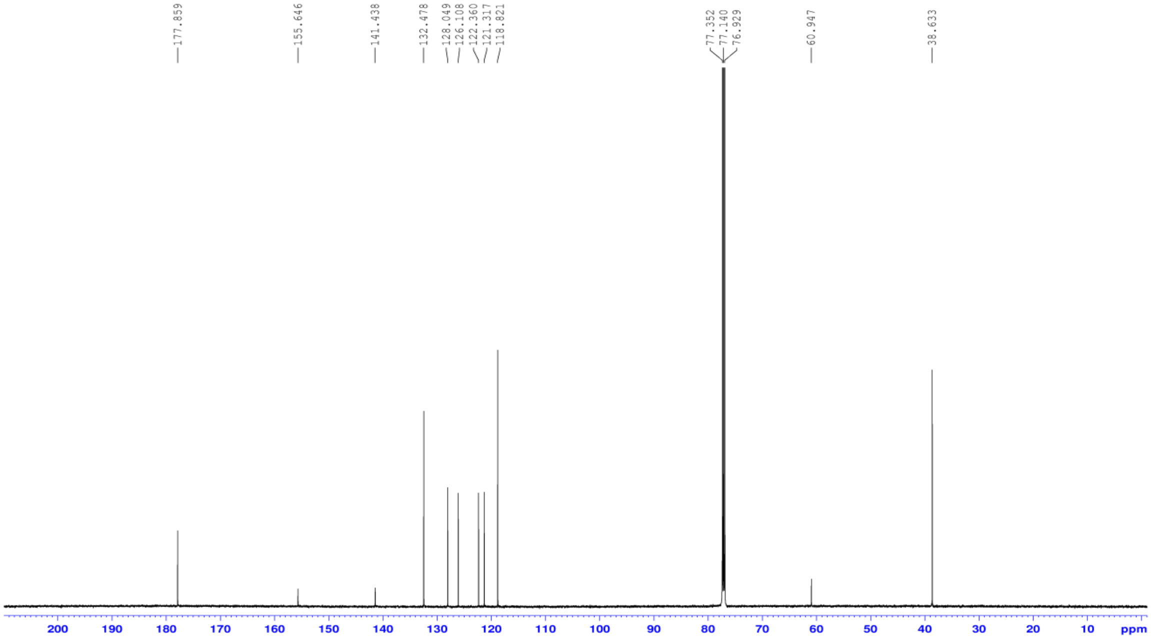

3,3-Diallyl-5,6-dimethoxy-3*H*-indole (3b)

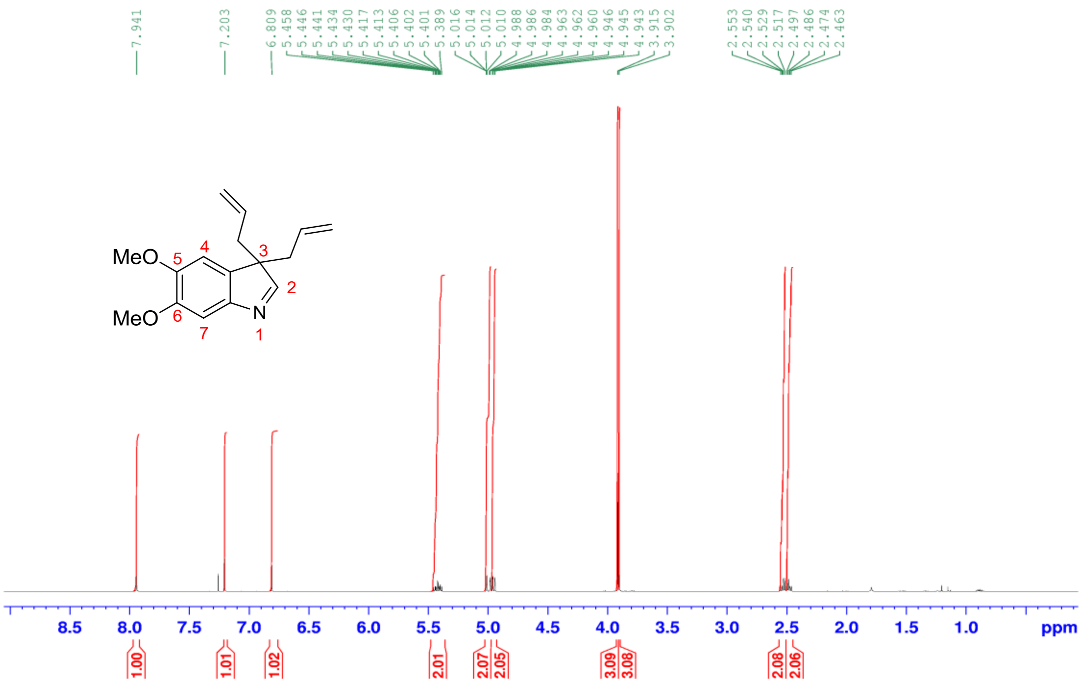

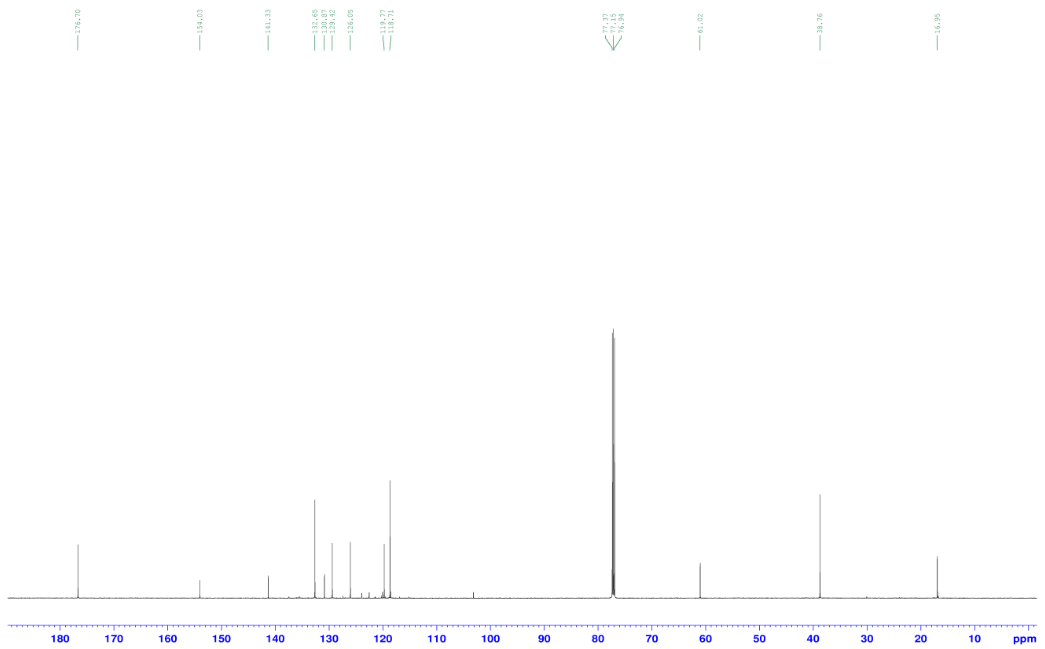

3, 3-Diallyl-5-methyl-3*H*-indole (3c)

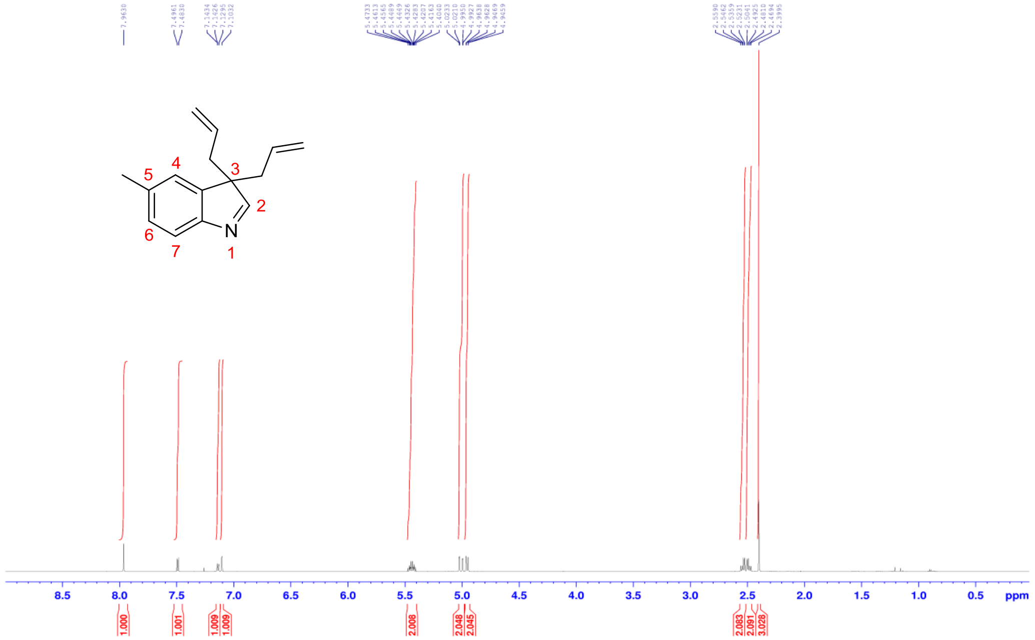

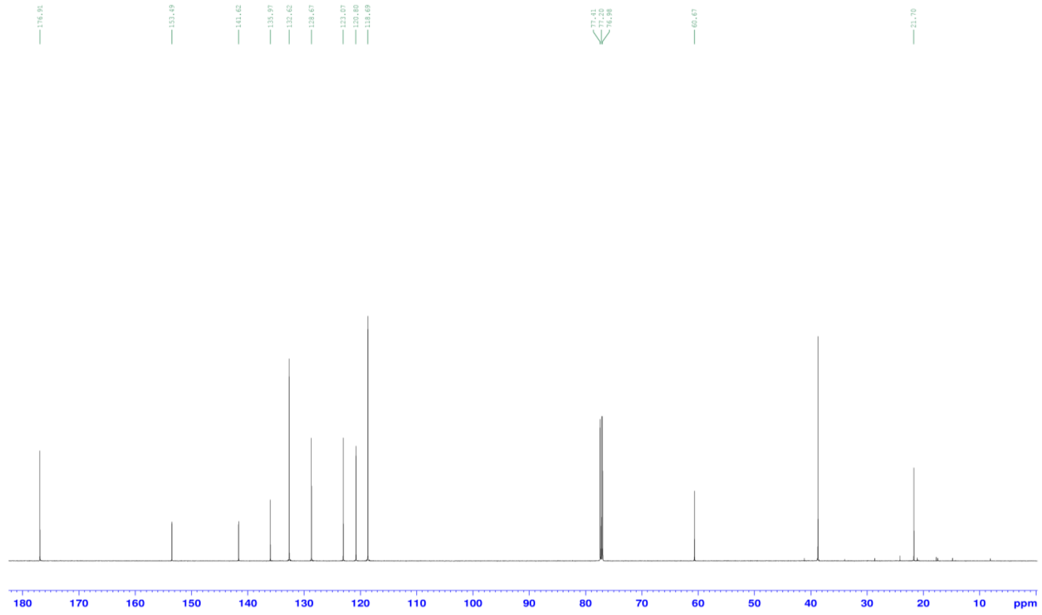

3,3-Diallyl-5-methoxy-3*H*-indole (3d)

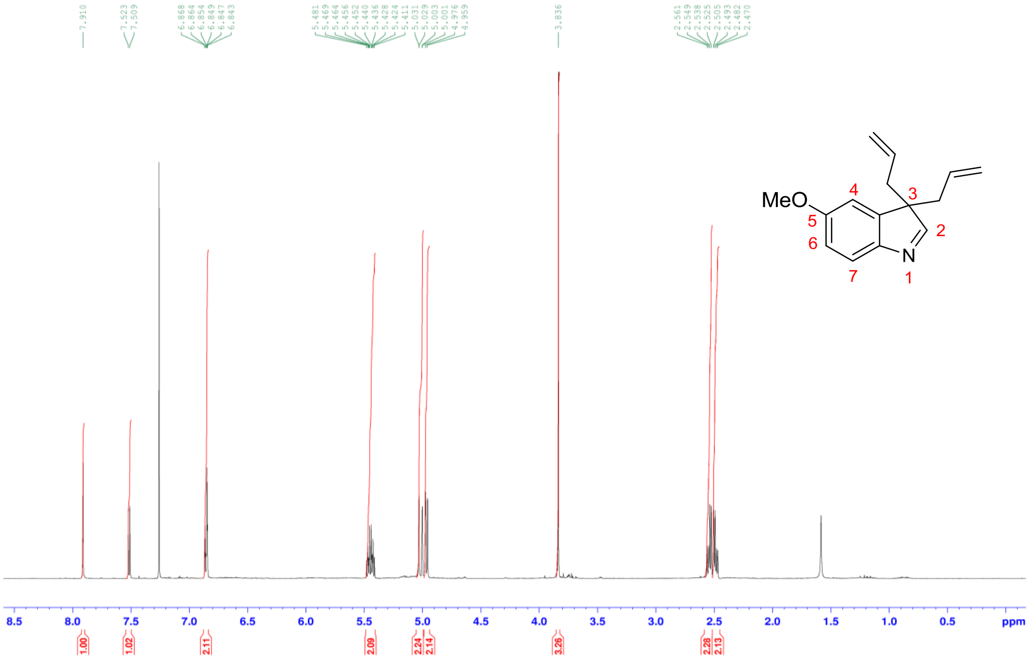

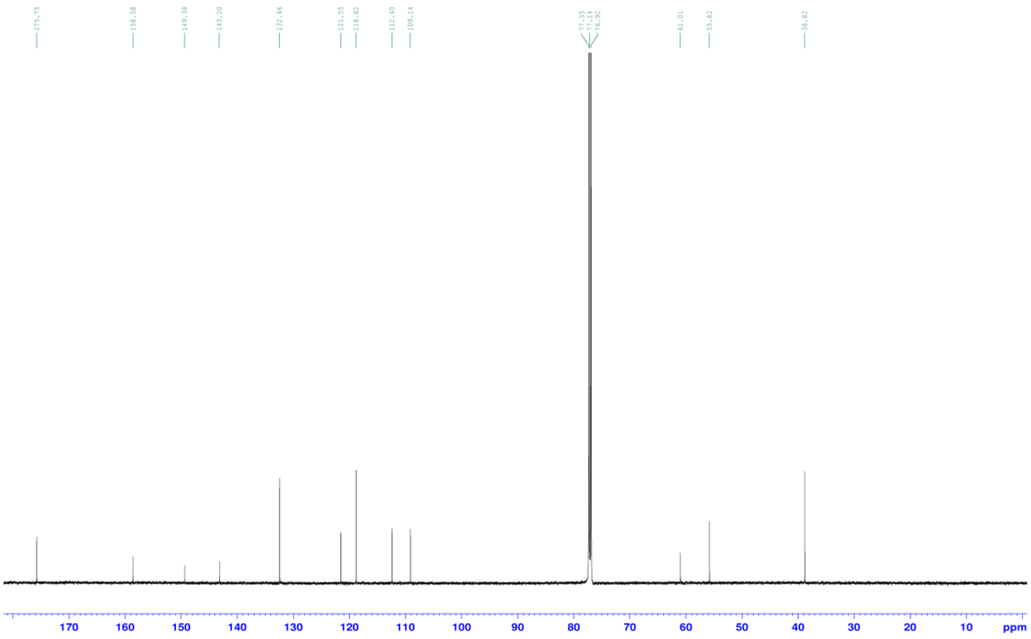

3,3-Diallyl-4-methyl-3*H*-indole (3e)

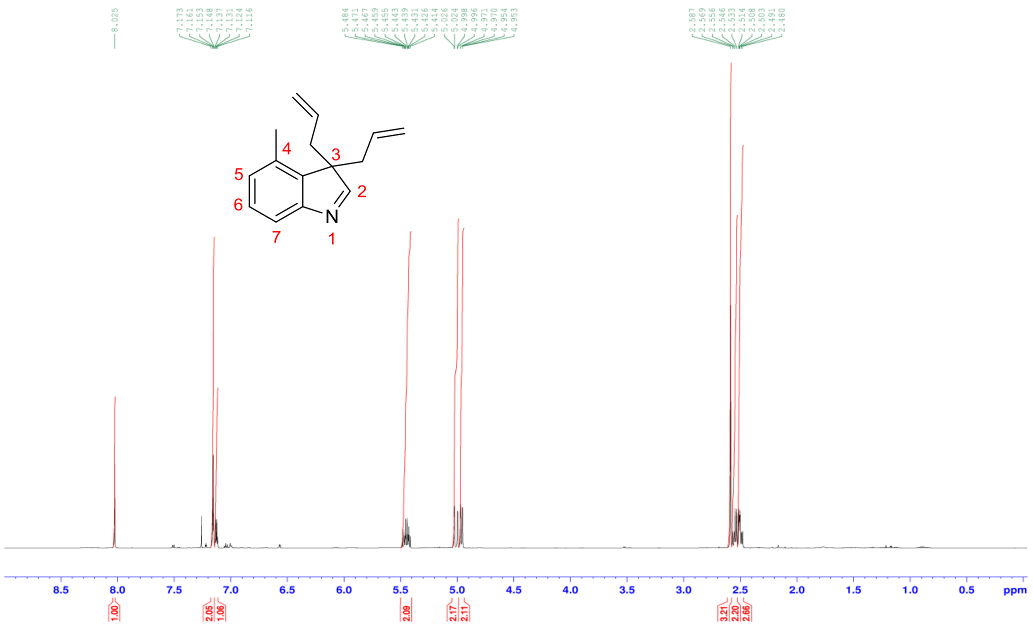

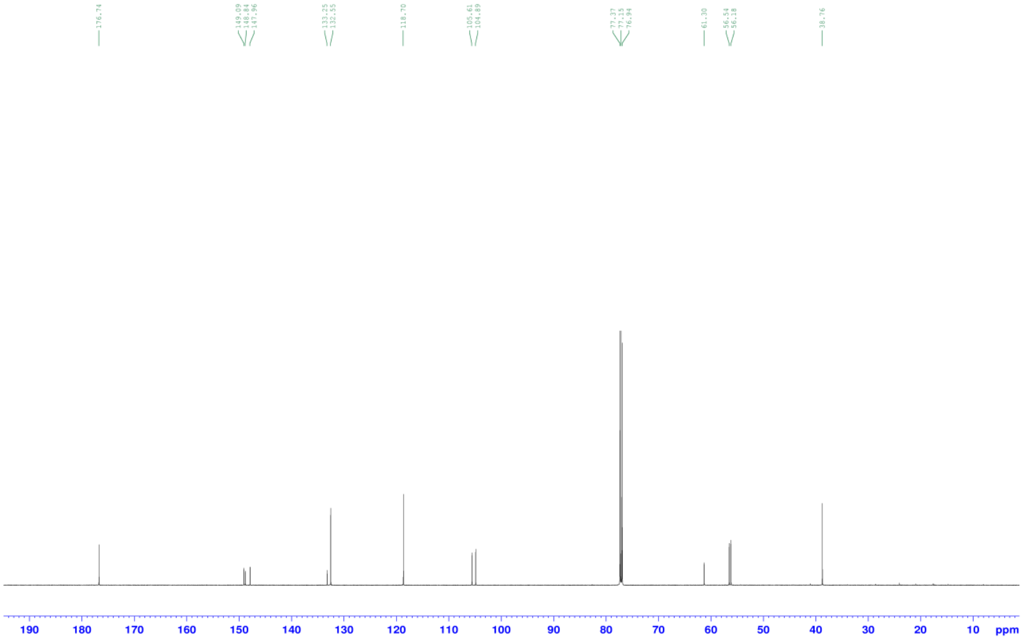

3,3-Diallyl-5-benzyloxy-3*H*-indole (3f)

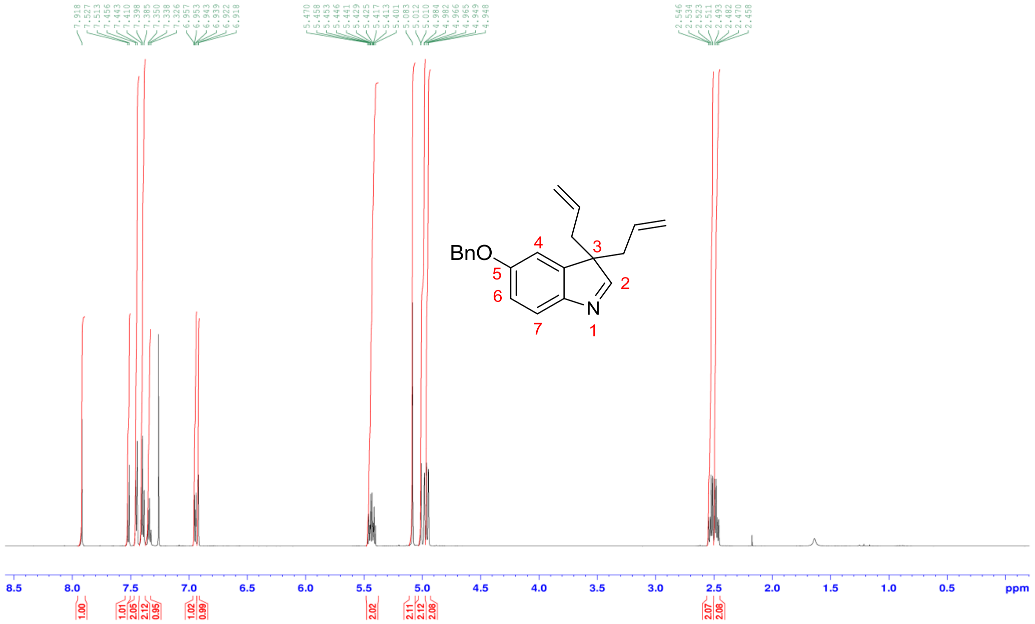

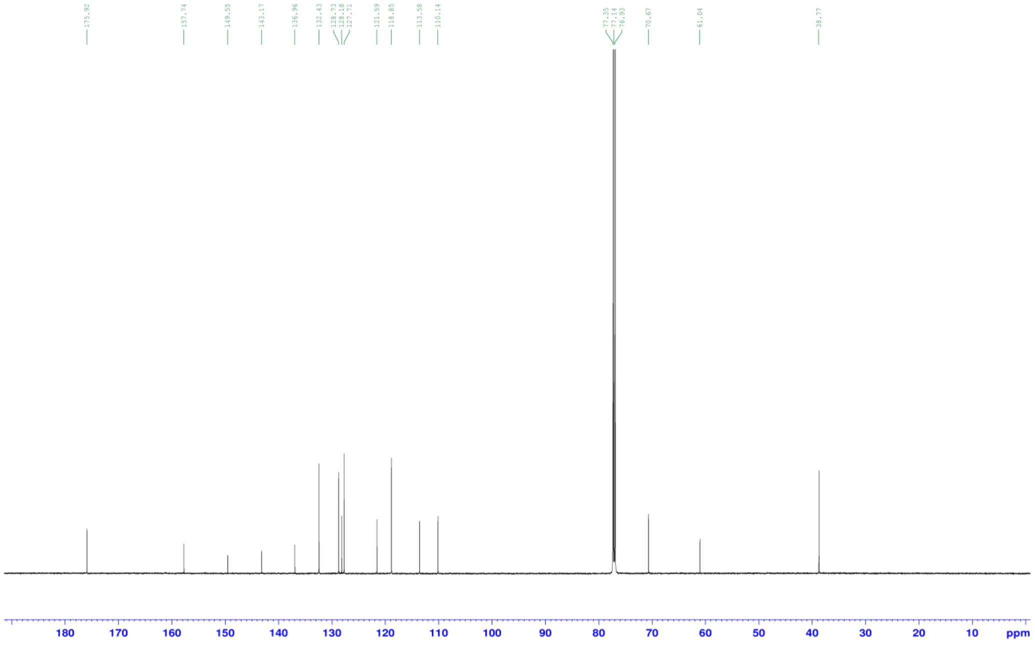

3,3-Diallyl-3,6,7,8-tetrahydrocyclopenta-3*H*-indole (3g)

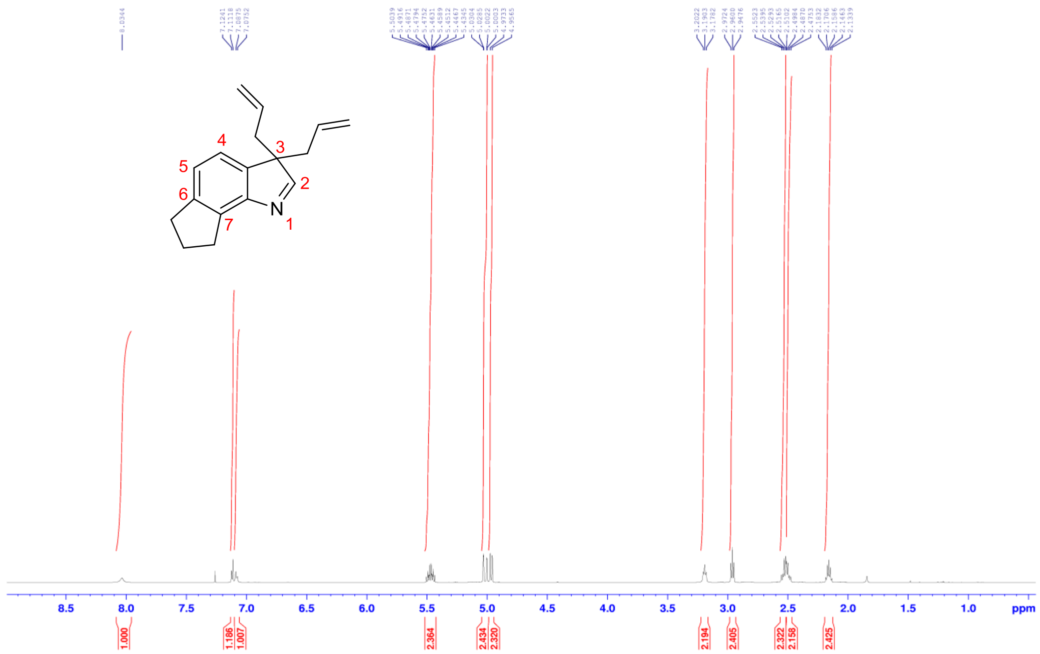

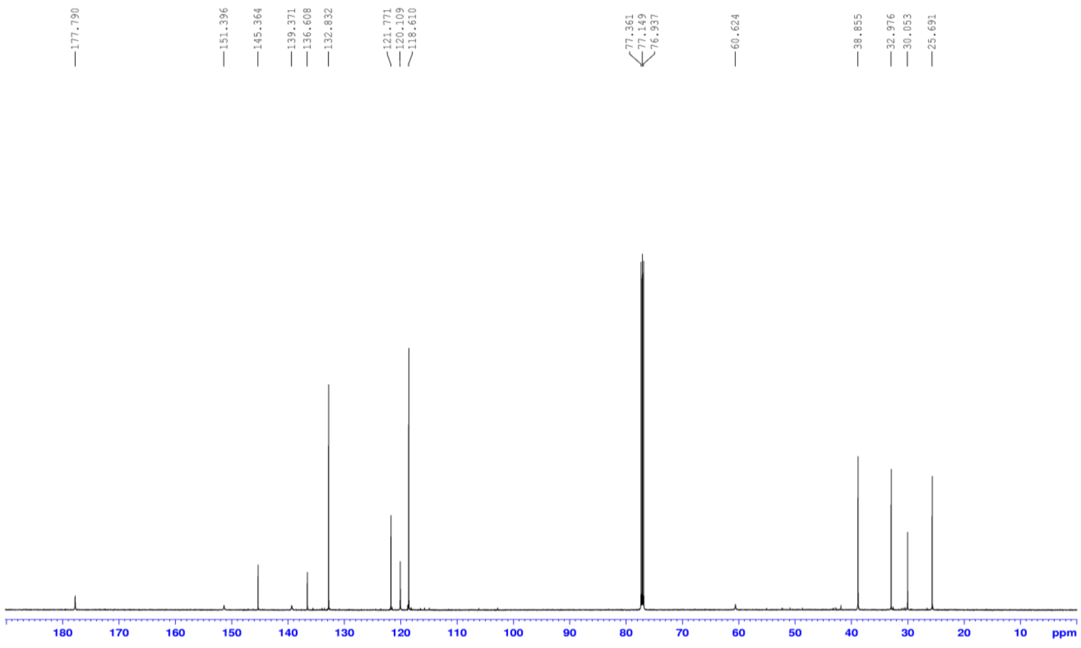

3,3-Diallyl-4-bromo-3*H*-indole (3h)

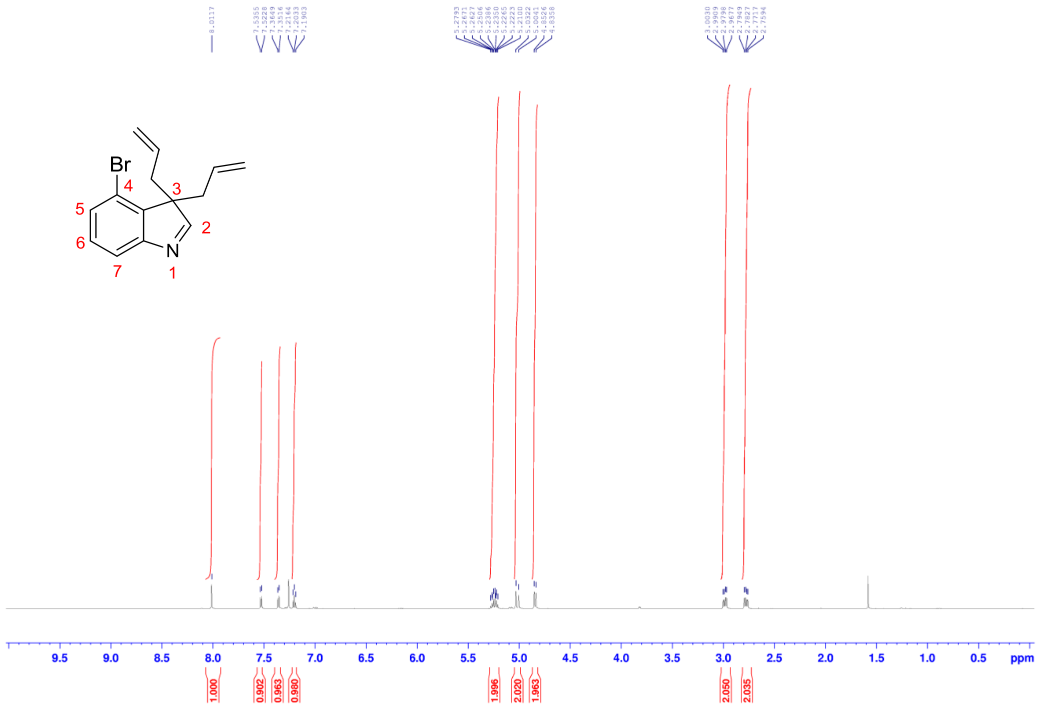

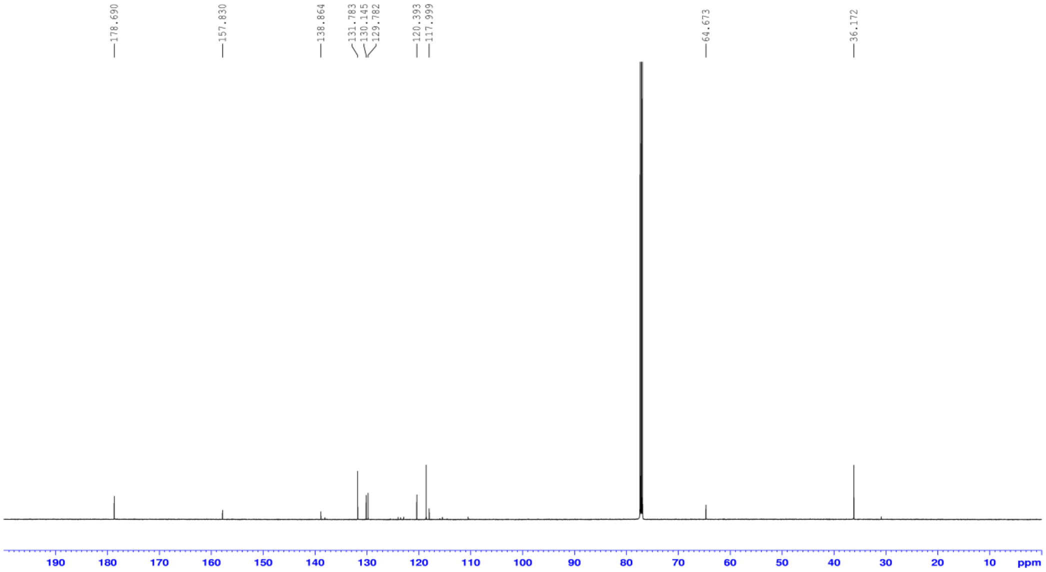

3,3-Diallyl-5-bromo-3*H*-indole (3i)

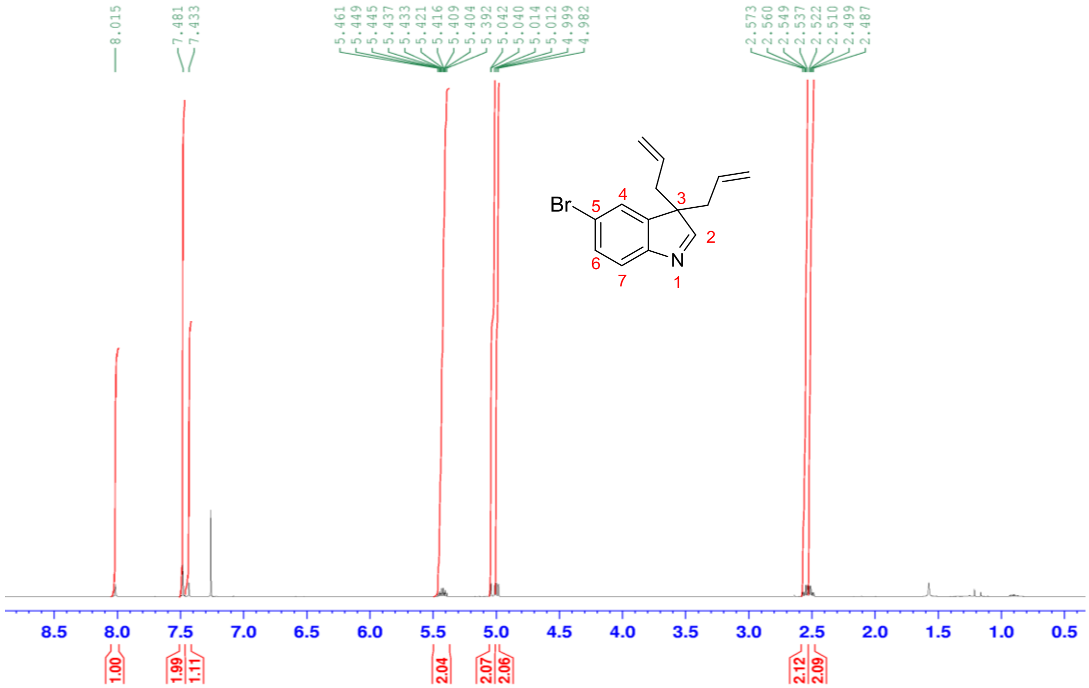

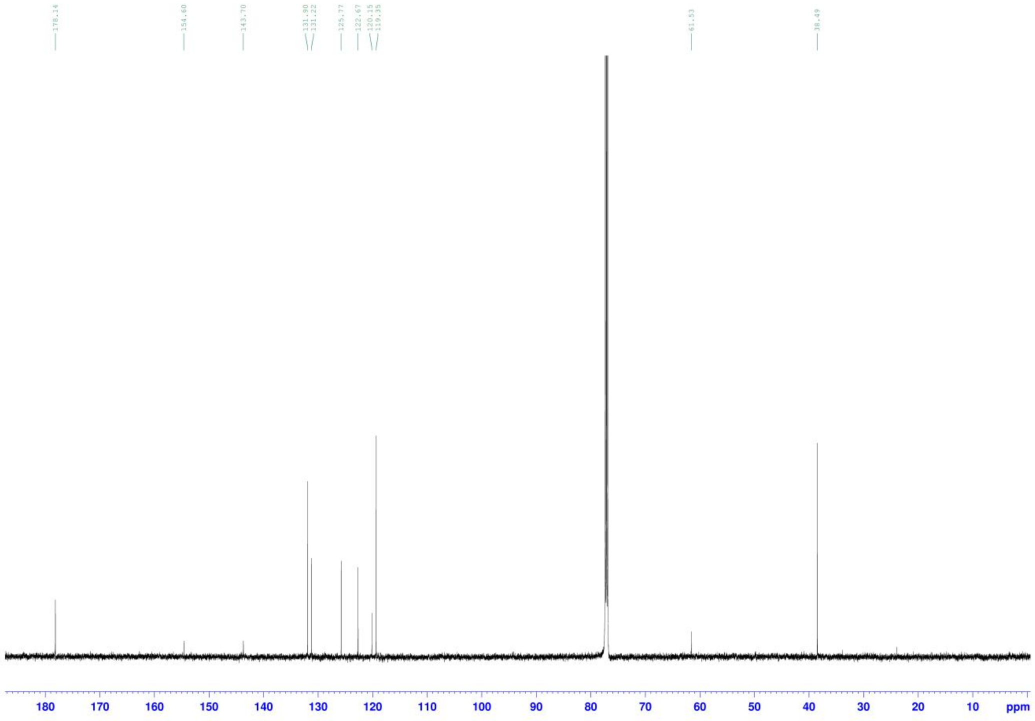

3,3-Diallyl-5-chloro-3*H*-indole (3j)

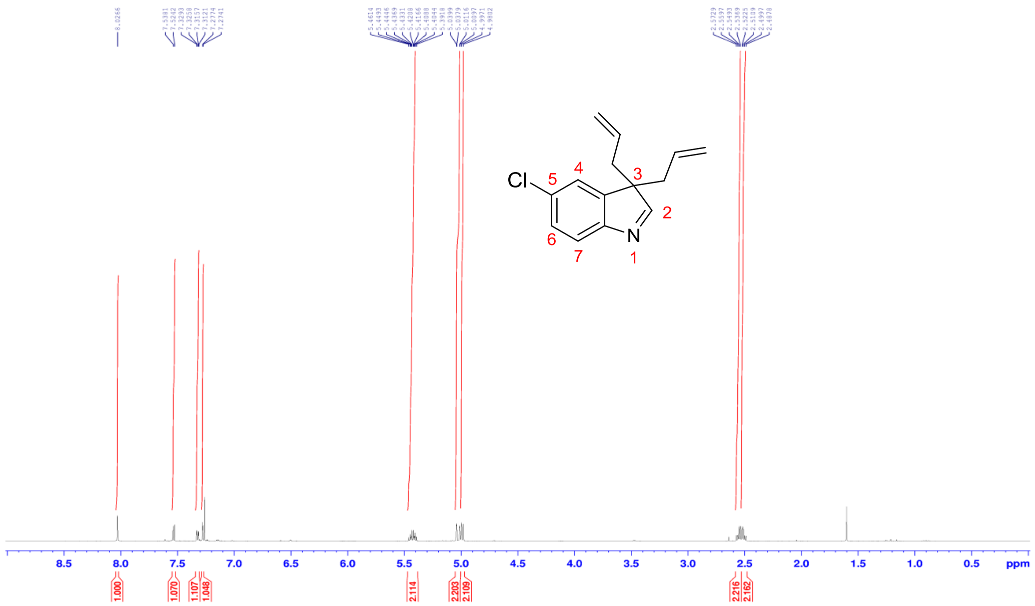

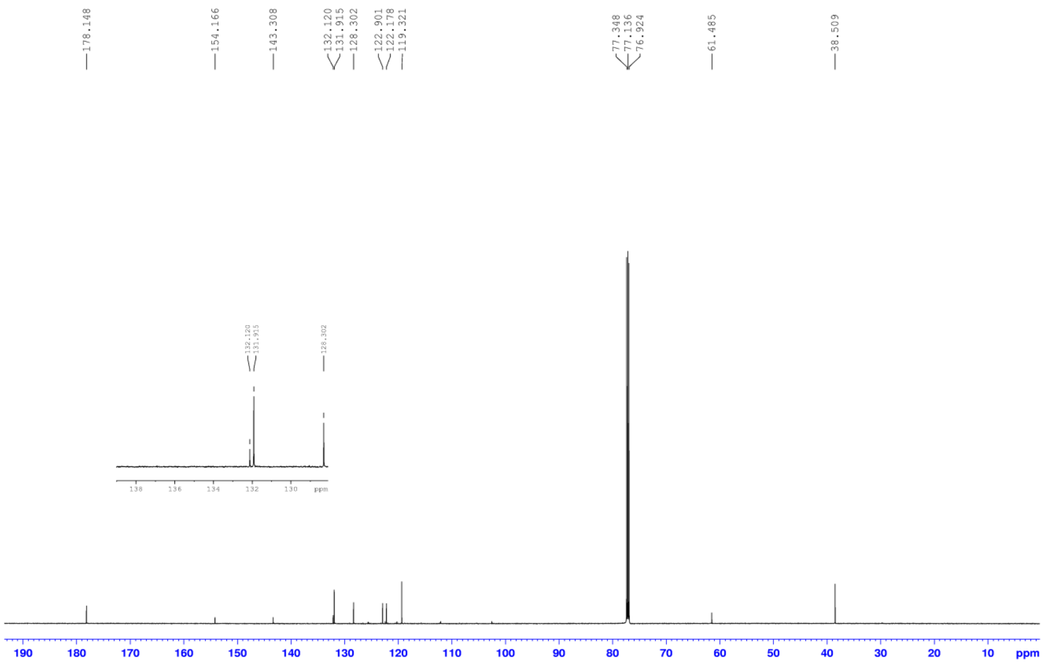

3,3-Diallyl-5-flouro-3*H*-indole (3k)

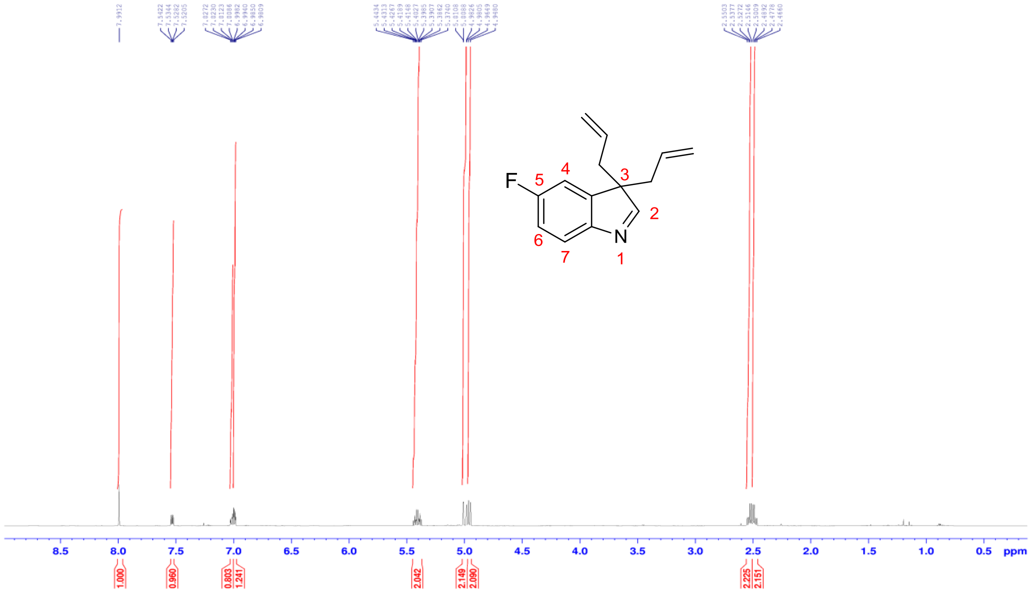

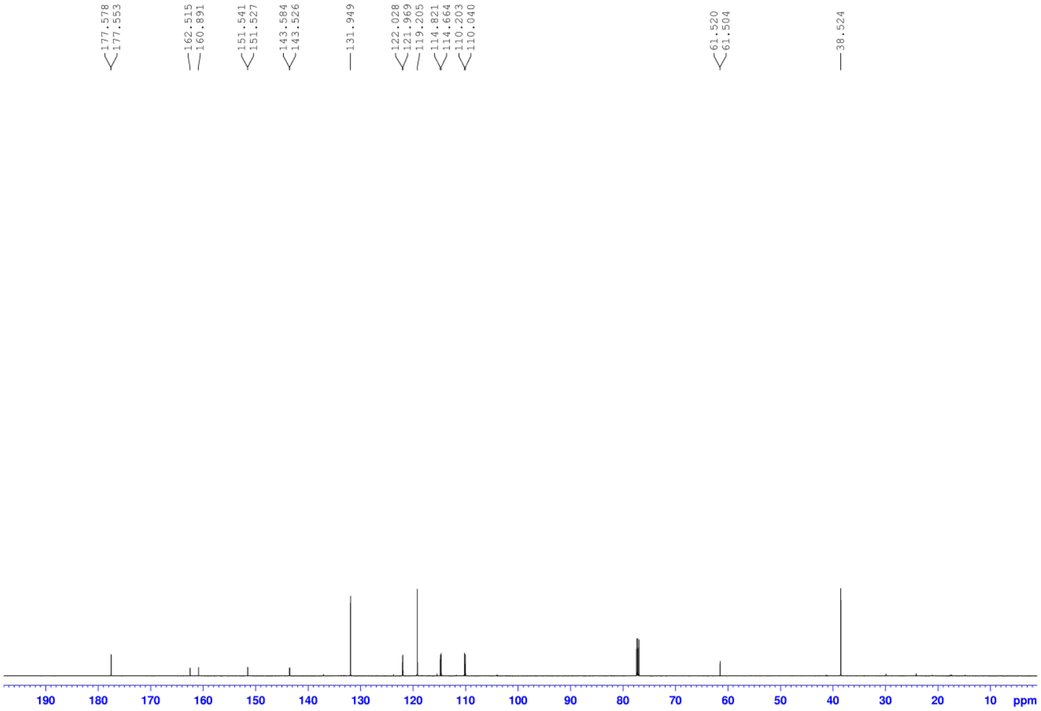

3,3-Diallyl-2-methyl-3*H*-indole (3l)

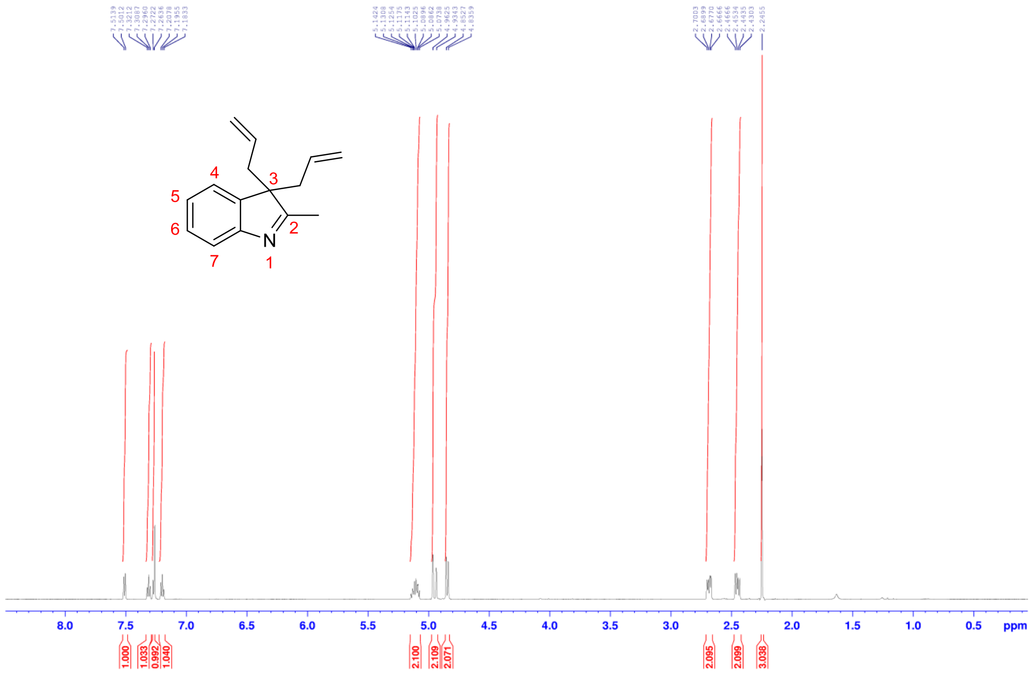

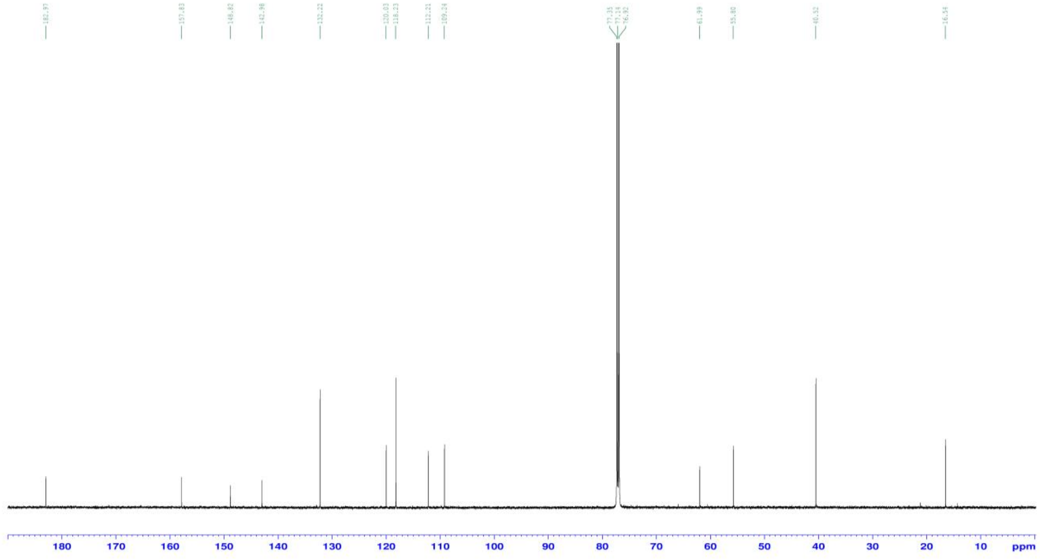

3,3-Diallyl-5-methoxy-2-methyl-3*H*-indole (3m)

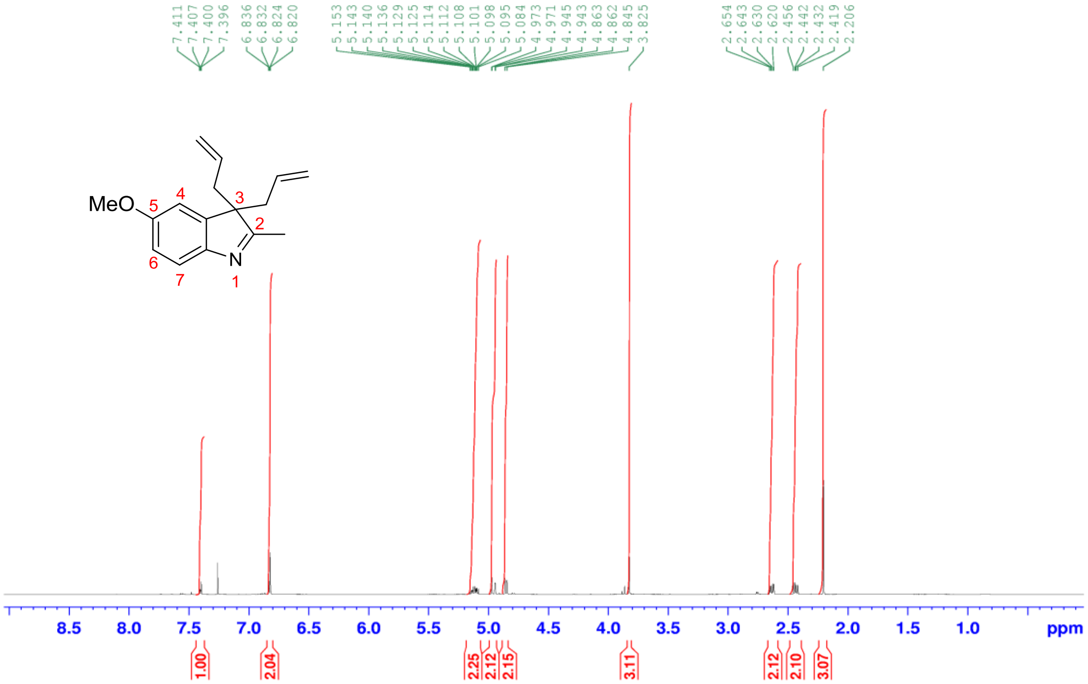

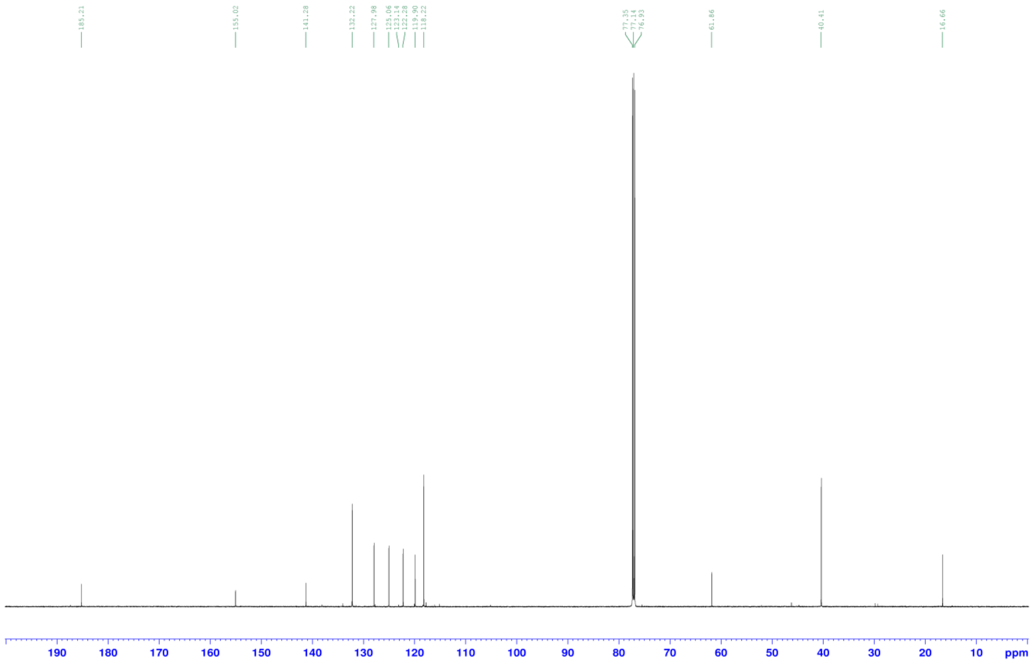

3,3-Diallyl-2-phenyl-3*H*-indole (3n)

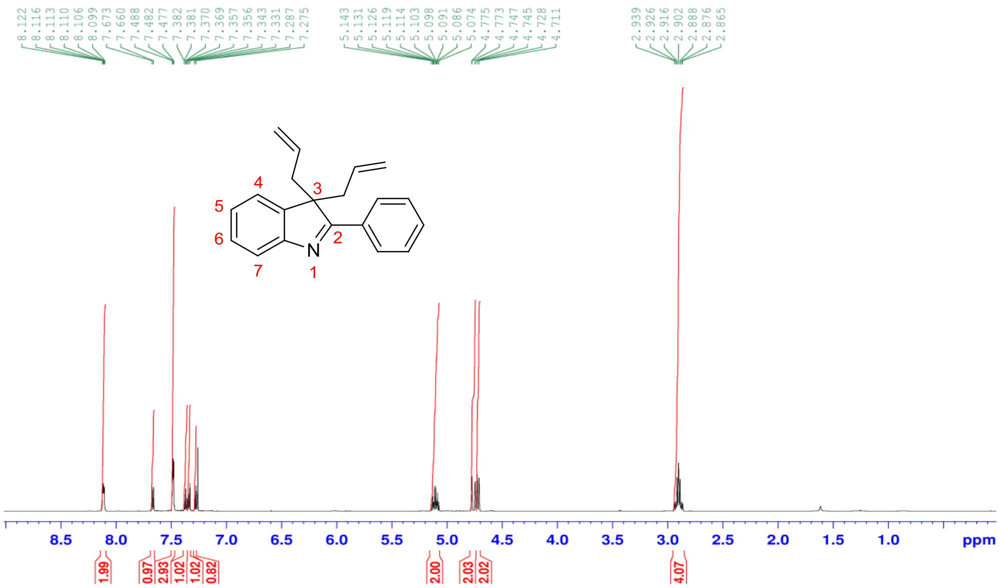

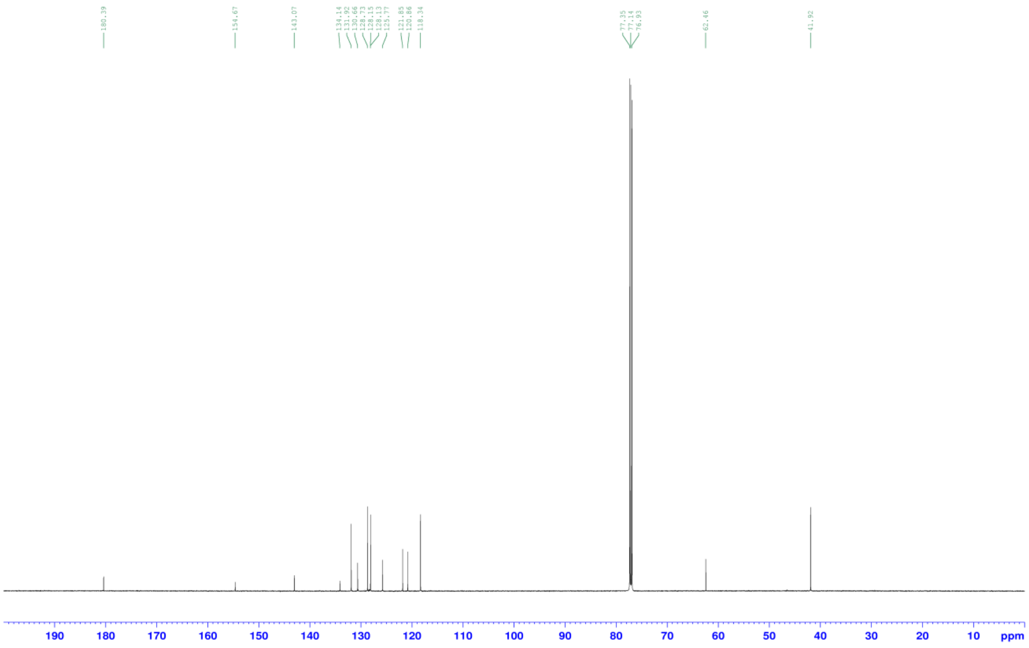

2-(((*tert*-Butyldimethylsilyl)oxy)methyl)-1*H*-indole

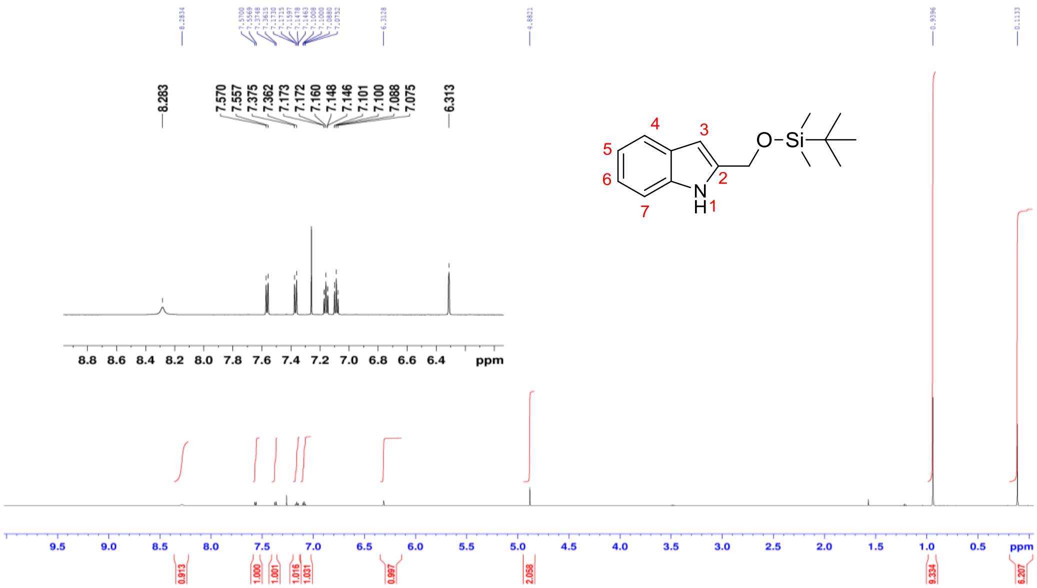

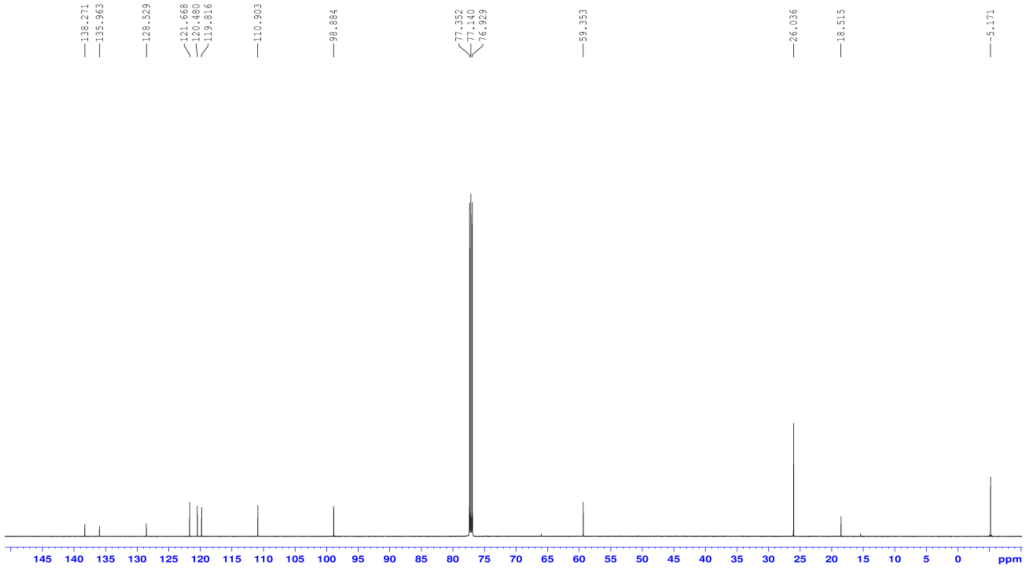

3,3-Diallyl-2-(((*tert*-butyldimethylsilyl)oxy)methyl)-3*H*-indole (3o)

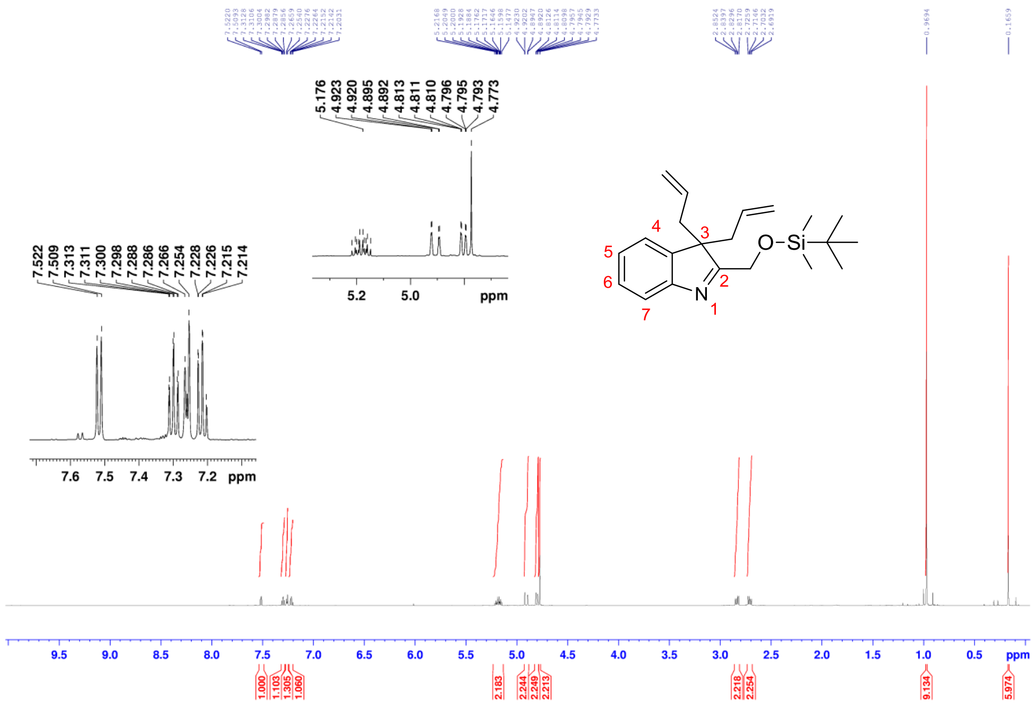

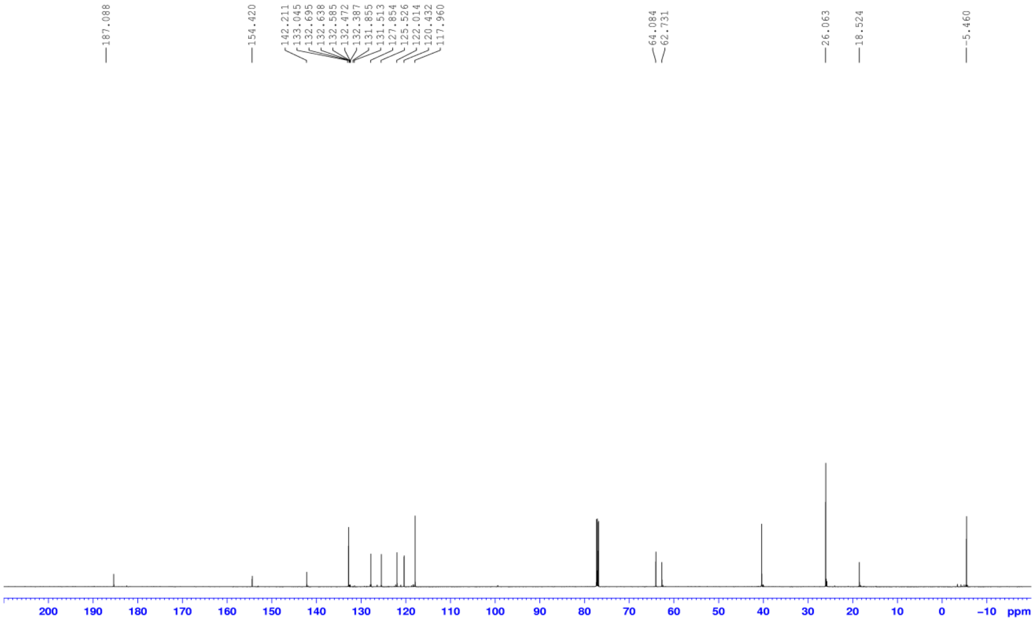

UGI reactions

3,3-Diallyl-1-benzoyl-*N*-(*tert*-butyl)indoline-2-carboxamide (8a)

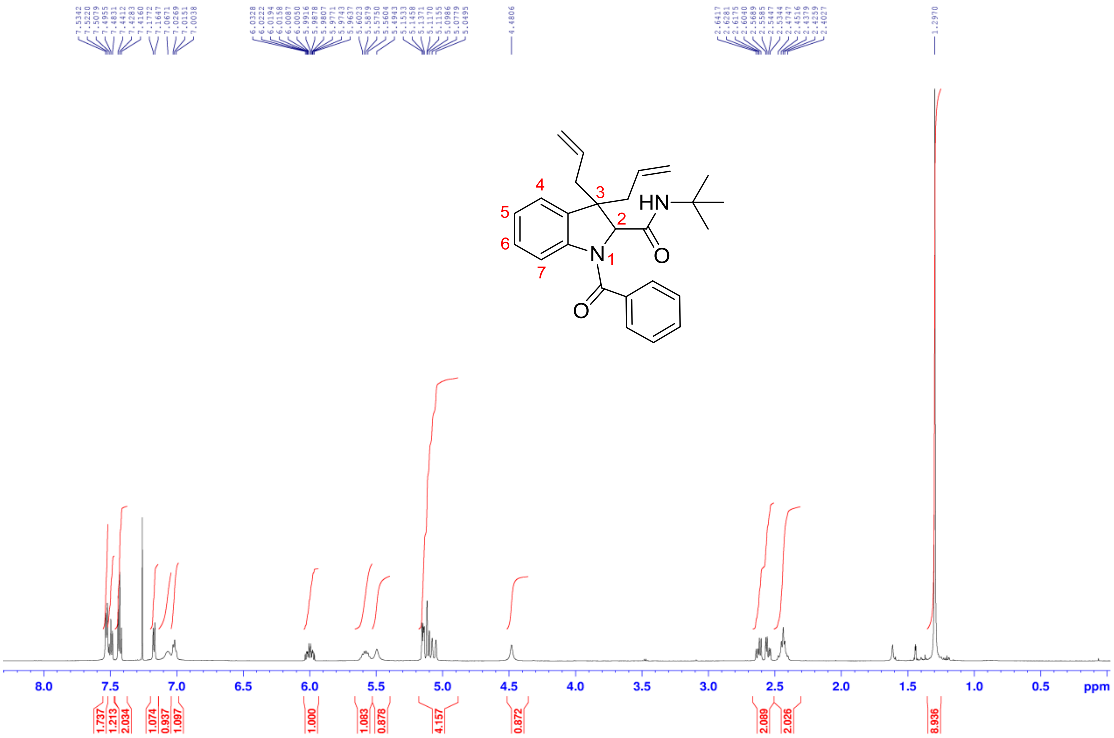

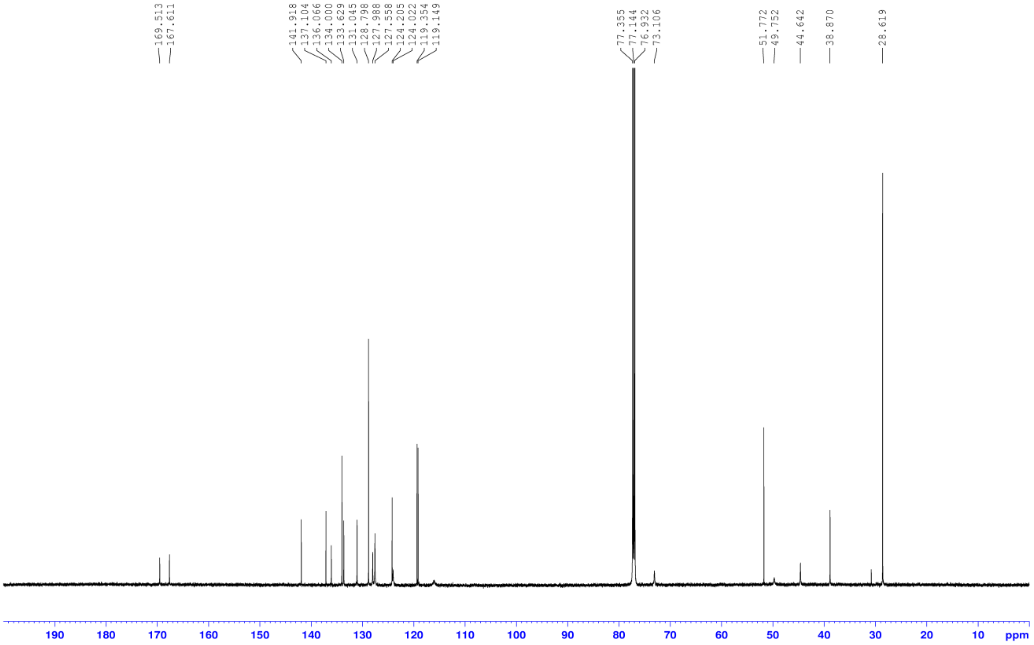

3,3-Diallyl-1-benzoyl-*N*-cyclohexylindoline-2-carboxamide (8b)

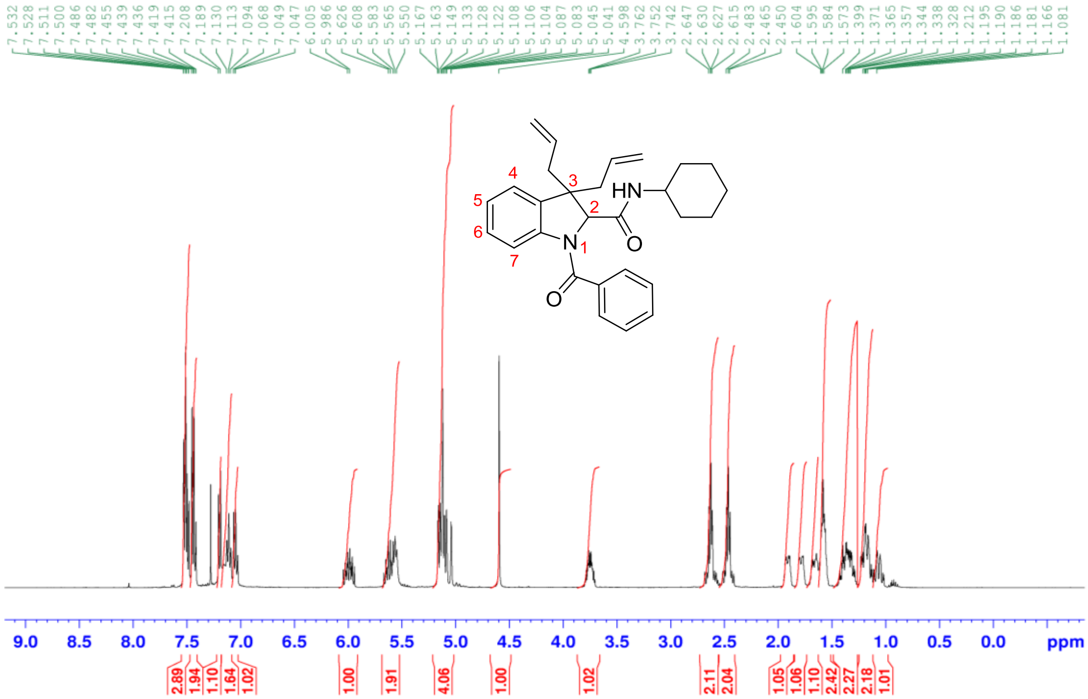

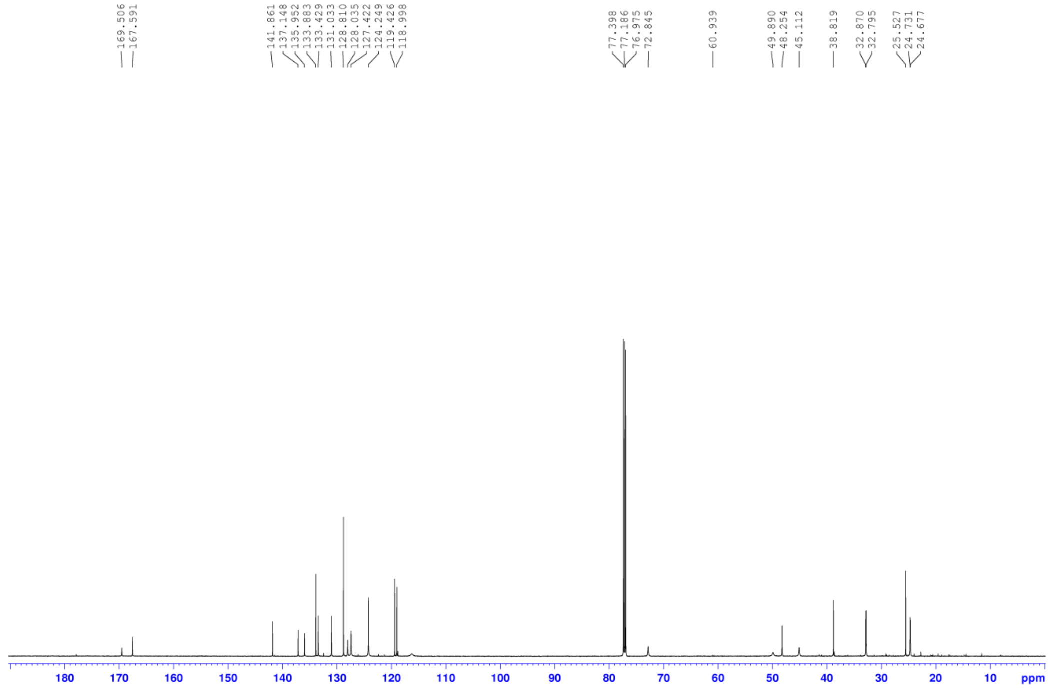

3,3-Diallyl-1-benzoyl-*N*-(*tert*-butyl)-5,6-dimethoxyindoline-2-carboxamide (8c)

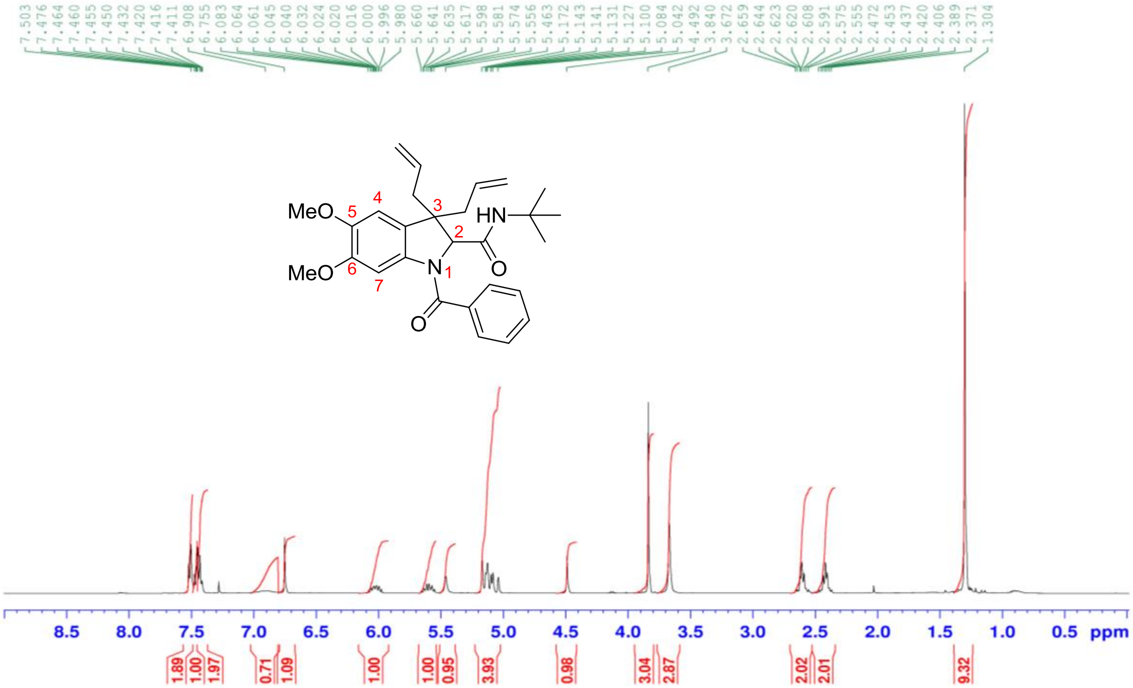

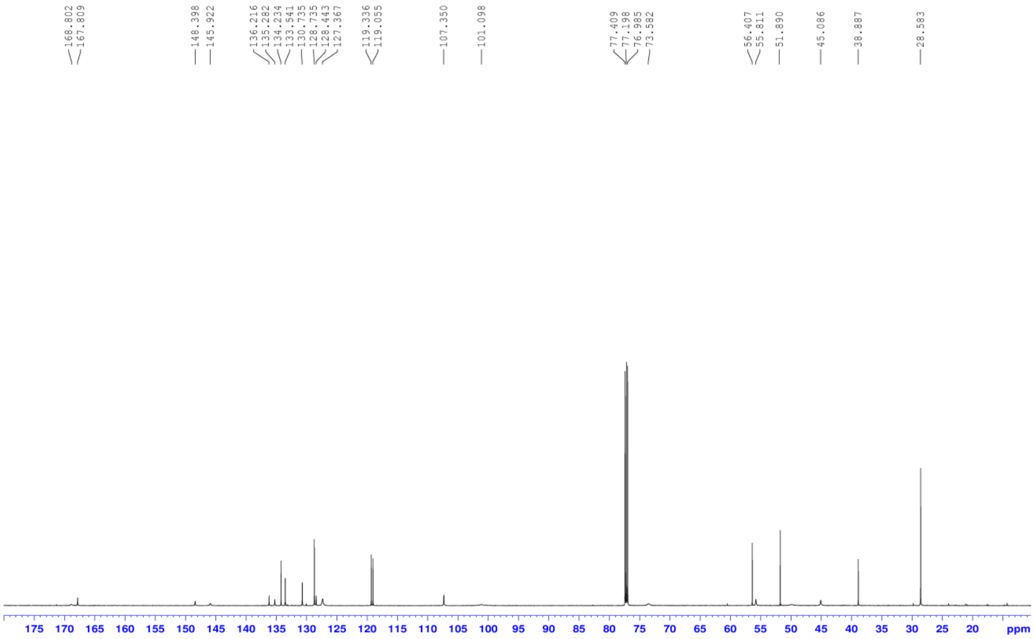

3,3-Diallyl-*N*-(*tert*-butyl)-1-(2-(2-fluoro)phenyl)acetyl)indoline-2-carboxamide (8d)

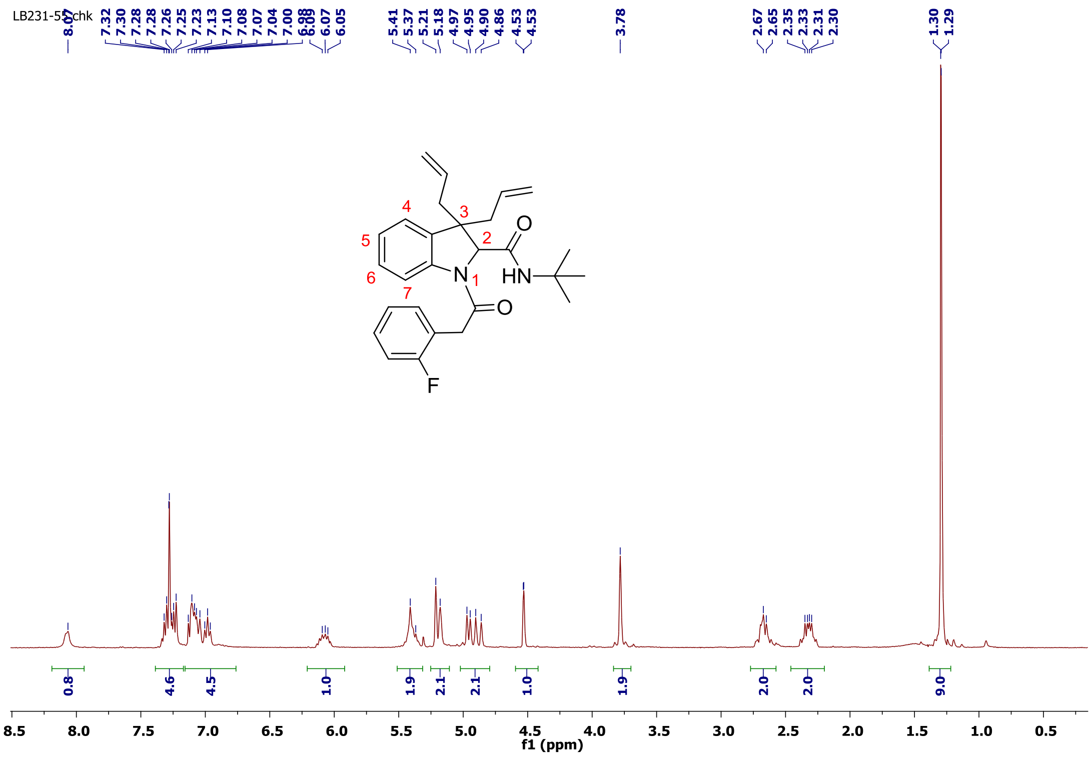

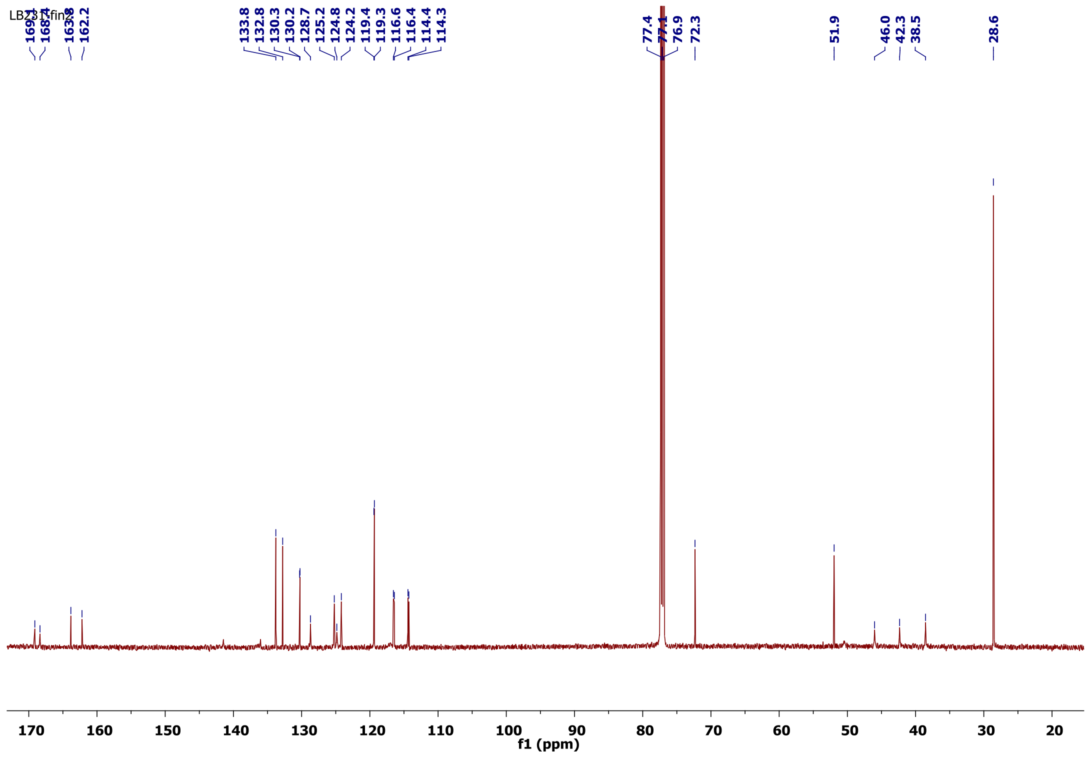

3,3-Diallyl-5-(benzyloxy)-*N*-(*tert*-butyl)-1-picolinoylindoline-2-carboxamide (8e)

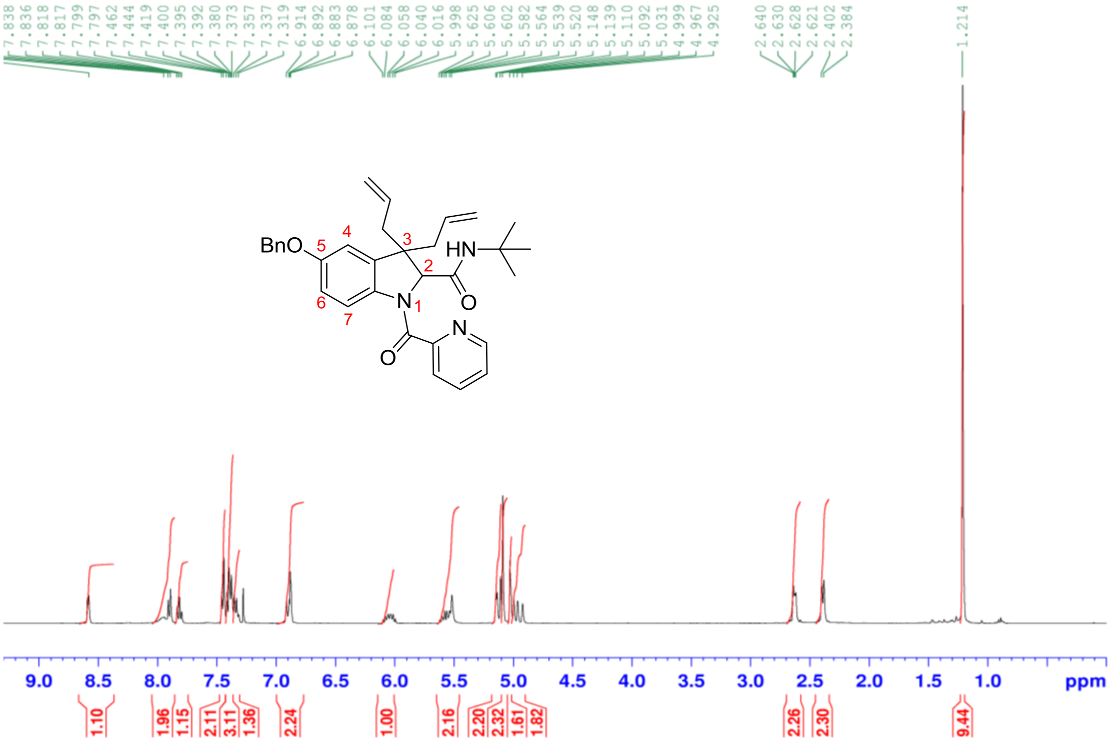

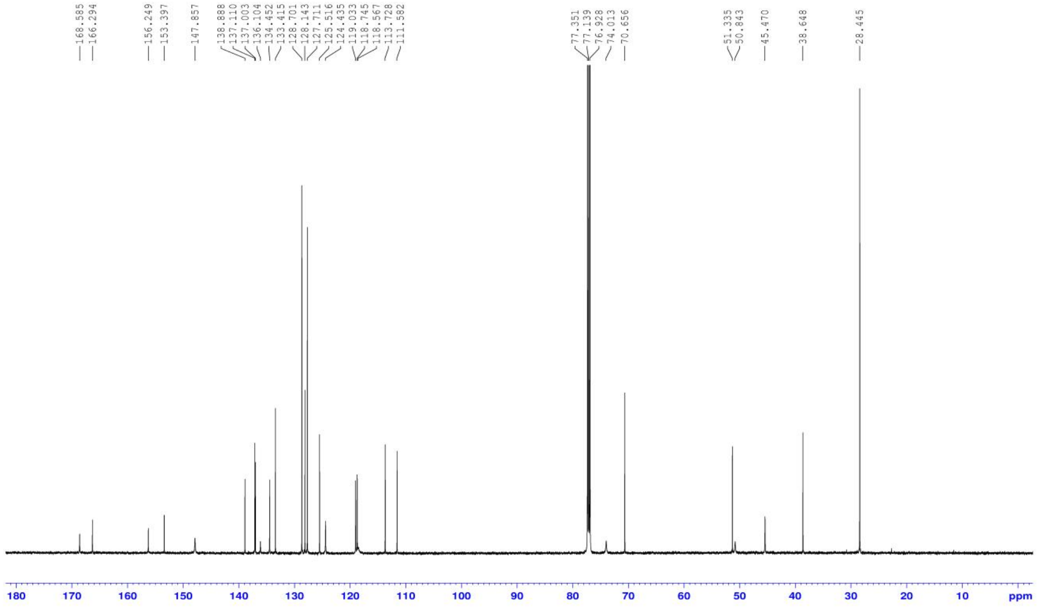

3,3-Diallyl-*n*pentyl-1-(1*H*-indole-3-carbonyl)indoline-2-carboxamide (8f)

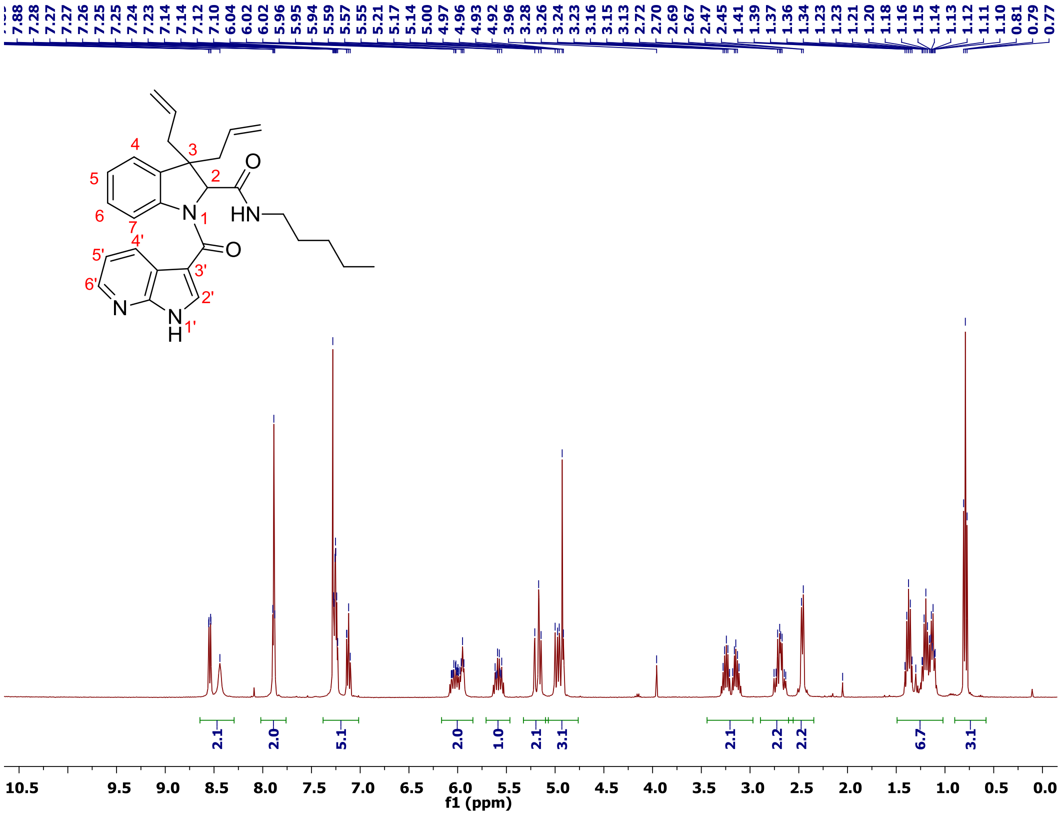

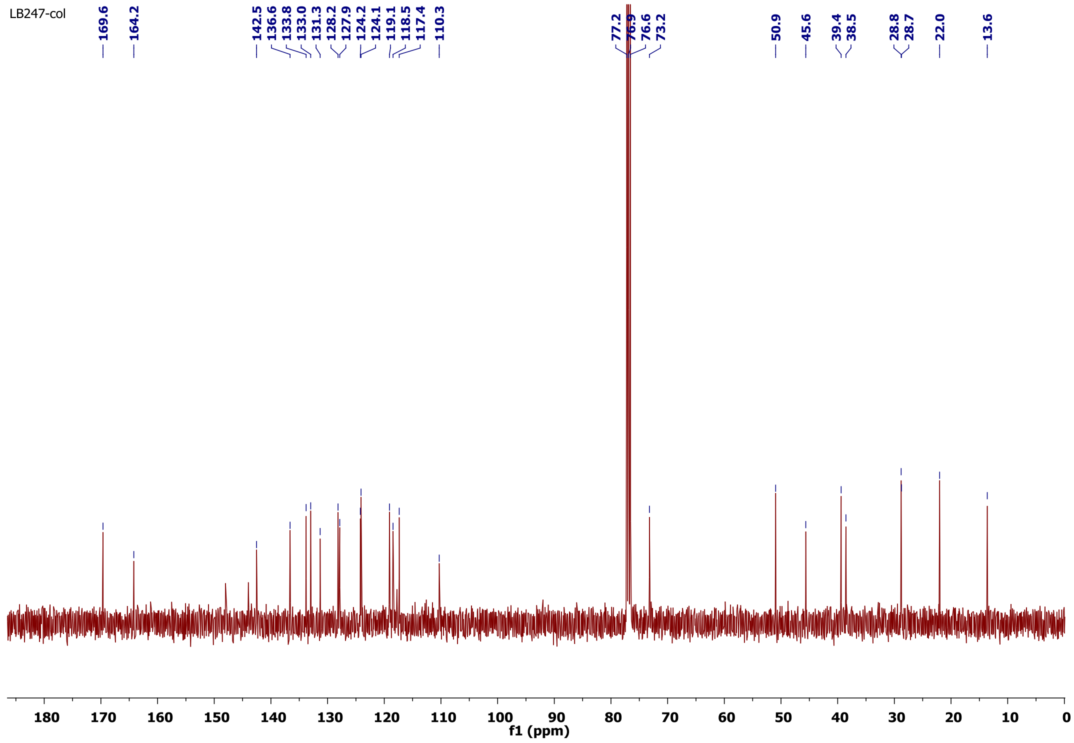

3,3-Diallyl-*N*-(*tert*-butyl)-1-(1*H*-pyrazole-3-carbonyl)indoline-2-carboxamide (8g)

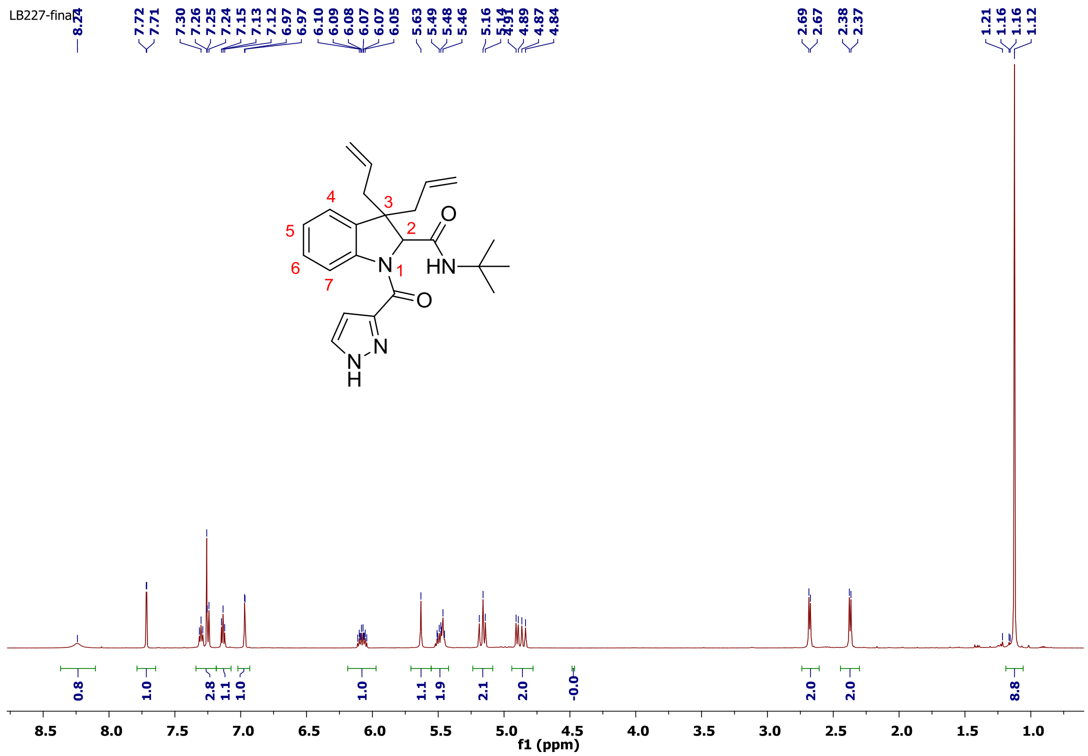

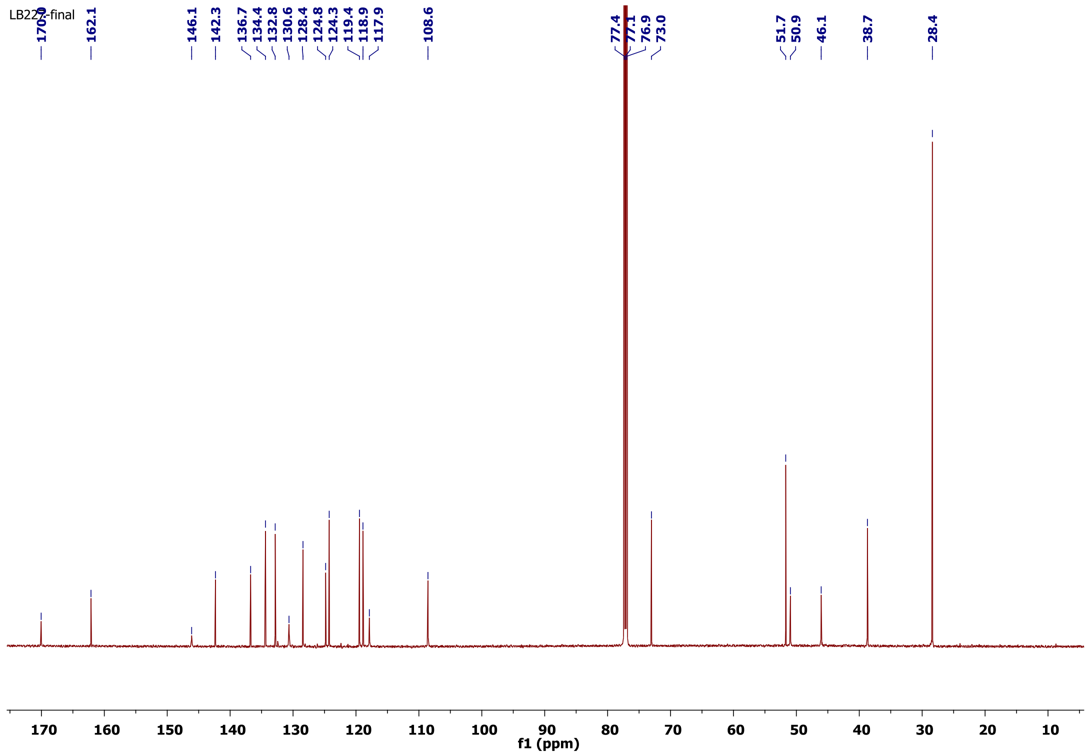

3,3-Diallyl-1-(1-carbamoylcyclopropanecarbonyl)-*N*-(4-methoxyphenyl)indoline-2-carboxamide (8h)

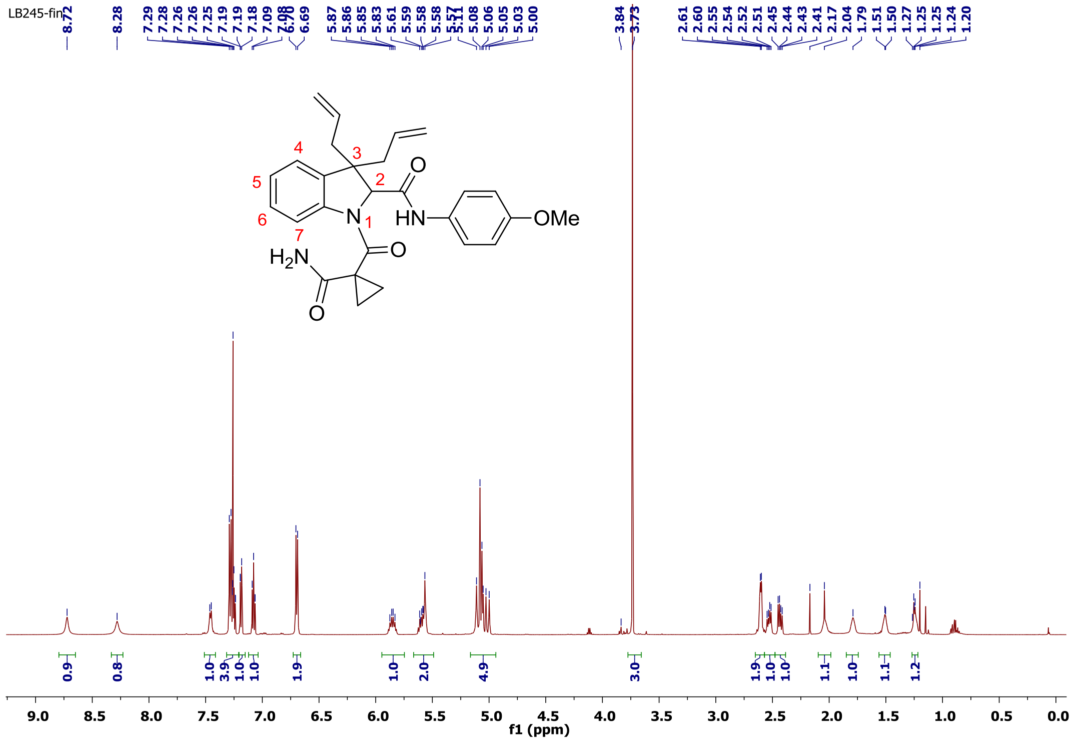

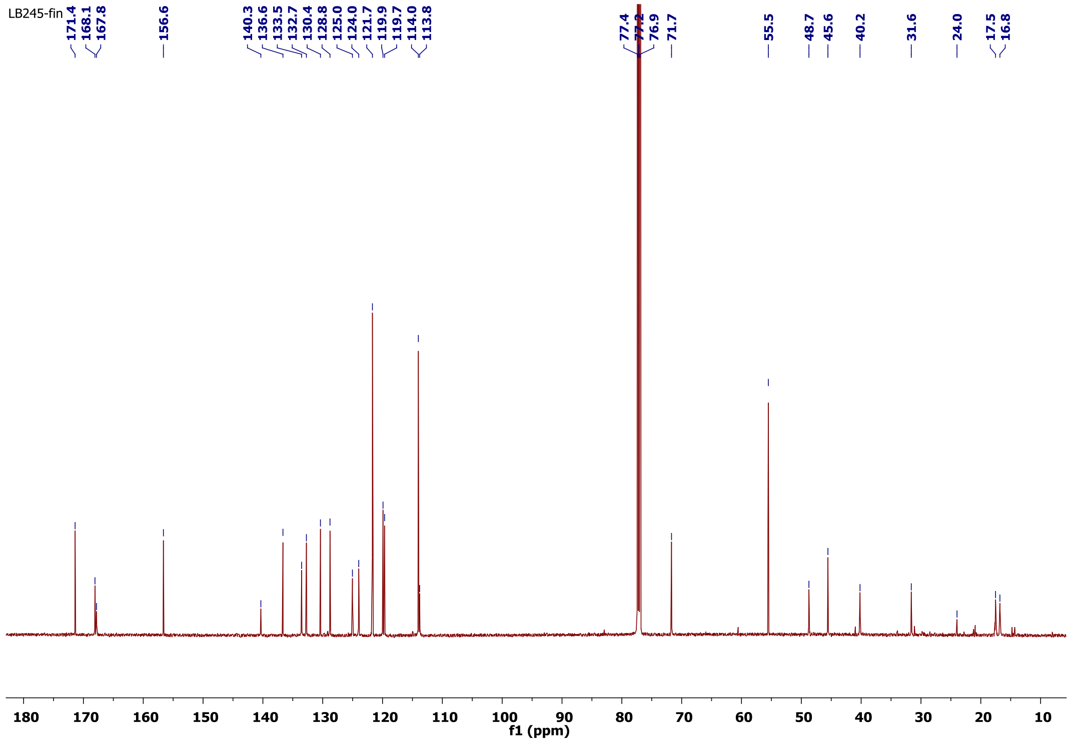

3,3-Diallyl-1-(2-chloroacetyl)-5-methoxy-*N*-*n*pentylindoline-2-carboxamide (8i)

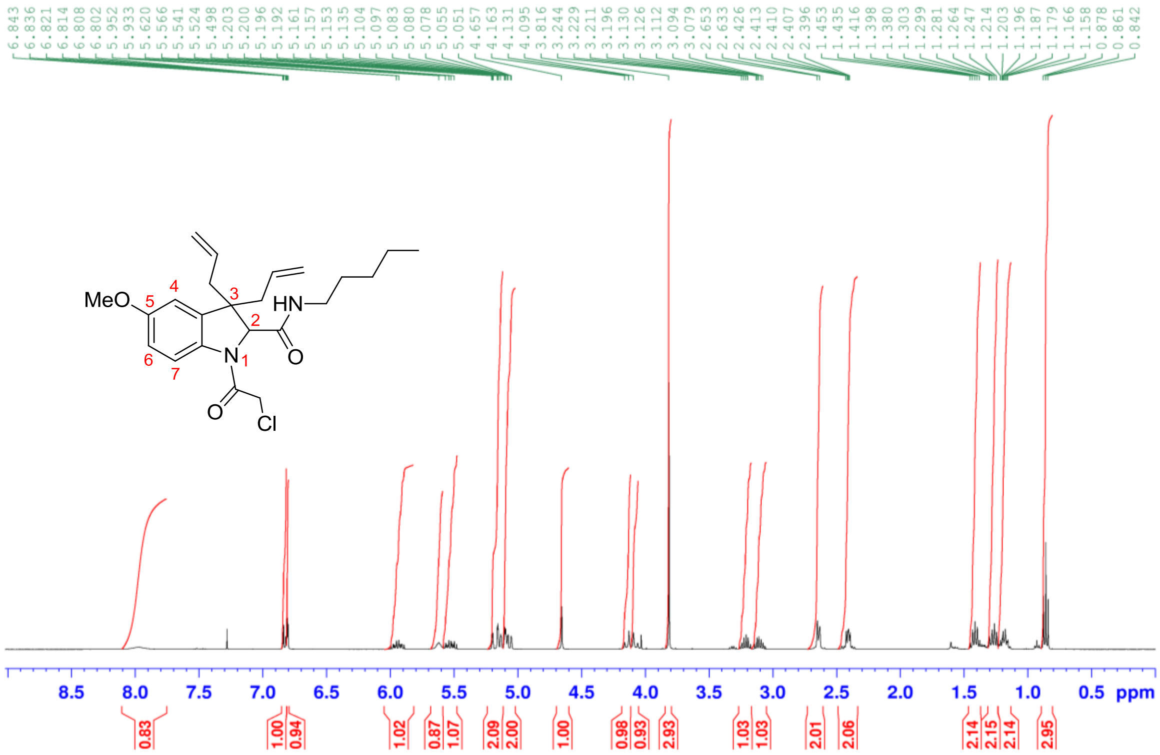

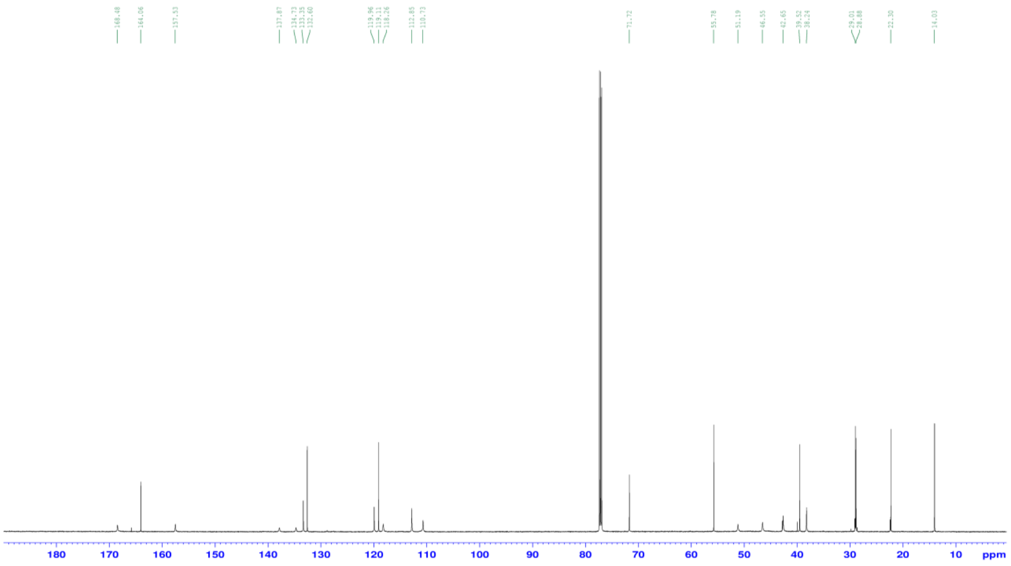

***tert*-Butyl(2-(3,3-diallyl-2-(*tert*-butylcarbamoyl)-5-chloroindolin-1-yl)-2-(oxoethyl)carbamate (8j)**

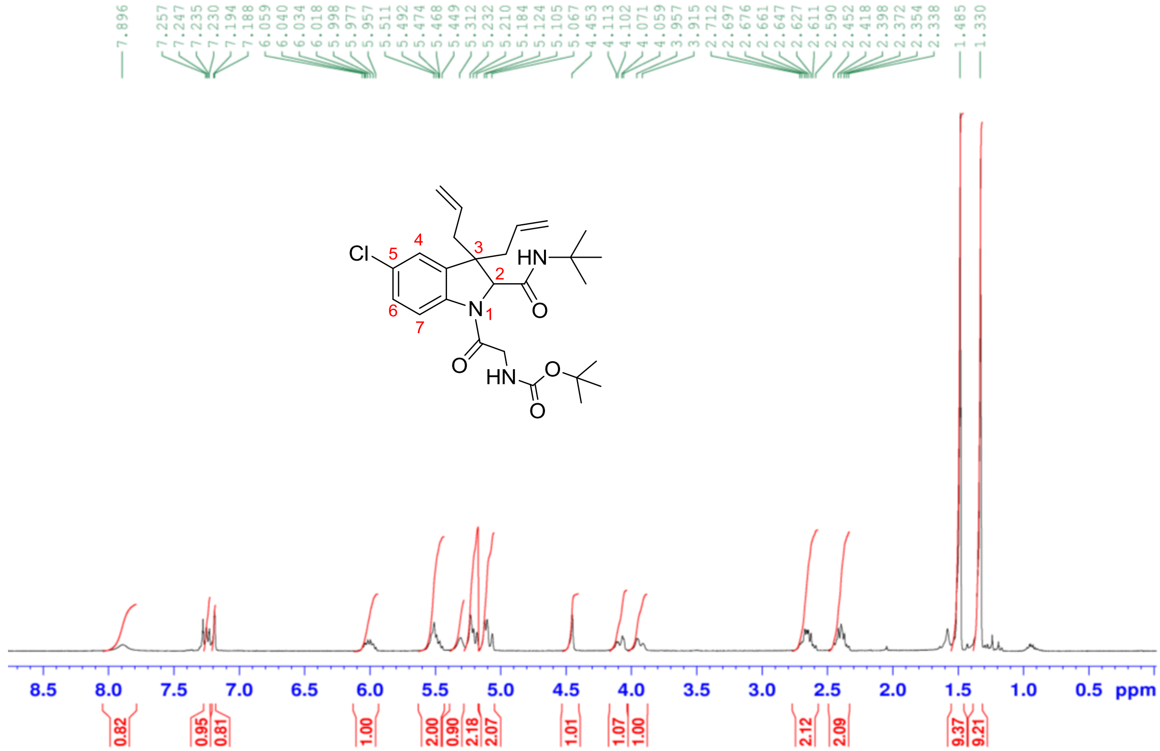

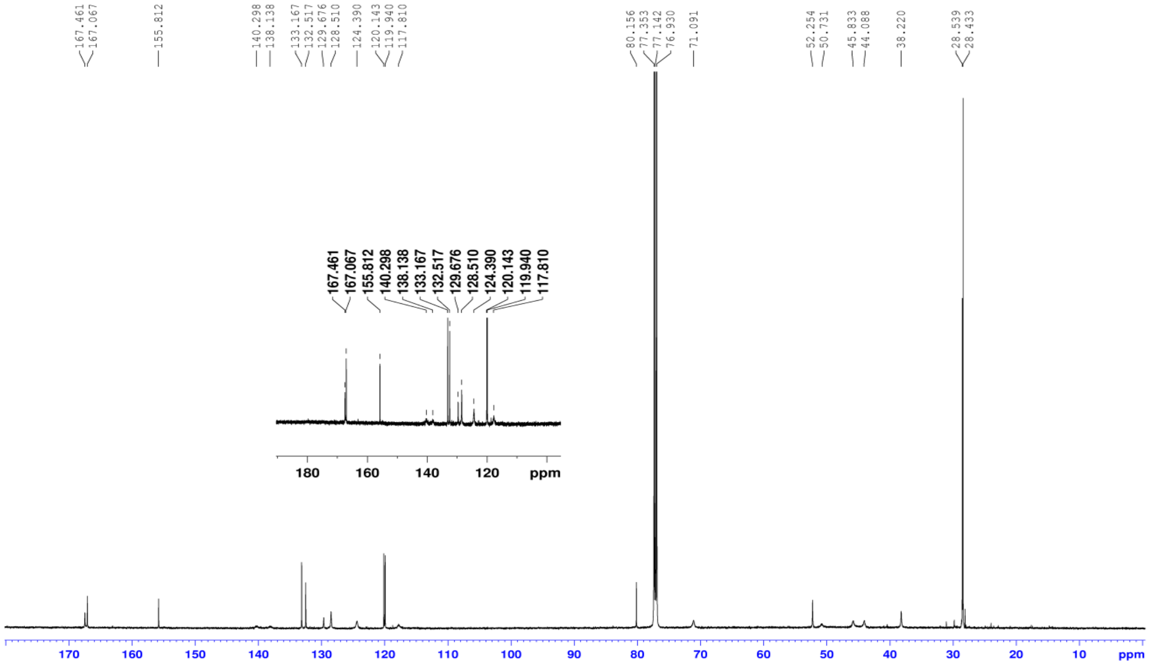

tert-Butyl (2-(3,3-diallyl-2-(_tert_-butylcarbamoyl)indolin-1-yl)-2-oxoethyl)carbamate (8k)

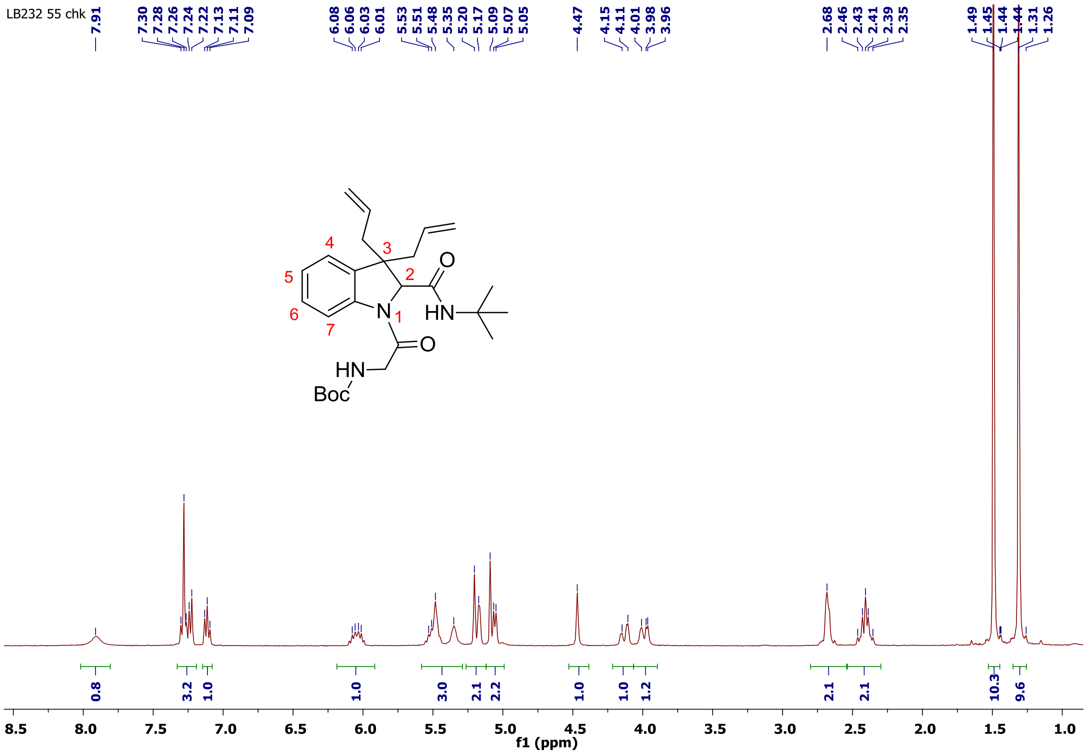

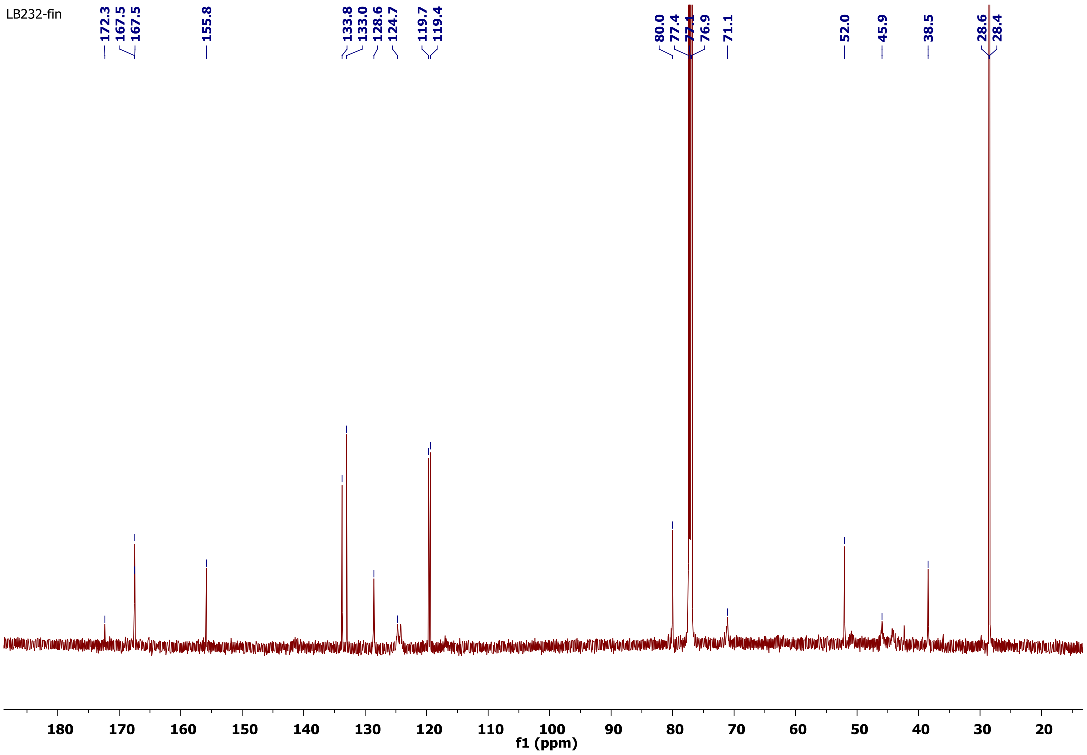

3,3-Diallyl-*N*-(*tert*-butyl)-1-(2-(methylamino)acetyl)indoline-2-carboxamide (8l)

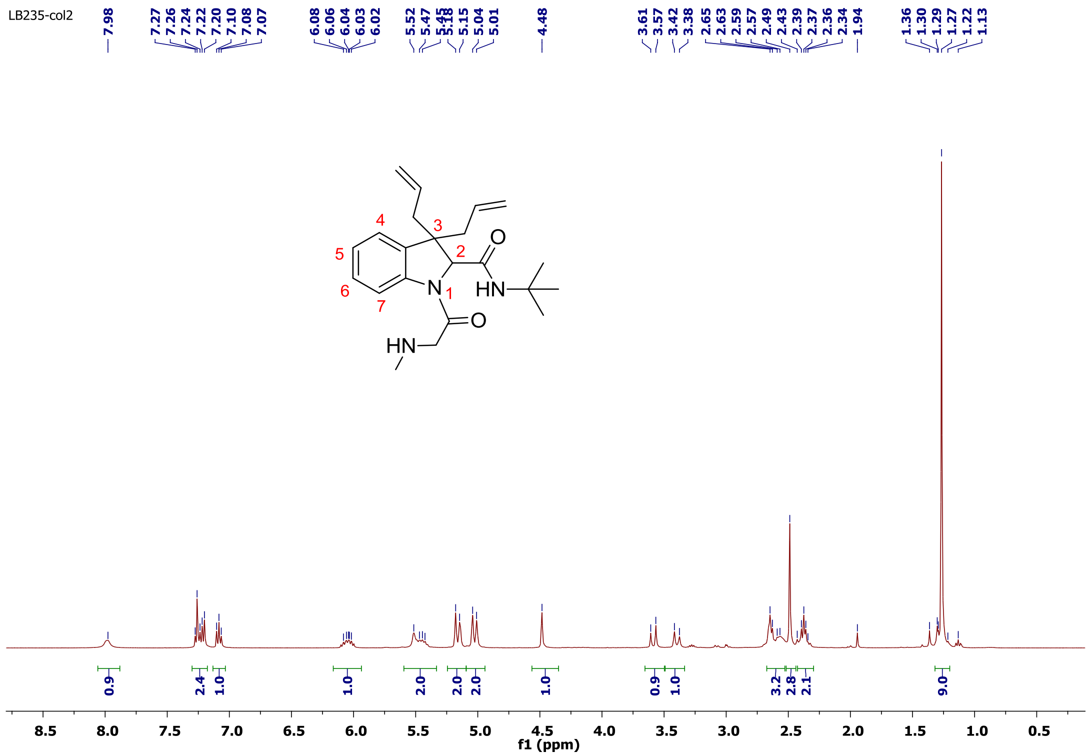

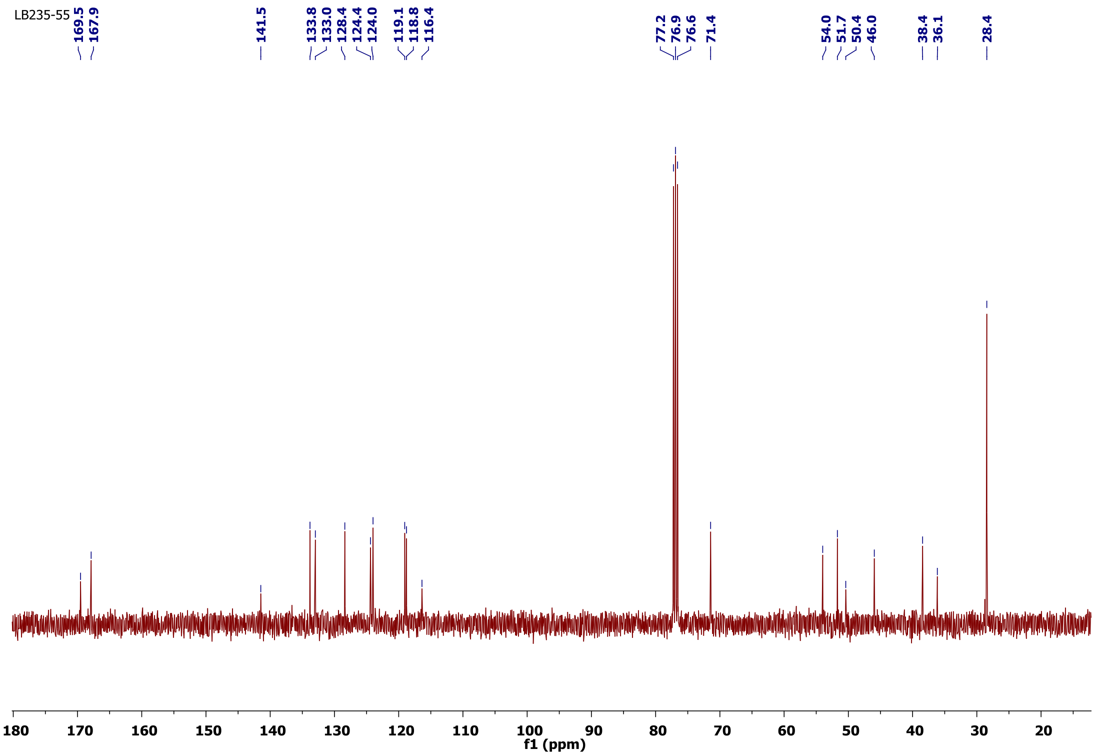

3,3-Diallyl-1-((S)-2-amino-3-phenylpropanoyl)-*N*-(*tert*-butyl)indoline-2-carboxamide (8m)

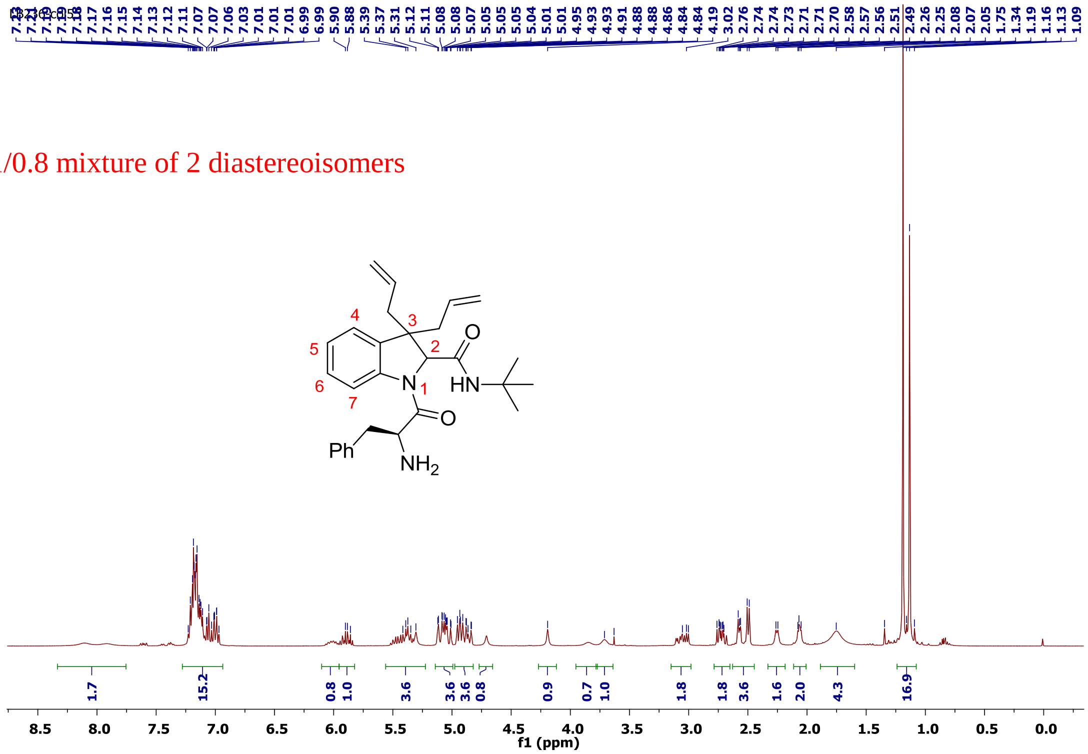

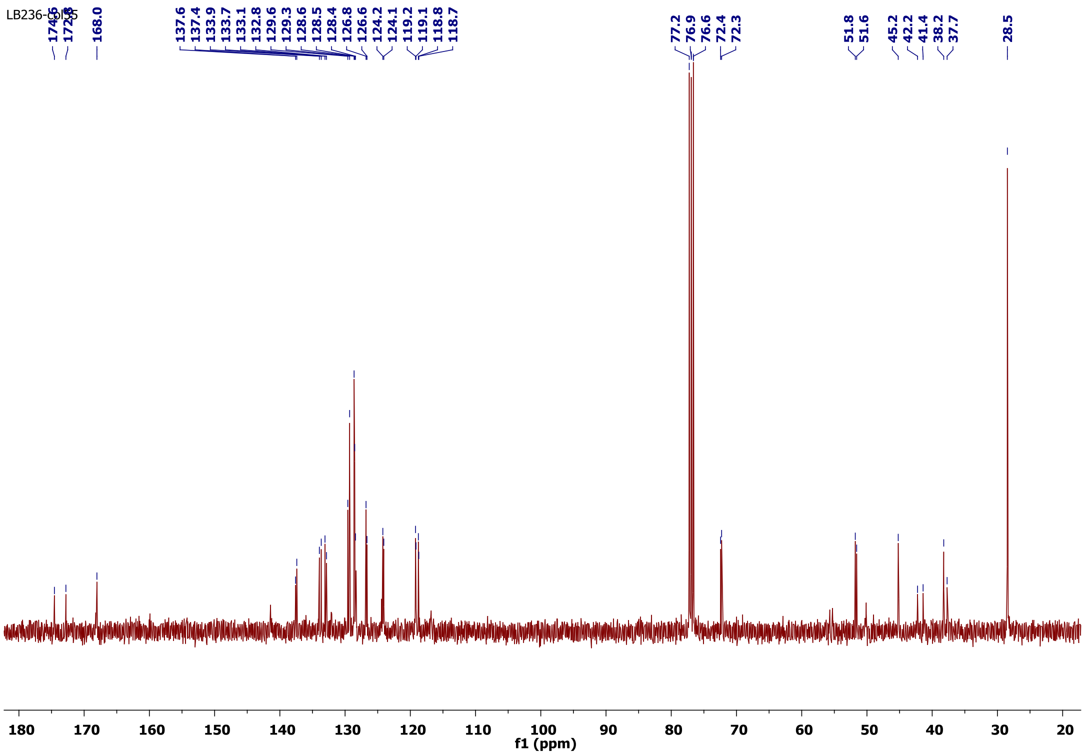

3,3-Diallyl-*N*-(*tert*-butyl)-1-((R)-2-hydroxy-2-phenylacetyl)-5-methoxyindoline-2-carboxamide (8n)

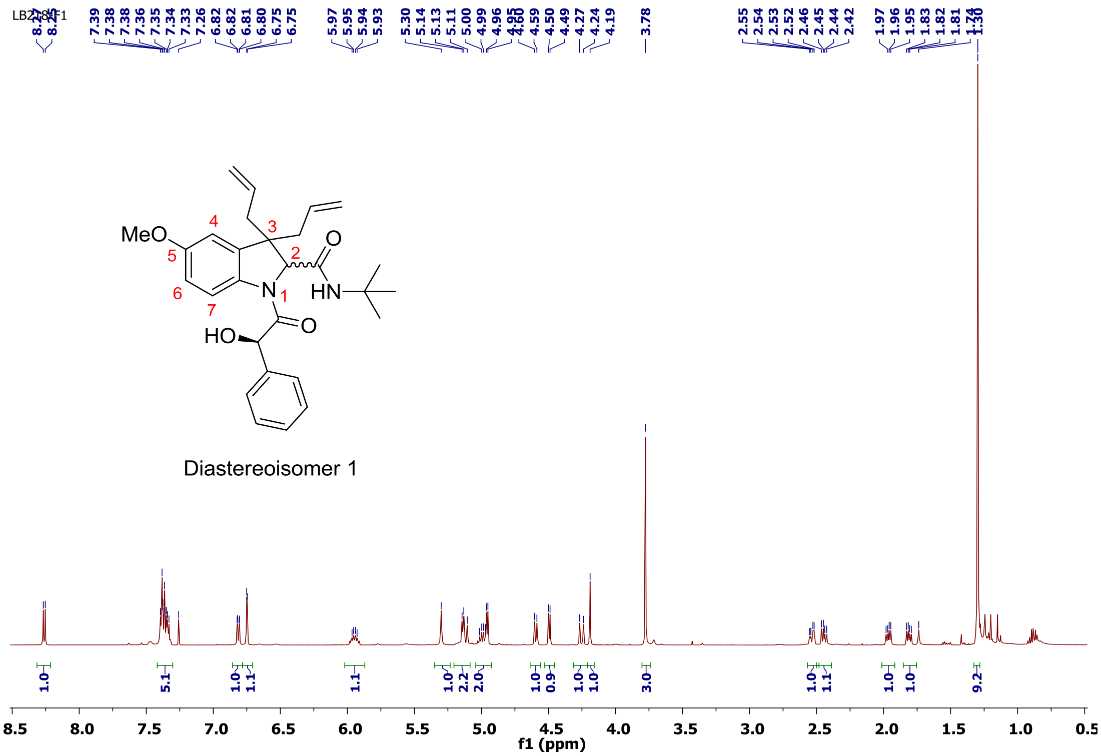

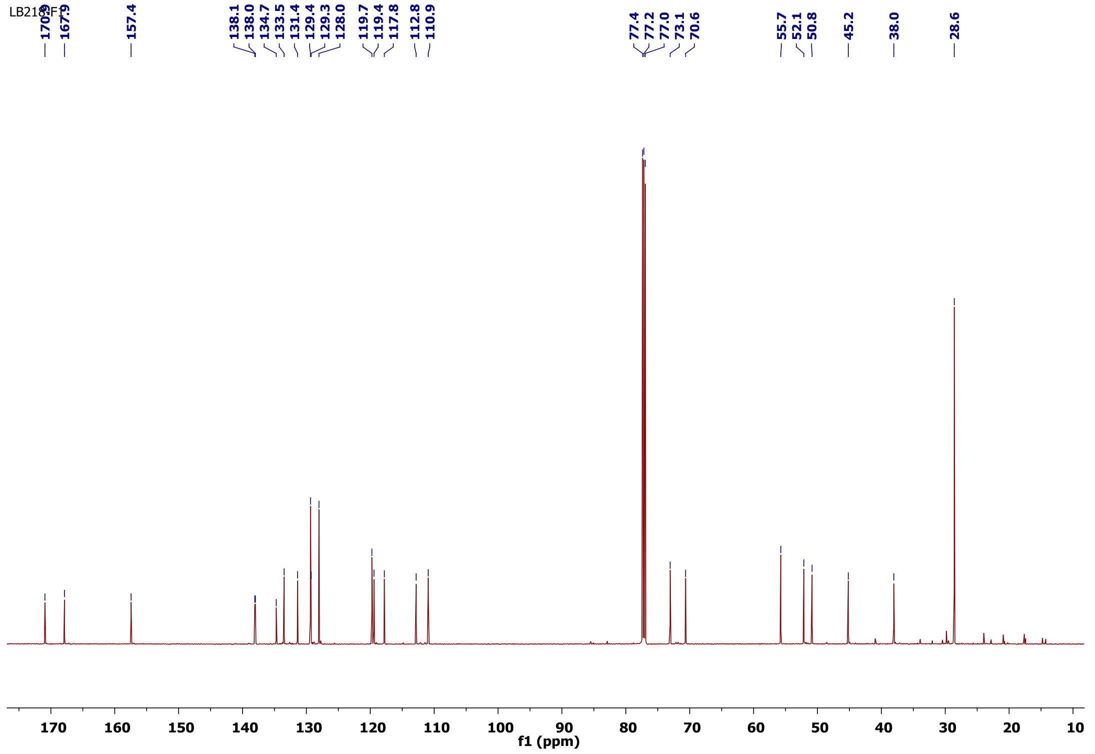

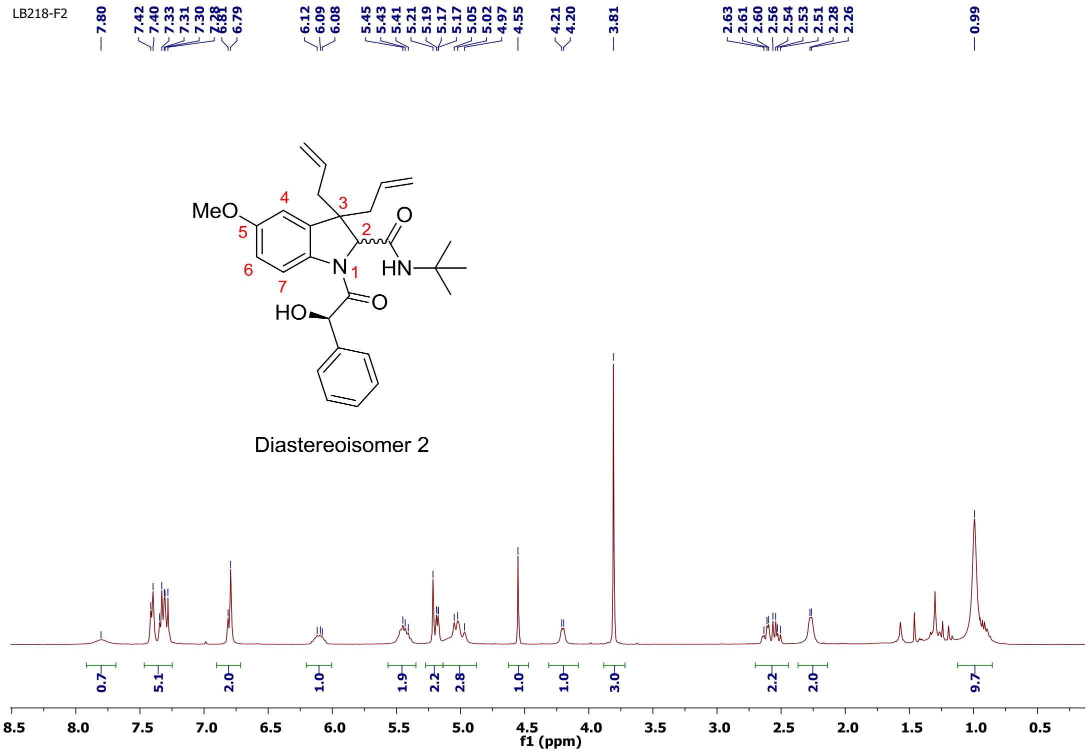
LB218-F2
7.80
7.42
7.40
7.33
7.31
7.30
7.28
6.81
6.79
6.12
6.09
6.08
5.45
5.43
5.41
5.21
5.19
5.17
5.17
5.05
5.02
4.97
4.55
4.21
4.20
3.81
2.63
2.61
2.60
2.56
2.54
2.53
2.51
2.28
2.26
0.99
MeO
4
5
6
7
3
2
N
1
O
HN
HO
O
Diastereoisomer 2
0.7
5.1
2.0
1.0
1.9
2.2
2.8
1.0
1.0
3.0
2.2
2.0
9.7
8.5
8.0
7.5
7.0
6.5
6.0
5.5
5.0
4.5
4.0
3.5
3.0
2.5
2.0
1.5
1.0
0.5
f1 (ppm)

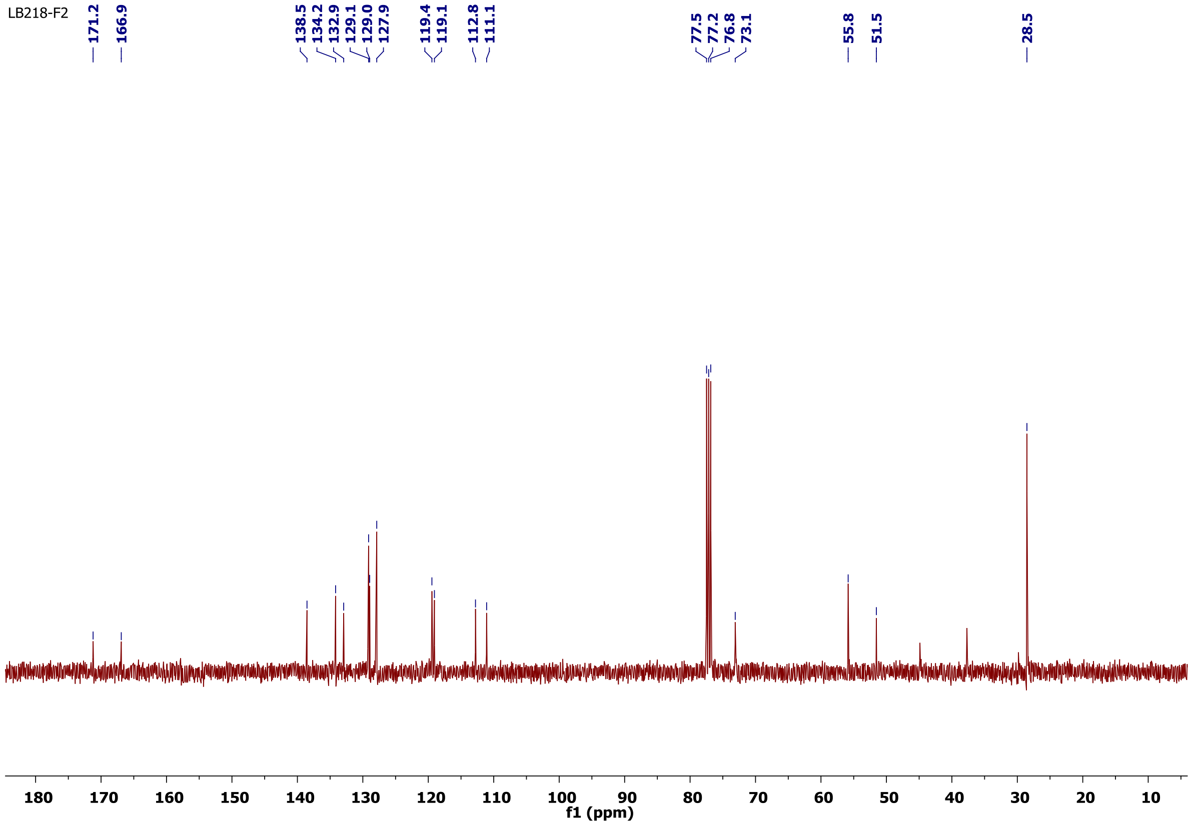
LB218-F2
171.2
166.9
138.5
134.2
132.9
129.1
129.0
127.9
119.4
119.1
112.8
111.1
77.5
77.2
76.8
73.1
55.8
51.5
28.5
180
170
160
150
140
130
120
110
100
90
80
70
60
50
40
30
20
10
f1 (ppm)

Methyl 3,3-diallyl-2-hydroxyindoline-1-carboxylate (10a)

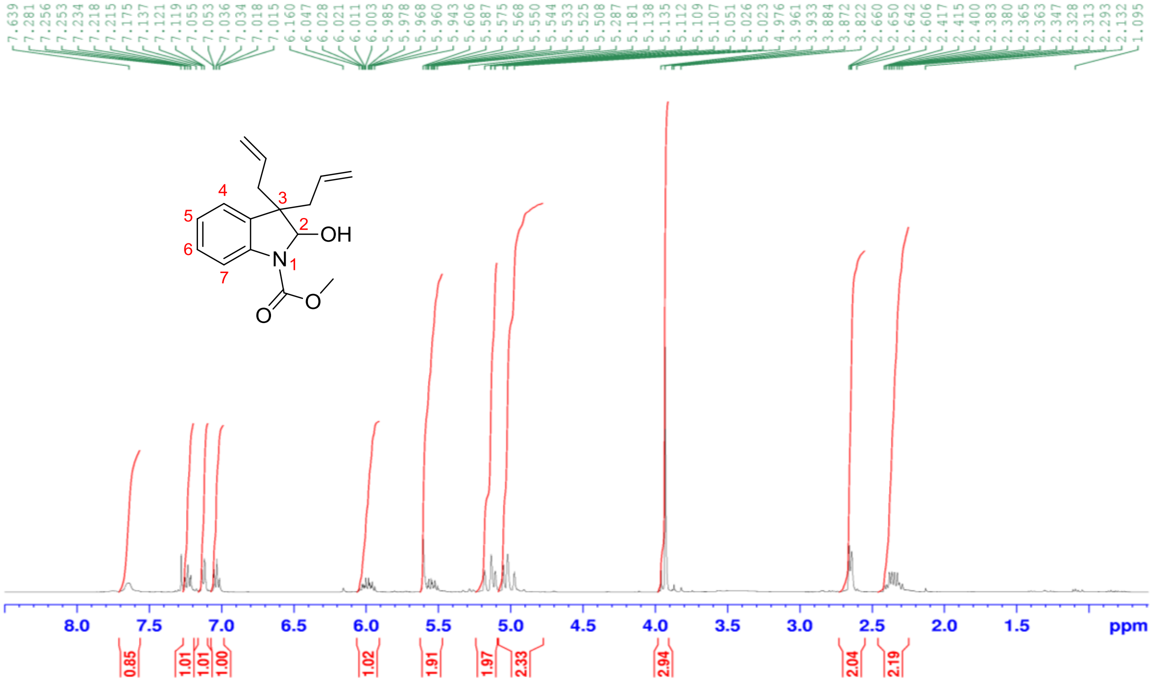

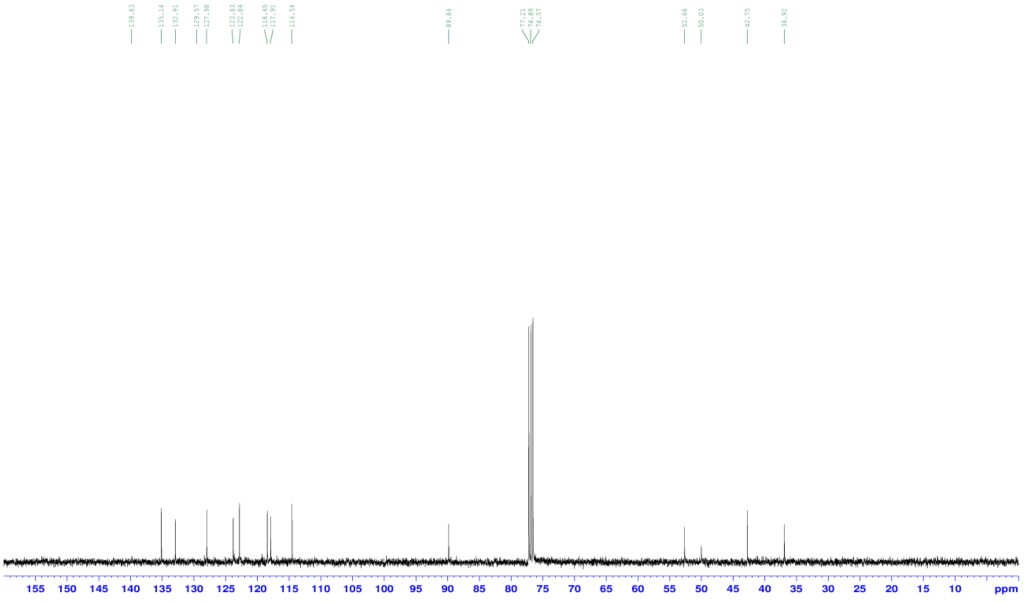

Methyl 3,3-diallyl-2-hydroxy-5-methoxyindoline-1-carboxylate (10b)

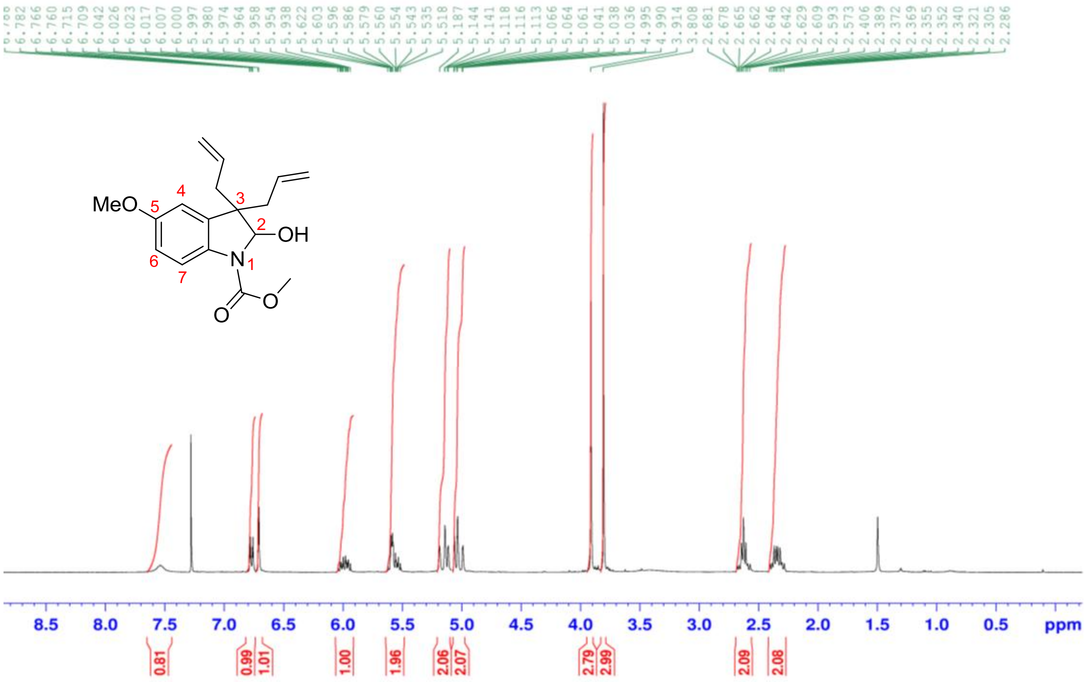

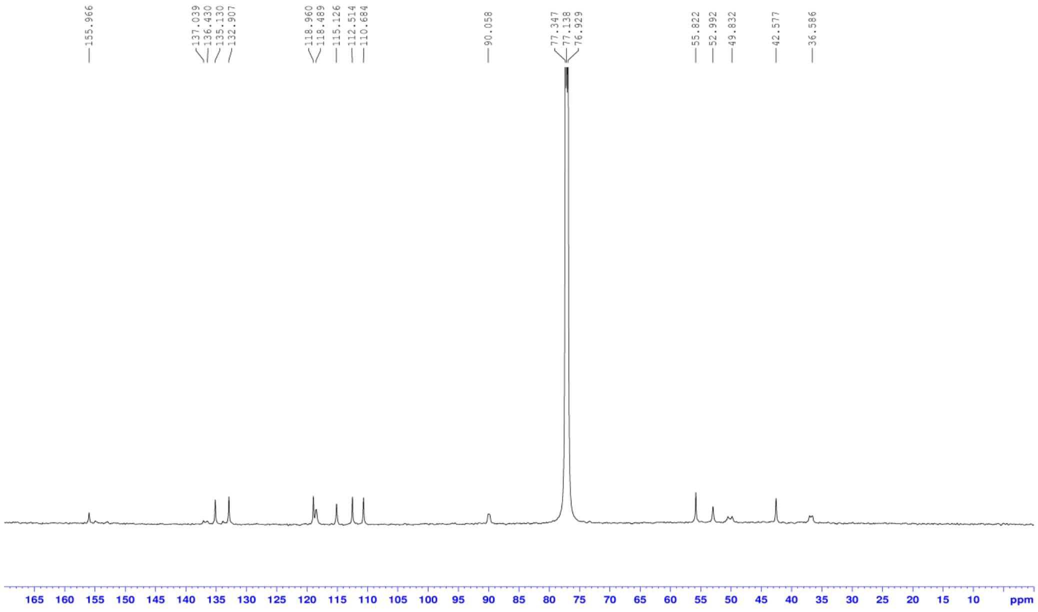

Isobutyl 3,3-diallyl-2-hydroxy-5-methoxyindoline-1-carboxylate (10c)

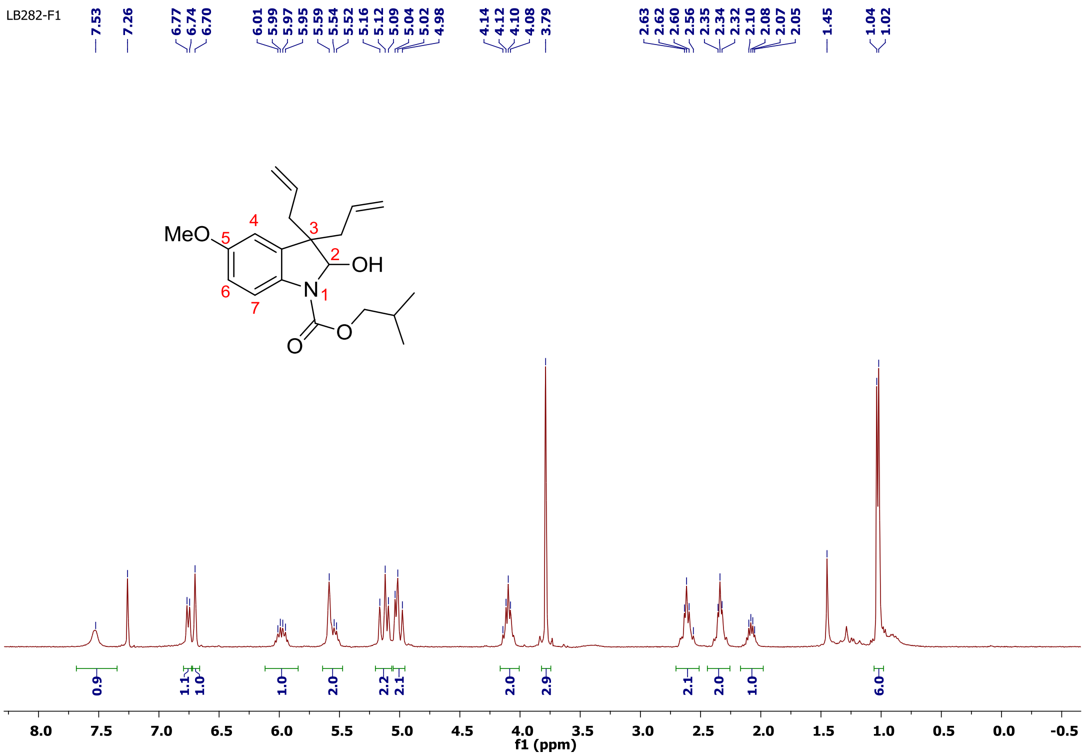

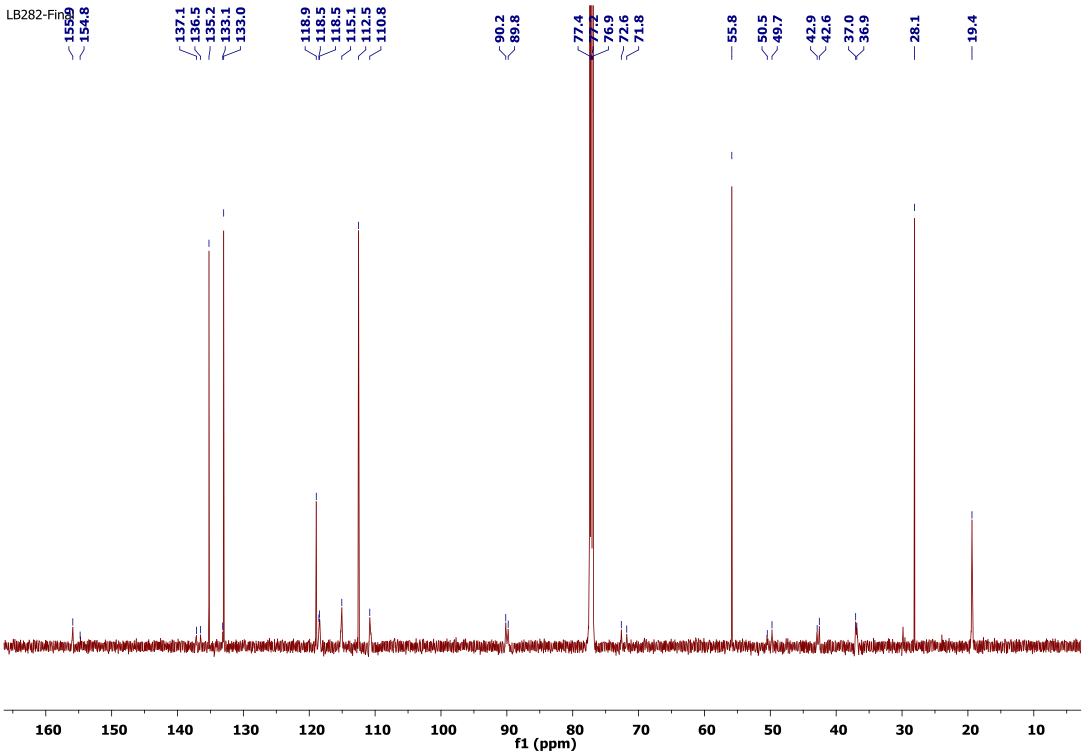

1-(3,3-Diallyl-2-methoxyindolin-1-yl)-2-phenylethanone (11d)

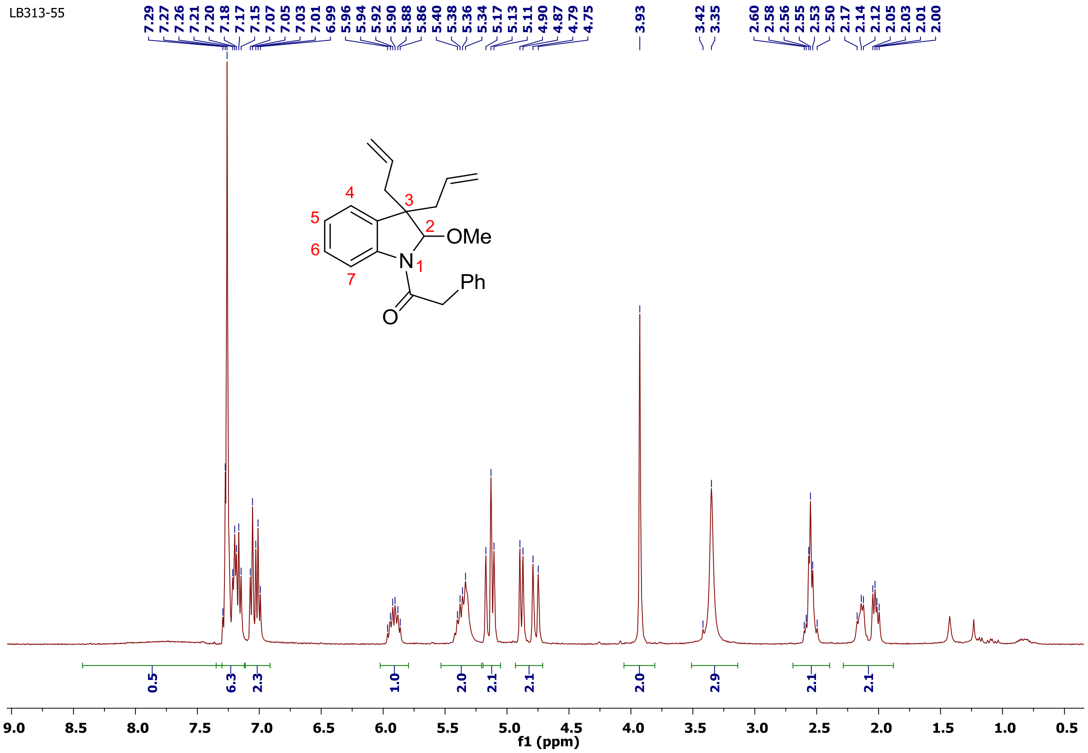

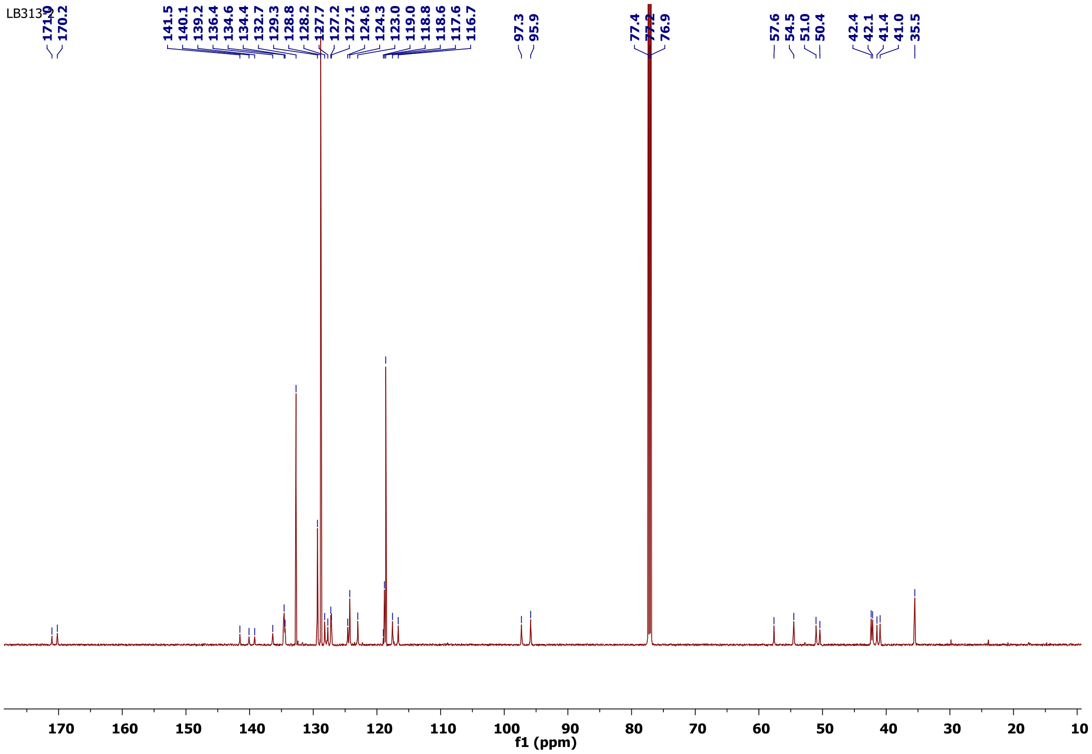

Methyl 3,3-diallyl-2-methyleneindoline-1-carboxylate (12)

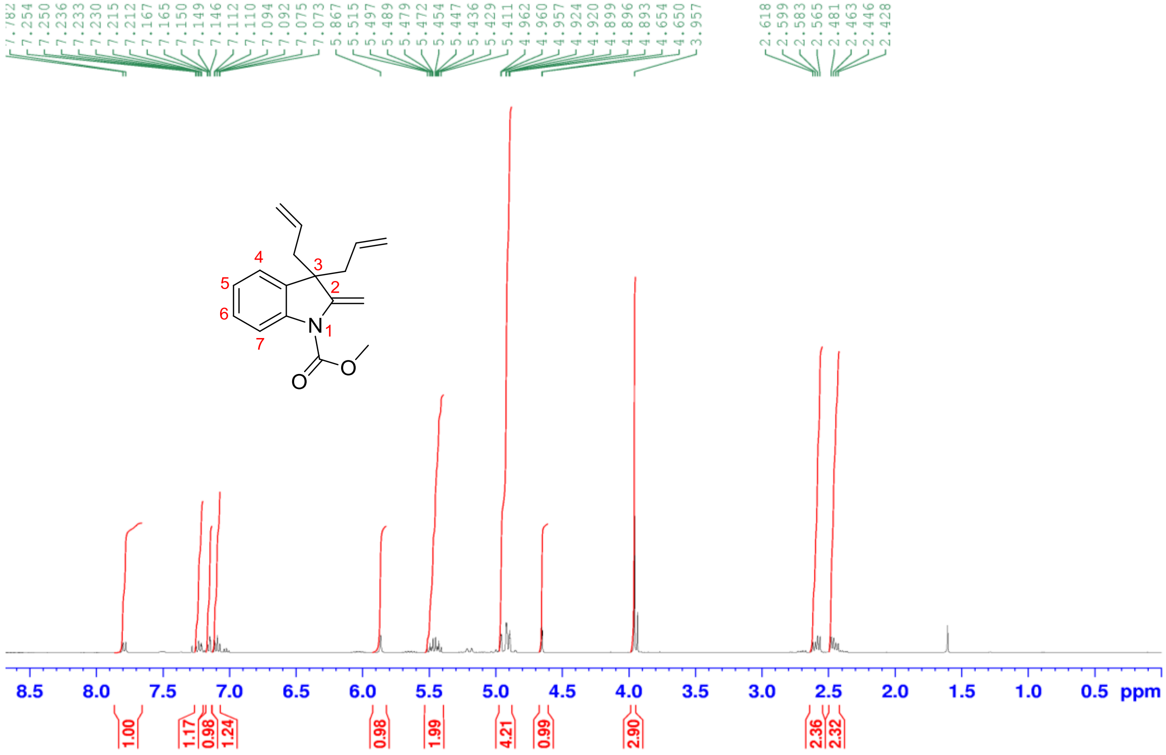

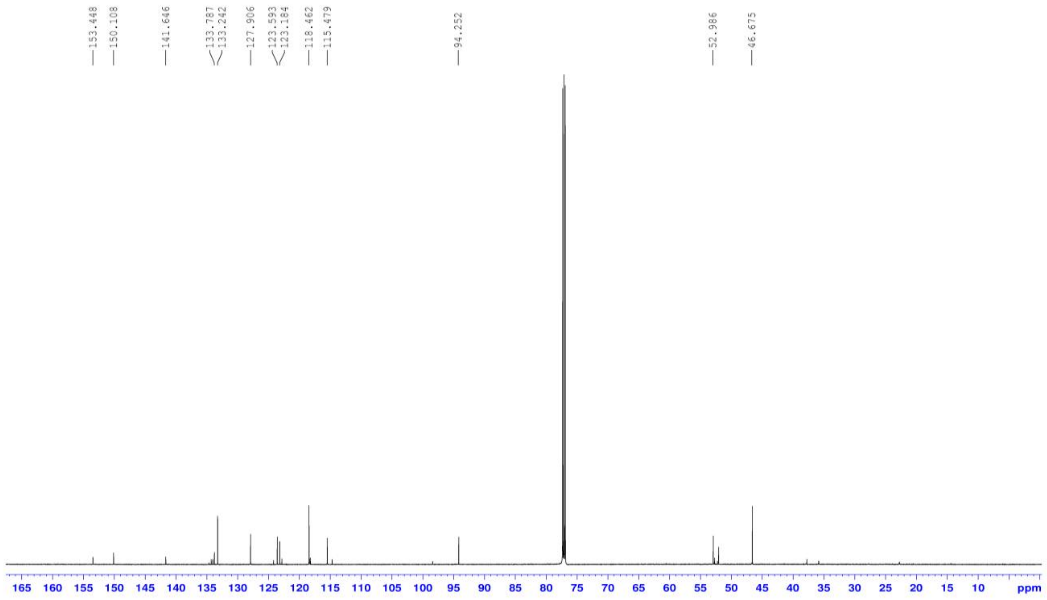

Synthesis of 2,3-diallylindoles

Methyl 2,3-diallyl-1*H*-indole-1-carboxylate (13a)

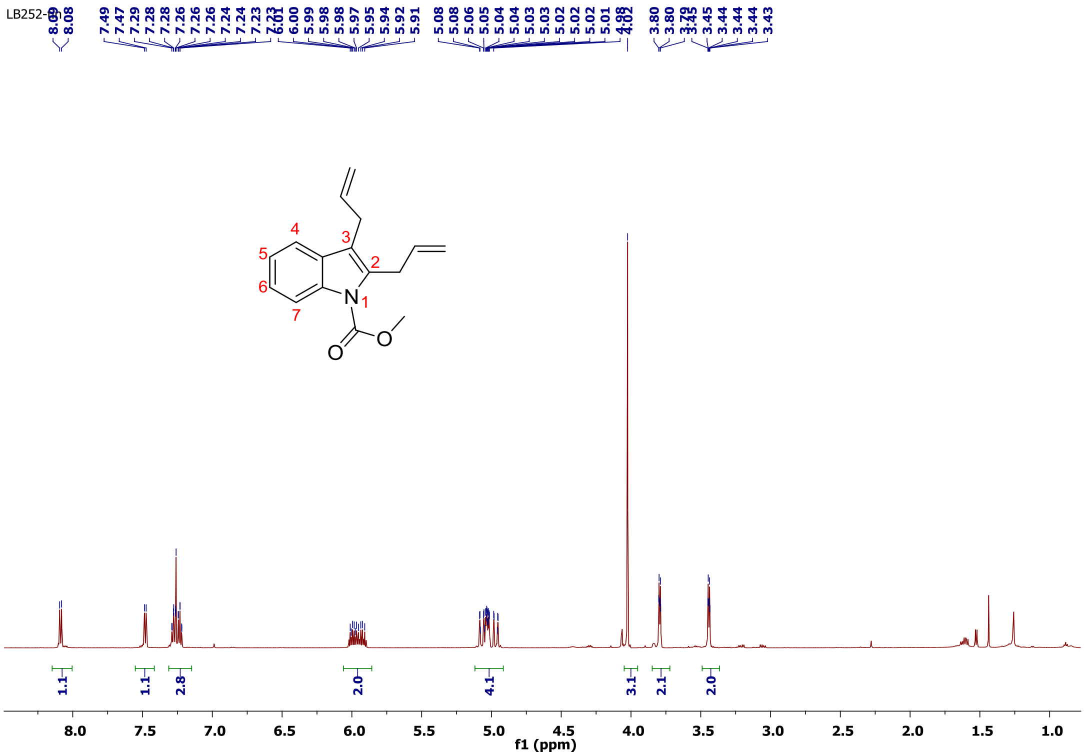

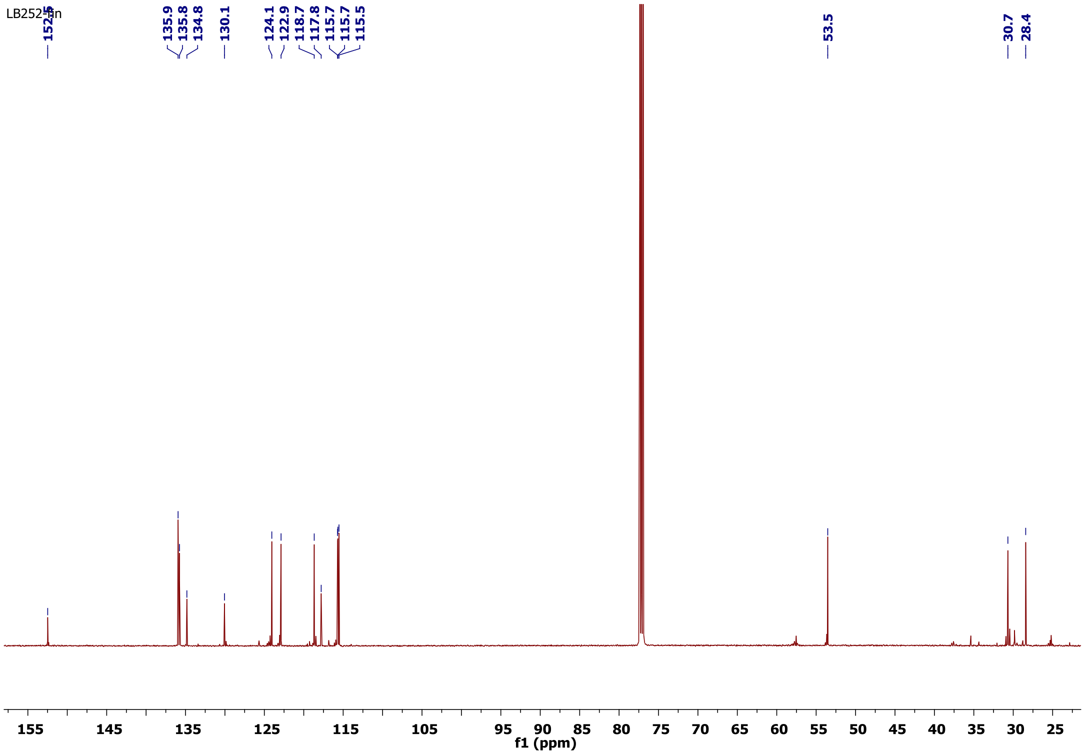

Isobutyl 2,3-diallyl-1*H*-indole-1-carboxylate (13c)

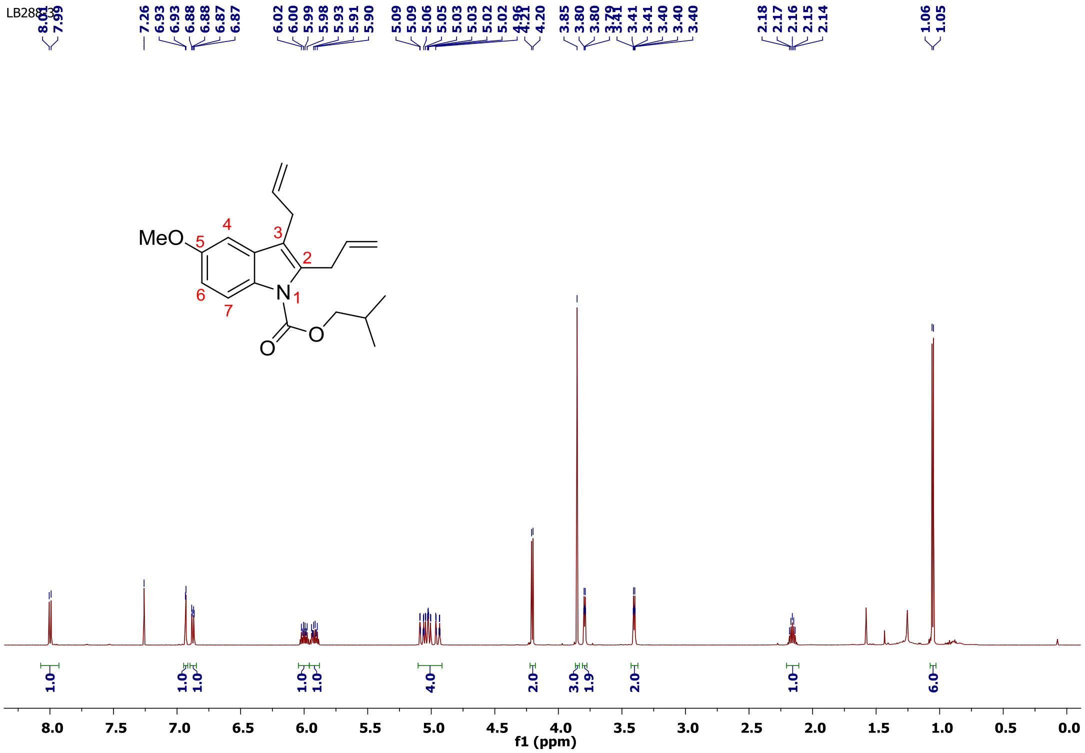

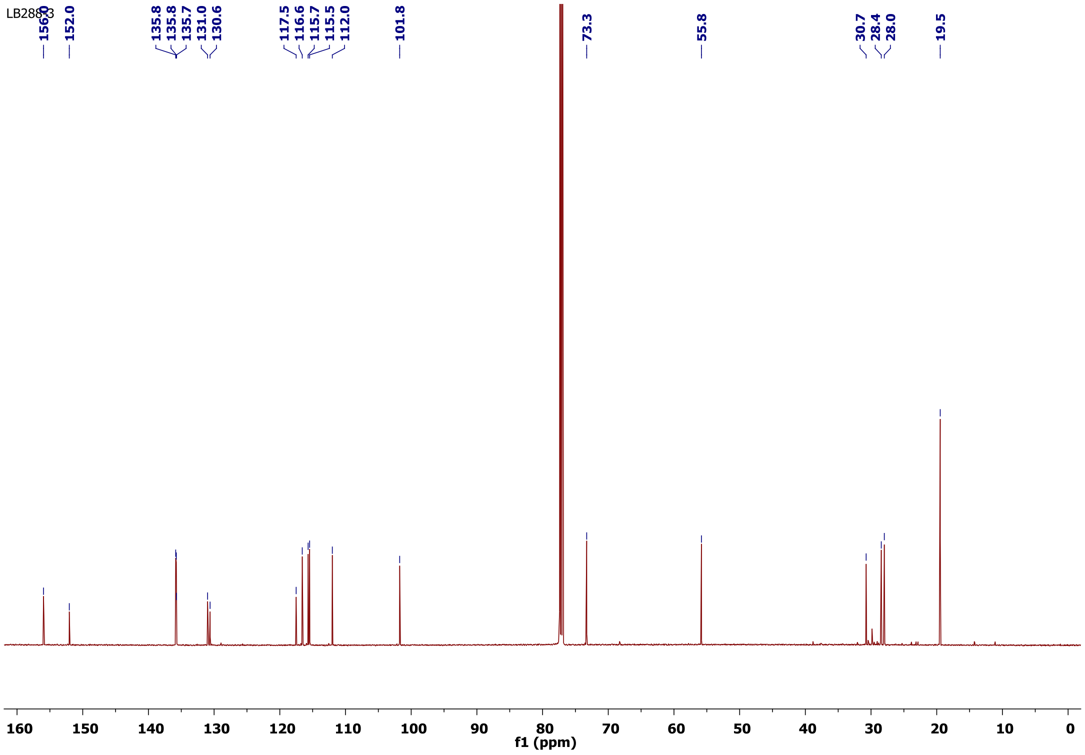

1-(2,3-Diallyl-5-methoxy-1*H*-indol-1-yl)-2-phenylethanone (13d)

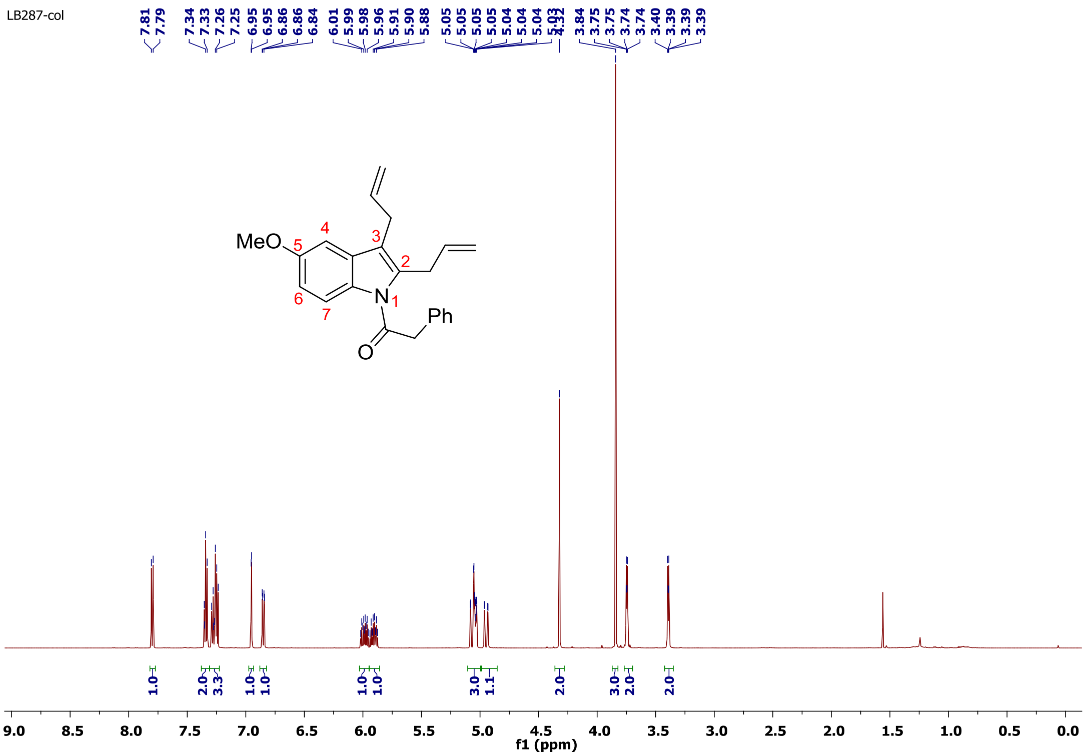

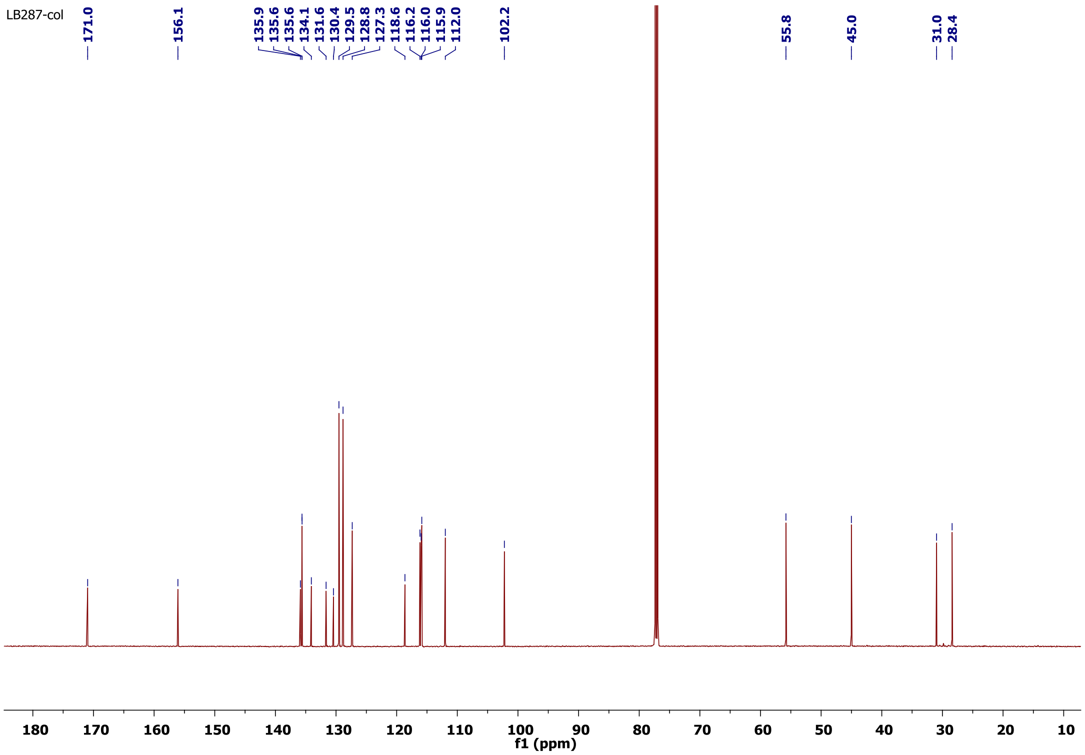

(2,3-Diallyl-1*H*-indol-1-yl)(4-methoxyphenyl)methanone (13e)

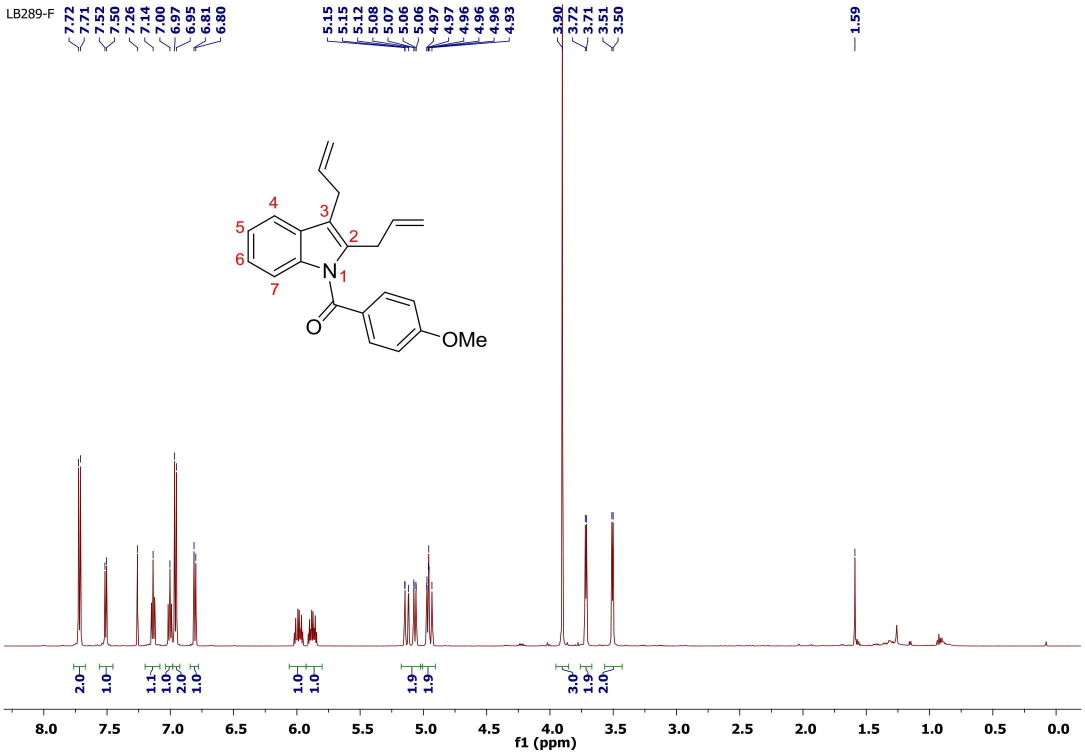

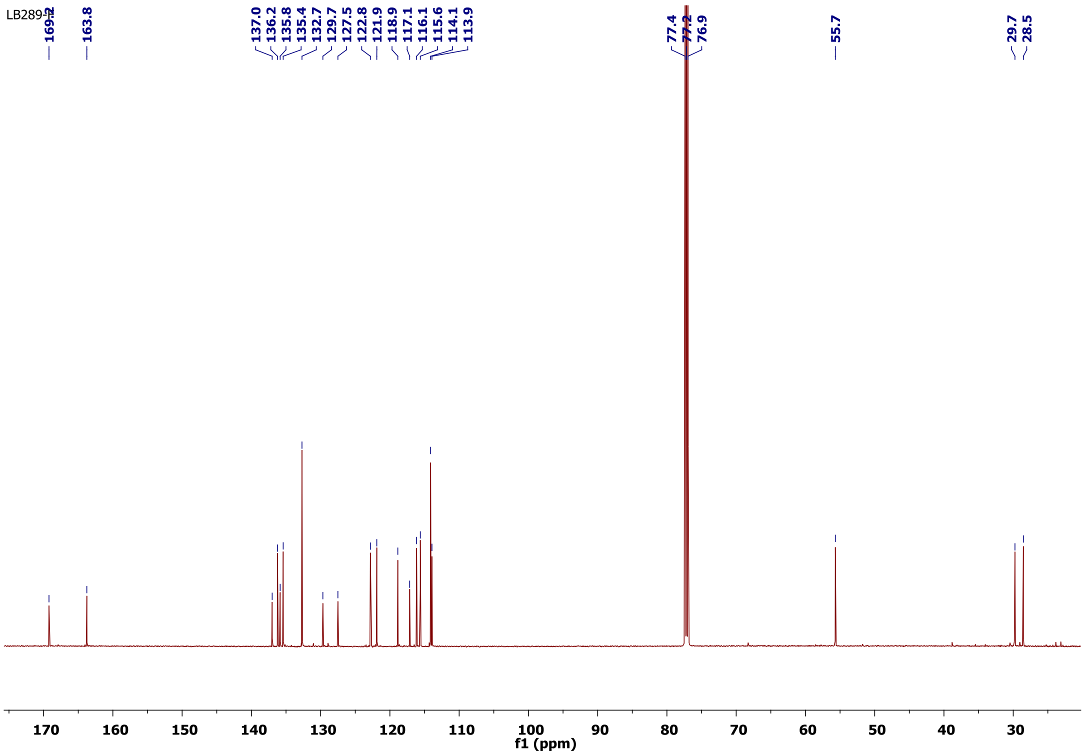

4-Nitrophenyl 2,3-diallyl-5-methyl-1*H*-indole-1-carboxylate (13f)

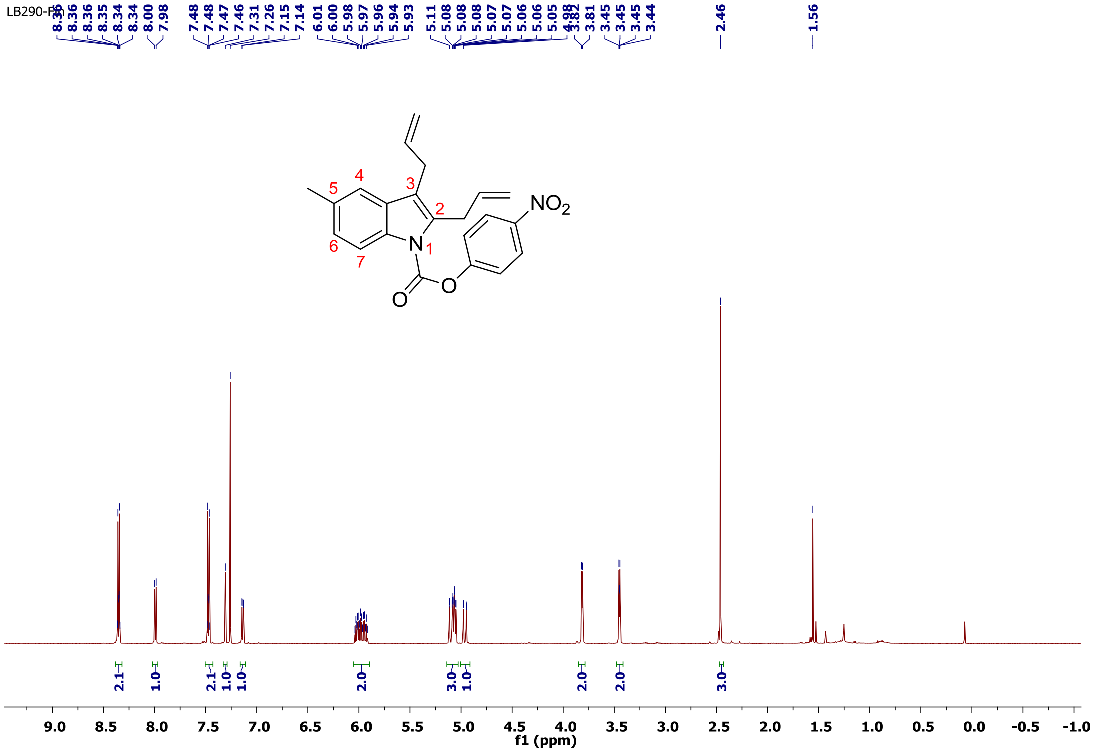

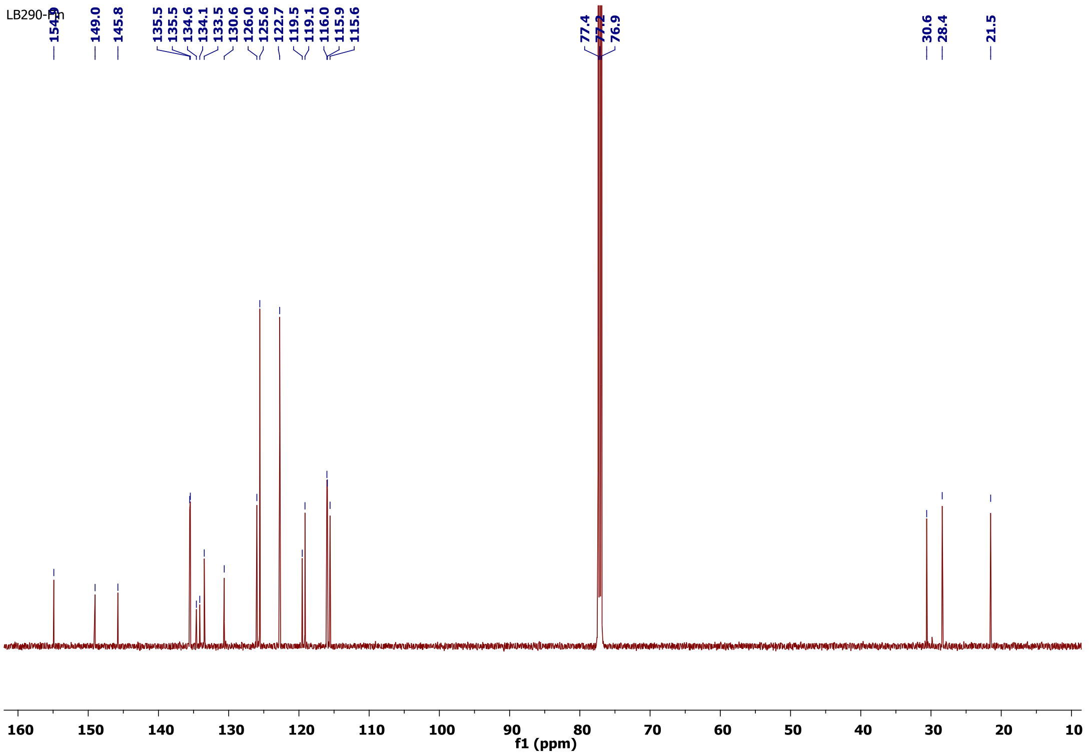

1-(3,3-Diallylindolin-2-yl)propan-2-one (14a)

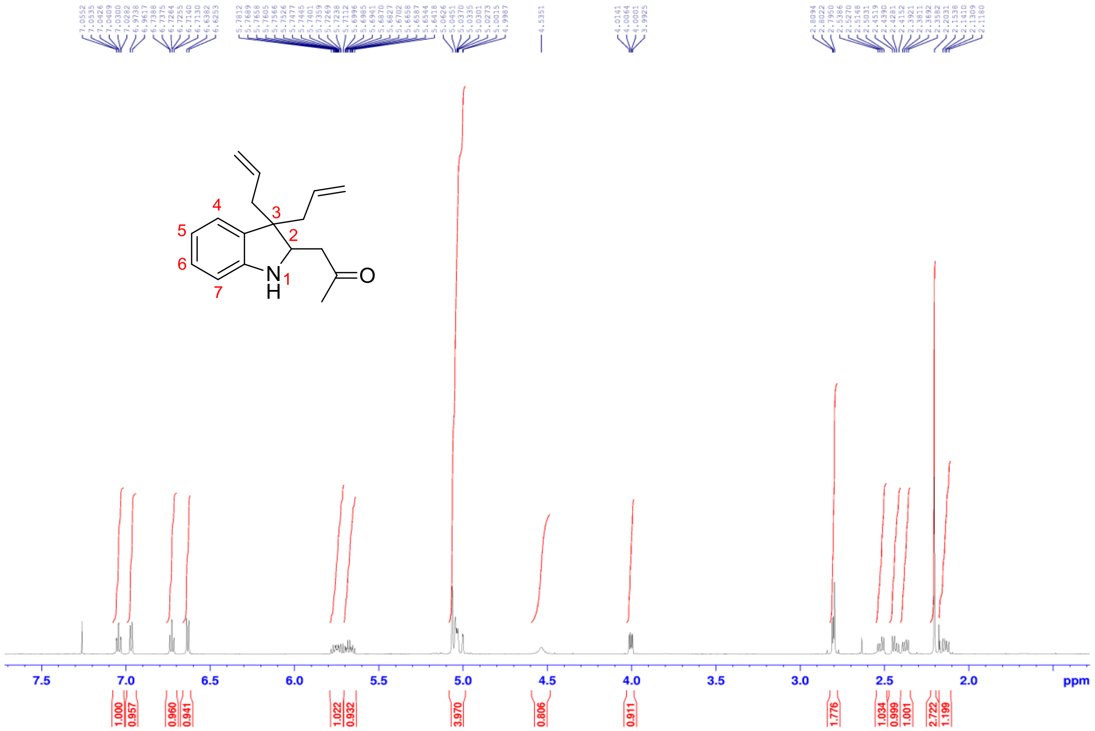

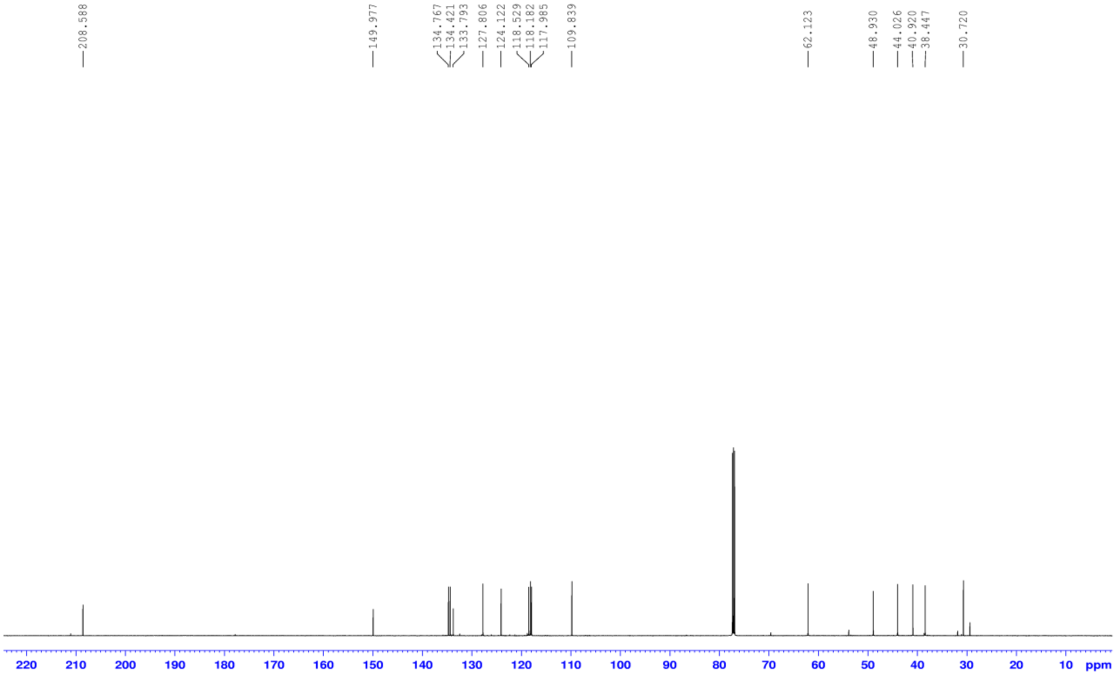

1-(3,3-Diallyl-5-methoxyindolin-2-yl)propan-2-one (14b)

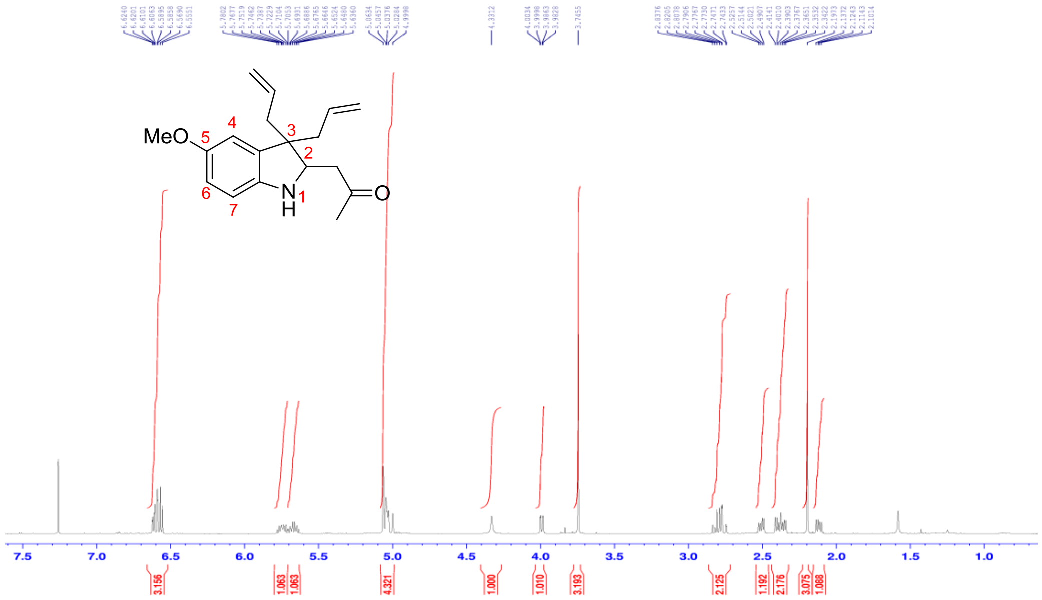

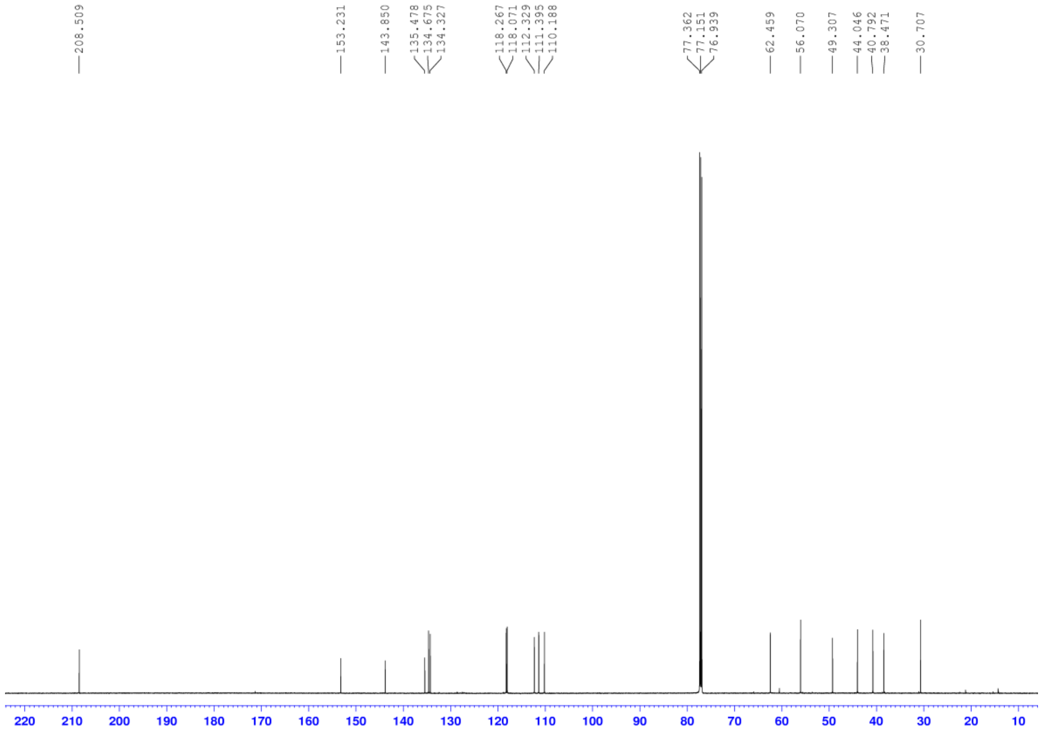

1-(3,3-Diallyl-5-chloroindolin-2-yl)propan-2-one (14c)

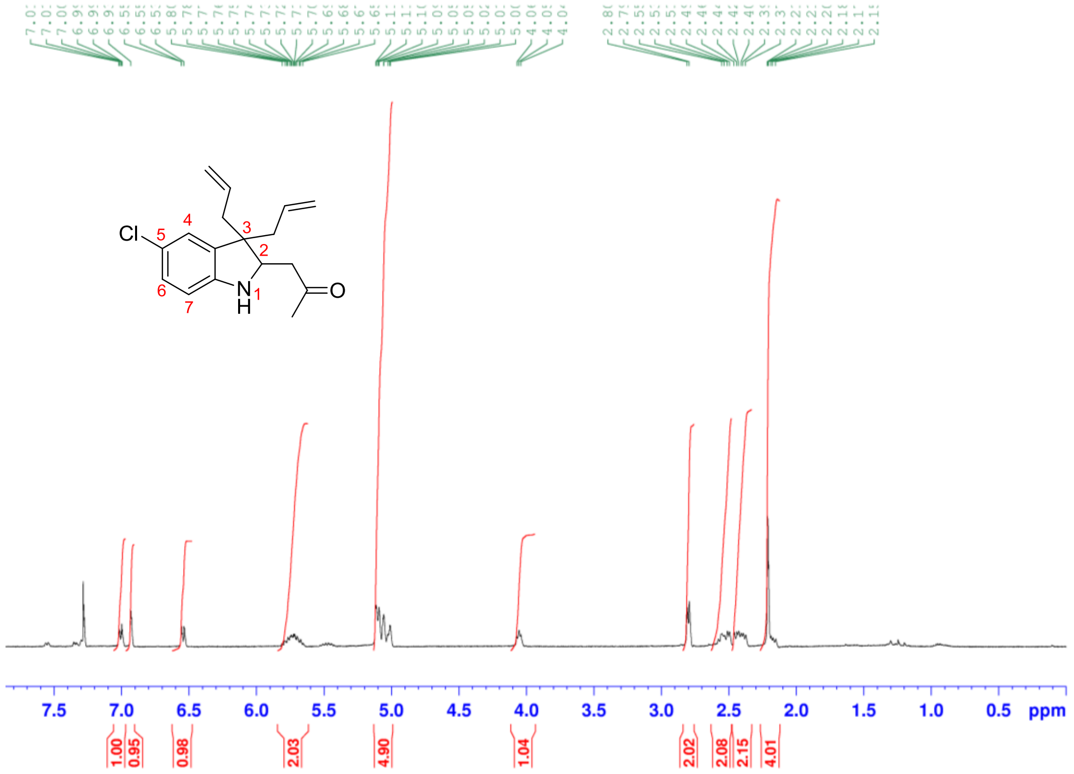

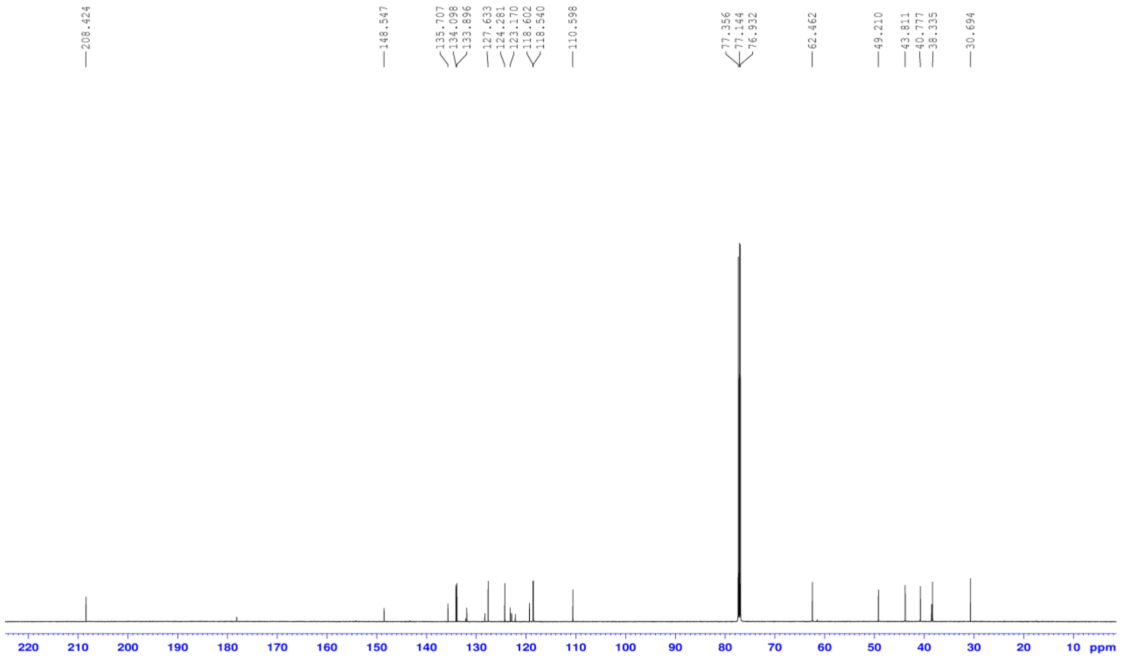

Ring closing metathesis reaction on UGI compounds

1'-Benzoyl-*N*-(*tert*-butyl)spiro[cyclopentane-1,3'-indolin]-3-ene-2'-carboxamide(15a)

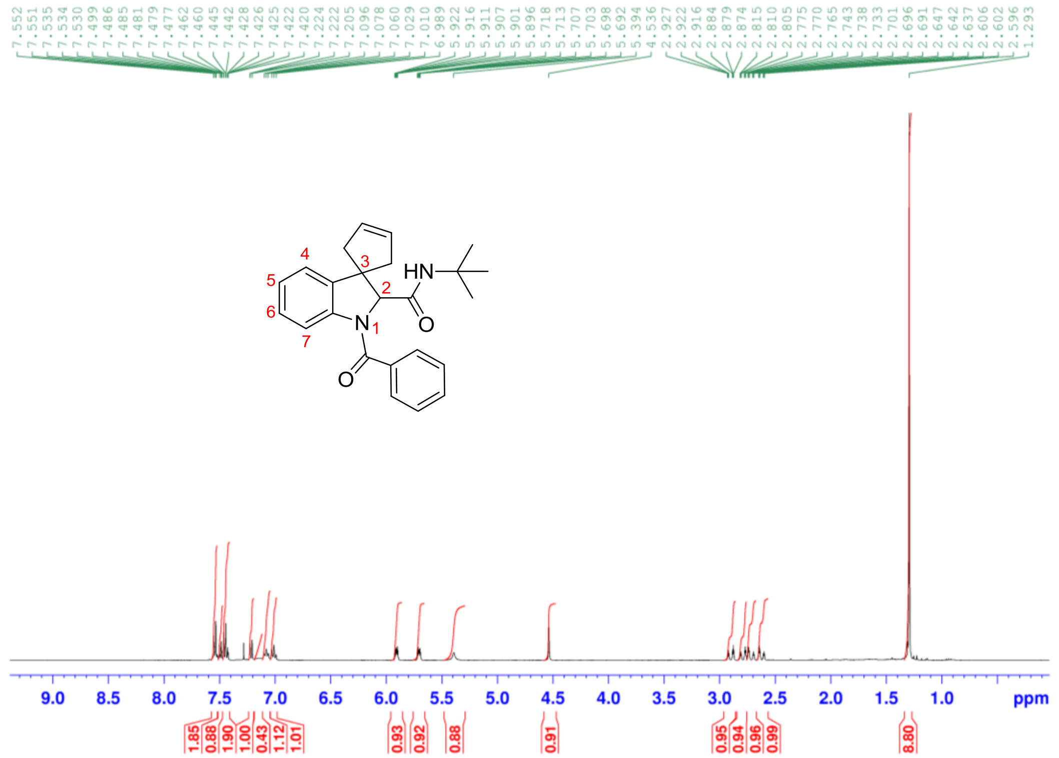

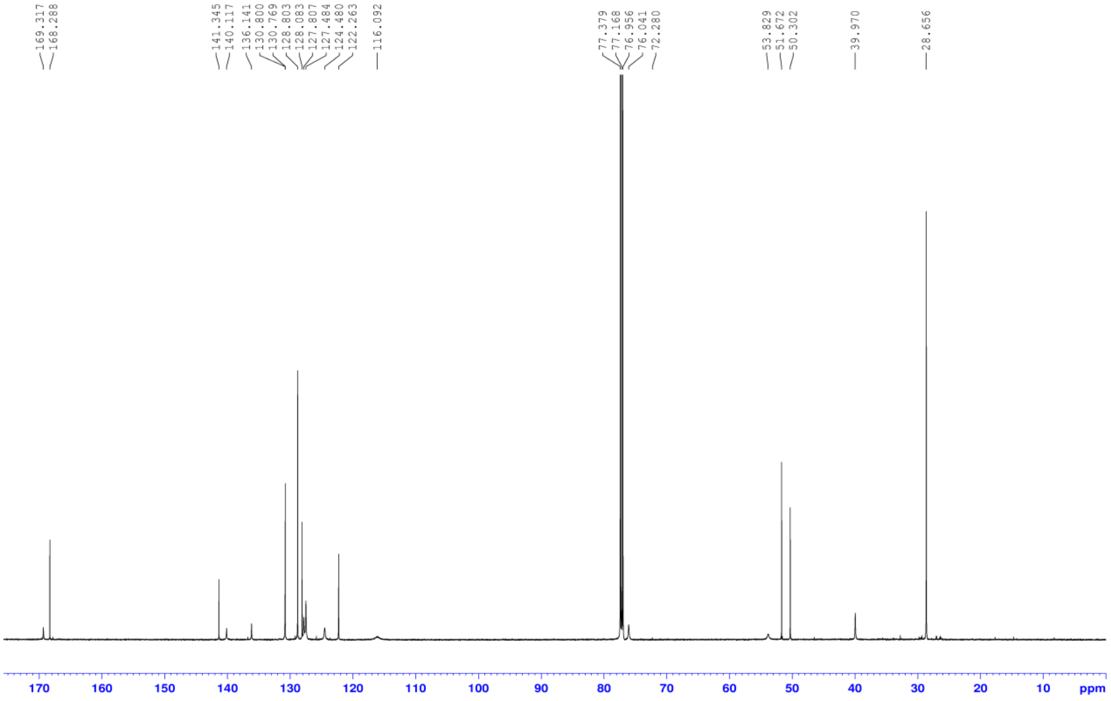

1'-Benzoyl-*N*-cyclohexylspiro[cyclopentane-1,3'-indolin]-3-ene-2'-carboxamide (15b)

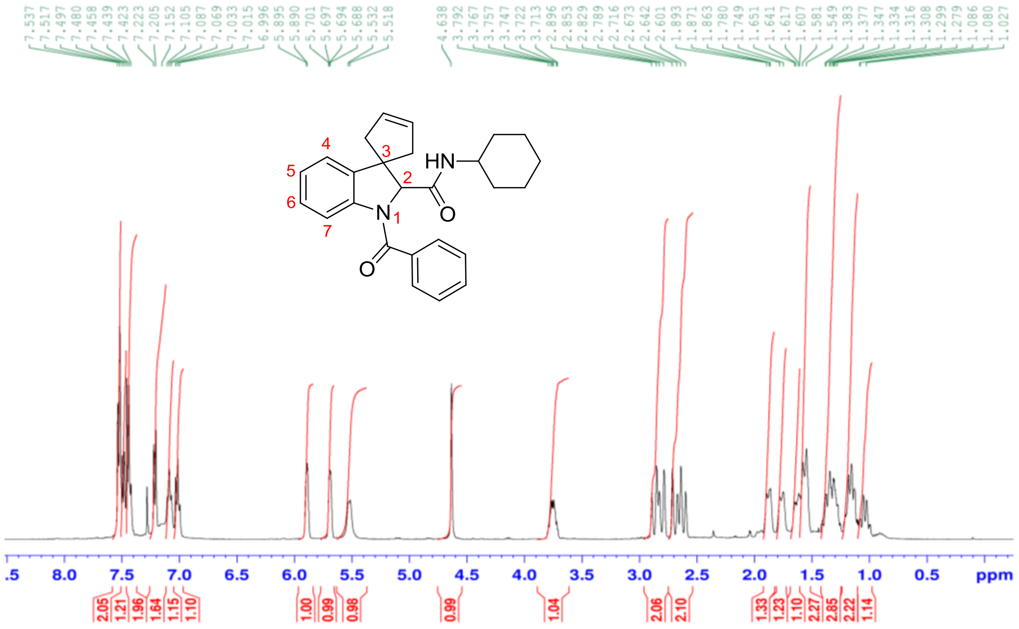

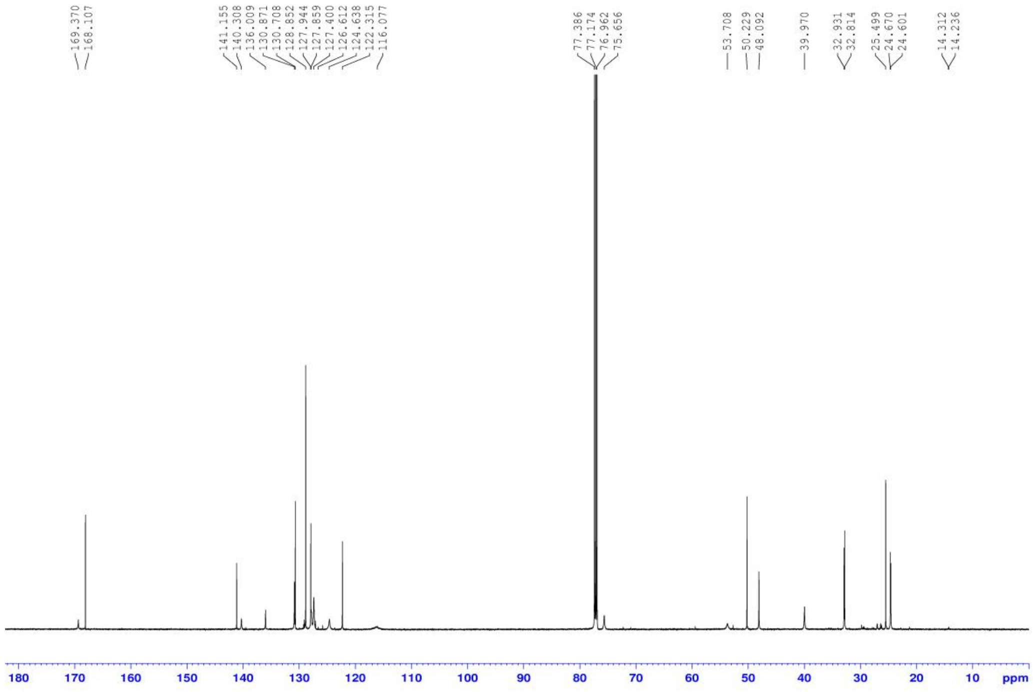

1'-Benzoyl-*N*-(*tert*-butyl)-5',6'-dimethoxyspiro[cyclopentane-1,3'-indolin]-3-ene-2'-carboxamide (15c)

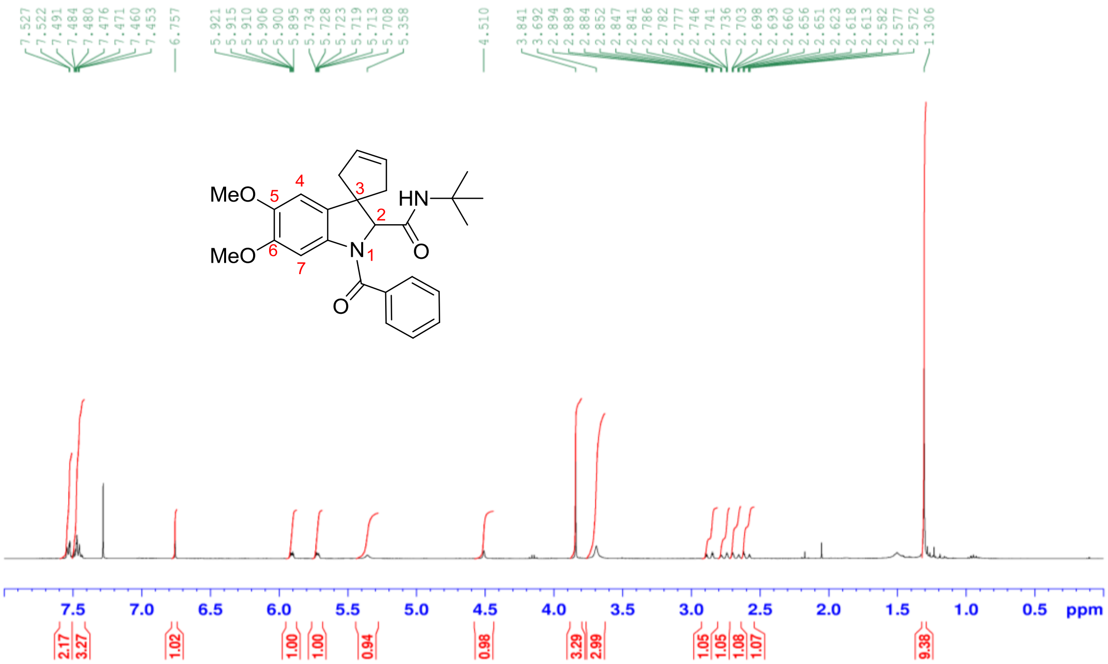

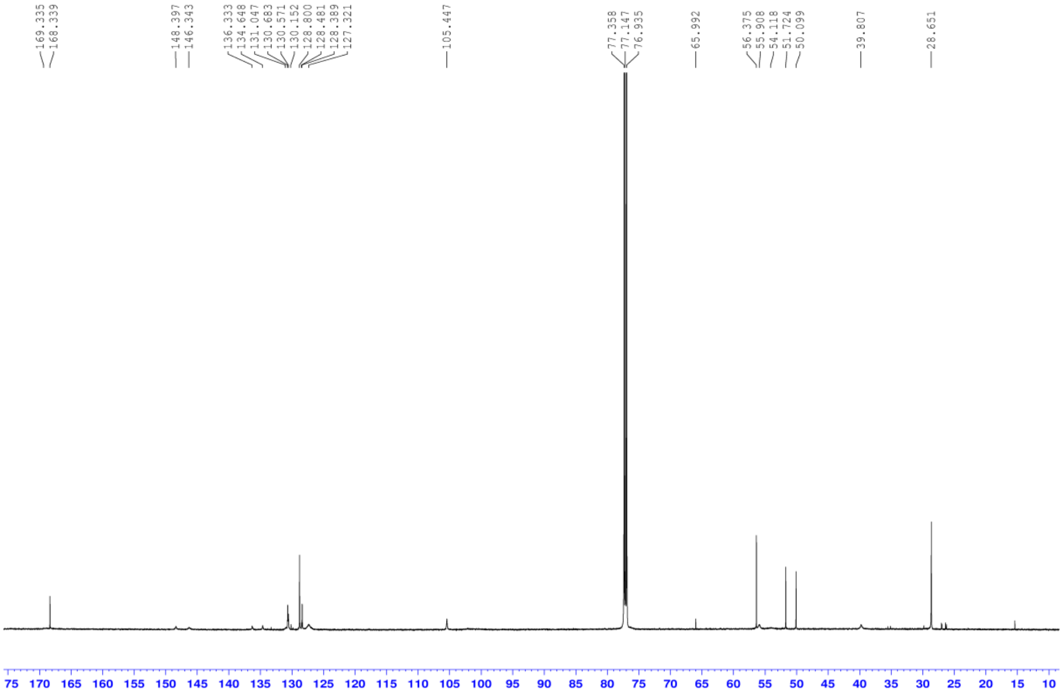

***tert*-Butyl (2-(2'-(*tert*-butylcarbamoyl)-5'-chlorospiro[cyclopentane-1,3'-indolin]-3-en-1'-yl)-2-oxoethyl)carbamate (15j)**

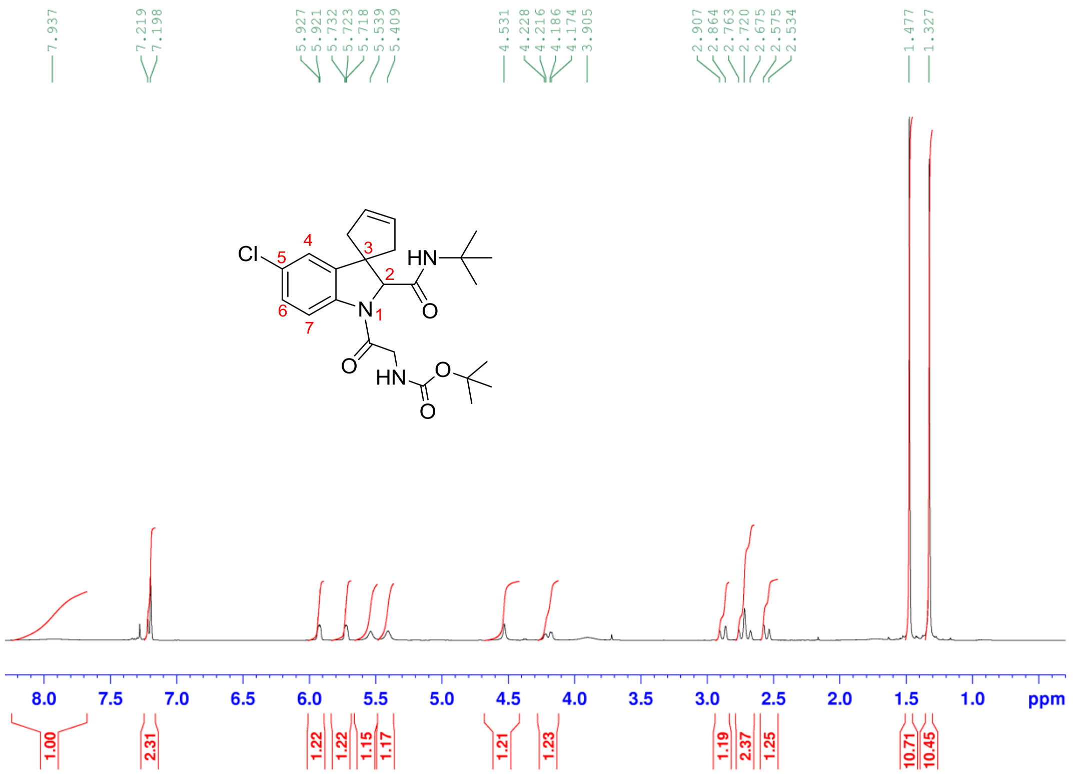

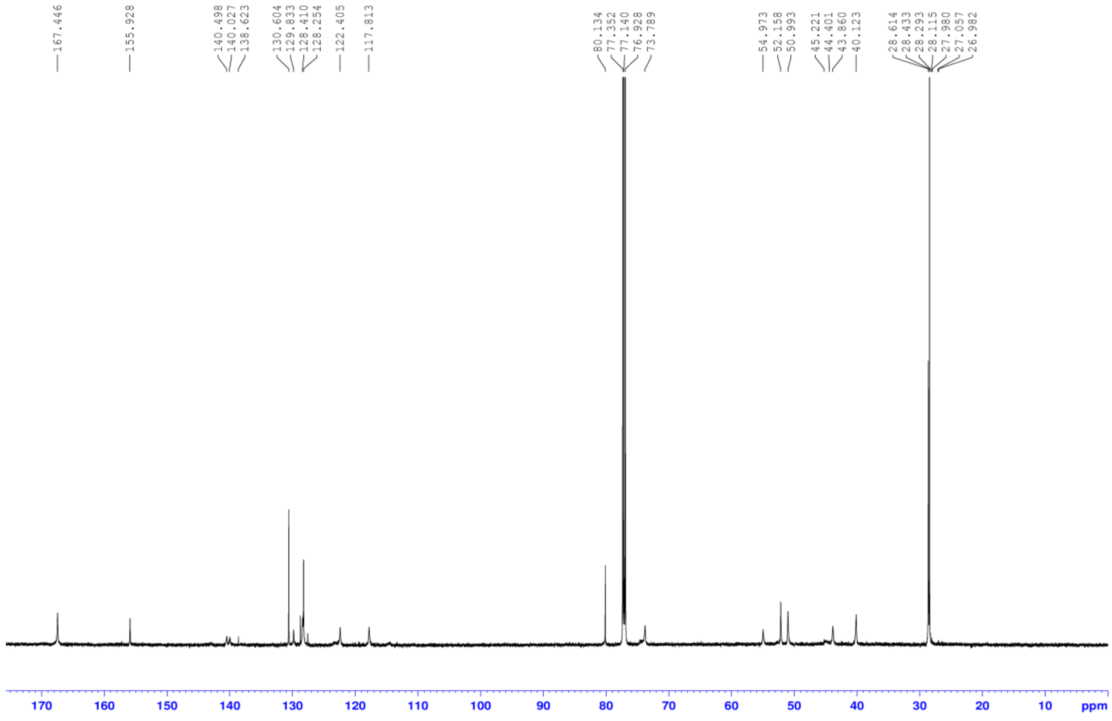

***tert*-Butyl(2-(2'-(*tert*-butylcarbamoyl)spiro[cyclopent[3]ene-1,3'-indolin]-1'-yl)-2-oxoethyl)carbamate (15k)**

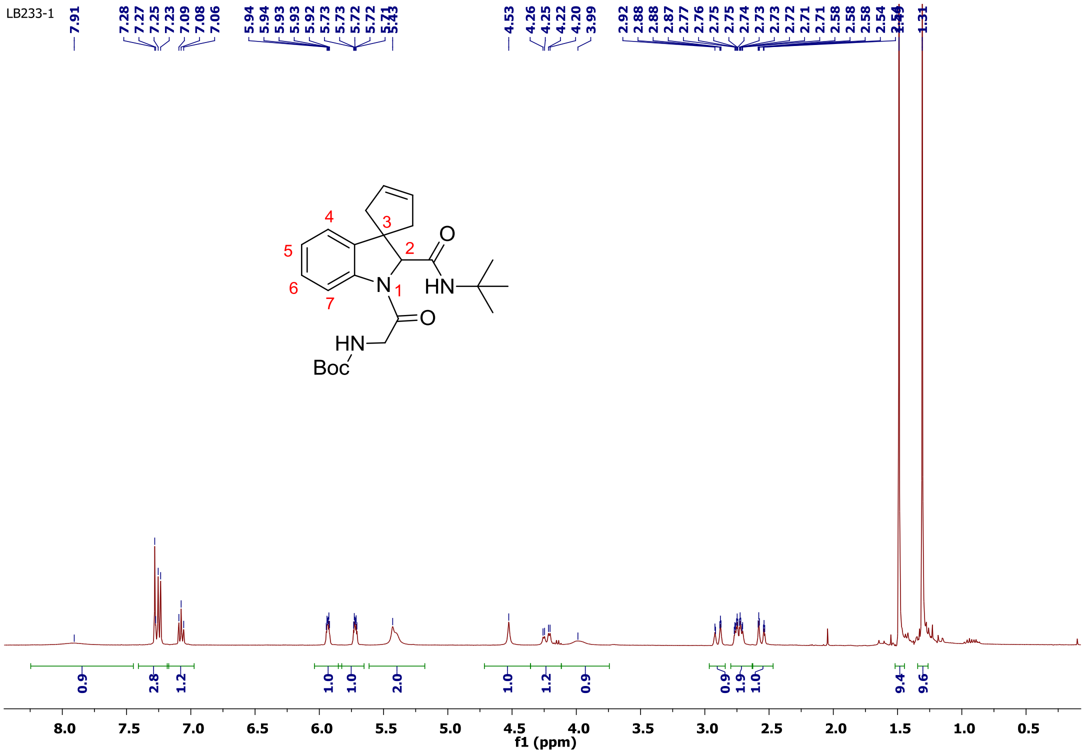

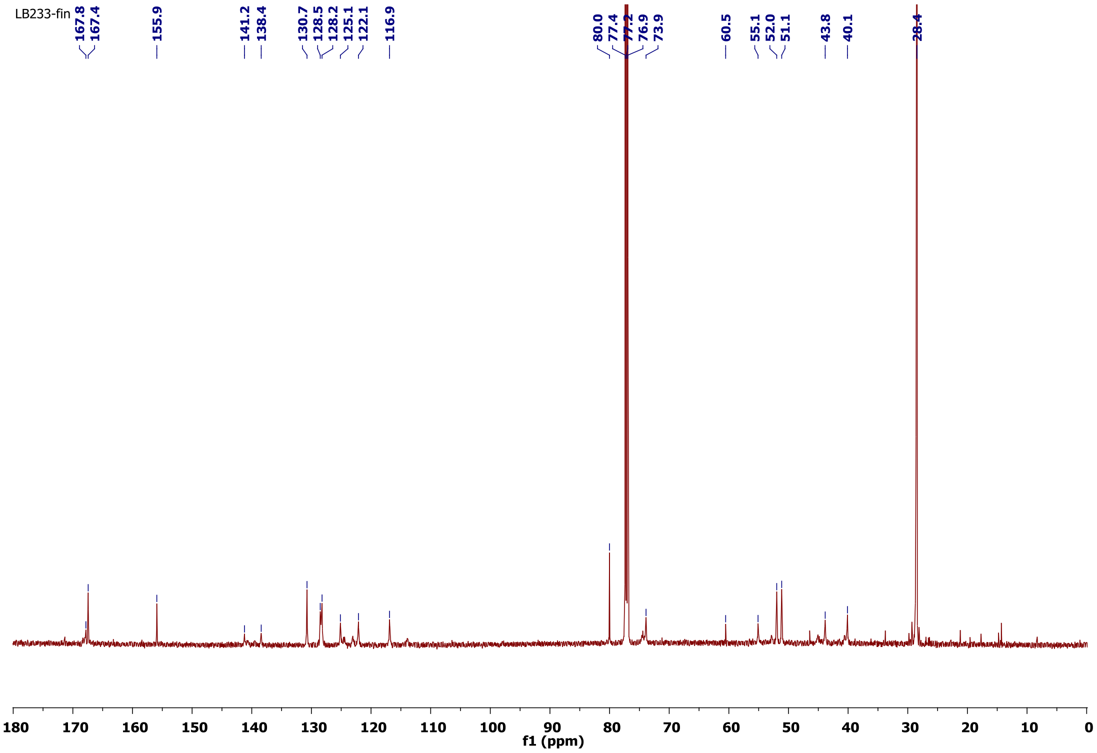

_N_-(_tert_-butyl)-1'-((R)-2-hydroxy-2-phenylacetyl)spiro[cyclopent[3]ene-1,3'-indoline]-2'-carboxamide (15n)

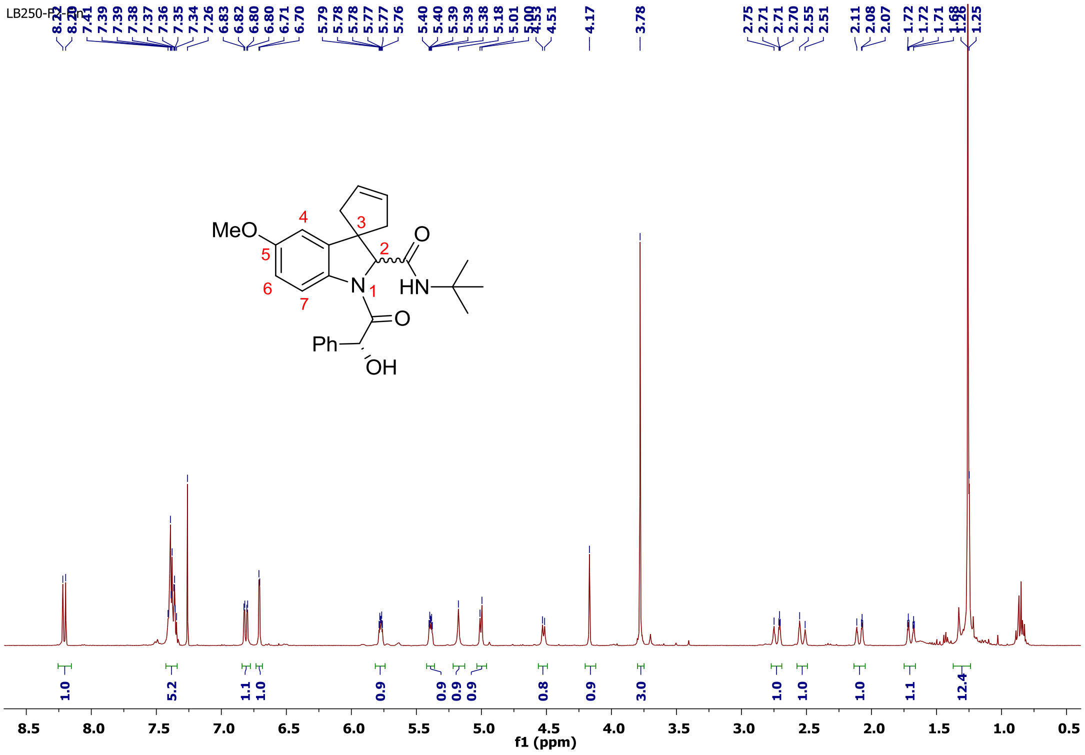

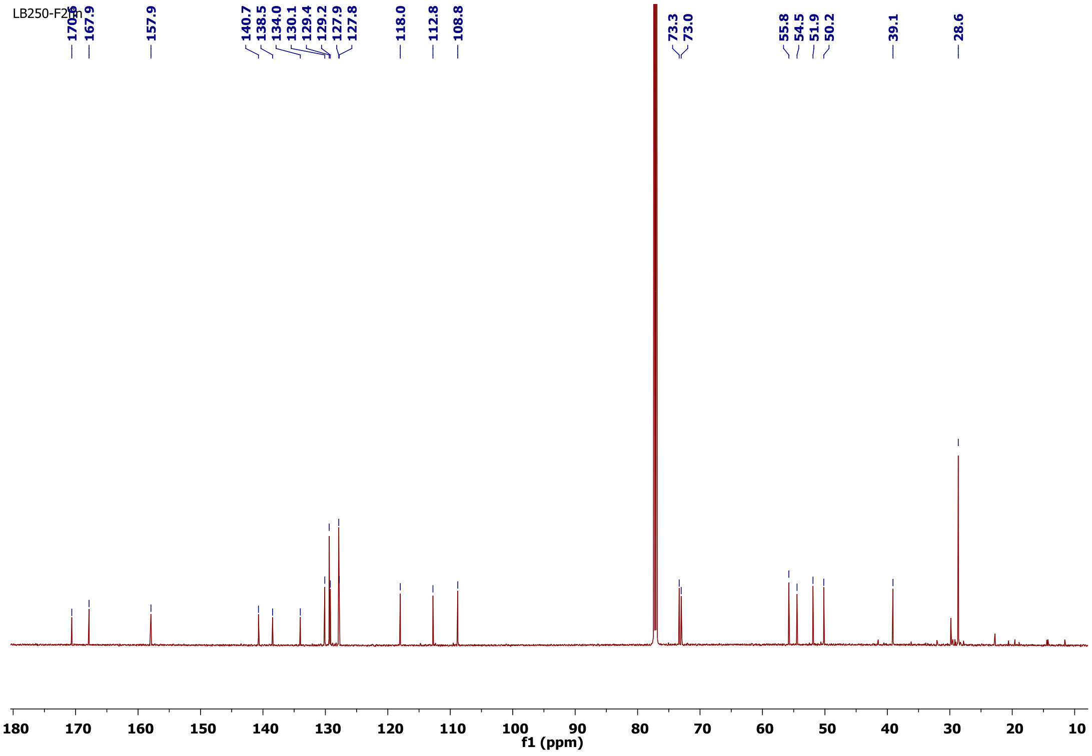

Ring closing metathesis reaction on substituted 3,3-diallyl-2-hydroxyindoline

Methyl 2'-hydroxyspiro[cyclopentane-1,3'-indolin]-3-ene-1'-carboxylate (16a)

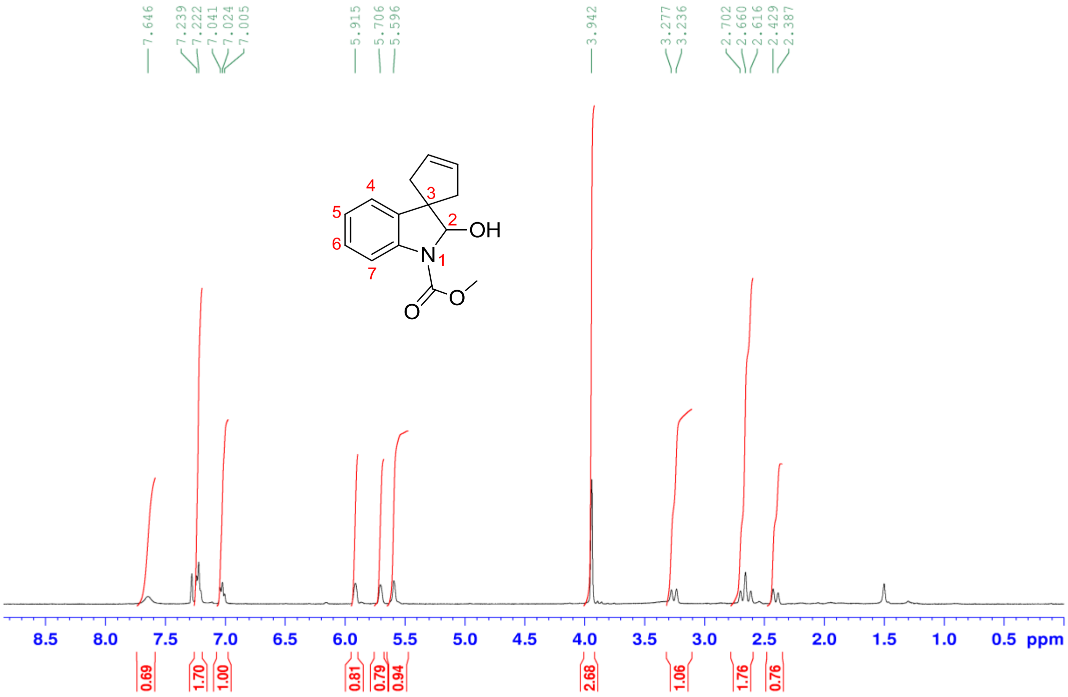

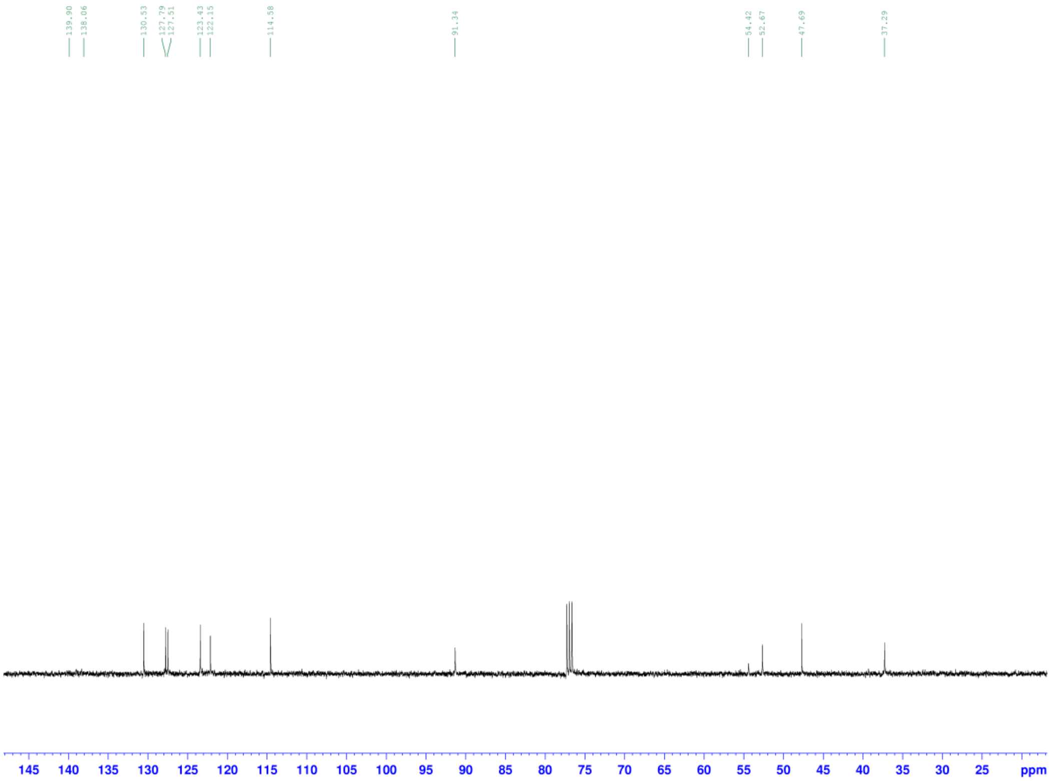

Methyl-2'-hydroxy-5'-methoxyspiro[cyclopentane-1,3'-indolin]-3-ene-1'-carboxylate (16b)

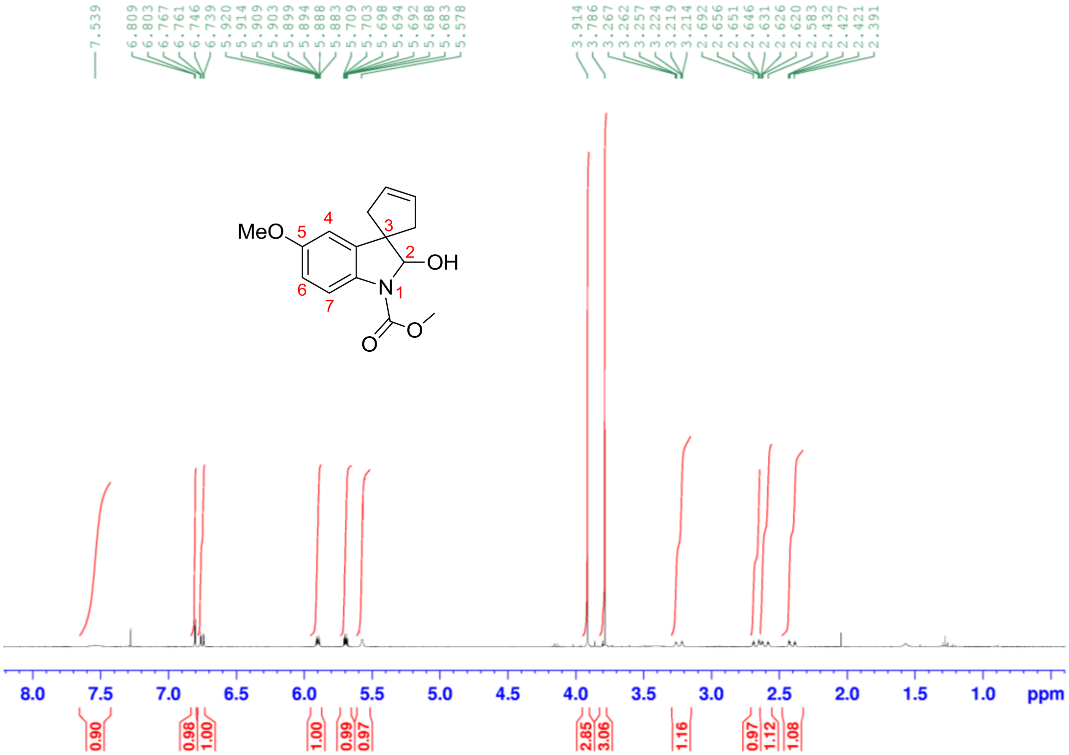

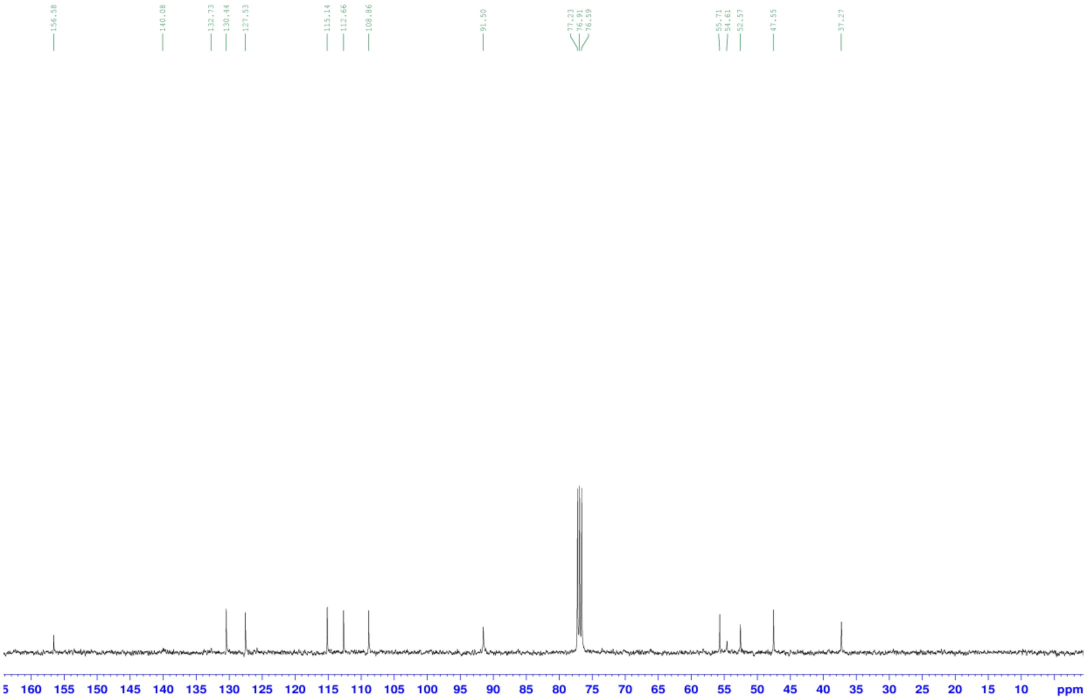

Synthesis of dihydro-1H-carbazole by ring closing metathesis

1-(6-Methoxy-1*H*-carbazol-9(4*H*)-yl)-2-phenylethanone (18d)

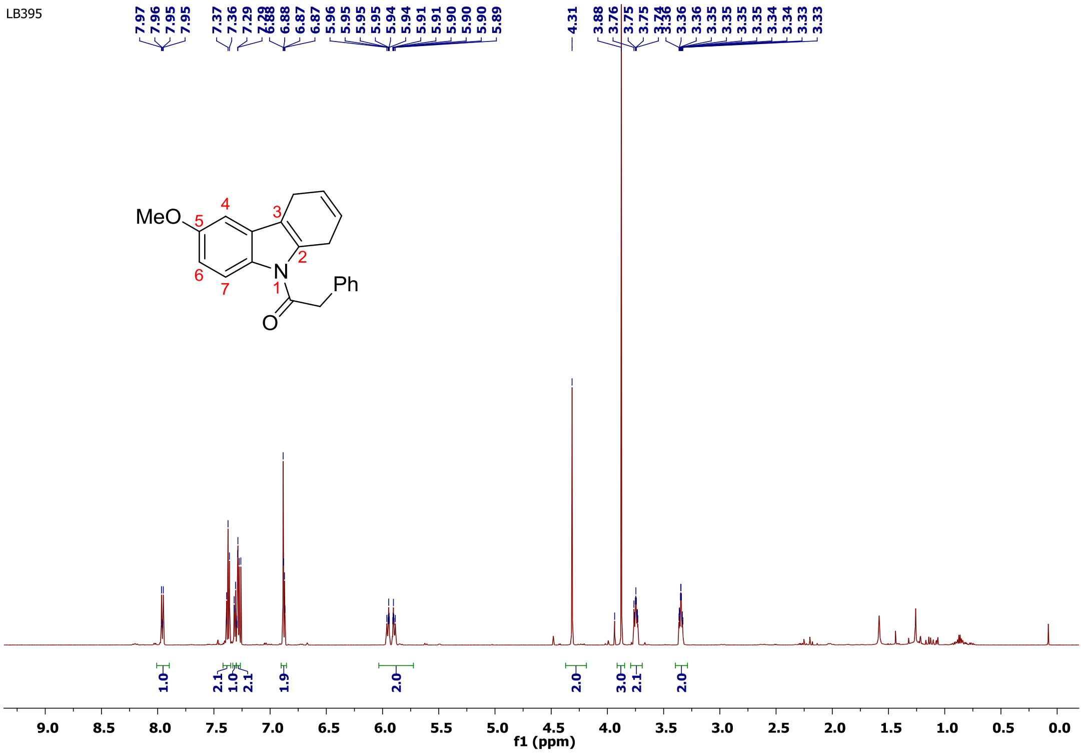

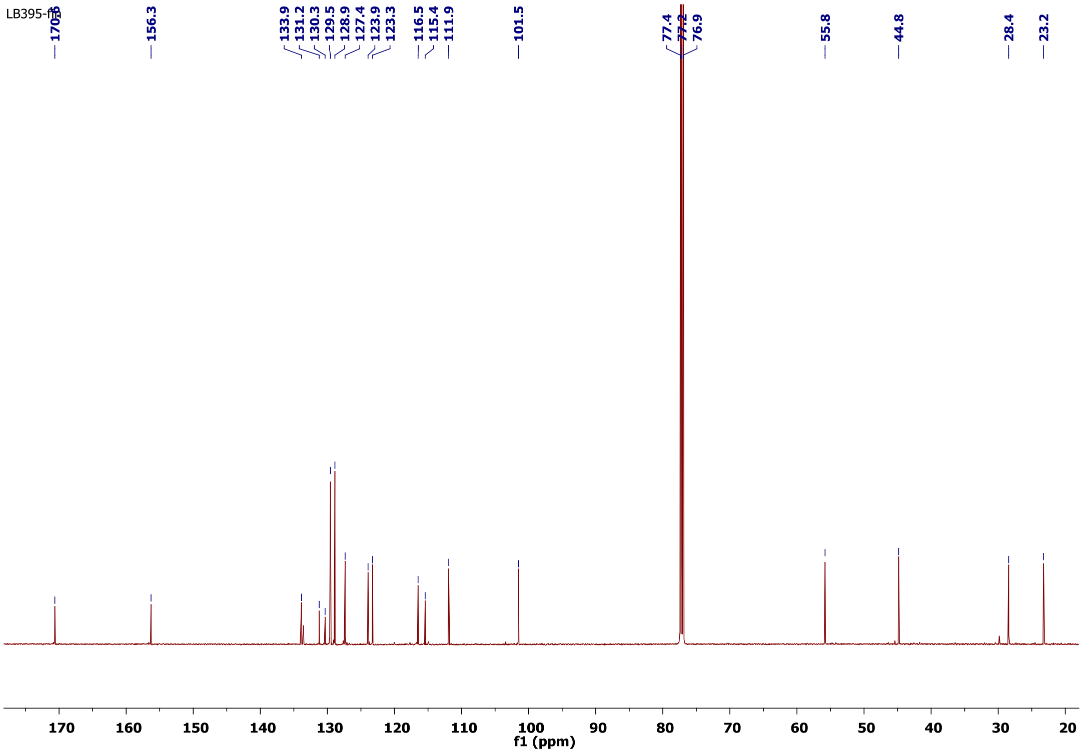

(L)-Proline catalysed asymmetric Mannich reaction: HPLC data

Solvent screening

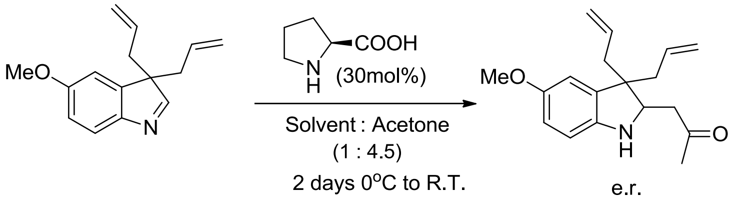

Solvents	Retention time (tr, minutes) minor/major *	E.R.
**DMSO**	15.86 : 17.51	98.7 : 1.3
**$CHCl_3$**	16.07: 17.87	99.1 : 0.9
**MeCN**	15.94 : 17.78	99.7: 0.4
**MeOH**	16.12 : 18.01	54.6: 45.4
**DMF**	16.10 : 17.92	98.9 : 1.1

*Chiralpak Daicel AD // hexane/ *i*PrOH (75/25) // 0.5 mL/min

Substrate scope

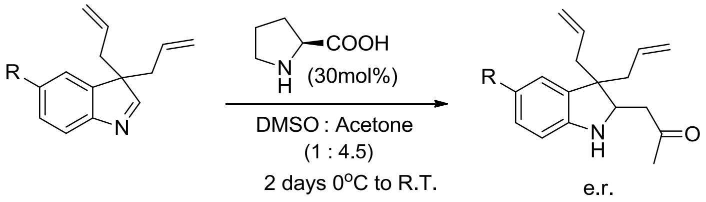

R	Retention time (tr, minutes) minor/major *	E.R.	Yield
**OMe**	15.12: 16.70	98.8 : 1.2	96
**Cl**	11.56 : 13.14	99.0: 1.0	64[a]
**H**	16.71: 21.45	99.3: 0.7	81

*Chiralpak Daicel AD // hexane/ *i*PrOH (75/25) // 0.5 mL/min

Chiral HPLC chromatograms of Mannich products

(Rac)-1-(3,3-Diallyl-5-methoxyindolin-2-yl)propan-2-one

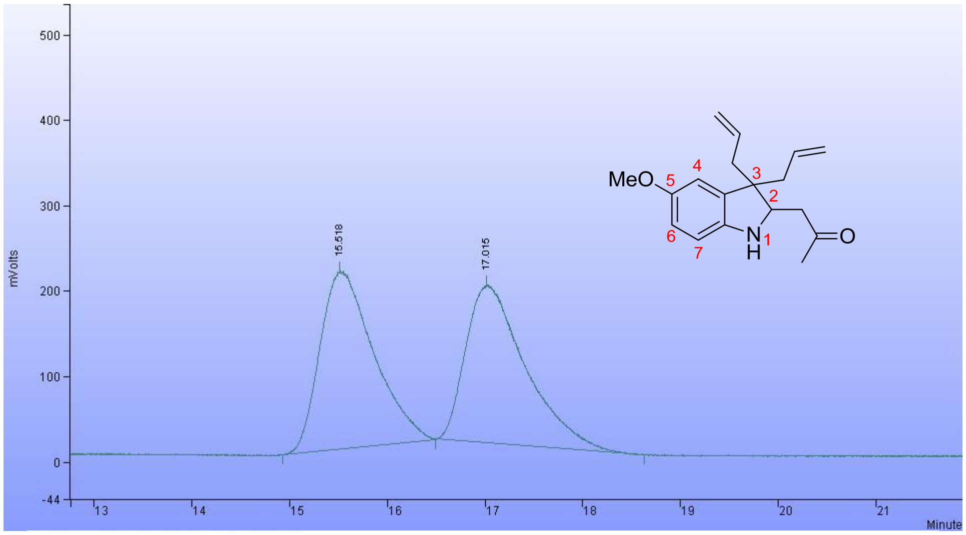

Peak No.	Peak Name	Result ()	Ret. Time (min)	Time Offset (min)	Area (counts)	Sep. Code	Width 1/2 (sec)	Status Codes
1		50.4550	15.518	0.000	7925187	BB	37.0	
2		49.5450	17.015	0.000	7782256	BB	36.8	
	Totals:	100.0000		0.000	15707443			

Enantioenriched 1-(3,3-Diallyl-5-methoxyindolin-2-yl)propan-2-one from asymmetric Mannich reaction

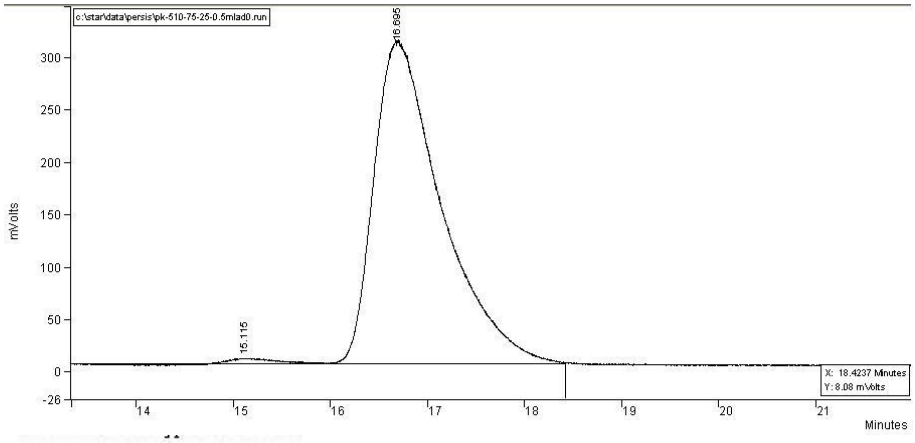

Peak No.	Peak Name	Result ()	Ret. Time (min)	Time Offset (min)	Area (counts)	Sep. Code	Width 1/2 (sec)	Status Codes
1		1.2183	15.115	0.000	184044	BB	32.8	
2		98.7817	16.695	0.000	14922880	BB	42.3	
	Totals:	100.0000		0.000	15106924			

(Rac)-1-(3,3-Diallyl-5-chloroindolin-2-yl)propan-2-one

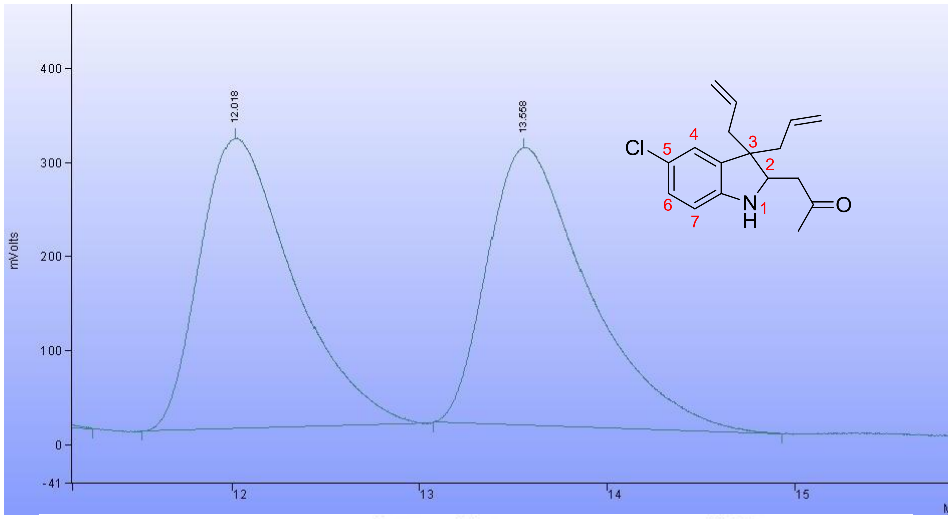

Peak No.	Peak Name	Result ()	Ret. Time (min)	Time Offset (min)	Area (counts)	Sep. Code	Width 1/2 (sec)	Status Codes
1		48.9942	12.018	0.000	10654493	BB	32.0	
2		51.0058	13.558	0.000	11091961	BB	33.0	
	Totals:	100.0000		0.000	21746454			

Enantioenriched 1-(3,3-Diallyl-5-chloroindolin-2-yl)propan-2-one from asymmetric Mannich reaction

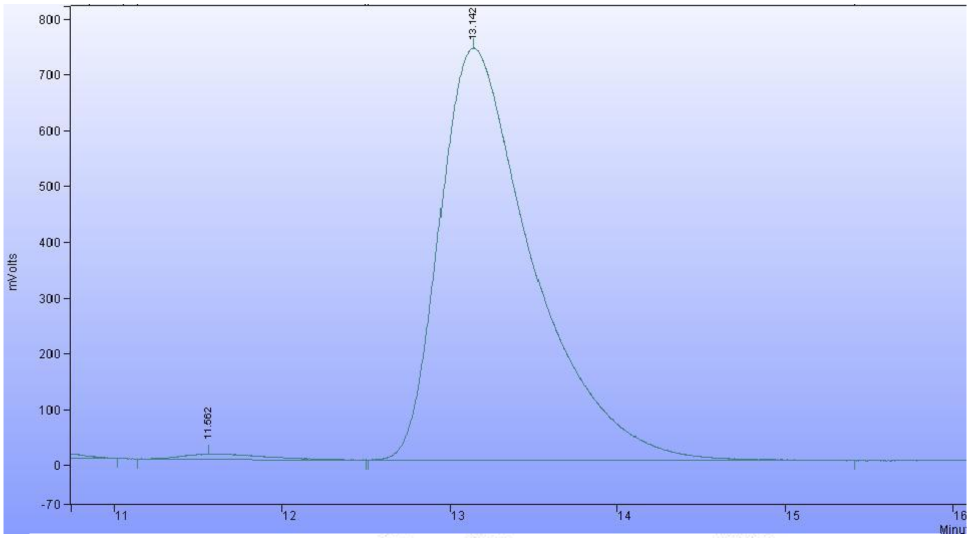

Peak No.	Peak Name	Result ()	Ret. Time (min)	Time Offset (min)	Area (counts)	Sep. Code	Width 1/2 (sec)	Status Codes
1		1.0440	11.562	0.000	301586	BB	27.7	
2		98.9560	13.142	0.000	28585526	BB	33.8	
	Totals:	100.0000		0.000	28887112			

(Rac)-1-(3,3-diallylindolin-2-yl)propan-2-one

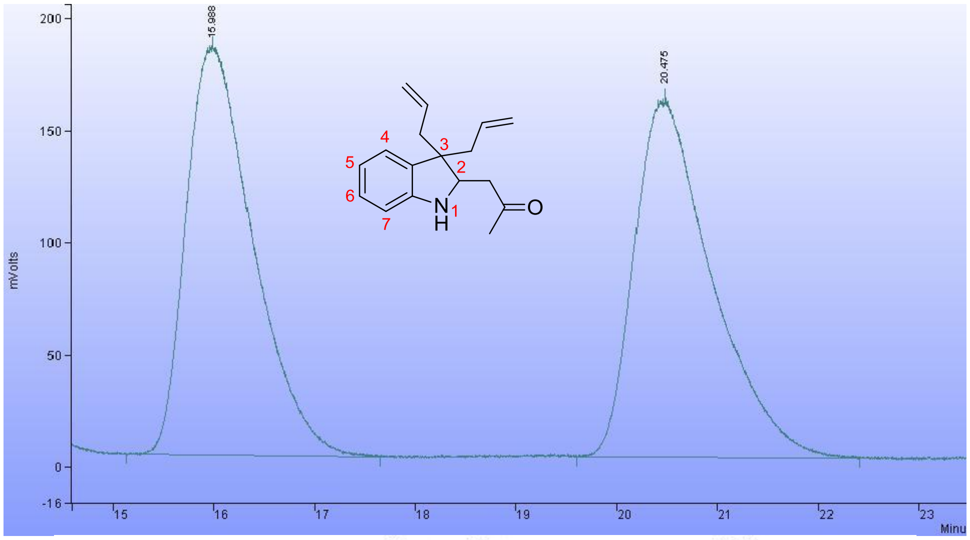

Peak No.	Peak Name	Result ()	Ret. Time (min)	Time Offset (min)	Area (counts)	Sep. Code	Width 1/2 (sec)	Status Codes
1		49.7419	15.988	0.000	8422639	BB	42.6	
2		50.2581	20.475	0.000	8510034	BB	47.7	
	Totals:	100.0000		0.000	16932673			

Enantioenriched 1-(3,3-diallylindolin-2-yl)propan-2-one from asymmetric Mannich reaction

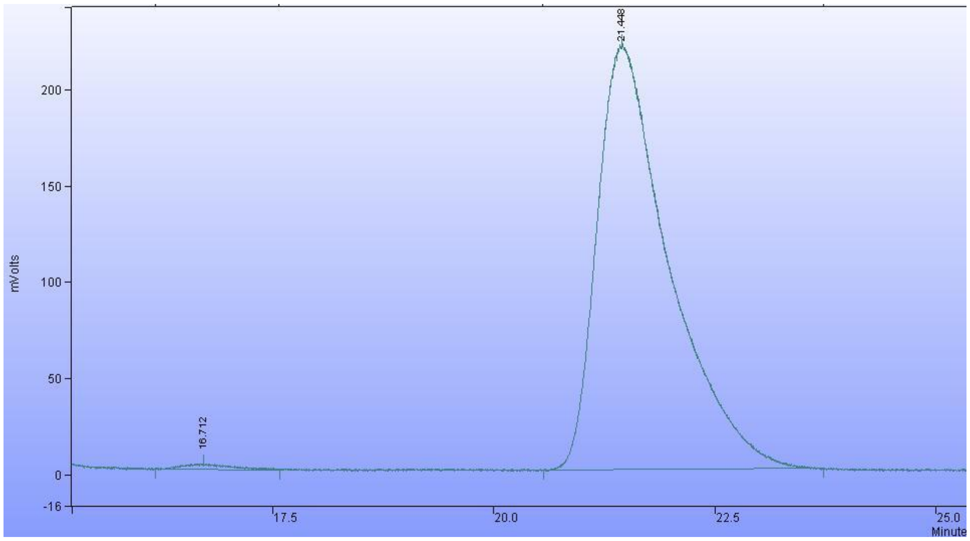

Supplement: Supplementary file 1 [file chem0020-13375-SD1.pdf]
